# Quantifying risks and interventions that have affected the burden of lower respiratory infections among children younger than 5 years: an analysis for the Global Burden of Disease Study 2017

**DOI:** 10.1016/S1473-3099(19)30410-4

**Published:** 2020-01

**Authors:** GBD 2017 Lower Respiratory Infections Collaborators, Christopher E Troeger, Ibrahim A Khalil, Brigette F Blacker, Molly H Biehl, Samuel B Albertson, Stephanie R M Zimsen, Puja C Rao, Degu Abate, Amha Admasie, Alireza Ahmadi, Mohamed Lemine Cheikh Brahim Ahmed, Chalachew Genet Akal, Fares Alahdab, Noore Alam, Kefyalew Addis Alene, Vahid Alipour, Syed Mohamed Aljunid, Rajaa M Al-Raddadi, Nelson Alvis-Guzman, Saeed Amini, Mina Anjomshoa, Carl Abelardo T Antonio, Jalal Arabloo, Olatunde Aremu, Hagos Tasew Atalay, Suleman Atique, Euripide F G A Avokpaho, Samah Awad, Ashish Awasthi, Alaa Badawi, Kalpana Balakrishnan, Joseph Adel Mattar Banoub, Aleksandra Barac, Quique Bassat, Neeraj Bedi, Derrick A Bennett, Krittika Bhattacharyya, Zulfiqar A Bhutta, Ali Bijani, Corey B Bills, Josip Car, Félix Carvalho, Carlos A Castañeda-Orjuela, Kate Causey, Devasahayam J Christopher, Aaron J Cohen, Lalit Dandona, Rakhi Dandona, Ahmad Daryani, Feleke Mekonnen Demeke, Shirin Djalalinia, Manisha Dubey, Eleonora Dubljanin, Eyasu Ejeta Duken, Maysaa El Sayed Zaki, Aman Yesuf Endries, Eduarda Fernandes, Florian Fischer, Joseph Frostad, Nancy Fullman, William M Gardner, Birhanu Geta, Keyghobad Ghadiri, Giuseppe Gorini, Alessandra C Goulart, Yuming Guo, Gessessew Bugssa Hailu, Arvin Haj-Mirzaian, Arya Haj-Mirzaian, Samer Hamidi, Hamid Yimam Hassen, Chi Linh Hoang, Nobuyuki Horita, Mihaela Hostiuc, Zakir Hussain, Seyed Sina Naghibi Irvani, Spencer L James, Ravi Prakash Jha, Jost B Jonas, André Karch, Amir Kasaeian, Tesfaye Dessale Kassa, Nicholas J Kassebaum, Adane Teshome Kefale, Yousef Saleh Khader, Ejaz Ahmad Khan, Gulfaraz Khan, Md Nuruzzaman Khan, Young-Ho Khang, Abdullah T Khoja, Ruth W Kimokoti, Adnan Kisa, Sezer Kisa, Niranjan Kissoon, Luke D Knibbs, Sonali Kochhar, Soewarta Kosen, Parvaiz A Koul, Ai Koyanagi, Barthelemy Kuate Defo, G Anil Kumar, Dharmesh Kumar Lal, Cheru Tesema Leshargie, Sonia Lewycka, Shanshan Li, Rakesh Lodha, Erlyn Rachelle King Macarayan, Marek Majdan, Abdullah A Mamun, Helena Manguerra, Varshil Mehta, Addisu Melese, Ziad A Memish, Desalegn Tadese Mengistu, Tuomo J Meretoja, Tomislav Mestrovic, Bartosz Miazgowski, Erkin M Mirrakhimov, Babak Moazen, Karzan Abdulmuhsin Mohammad, Shafiu Mohammed, Lorenzo Monasta, Catrin E Moore, Lidia Morawska, Jonathan F Mosser, Seyyed Meysam Mousavi, Srinivas Murthy, Ghulam Mustafa, Javad Nazari, Cuong Tat Nguyen, Huong Lan Thi Nguyen, Long Hoang Nguyen, Son Hoang Nguyen, Katie R Nielsen, Muhammad Imran Nisar, Molly R Nixon, Felix Akpojene Ogbo, Anselm Okoro, Andrew T Olagunju, Tinuke O Olagunju, Eyal Oren, Justin R Ortiz, Mahesh P A, Smita Pakhale, Maarten J Postma, Mostafa Qorbani, Reginald Quansah, Alireza Rafiei, Fakher Rahim, Vafa Rahimi-Movaghar, Rajesh Kumar Rai, Marissa Bettay Reitsma, Mohammad Sadegh Rezai, Aziz Rezapour, Maria Jesus Rios-Blancas, Luca Ronfani, Dietrich Rothenbacher, Salvatore Rubino, Zikria Saleem, Evanson Zondani Sambala, Abdallah M Samy, Milena M Santric Milicevic, Rodrigo Sarmiento-Suárez, Benn Sartorius, Miloje Savic, Monika Sawhney, Sonia Saxena, Alyssa Sbarra, Seyedmojtaba Seyedmousavi, Masood Ali Shaikh, Aziz Sheikh, Mika Shigematsu, David L Smith, Chandrashekhar T Sreeramareddy, Jeffrey D Stanaway, Mu'awiyyah Babale Sufiyan, Mohamad-Hani Temsah, Belay Tessema, Bach Xuan Tran, Khanh Bao Tran, Afewerki Gebremeskel Tsadik, Irfan Ullah, Rachel L Updike, Tommi Juhani Vasankari, Yousef Veisani, Fiseha Wadilo Wada, Yasir Waheed, Katie Welgan, Kirsten E Wiens, Charles Shey Wiysonge, Ebrahim M Yimer, Naohiro Yonemoto, Zoubida Zaidi, Heather J Zar, Stephen S Lim, Theo Vos, Ali H Mokdad, Christopher J L Murray, Hmwe Hmwe Kyu, Simon I Hay, Robert C Reiner

## Abstract

**Background:**

Despite large reductions in under-5 lower respiratory infection (LRI) mortality in many locations, the pace of progress for LRIs has generally lagged behind that of other childhood infectious diseases. To better inform programmes and policies focused on preventing and treating LRIs, we assessed the contributions and patterns of risk factor attribution, intervention coverage, and sociodemographic development in 195 countries and territories by drawing from the Global Burden of Diseases, Injuries, and Risk Factors Study 2017 (GBD 2017) LRI estimates.

**Methods:**

We used four strategies to model LRI burden: the mortality due to LRIs was modelled using vital registration data, demographic surveillance data, and verbal autopsy data in a predictive ensemble modelling tool; the incidence of LRIs was modelled using population representative surveys, health-care utilisation data, and scientific literature in a compartmental meta-regression tool; the attribution of risk factors for LRI mortality was modelled in a counterfactual framework; and trends in LRI mortality were analysed applying changes in exposure to risk factors over time. In GBD, infectious disease mortality, including that due to LRI, is among HIV-negative individuals. We categorised locations based on their burden in 1990 to make comparisons in the changing burden between 1990 and 2017 and evaluate the relative percent change in mortality rate, incidence, and risk factor exposure to explain differences in the health loss associated with LRIs among children younger than 5 years.

**Findings:**

In 2017, LRIs caused 808 920 deaths (95% uncertainty interval 747 286–873 591) in children younger than 5 years. Since 1990, there has been a substantial decrease in the number of deaths (from 2 337 538 to 808 920 deaths; 65·4% decrease, 61·5–68·5) and in mortality rate (from 362·7 deaths [330·1–392·0] per 100 000 children to 118·9 deaths [109·8–128·3] per 100 000 children; 67·2% decrease, 63·5–70·1). LRI incidence declined globally (32·4% decrease, 27·2–37·5). The percent change in under-5 mortality rate and incidence has varied across locations. Among the risk factors assessed in this study, those responsible for the greatest decrease in under-5 LRI mortality between 1990 and 2017 were increased coverage of vaccination against *Haemophilus influenza* type b (11·4% decrease, 0·0–24·5), increased pneumococcal vaccine coverage (6·3% decrease, 6·1–6·3), and reductions in household air pollution (8·4%, 6·8–9·2).

**Interpretation:**

Our findings show that there have been substantial but uneven declines in LRI mortality among countries between 1990 and 2017. Although improvements in indicators of sociodemographic development could explain some of these trends, changes in exposure to modifiable risk factors are related to the rates of decline in LRI mortality. No single intervention would universally accelerate reductions in health loss associated with LRIs in all settings, but emphasising the most dominant risk factors, particularly in countries with high case fatality, can contribute to the reduction of preventable deaths.

**Funding:**

Bill & Melinda Gates Foundation.

## Introduction

Lower respiratory infections (LRIs) are the leading infectious cause of death among children younger than 5 years globally, and mortality due to LRIs has declined substantially since the 1990s.^1^ Accelerating and maintaining these declines is essential to meeting Sustainable Development Goals for under-5 childhood mortality and ensuring that children everywhere have the opportunity to live a full, healthy life. Yet, no country has a national pneumonia control strategy and pneumonia attracts a small fraction of international development assistance and research and development funding.^2^ Several global initiatives have sought to fill this gap and provide guidance on the most efficient interventions to avert illness and mortality and to champion LRI as a preventable cause of death.^2^, ^3^, ^4^, ^5^ These programmes have typically categorised risk factors and interventions into groups that are defined by the stage in the morbidity pathway at which they occur, including protection against illness, prevention of infection, and treatment of disease.^4^, ^5^

**Research in context**

**Evidence before this study**

Lower respiratory infections (LRIs) haven previously been identified as the leading infectious cause of death among children younger than 5 years. Several prominent global burden estimation groups, the WHO Maternal and Child Epidemiology Estimation group, and the Global Burden of Diseases, Injuries, and Risk Factors Study (GBD) have iteratively quantified the morbidity and mortality associated with LRIs. Based on these findings, several initiatives have sought to give guidance about effective ways to reduce health loss due to LRIs, including the Global Action Plan for Pneumonia and Diarrhoea, The Missing Piece, and a 2013 *Lancet* Series about effective ways to reduce child mortality. We have previously published estimates of LRI mortality from GBD 2015 and 2016 and in those Articles, have looked at risks and interventions. We conducted a search in PubMed on April 30, 2019, using the search terms “(“lower respiratory infection” OR pneumonia) AND mortality AND global AND risk AND trend*)”. After removing publications using GBD results, we found 49 articles, many of which reported on single risk factors or countries. These manuscripts have been primarily cross-sectional and, to our knowledge, no other study has attempted to evaluate changes in LRI disease burden over time due to demographic changes and changes in risk factor exposure.

**Added value of this study**

Here we report findings from GBD 2017, which builds on previous iterations of GBD with additional data and modelling improvements. We use estimates for 13 risk factors or interventions for LRI morbidity or mortality, produced for GBD, to evaluate changes in LRI mortality among children younger than 5 years. We use a conceptual framework to group these risk factors into categories of those that primarily prevent initial LRI episodes (such as the pneumococcal conjugate vaccine) and those that primarily protect children with LRIs from dying (such as antibiotic therapy). A major component of GBD is producing internally consistent and externally comparable estimates for all locations and over time, which allows us to identify countries where the incidence or mortality has changed most rapidly and to evaluate the risk factors or interventions are most associated with these changes. We provide cross-sectional and longitudinal estimates of the reasons for which children are dying from LRIs, how this varies, and where specific interventions might have the greatest impact.

**Implications of all the available evidence**

The incidence and mortality due to LRIs among HIV-negative children younger than 5 years has declined in many parts of the world, particularly because of decreased exposure to household air pollution, reductions in prevalence of childhood wasting, and increased vaccine coverage. However, there is variation by country, suggesting that there is no single intervention that will substantially reduce LRI mortality in every country. Individual countries or regions must consider their specific context to identify strategies to reduce LRI disease burden. Our results, while being mindful of the limitations of modelled estimates, can help provide the evidence needed to develop plans to give children everywhere a chance at a life free from LRIs.

The decline in under-5 LRI mortality has not been universal and has varied between countries.^6^ Understanding why it declined faster in some countries than in others provides specific, actionable evidence to further reduce disease burden. The Global Burden of Diseases, Injuries, and Risk Factors Study 2017 (GBD 2017) is a systematic, scientific effort to quantify morbidity and mortality, including LRIs and their risk factors. We used results from GBD 2017 to assess which countries have performed best in reducing under-5 LRI mortality and compare countries on the basis of mortality rates, case fatality, and changes in risk factor exposure. This Article identifies countries where the change in under-5 LRI mortality has been largest, and uses the expansive set of estimates produced for GBD 2017 to analyse these changes, aiming to assess how and why they have occurred and to provide a roadmap for strategies to accelerate declines in mortality.

## Methods

### Overview

Detailed methods on GBD and on LRI estimation in GBD have been previously published.^1^, ^6^, ^7^, ^8^, ^9^ We describe these methods briefly. There were no substantial modelling changes between GBD 2016 and GBD 2017. LRIs are defined as diseases of the lower airways including pneumonia and bronchiolitis. Uncertainty in the LRI estimates are maintained through the modelling process using draws and is reflected as 2·5th and 97·5th percentiles of the posterior distribution. In compliance with the Guidelines for Accurate and Transparent Health Estimates Reporting, data and code for GBD 2017 are publicly available. There are four main components of the analysis that we share here: LRI mortality estimation; LRI morbidity estimation; estimation of LRI mortality attributable to the independent effects of risk factors; and an analysis of trends in LRI mortality.

### LRI mortality and morbidity estimation

Most causes of death in GBD 2017, including LRI, are modelled with the Cause of Death Ensemble model tool.^1^, ^10^ This statistical tool is designed to create a wide variety of models using a covariate selection algorithm and then to weight these models on the basis of their out-of-sample predictive validity. We combined these models into an ensemble that predicts LRI mortality by age, sex, year, and location from 1980 to 2017. The model for LRI used vital registration data, demographic surveillance data, and verbal autopsy data. Covariates included childhood growth failure, ambient and household air pollution, nutritional deficiency, Socio-Demographic Index (SDI), and maternal education, among others (appendix pp 6). Causes of death in the GBD study are mutually exclusive and each death has one cause. Importantly, any LRI death among people with HIV is considered to have HIV as the underlying cause of death, therefore our results represent LRI mortality among HIV-negative children younger than 5 years (appendix p 2).

The incidence and prevalence of LRI were modelled using DisMod-MR 2.1 (DisMod), a Bayesian meta-regression tool.^7^ One of the primary advantages of DisMod is that it enforces consistency between incidence, prevalence, recovery, and mortality by solving a series of ordinary differential equations. Input data for this model are from population-representative surveys, health-care utilisation records, and scientific literature. We used two covariates to help predict in areas with little or no data coverage: a composite indicator of the cumulative risk exposure for LRI, called the summary exposure variable and developed for GBD, and the SDI (appendix pp 9, 10).

### LRI trend analysis

We applied the results of the aforementioned models to spatiotemporal patterns. We compared estimates of LRI mortality and incidence in 1990 and 2017. To group countries into categories of similar burden, we identified country groupings on the basis of the burden in 1990. We split countries into four groups on the basis of the median mortality rate and incidence in 1990 and defined them as: high mortality and high incidence, high mortality and low incidence, low mortality and high incidence, and low mortality and low incidence.

### Case fatality ratio

The case fatality ratio is defined as the ratio of number of deaths to number of incident cases. We fit a log-normal regression using SDI to predict the expected change in LRI case fatality ratio. This was considered the baseline change in case fatality ratio that is explained by SDI.

### Risk factor attribution

Risk factors in GBD 2017 are causally related to LRI incidence or mortality.^8^ In this study, we analysed 13 of the risk factors for LRI identified in GBD 2017 (ambient air pollution, household air pollution, low *Haemophilus influenzae* type b [Hib] vaccine coverage, low pneumococcal conjugate vaccine [PCV] coverage, no handwashing, second-hand smoking, zinc deficiency, breastfeeding, low antibiotic coverage, low birthweight and short gestation, stunting, underweight, and wasting; appendix pp 13–15). The estimation strategy for risk factors involved a counterfactual approach that quantifies the level of exposure to the risk factor in a population and the relative risk of LRI given exposure. Typically, the exposure in a population is modelled on the basis of surveys and scientific literature and the risk of LRI is derived from published meta-analyses. Childhood growth failure risks were estimated as a continuous exposure of the height or weight Z scores. Likewise, air pollution was considered a continuous exposure of the amount of fine particulate matter smaller than 2·5 μm in diameter. Other risk factors, such as low vaccine coverage, are modelled when the exposure is a population prevalence of being exposed to that risk factor (eg, the population prevalence of being unvaccinated for low vaccine coverage). Descriptions of the risk-factor exposure models and relative risks are provided in the appendix (pp 13–66). Risk factors in GBD are part of a comparative risk assessment framework and are modelled independently.^8^ Therefore, in our study, the burden associated with each risk factor can be considered as the LRI mortality that could be averted if exposure to that risk factor was eliminated. Since they were modelled independently, our analysis does not quantify the potential impact of combined interventions and combining risk-factor burden by summing risk factors is not appropriate and could lead to greater attribution than disease burden.

### Intervention efficiency assessment

To assess the efficiency of targeted interventions for each risk factor among children younger than 5 years, we took advantage of the counterfactual definition of risk-factor burden such that the LRI mortality rate attributable to each risk factor was equivalent to the reduction expected given complete absence of the risk factor.^8^ For example, for vaccines, the risk exposure was defined as no vaccination, so the counterfactual was full vaccine coverage.

We classified risk factors into two categories based on their biological mechanism of risk and modelled after a conceptualisation proposed by the Global Action Plan for the Prevention and Control of Pneumonia and Diarrhoea.^3^ Conceptually, prevention risks are those that increase the probability of developing a LRI and include ambient air pollution, household air pollution, low Hib vaccine coverage, low PCV coverage, no access to a handwashing station with soap and water, second-hand smoke exposure, and zinc deficiency (appendix p 12). Protection risks are those that increase the probability of dying once a child developed an LRI and include suboptimal breastfeeding, low antibiotic coverage, low birthweight and short gestation, childhood stunting, childhood underweight, and childhood wasting (appendix p 13–15). We decomposed the effect of the change in exposure to each risk factor on the LRI mortality rate between 1990 and 2017, accounting for the independent effects of population growth, population ageing, and other drivers of LRI mortality. This process has been described in detail elsewhere.^6^, ^8^

### Role of the funding source

The funder of the study played no role in study design, data collection, data analysis, data interpretation, or writing of the report. All collaborators had full access to all the data in the study and the corresponding author had final responsibility for the decision to submit for publication.

## Results

Globally, LRIs were the leading infectious cause of death among children younger than 5 years in 2017 (808 920 deaths, 95% uncertainty interval [UI] 747 286–873 591; table), responsible for 15·0% (14·0–16·0) of all under-5 deaths. There was no substantial difference in the under-5 LRI mortality rate between boys (118·2 deaths, 108·2–129·6, per 100 000 boys) and girls (119·5 deaths, 109·6–129·6, per 100 000 girls; data are available on GBD-Compare). Since 1990, there has been a substantial decrease in the number of deaths (65·4% decrease, 61·5–68·5; from 2 337 538 deaths to 808 920 deaths), the mortality rate (67·2% decrease, 63·6–70·2; from 362·7 deaths, 330·1–392·0, per 100 000 children to 118·9 deaths, 109·8–128·3, per 100 000 children; table), and the percent of under-5 deaths that were due to LRIs (24·6% decrease, 17·2–30·4; from 19·9%, 18·1–21·4, to 15·0%, 14·0–16·0) among children younger than 5 years.

**Table:** Deaths and case fatality attributable to and incidence of lower respiratory infections among children younger than 5 years by Global Burden of Diseases, Injuries, and Risk Factors Study regions and super-regions, 2017

	**Deaths (95% UI)**	**Mortality rate per 100 000 (95% UI)**	**Percentage change mortality rate (95% UI), 1990–2017**	**Incidence per 100 000 (95% UI)**	**Percentage incidence change (95% UI), 1990–2017**	**Case fatality ratio (95% UI)**	**Attributable fraction for all risks (95% UI)**	**Attributable fraction for prevention-associated risks (95% UI)**	**Attributable fraction for protection-associated risks (95% UI)**
**Global**	**808 920 (747 286 to 873 591)**	**118·9 (109·8 to 128·3)**	**–67·2% (−70·2 to −63·6)**	**12 197·8 (9 762·1 to 14 908·7)**	**–32·4% (−37·5 to −27·2)**	**1·0% (0·9 to 1·1)**	**93·4% (90·3 to 95·7)**	**65·2% (50·2 to 77·6)**	**82·0% (62·6 to 92·1)**
**Central Europe, eastern Europe, and central Asia**	**16 040 (14 296 to 18 051)**	**57·3 (51·0 to 64·4)**	**–66·8% (−70·7 to −62·3)**	**9 219·6 (7 103·3 to 11 586·8)**	**–36·3% (−43·2 to −28·6)**	**0·6% (0·6 to 0·7)**	**84·5% (77·5 to 89·7)**	**47·8% (33·5 to 62·2)**	**75·8% (53·6 to 88·4)**
Central Asia	13 937 (12 246 to 15 922)	145·4 (127·7 to 166·1)	–69·9% (−73·8 to −65·2)	6 206·9 (5 003·0 to 7 601·2)	–46·9% (−53·8 to −39·2)	2·3% (2·2 to 2·6)	85·1% (78·1 to 90·1)	47·5% (32·6 to 62·2)	77·2% (54·6 to 89·5)
Central Europe	707 (632 to 795)	12·5 (11·2 to 14·1)	–84·2% (−86·1 to −82·1)	8 700·5 (6 862·8 to 10 868·4)	–26·9% (−34·5 to −18·2)	0·1% (0·1 to 0·2)	80·5% (71·7 to 87·6)	53·0% (39·9 to 66·1)	71·4% (47·0 to 85·5)
Eastern Europe	1 396 (1 277 to 1 509)	10·9 (10·0 to 11·8)	–78·2% (−80·2 to −76·5)	11 710·4 (8 712·6 to 15 012·1)	–32·5% (−41·5 to −23·2)	0·1% (0·1 to 0·1)	81·2% (72·5 to 87·8)	49·0% (34·2 to 63·1)	72·6% (50·7 to 85·1)
**High income**	**1 857 (1 702 to 2 027)**	**3·2 (2·9 to 3·5)**	**–70·6% (−73·2 to −67·9)**	**4 843·7 (3 772·5 to 6 137·4)**	**–19·5% (−24·9 to −13·4)**	**0·1% (0·1 to 0·1)**	**67·7% (55·8 to 78·1)**	**28·8% (16·0 to 44·7)**	**66·5% (42·0 to 82·1)**
Australasia	42 (32 to 53)	2·3 (1·8 to 2·9)	–65·4% (−76·2 to −53·4)	5 798·0 (4 448·5 to 7 449·4)	2·2% (−6·7 to 11·5)	0·0% (0·0 to 0·0)	66·6% (53·8 to 78·1)	23·0% (10·1 to 41·9)	65·8% (40·8 to 82·1)
High-income Asia Pacific	180 (163 to 197)	2·4 (2·2 to 2·6)	–72·2% (−75·9 to −68·4)	8 472·0 (6 595·5 to 10 872·2)	–3·9% (−14·8 to 8·2)	0·0% (0·0 to 0·0)	75·3% (63·3 to 84·6)	33·3% (20·9 to 48·9)	70·5% (43·5 to 85·9)
High-income North America	684 (618 to 742)	3·2 (2·9 to 3·5)	–60·6% (−65·9 to −56·2)	4 791·2 (3 679·0 to 6 155·8)	–29·6% (−33·9 to −25·3)	0·1% (0·1 to 0·1)	57·1% (43·7 to 70·2)	21·0% (9·1 to 37·6)	61·0% (36·9 to 78·4)
Southern Latin America	568 (466 to 696)	11·1 (9·1 to 13·6)	–77·5% (−82·0 to −72·3)	11 895·3 (9 444·5 to 14 789·3)	–11·2% (−23·8 to 2·8)	0·1% (0·1 to 0·1)	78·3% (67·7 to 86·7)	37·1% (19·7 to 56·4)	73·2% (48·2 to 87·0)
Western Europe	383 (350 to 426)	1·7 (1·6 to 1·9)	–72·0% (−75·6 to −68·8)	1 940·4 (1 526·6 to 2 431·0)	–19·1% (−24·6 to −13·2)	0·1% (0·1 to 0·1)	67·4% (55·2 to 78·1)	28·1% (16·0 to 43·6)	64·5% (40·9 to 80·1)
**Latin America and Caribbean**	**21 606 (19 618 to 24 079)**	**42·4 (38·5 to 47·3)**	**–79·1% (−81·9 to −75·8)**	**12 192·4 (9 920·3 to 14 782·1)**	**–37·4% (−42·4 to −31·9)**	**0·3% (0·3 to 0·4)**	**78·8% (70·5 to 85·9)**	**44·5% (30·5 to 58·4)**	**72·4% (45·9 to 87·8)**
Andean Latin America	3 787 (2 988 to 4 694)	56·5 (44·6 to 70·0)	–87·0% (−90·0 to −83·3)	16 610·1 (14 120·4 to 19 324·6)	–40·3% (−46·5 to −33·0)	0·3% (0·3 to 0·4)	74·7% (64·8 to 83·0)	40·0% (23·0 to 57·9)	68·6% (40·4 to 86·2)
Caribbean	3 932 (2 985 to 5 131)	100·5 (76·3 to 131·2)	–51·8% (−63·9 to −35·7)	11 164·6 (8 986·4 to 13 596·2)	–9·9% (−18·2 to 0·5)	0·9% (0·8 to 1·0)	89·3% (84·1 to 93·2)	68·9% (53·4 to 81·9)	76·7% (52·9 to 90·1)
Central Latin America	9 257 (8 062 to 10 826)	38·3 (33·3 to 44·7)	–73·6% (−77·4 to −68·5)	15 259·9 (12 336·1 to 18 680·2)	–39·9% (−45·4 to −33·9)	0·3% (0·2 to 0·3)	78·9% (70·7 to 85·6)	41·8% (26·9 to 56·8)	72·5% (45·2 to 88·0)
Tropical Latin America	4 630 (4 163 to 5 158)	28·8 (25·9 to 32·0)	–85·8% (−88·6 to −83·7)	5 990·7 (4 896·3 to 7 296·4)	–45·2% (−49·3 to −40·9)	0·5% (0·4 to 0·5)	73·2% (62·3 to 82·7)	28·0% (17·2 to 41·5)	70·6% (41·9 to 87·4)
**North Africa and Middle East**	**43 558 (37 550 to 49 735)**	**67·7 (58·3 to 77·3)**	**–76·5% (−80·7 to −71·1)**	**19 258·4 (15 414·9 to 23 501·0)**	**–25·6% (−32·2 to −19·0)**	**0·4% (0·3 to 0·4)**	**91·9% (87·6 to 94·9)**	**62·3% (46·8 to 75·6)**	**81·1% (58·5 to 92·3)**
**South Asia**	**249 595 (225 643 to 275 313)**	**143·1 (129·4 to 157·9)**	**–71·2% (−74·9 to −66·7)**	**13 153·1 (10 465·5 to 16 238·0)**	**–22·7% (−28·7 to −16·2)**	**1·1% (1·0 to 1·2)**	**95·9% (93·9 to 97·4)**	**65·4% (50·5 to 77·3)**	**83·2% (66·8 to 92·1)**
**Southeast Asia, east Asia, and Oceania**	**63 661 (58 190 to 69 821)**	**45·0 (41·1 to 49·3)**	**–85·7% (−87·2 to −83·7)**	**13 383·7 (10 686·7 to 16 401·7)**	**–38·8% (−44·3 to −32·3)**	**0·3% (0·3 to 0·4)**	**88·7% (83·6 to 92·8)**	**61·2% (45·3 to 75·0)**	**78·8% (56·2 to 90·8)**
East Asia	22 824 (20 743 to 25 438)	27·1 (24·6 to 30·2)	–90·7% (−91·9 to −89·2)	9 376·4 (7 387·3 to 11 625·0)	–54·8% (−59·6 to −49·2)	0·3% (0·3 to 0·3)	83·2% (76·5 to 88·9)	61·5% (41·9 to 77·8)	70·9% (46·3 to 85·7)
Oceania	1 770 (1 295 to 2 325)	99·5 (72·8 to 130·7)	–48·1% (−63·1 to −26·9)	16 573·7 (13 249·1 to 20 596·4)	–12·5% (−22·0 to −1·8)	0·6% (0·5 to 0·6)	93·1% (89·9 to 95·6)	68·3% (50·0 to 82·7)	85·2% (64·2 to 94·7)
Southeast Asia	39 066 (34 532 to 44 291)	70·2 (62·1 to 79·6)	–80·7% (−83·5 to −77·1)	19 344·3 (15 535·4 to 23 655·9)	–20·7% (−27·5 to −13·0)	0·4% (0·3 to 0·4)	91·7% (87·5 to 94·8)	61·2% (46·0 to 74·3)	82·6% (61·1 to 93·1)
**Sub-Saharan Africa**	**412 604 (357 299 to 471 442)**	**252·5 (218·7 to 288·6)**	**–62·9% (−67·6 to −56·8)**	**10 493·2 (8 558·0 to 12 858·7)**	**–34·5% (−39·0 to −29·4)**	**2·4% (2·2 to 2·6)**	**94·0% (90·9 to 96·2)**	**68·0% (50·6 to 81·8)**	**82·8% (61·9 to 93·2)**
Central sub-Saharan Africa	47 357 (37 232 to 58 184)	239·7 (188·4 to 294·5)	–61·8% (−69·9 to −51·0)	11 728·4 (9 490·0 to 14 347·2)	–28·4% (−36·3 to −19·7)	2·0% (2·0 to 2·1)	94·2% (91·0 to 96·4)	68·5% (48·8 to 83·9)	82·9% (58·6 to 94·3)
Eastern sub-Saharan Africa	111 613 (99 529 to 124 670)	176·3 (157·2 to 196·9)	–71·2% (−75·4 to −65·6)	12 894·4 (10 363·6 to 15 813·8)	–33·7% (−38·7 to −28·6)	1·4% (1·2 to 1·5)	93·3% (90·0 to 95·7)	66·7% (51·4 to 78·4)	81·3% (59·0 to 92·6)
Southern sub-Saharan Africa	10 513 (9 192 to 12 063)	123·1 (107·7 to 141·3)	–54·7% (−61·5 to −46·6)	7 357·1 (6 032·7 to 8 847·9)	–30·3% (−35·5 to −24·5)	1·7% (1·6 to 1·8)	87·4% (81·5 to 92·1)	51·9% (36·5 to 66·3)	77·0% (51·0 to 90·7)
Western sub-Saharan Africa	243 122 (198 471 to 290 155)	338·7 (276·5 to 404·3)	–60·2% (−67·1 to −50·7)	8 408·3 (6 875·4 to 10 219·7)	–37·7% (−42·7 to −31·7)	4·0% (4·0 to 4·0)	94·5% (91·7 to 96·5)	69·3% (49·0 to 84·9)	83·8% (63·7 to 93·8)

Estimates for every country are available in the appendix (pp 67–92). UI=uncertainty interval.

**Figure 1:**
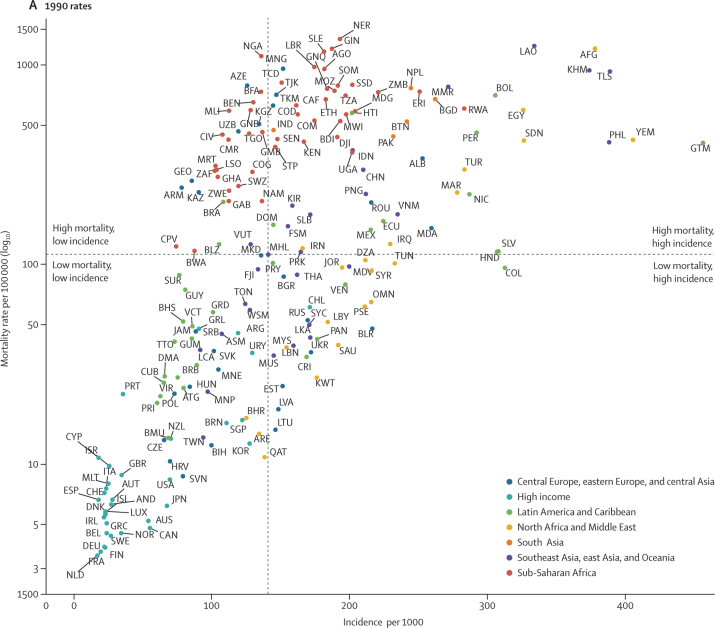

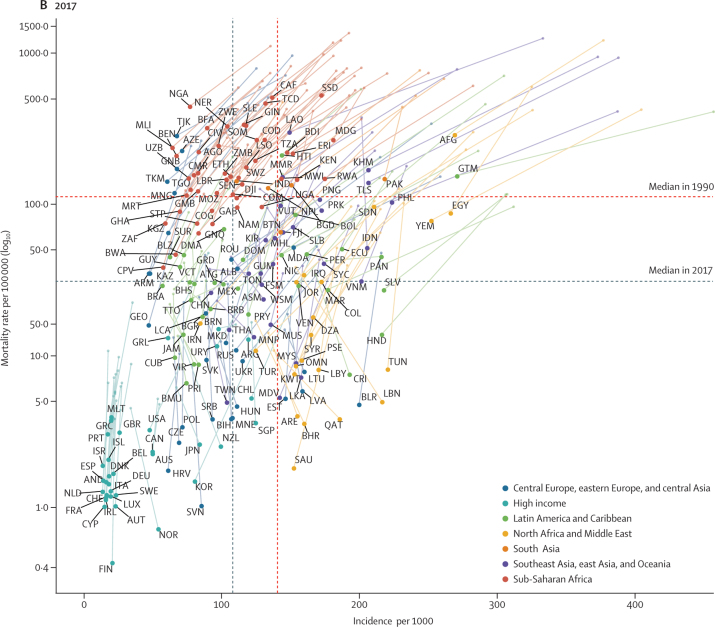
Under-5 LRI incidence and mortality rates in 1990 (A) and 2017 (B) Points represent countries (labelled according to the International Organization for Standardization 3166 alpha-3 codes) and the colour indicates the Global Burden of Diseases, Injuries, and Risk Factors Study super-region each of them belongs to. The vertical line indicates the median incidence among all countries and the horizontal line indicates the median mortality rate among all countries. The plots are therefore divided into four quadrants based on each country's relative incidence and mortality rate compared with all other countries in 1990 and in 2007.

**Figure 2:**
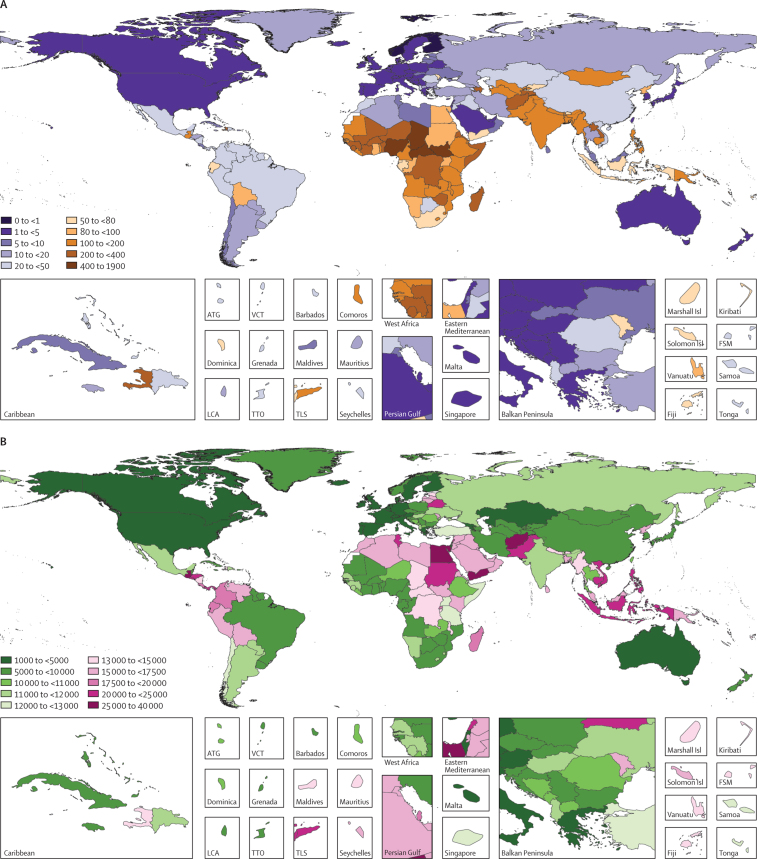

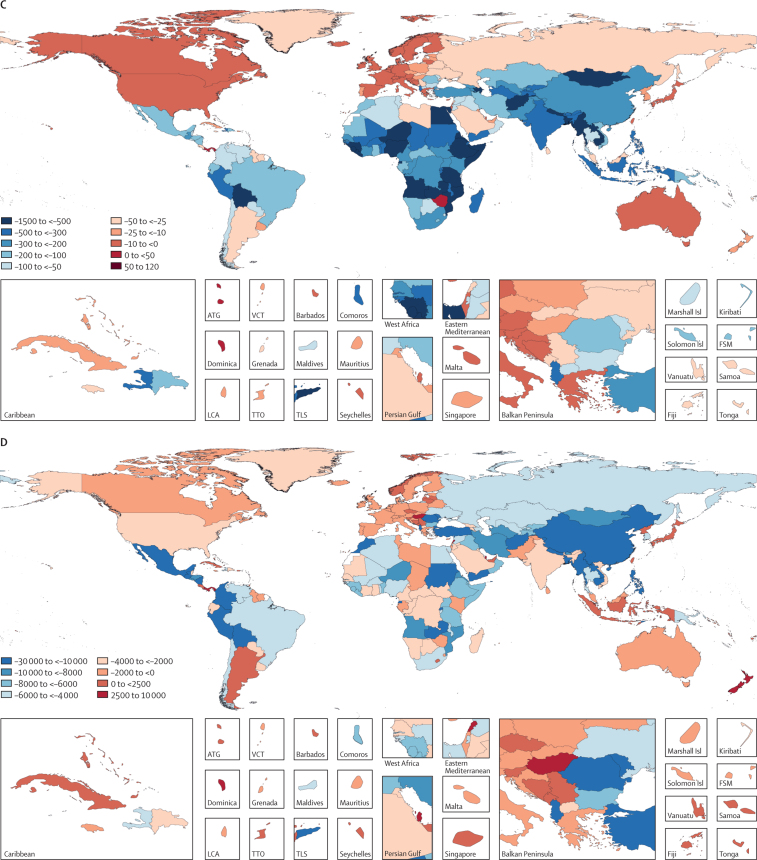
Global distribution of LRI burden among children younger than 5 years (A) Under-5 LRI mortality rate in 2017; (B) LRI incidence per 100 000 child-years in 2017; (C) absolute difference in LRI mortality rate between 1990 and 2017; and (D) absolute difference in LRI incidence rate between 1990 and 2017. ATG=Antigua and Barbuda. FSM=Federated States of Micronesia. Isl=Islands. LCA=Saint Lucia. LRI=lower respiratory infection. TLS=Timor-Leste. TTO=Trinidad and Tobago. VCT=Saint Vincent and the Grenadines.

Most under-5 LRI deaths in 2017 occurred in India (185 429 deaths, 95% UI 167 676–204 328), Nigeria (153 069 deaths, 115 332–196 193), and Pakistan (40 480 deaths, 28 805–57 002; appendix pp 67–92). The highest LRI mortality rate occurred in South Sudan (527·7 deaths, 386·2–707·5, per 100 000 children; Figure 1, Figure 2; appendix pp 67–92). Likewise, reductions in LRI mortality rates have varied by location: Turkey (96·4% decline, 94·4–97·6) declined at the fastest rate whereas Niger experienced the largest absolute reduction in under-5 LRI mortality rate, from the highest mortality rate globally in 1990 (1349·0 deaths, 1027·0–1714·3, per 100 000 children) to 329·7 deaths (231·0–451·6) per 100 000 children in 2017 (ie, 1019·3 fewer deaths, 796·0–1262·7, per 100 000 children; Figure 1, Figure 2; appendix pp 67–92). Between 1990 and 2005, the fastest annualised rate of change in LRI mortality rate occurred in Oman (14·9% decrease per year) and the fastest annualised rate of change between 2000 and 2017 occurred in Saudi Arabia (12·7% decrease per year; data not shown, available on GBD-Compare).

The global LRI incidence among children younger than 5 years was 12 197·8 new cases (95% UI 9762·1–14 908·7) per 100 000 child-years. LRI incidence was highest in Guatemala (27 126·3 new cases, 22 443·4–32 304·3, per 100 000 child-years; Figure 1, Figure 2; appendix pp 67–92). LRI incidence declined globally (from 18 054·0 new cases, 14 808·2–21 833·2, per 100 000 child-years, in 1990; 32·4% decrease, 27·2–37·5) with the fastest declines in Turkmenistan (58·0% decrease, 50·9–64·5), Mongolia (56·6% decrease, 48·9–63·9), and China (56·0% decrease, 50·5–60·6; figure 1; appendix pp 67–92). However, the incidence of LRI increased in some locations such as Norway (58·9% increase, 44·4–75·1; from 3406·1 new cases, 2659·4–4294·7, per 100 000 child-years to 5411·7 new cases, 4115·6–7016·1, per 100 000 child-years) and Lebanon (40·8% increase, 21·0–59·1; from 15 400·6 new cases, 12 300·8–19 254·4, per 100 000 child-years to 21 680·6 new cases, 16 166·6–28 291·3, per 100 000 child-years; Figure 1, Figure 2; appendix pp 67–92). Additional results by age, sex, location, and year from 1990 to 2017 are available on GBD-Compare.

**Figure 3:**
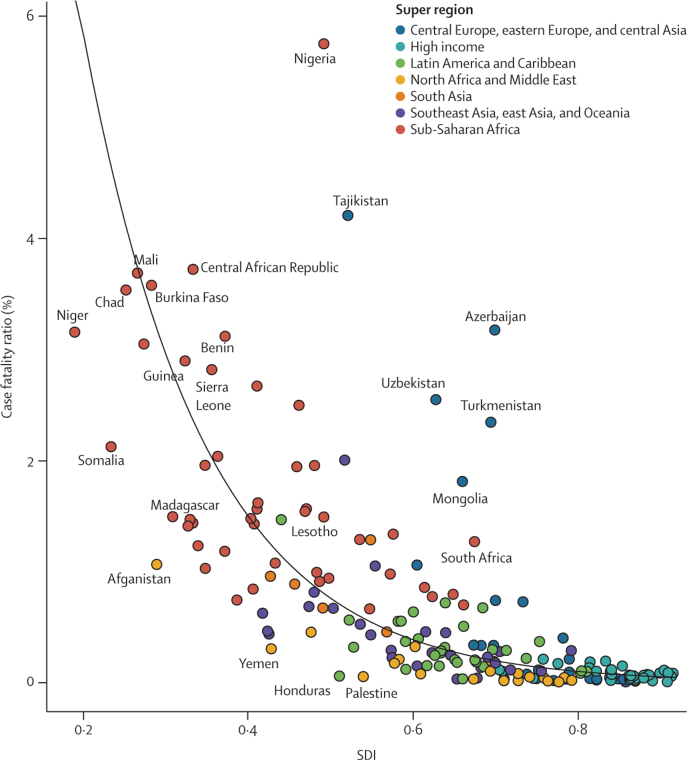
Case fatality ratio among children under-5 in 2017 We used the Socio-demographic Index as a predictor of the case fatality ratio by country. The solid black line indicates a log-linear curve for these values.

The global case fatality ratio for LRIs decreased from 2·0% (2·2–1·8) in 1990, to 1·0% (95% UI 0·9 to 1·1) in 2017. In 2017, the lowest case fatality ratios globally occurred in Saudi Arabia (<0·1%, <0·1 to <0·1) and Slovenia (<0·1%, <0·1 to <0·1), whereas the highest occurred in Nigeria (5·8%, 5·4 to 6·0) and Tajikistan (4·2%, 4·2 to 4·3; figure 3; appendix pp 67–92). In 2017, if all countries with a case fatality ratio exceeding the global average had been reduced to the global average, then 291 611 deaths would be averted. Some countries in central Asia (eg, Azerbaijan, Mongolia, and Tajikistan) and in western sub-Saharan Africa (eg, Guinea, Nigeria, and Sierra Leone) had case fatality ratios much higher than expected on the basis of SDI alone (figure 3). If these countries had experienced case fatality ratios corresponding with the average relationship between case fatality ratio and SDI, an additional 326 900 deaths, including 133 600 in Nigeria, could possibly have been averted in 2017.

Overall, 93·4% (95% UI 90·3 to 95·7) of under-5 LRI mortality could be attributed to risk factors and interventions modelled by GBD in 2017 (table). Because of the counterfactual strategy in risk factor attribution, this suggests that 755 513 under-5 LRI deaths (691 459 to 819 746) would have been avertable if exposures to all risk factors had been reduced to their theoretical minimum levels. Risk factors in the GBD study are not mutually exclusive and so individual attributable fractions might sum to more than 100%. Protection-related risk factors were responsible for 82·0% (62·6 to 92·1) of under-5 LRI deaths in 2017 (table), including 52·6% (35·1 to 62·8) of under-5 LRI deaths attributable to wasting, 14·7% (1·6 to 34·7) to stunting, 11·5% (7·6 to 20·4) to underweight, and 7·4% (4·2 to 11·1) to non-exclusive breastfeeding (data available on GBD-Compare). Interventions to prevent risk exposure could have averted 65·2% (50·2 to 77·6) of under-5 LRI deaths in 2017 (table) including 11·2% (7·3 to 14·8) of deaths attributable to insufficient handwashing with soap, 28·5% (22·4 to 34·1) to household air pollution, 17·5% (13·2 to 22·6) to ambient particulate matter pollution, 19·2% (16·5 to 21·8) to low PCV coverage, and 9·6% (<0·1 to 20·6) due to low Hib vaccine coverage (data available on GBD-Compare).

**Figure 4:**
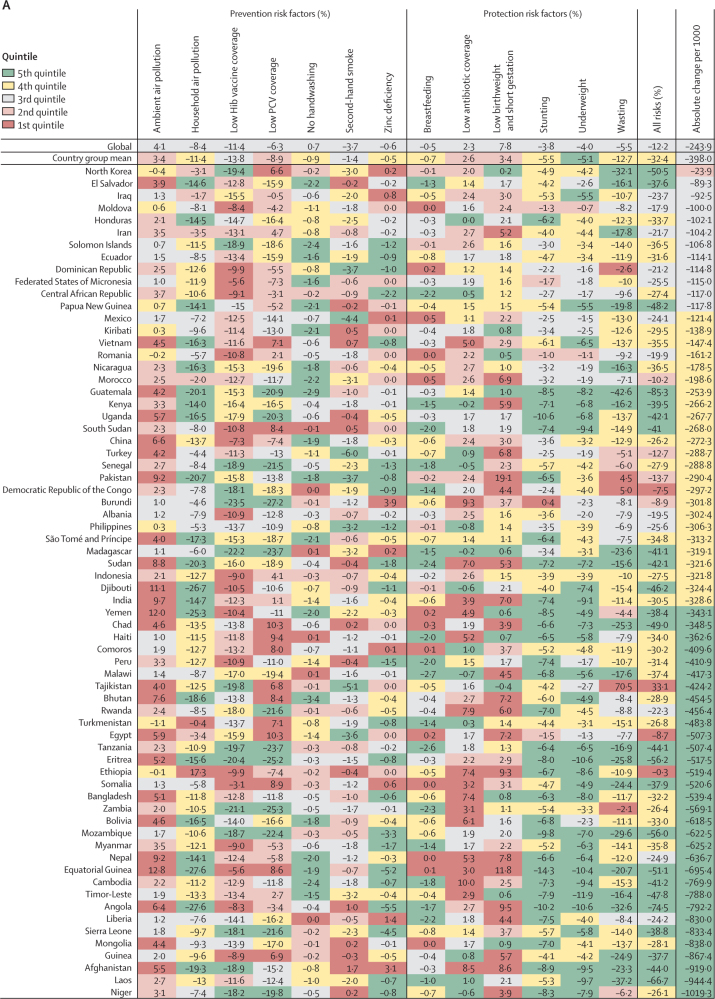

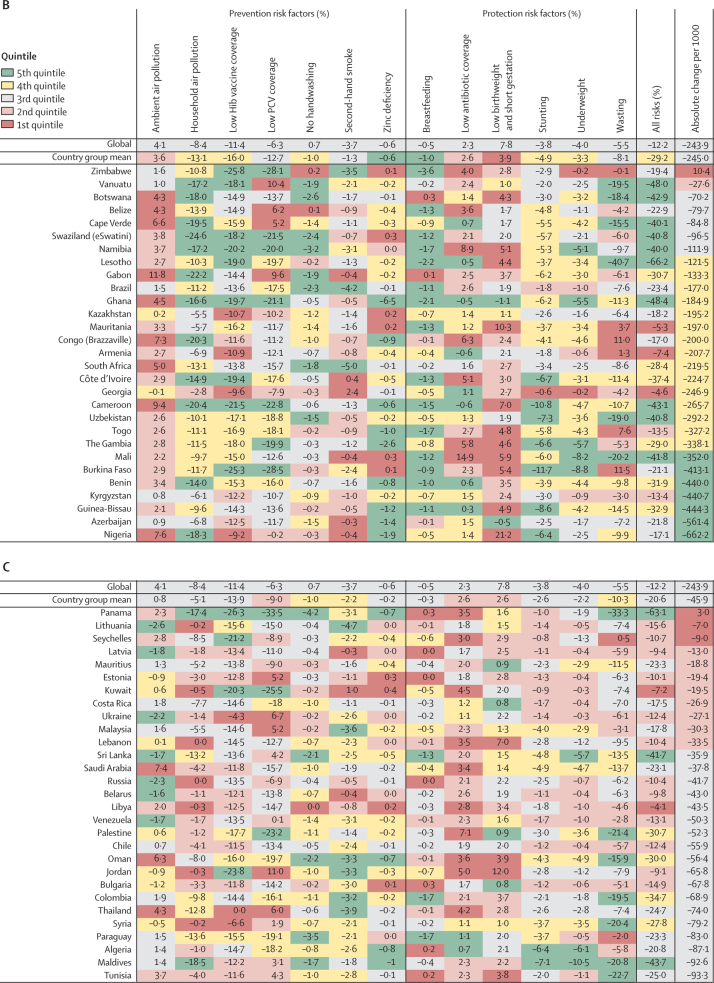

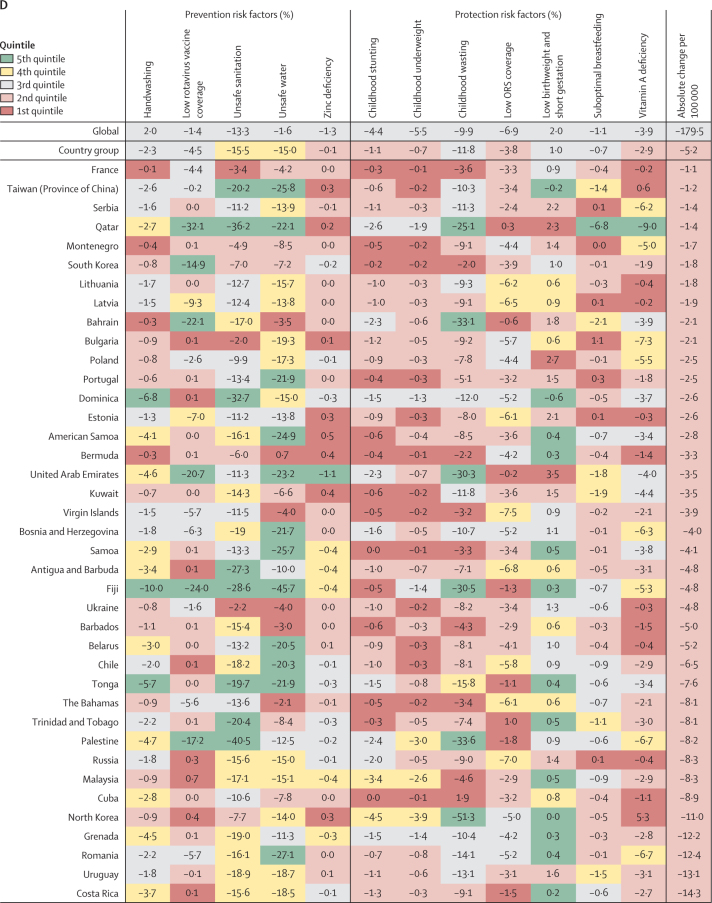
Change in LRI mortality rate attributable to changes in risk factor exposure by country, 1990–2017 Countries are grouped by their mortality and incidence (higher or lower than the global median in 1990, as identified by the quandrants in figure 1A) and are ordered within each group from slowest absolute change in under-5 LRI mortality rate per 1000 children between 1990 and 2017. Colors indicate the quintile for the absolute change in each risk factor attributable fraction among all countries. Country groupings are: (A) high mortality, high incidence (n=68); (B) high mortality, low incidence (n=29); (C) low mortality, high incidence (n=29); and (D) low mortality, low incidence (n=69). Hib=*Haemophilus influenzae* type b. PCV=pneumococcal conjugate vaccine.

At the global level, changes to all risk factors for LRI mortality accounted for a 12·2% decrease (95% UI 11·6–13·1) between 1990 and 2017 (figure 4; appendix pp 93–111). Globally, increased Hib vaccine coverage (11·4%, 0·0–24·5) and PCV coverage (6·3%, 6·1–6·3) were responsible for large decreases in LRI mortality among children younger than 5 years between 1990 and 2017 (figure 4; appendix pp 93–111). This effect was evident also in all subgroups of countries classified according to their mortality and incidence rates in 1990. Although decreased exposure to household air pollution reduced LRI mortality by 8·4% (6·8–9·2), increased exposure to ambient air pollution increased mortality by 4·1% (2·7–6·2; figure 4).

In 1990, both the mortality and incidence rates were higher than the corresponding country-group mean values in 68 countries, which were categorised as high mortality and high incidence (upper right quadrant of figure 1A; figure 4A). From 1990 to 2017, the under-5 LRI mortality rate declined by a greater amount than the global median in 50 (74%) of these 68 countries and the LRI mortality rate decreased by a mean of 398·0 deaths (95% UI 100·7–857·8) per 100 000 children in these countries (Figure 1, Figure 4). These countries tended to have large decreases in LRI mortality attributable to changes in childhood growth failure indicators, including a mean 12·7% (2·3–31·2) reduction due to childhood wasting, 5·5% (1·5–9·5) reduction due to childhood stunting, and 5·1% (1·6–10·2) reduction due to childhood underweight (figure 4A). Among the countries with the greatest magnitude change, childhood stunting accounted for a 14·3% (2·5–28·9) decrease in LRI mortality rate in Equatorial Guinea and a 10·6% (2·0–22·8) decrease in Uganda. Childhood underweight accounted for an 11·9% (7·5–19·7) decrease in LRI mortality rate in Timor-Leste and a 10·6% (6·8–18·0) decrease in Angola. Changes in childhood wasting were responsible for a 42·6% (26·3–55·3) decrease in LRI mortality rate in Guatemala and a 37·2% (29·0–42·2) decrease in Laos (figure 4A). The greatest absolute decline in LRI mortality rate occurred in Niger and the vaccine-related risk factors were responsible for the largest decrease in LRI mortality (18·2% [0·0–37·3] decrease due to increased Hib vaccine coverage and 19·8% [18·6–20·0] decrease due to increased PCV coverage). Some countries in this group had large reductions in LRI mortality due to household air pollution (27·6% [20·4–33·3] reduction in Angola) and vaccine coverage (23·5% [0·0–51·0] decline due to increased Hib vaccine coverage and 27·2% [23·0–30·5] decline due to increased PCV coverage in Burundi). The LRI mortality rate increased due to ambient air pollution in 64 (94%) of 68 countries in this group (median increase 3·4% [0·0–9·5]; figure 4A; appendix pp 93–111).

In 29 countries, the mortality rate was higher than the global median but the incidence was lower than the global median in 1990. We classified these countries as having high mortality and low incidence (upper left quadrant of figure 1; figure 4B). This group of countries had a mean decline in LRI mortality rate of 245·0 deaths (44·7–514·6) per 100 000 children during 1990–2017. Countries in this group tended to have large reductions in LRI mortality attributable to changes in household air pollution (mean decrease 13·1% [5·6–21·5]), including a 24·6% (17·9–30·6) decline in Swaziland (eSwatini). Increased Hib vaccine coverage also contributed to a substantial reduction in LRI mortality in this group of countries (16·0% decrease [10·0–23·8]). This group also had small declines in LRI mortality attributable to improved breastfeeding (mean 1·0% [0·0–2·2]) and zinc deficiency (mean 0·6% [0·2–2·3]). The LRI mortality rate decreased by 662·2 deaths (554·7–755·6) per 100 000 children in Nigeria, where the largest attributable changes were due to household air pollution (18·3% decrease [11·9–22·9]), childhood wasting (9·9% decrease [8·5–11·1]), and childhood stunting (6·4% decrease [1·4–13·5]; figure 4B; appendix pp 93–111).

In 29 countries, the mortality rate was lower than the global median in 1990 but the incidence was higher than the global median. We classified these countries as having low mortality and high incidence (lower right quadrant of figure 1; figure 4C). The LRI mortality rate decreased by 45·9 deaths (95% UI 7·8–90·4) per 100 000 children in these countries and the absolute change in the LRI mortality rate was in the 3rd quintile for 18 (62%) of 29 countries. Relative to other groups, this group of countries had greater reductions attributable to behavioural risk factors such as no handwashing (1·0% mean decline [0·2–3·0]), second-hand smoke exposure (2·2% mean decline [0·3–3·7]), and childhood wasting (10·3% mean decline [2·3–22·2]). Although vaccine coverage reduced LRI mortality in this country group, this reduction was similar to the all-country mean for Hib vaccine coverage (13·9% mean reduction [5·2–22·8]) and slower than the all-country mean for PCV coverage (9·0% mean reduction [6·4–24·6]). By contrast, these countries had mean increases in LRI mortality attributable to low antibiotic coverage (2·6% median increase [1·1–4·8]) and low birthweight and short gestation (2·6% median increase [0·1–5·7]). Many of these countries did not introduce the PCV, which was responsible for an increase in LRI mortality in ten countries (figure 4C), with Jordan (11·0% increase [6·2–15·9]) and Ukraine (6·7% increase [3·8–9·8]) having the largest increase in mortality rate due to low PCV coverage (appendix pp 93–111).

In 69 countries, both mortality and incidence were lower than the global median in 1990. We classified these countries as having low mortality and low incidence (lower left quadrant of figure 1; figure 4D). The mean decline in LRI mortality rate in these countries was 13·7 deaths (95% UI 1·7 to 39·9) per 100 000 children. Many of these countries reduced exposure to ambient air pollution (the greatest reduction was a 3·5% decline, 2·5 to 3·8, in the Czech Republic). The mean decline due to second-hand smoke exposure was 2·2% (−0·3 to 5·9) and was greatest in Greece (8·8% reduction, 7·3 to 10·9) and Iceland (7·5% reduction, 5·6 to 9·4; figure 4D). Countries in this group generally had decreases in LRI mortality attributable to greater vaccine coverage but these changes were similar to the decrease across all countries (appendix pp 93–111).

## Discussion

At the global level, LRI mortality declined substantially among children younger than 5 years between 1990 and 2017. Despite these declines, such progress has not occurred equally across countries, and LRIs remained the leading infectious cause of death among children younger than 5 years in 2017. In most locations, LRI incidence declined more slowly than mortality, suggesting that improvements in protecting against death are probably outpacing improvements in reducing the underlying risk of infection. Although SDI is strongly associated with under-5 LRI mortality, changes in SDI were not strongly associated with changes in mortality rates between 1990 and 2017. At the global level, 93·4% of under-5 LRI deaths were attributable to a risk factor. Among risk factors estimated in GBD 2017, we have conceptualised the change in under-5 LRI burden into two components, the changes in risks that predispose children to disease (prevention risk factors) and those that increase the risk of mortality given disease (protection risk factors). These categories are likely to partly overlap. For example, childhood growth failure, via immunological mechanisms, might make children both more likely to get sick and die from an LRI. Such a distinction is broad but provides a conceptual framework with which to assess the relationship between trends in risk factor exposure, mortality, incidence, and case fatality.

One of the main findings from our study is that the drivers of change in LRI mortality are not universal and might be highly specific to each location and that strategies to reduce health loss due to LRI must be tailored to a given setting. For example, the three countries with the greatest absolute decline in LRI mortality rate were Niger (1019·3 fewer deaths per 100 000 children), Laos (944·4 fewer deaths per 100 000 children) and Afghanistan (919·0 fewer deaths per 100 000 children). The Hib and pneumococcal vaccines were responsible for the largest percent change in Niger, whereas changes in childhood wasting explained more than 20% of the decline in Laos and Afghanistan, and lower exposure to household air pollution in Afghanistan decreased LRI mortality rate by 19·3%. By contrast, the two countries that observed the largest relative decline in LRI mortality between 1990 and 2017, China and Turkey, had relatively small changes attributable to changes in risk factors. Under-5 LRI mortality in China decreased by 91·2% between 1990 and 2017, whereas the mortality rate decreased by 26·2% because of changes in risk factor exposure. This suggests that other factors might explain the change in some countries.

Other studies have identified economic development, health-care reform to provide government-sponsored health care in rural areas,^11^, ^12^ improved detection of pneumonia following the severe acute respiratory syndrome and influenza virus subtype H1N1 pandemics, and programmes to reduce household air pollution from solid fuels as the main drivers of reduction in LRI mortality in China.^13^ The decline in LRI mortality in China might also be related to a so-called nutritional transition—similar to a transition previously observed in the USA and other high-income countries, but occurring at lower gross national product^14^—and might be explained by agricultural policies (including subsidisation and subsequent increase in soya bean oil and soya consumption),^15^ urbanisation,^16^ liberalisation of food production,^15^ and rapid economic development (exceeding 8% annually) in China during this time.^17^ This study did not include covariates that would specifically capture changes in infrastructure or food production but such changes might be reflected in the rapid increase in SDI observed in China between 1990 and 2017, and these trends deserve further investigation. Interventions and structural policies that aim to improve childhood nutrition can play a substantial role in protecting children from dying because of infectious diseases, including LRIs.^5^

China and Turkey have some of the fastest improvements in the Healthcare Access and Quality Index, a composite metric of amenable mortality.^18^ This improvement in preventing amenable causes of death suggests that the health-care systems in these countries have improved and are likely to have contributed to the rapid declines in LRI mortality. Access to care, adherence to treatment protocols, maternal education, appropriate technology, and adequate health-care staffing have all been identified as predictors of under-5 LRI survival^5^ that were not quantified in this study and might be components of a comprehensive LRI treatment strategy.

Our study found that low antibiotic use for LRIs was responsible for an increase in LRI mortality, particularly among countries with high mortality in 1990. An analysis of appropriate antibiotic therapy for childhood pneumonia determined that the availability and accessibility of WHO-recommended antibiotics is not equitable in some locations with substantial variation within countries.^19^ Antibiotic use for symptoms of pneumonia varied greatly among low-income and middle-income countries,^20^ and was estimated at just 18·8% globally, in 2017, in this study. A cohort study consisting of eight sites and a mixture of urban and rural locations found that 61% of children aged 0–2 years with acute LRI received antibiotics, including 69% in rural Tanzania and 86% in urban Bangladesh.^21^ Community case management of acute respiratory infections could reduce pneumonia-related mortality by 32%, according to a systematic review,^22^ and effective treatments such as oxygen therapy, rehydration, and antibiotics can dramatically reduce LRI mortality.

Appropriate care for LRIs might also depend on the availability and utilisation of health care. Primary health care in some low-income and middle-income countries has experienced challenges in funding, with general underuse of facilities and neglected human resources. Nigeria had the highest case fatality ratio globally in 2017 (5·7%, 95% UI 5·4–6·0) but had average coverage of antibiotics, suggesting other factors might be driving the high rate. Nigeria is a country of complex demographic, geographic, and cultural characteristics with substantial variation in health and health-care utilisation and performance indicators.^23^ It has the highest population of any country in Africa and by far the most deaths due to LRI among children younger than 5 years. Yet, a review of published literature on child health interventions found only 18 papers from Nigeria.^24^ Building evidence from high-burden countries should be a priority and is essential to a complete understanding of health loss due to LRI. One qualitative study^25^ revealed demand-side barriers to health-care utilisation in Nigeria, suggesting that costs, physical distance, cultural considerations, and knowledge of symptoms and warning signs might all play a role in delayed care-seeking and could explain behaviours in other locations. In one study^26^ of six countries in sub-Saharan Africa, only 30% of care givers recognised fast breathing or difficulty in breathing as a symptom of LRI. Among several countries in western sub-Saharan Africa, the proportion of care givers who reported seeking treatment for symptoms of pneumonia ranged from 27·4% in Chad to 73·2% in Sierra Leone and was 41·9% in Nigeria.^26^

Appropriate care and treatment to protect against LRI mortality are important but the two risk factors responsible for the largest reduction in LRI mortality at the global level and for the largest prevention of incident episodes of LRI are Hib vaccine coverage and household air pollution. Vaccines have been an important part of LRI prevention in many countries. In 2011, 2% of US$30·6 billion in international assistance for health spending was spent on pneumonia, and about 82% of that was through Gavi, the Vaccine Alliance, suggesting that nearly all pneumonia funding was for vaccines.^27^ A different analysis found that between 2000 and 2015, pneumonia received approximately $3 billion in funding for research and development, compared with more than $38 billion that went towards HIV/AIDS research during the same time.^28^ Of that $3 billion, about $839 million went to treatment, another $164 million to diagnostics, and $858 million went to vaccines.^28^

Globally, Hib and pneumococcal vaccines were responsible for an estimated 11·4% (Hib) and 6·3% (PCV) decrease in LRI mortality. Although nearly every country has introduced the Hib vaccine in their immunisation programmes, several high-burden countries such as Nigeria, Chad, and Somalia have not introduced the pneumococcal vaccine. Studies have shown substantial reductions in hospital admissions for pneumonia, invasive Hib, and pneumococcal pneumonia following vaccine introduction.^29^, ^30^, ^31^, ^32^ Although more countries might introduce PCV with Gavi support, there is uncertainty regarding the sustainability of these vaccines among countries that approach graduation from Gavi.^33^ The continued and expanded use of these vaccines have substantial impacts on under-5 LRI mortality and should be prioritised as part of routine immunisation programmes to prevent these deaths.

Two other risk factors that are associated with preventing incident cases of LRI had opposite trends in burden attribution. Between 1990 and 2017, LRI mortality decreased by 8·4% globally due to reductions in household air pollution but increased by 4·1% due to ambient air pollution. A pair of systematic reviews and meta-analyses found that exposure to household air pollution from solid fuel use increased the risk of pneumonia among children younger than 5 years.^34^, ^35^ A pair of randomised controlled trials found little statistical evidence of a reduction in pneumonia incidence with provision of chimneys^36^ or cleaner burning cookstoves,^37^ and several more studies investigating household air pollution and childhood pneumonia are in progress.^38^, ^39^ This suggests that the provision of chimneys or cleaner burning practices does not result in a sufficient reduction of air pollution to translate into a reduction in health effects. This finding is consistent with the analysis and recommendations by the WHO guidelines. Rapid urbanisation in many countries and a shift from conventional heating sources, such as coal and wood, to natural gas have probably contributed to reduced exposure to household air pollution.^40^ Reduced exposure to household air pollution was strongly associated with decreased incidence and mortality, particularly in high-burden countries in our study. We have previously described the interplay between development and ambient air pollution that is occurring in many countries that are rapidly urbanising and growing economically.^6^ Preventing LRI cases and deaths should be a part of any policy conversation regarding the burning of fossil fuels, and focusing on health effects of carbon-based energy might be a powerful rhetorical tool in developing policy to reduce air pollution.^41^, ^42^, ^43^

There are several limitations in this study. We have previously described some of the data gaps associated with LRI mortality modelling, specifically among countries with the highest estimated LRI burden.^6^ All our estimates are reported with uncertainty intervals and these intervals are larger in areas where we have fewer data. We attempt to improve our models by using patterns from locations where we do have data and by the relationship between LRI mortality and covariates. Many of the covariates used in the mortality modelling are also part of the risk factor attribution. Risk factors in GBD are typically a combination of population-level exposure and the relative risk of an outcome. The exposure to each risk factor is a covariate in our model. This analysis is strengthened by our ability to use risk factors to predict time trends in LRI mortality. Yet, the risk factor exposure used at multiple points in this analysis are also modelled values, dependent on data availability. Although we have quantified several interventions for LRI health-loss and antibiotic use, we do not explicitly account for health-care seeking behaviours, primary health-care availability, and specific recommended diagnostics or treatments for LRI. Understanding patterns in diagnostics and the use of pulse oximetry, chest x-rays, and oxygen therapy might reveal additional information about the drivers in LRI mortality trends. We do not have a modelled covariate for influenza vaccine coverage. Additionally, our model quantifies LRI deaths in HIV-negative people only because any deaths due to select infectious diseases, including LRI, among people with HIV, are classified as HIV deaths in the International Classification of Diseases; these deaths are captured in the overall HIV-related mortality estimation in GBD.^1^ To date, we have not separately quantified LRI deaths in HIV-infected people. As GBD is an iterative endeavour, we can examine this in our future work.

The global reduction in under-5 LRI mortality from 1990 to 2017 should be viewed as simultaneously a major public health achievement and as a call for renewed efforts to continue to reduce the burden of disease. Declines in LRI burden over time have been highly specific by location and are not fully explained by improvements in sociodemographic status alone. These results illustrate the contribution of improvements in air pollution, Hib and PCV vaccine coverage, child nutrition, and effective treatment to reducing disease and death due to LRI. Quantitative estimates of LRI incidence, mortality, and risk factor exposure can serve as a tool to sustain the progress made since 1990 and to design targeted strategies for countries where high LRI burden remains.

## Data sharing

In compliance with the Guidelines for Accurate and Transparent Health Estimates Reporting, data and code for GBD 2017 are publicly available.
