# Correction to *Lancet Infect Dis* 2020; 20: 60–79

**DOI:** 10.1016/S1473-3099(26)00376-2

**Published:** 2026-09

*GBD 2017 Lower Respiratory Infections Collaborators. Quantifying risks and interventions that have affected the burden of lower respiratory infections among children younger than 5 years: an analysis for the Global Burden of Disease Study 2017.* Lancet Infect Dis *2020;* **20:** 60–79—In this Article, the final sentence of the figure 1 legend should have been “The plots are therefore divided into ... compared with all other countries in 1990 and in 2017.” Additionally, in figure 4, the absolute change in mortality rate should have been per 100 000 children throughout. These corrections have been made as of July 6, 2026.

