# Supplementary material for: Quantifying risks and interventions that have affected the burden of lower respiratory infections among children younger than 5 years: an analysis for the Global Burden of Disease Study 2017
Source: Lancet Infect Dis. 2020 Jan;20(1):60–79. doi: 10.1016/S1473-3099(19)30410-4 (PMC7185492; doi:10.1016/S1473-3099(19)30410-4)
Supplement: Supplementary appendix [file mmc1.pdf]

THE LANCET Infectious Diseases

Supplementary webappendix

This webappendix formed part of the original submission and has been peer reviewed. We post it as supplied by the authors.

Supplement to: GBD 2017 Lower Respiratory Infections Collaborators. Quantifying risks and interventions that have affected the burden of lower respiratory infections among children younger than 5 years: an analysis for the Global Burden of Disease Study 2017. *Lancet Infect Dis* 2019; published online Oct 30. https://doi.org/10.1016/S1473-3099(19)30410-4.

Appendix for: *Quantifying risks and interventions to prevent and protect against lower respiratory infection health-loss among children under-5 years old: An analysis of the Global Burden of Disease study 2017*

GBD 2017 Lower respiratory infections Collaborators

This appendix is made up of several parts. The first is a detailed description of lower respiratory infection (LRI) modeling in the Global Burden of Disease study 2017 (GBD 2017). This description of LRI modeling is on pages 2-11. Pages 13-15 have a table detailing the risk factors assessed in this work including definitions for the risk and exposure. Following that, a description of input data and modeling strategy for each risk factor or intervention used in the analysis is provided (pages 16-58). Lastly, a Supplementary Results table provides estimates for the major results reported in the manuscript by country and GBD region.

Lower respiratory infection mortality methodology		2
Lower respiratory infection incidence methodology		8
Risk factor summary descriptive table		12
Risk factor methodology summaries		
	Child growth failure	15
	Household air pollution	21
	Ambient (outdoor) air pollution	23
	Secondhand smoking	32
	Summary table of particulate matter relative risks	35
	Unsafe hygiene	37
	Antibiotics for LRI	39
	Hib and Pneumococcal vaccines	42
	Zinc deficiency	46
	Low birth weight and short gestation	47
	Suboptimal breastfeeding	59
References		62
Supplementary country results tables		66
Maps of Socio-demographic Index		111

Lower respiratory infection modeling

Modeling LRI mortality methods

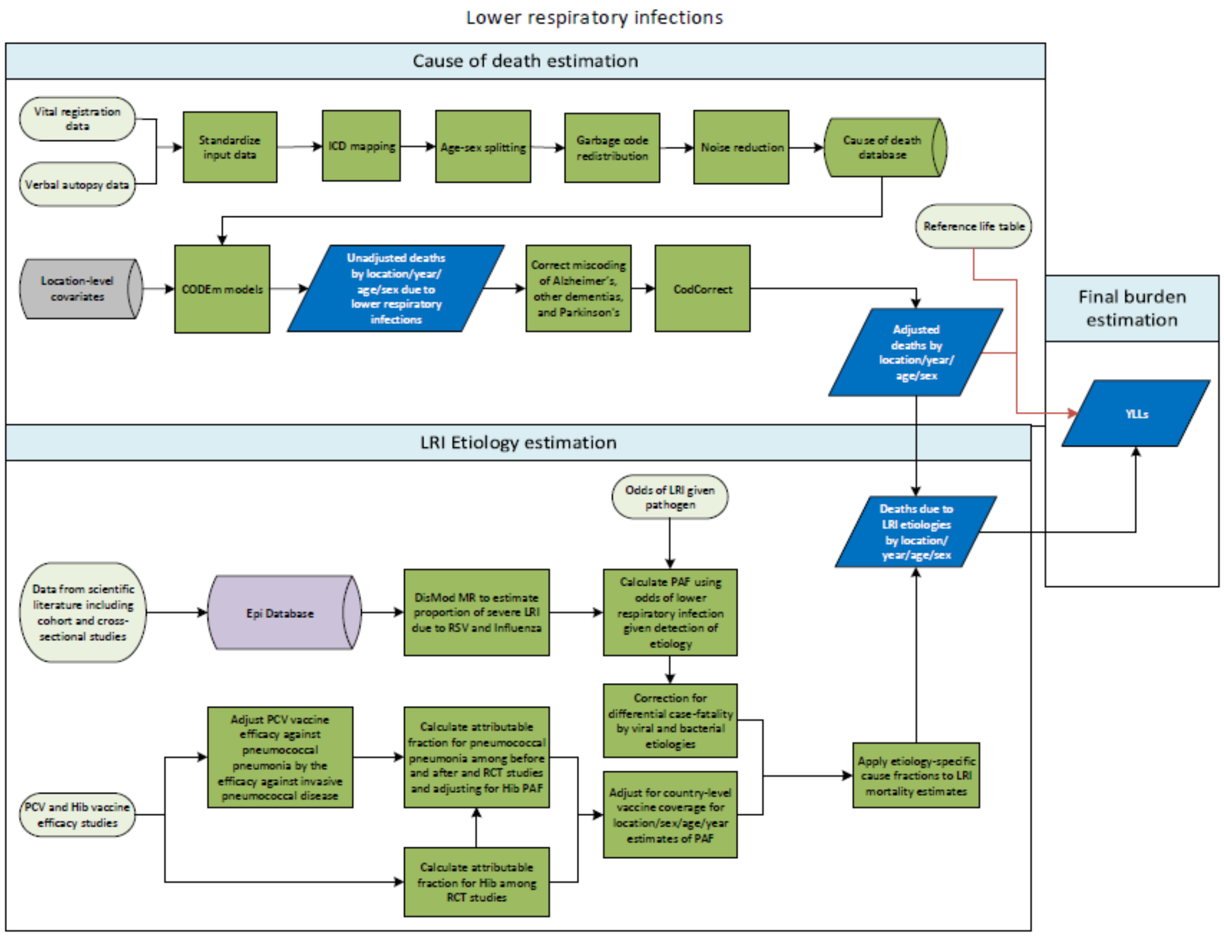

Input data

We used all available data from vital registration systems, surveillance systems and verbal autopsy (**Table 1 and Figure 1**). We checked for and excluded outliers from our data by country or region. We also excluded ICD9-coded mortality data in Sri Lanka (1982, 1987–1992), ICD9-coded neonatal mortality data in Guatemala (1980, 1981, 1984, 2000–2004), and medically-coded cause of death data (MCCD) and Civil Registration System data in many Indian states (1986–2013).

There are three main aspects of cause of death data preparation. The first is age-sex splitting. This is necessary because many data are only available in age ranges or for both sexes and all COD data must be sex-age specific. This occurs after using all data to produce a global age/sex pattern which is then used to proportionally split those data. The next step is garbage code redistribution. This takes deaths that are coded to causes of death that are not part of the GBD hierarchy and assigns them to an appropriate

cause of death. A relevant example would be deaths coded to sepsis. This is a non-specific cause of death. A series of regressions determines the proportion of these garbage codes that should be reassigned to each cause. The last step is noise reduction which is essentially a way to move data points up or down in magnitude based on regional trends. The amount of change is dependent on the data variance. Those points with greater variance are subject to be moved more than those with lesser variance. Data points from verbal autopsy studies and data points that are not nationally representative have variance that is inflated to account for these issues.

Table 1. Cause-specific mortality input data.

**Input data**	**GBD 2017**
**Total data sources**	**19,827 geography-years**
Vital registration data	17,374 geography-years
Sample registration data	740 geography-years
Verbal autopsy data	1,153 geography-years
Surveillance data	560 geography-years

Figure 1. Number of geography-years of mortality data used in LRI mortality modelling.

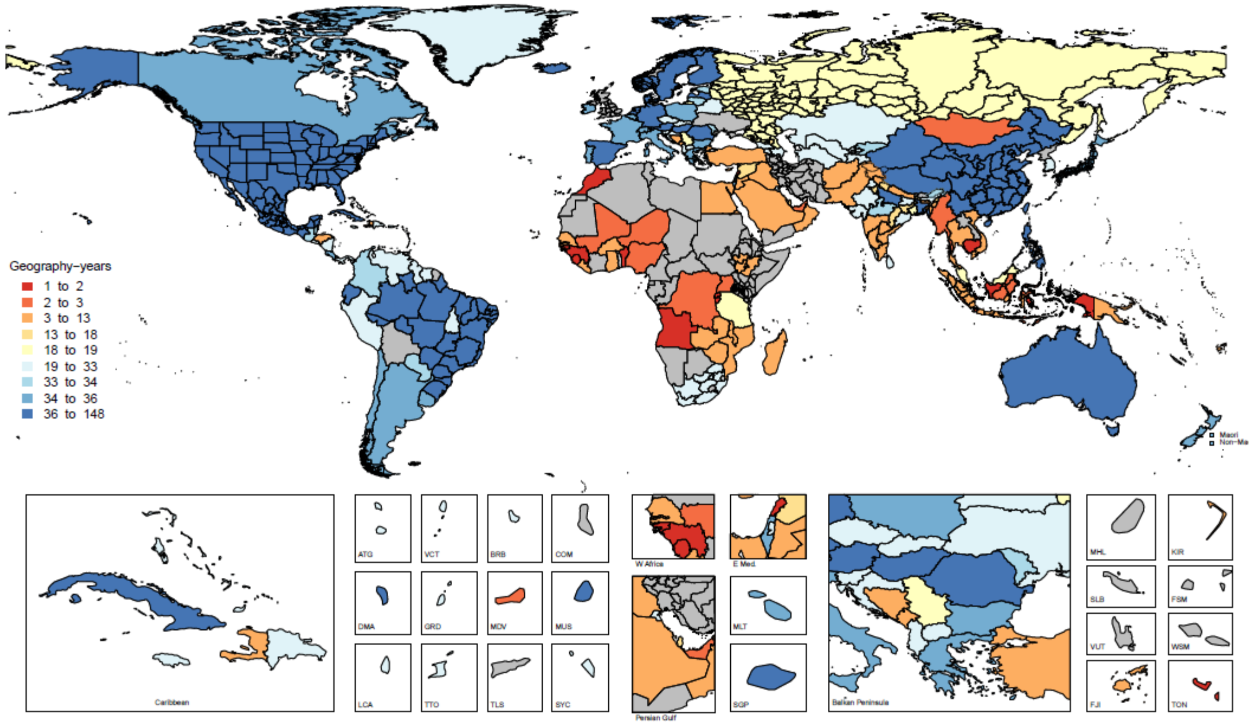

Modeling strategy

Lower respiratory infection (LRI) mortality was estimated in the Cause of Death Ensemble modeling platform (CODEm). We estimated LRI mortality separately for males and females and for children under 5 years and older than 5 years. We used country-level covariates to inform our CODEm models (**Table 2**). We evaluated our LRI cause of death models using in and out of sample predictive performance.

CODEm is a Bayesian statistical model and uses spatial priors from a hierarchical structure to inform the mortality models. CODEm produces a large suite of models based on either cause fraction or mortality rate, uses mixed-effects linear and space-time Gaussian process regression models, and a covariate selection process. Each sub-model is evaluated using out-of-sample predictive validity. Thirty percent of the data are excluded from the initial model fits and 15% are used to evaluate component models and 15% used to build the ensembles. The sub-models are ranked using 15% of the data based on their out-of-sample predictive validity. The proportion weighting of the ensemble sub-models is evaluated using the remaining 15% of the hold-out data. This weighting scheme evaluates ensemble models that are built with ranked sub-models contributing proportionally more or fewer draws to the final ensemble. The final ensemble model is evaluated against other ensemble models using the same fit statistics (in-sample, out-of-sample root mean squared error and data coverage). Detailed

information on this process can be found in Foreman et al 2012[1] and in the GBD 2017 Mortality and Causes of Death manuscript.[2]

LRI mortality is estimated for 23 age groups, 774 locations, both sexes, and every year from 1980-2017. We estimated LRI mortality separately for males and females and for children under 5 years and older than 5 years due to expected underlying differences in the risk of mortality between these age groups. Data-rich and data-poor geographic locations were modelled separately and these models were then hybridised for a global model. This was to maintain proper uncertainty in the models where trusted data on causes of death exist. For a detailed description of the input data coverage, completeness, and reliability of the cause of death data in GBD 2017, please refer to the scoring system introduced in the GBD 2016 Mortality Collaborators manuscript.[3]

Like all models of mortality in GBD, LRI mortality models are single-cause, requiring in effect that the sum of all mortality models must be equal to the all-cause mortality envelope. We correct LRI mortality, and other causes of mortality, by re-scaling them according to the uncertainty around the cause-specific mortality rate. This process is called CoDCorrect and is essential to ensure internal consistency among causes of death.

In CODEm, the "level" of a covariate should reflect its position in a causal pathway where 1 is most proximally related to LRI mortality (causal) and 3 is distally related or a proxy for LRI mortality. Details on CODEm covariate and sub-model selection can be found elsewhere[4] but the core idea is that submodels are built first using level 1 covariates and by adding covariates until the coefficient is either not significant or changes *direction.* The "direction" of a covariate is the sign of the coefficient. For covariates where the direction is positive, the covariate should be associated with LRI mortality such that greater exposure to the covariate is related to greater LRI mortality. The inverse is true for covariates where the direction is negative.

Table 2. The covariates used in LRI mortality modeling. The *Level* represents the strength of the association between the covariate and LRI mortality from 1 (proximally

related) to 3 (distally related). The *Direction* indicates the positive or negative association between the covariate and LRI mortality.

Level	Covariate	Direction
1	Childhood stunting SEV	+
	Childhood underweight SEV	+
	Childhood wasted SEV	+
	Indoor air pollution	+
	Short gestation SEV	+
	Low weight gestation	+
	LRI summary exposure variable	+
	Second-hand smoking prevalence	+
	Antibiotics for LRI	-
	Hib vaccine coverage	-
	Pneumococcal conjugate vaccine coverage	-
2	Discontinued breastfeeding SEV	+
	Vitamin A deficiency	+
	Zinc deficiency	+
	DTP3 vaccine coverage	-
	Healthcare access and quality index	-
3	Outdoor air pollution ($PM_{2.5}$)	+
	Population density > 1000/km^2	+
	Sanitation SEV	+
	Handwashing	-
	LDI per capita	-
	Maternal education	-
	Socio-demographic Index	-

Comparison to other global health estimates

The number of deaths due to lower respiratory infections among children under-5 in the year 2016 is shown for the WHO-MCEE group as well as for GBD 2017 and GBD 2016.

Location	WHO-MCEE	GBD 2017	GBD 2016
**Global**	**878,829**	**860,373**	**701,000**
Indonesia	20,009	11,885	14,300
India	157,999	206,277	167,500
Pakistan	62,680	43,263	30,600
Democratic Republic of the Congo	48,961	37,173	30,800
Ethiopia	30,667	26,242	21,800
Nigeria	140,256	157,922	61,600

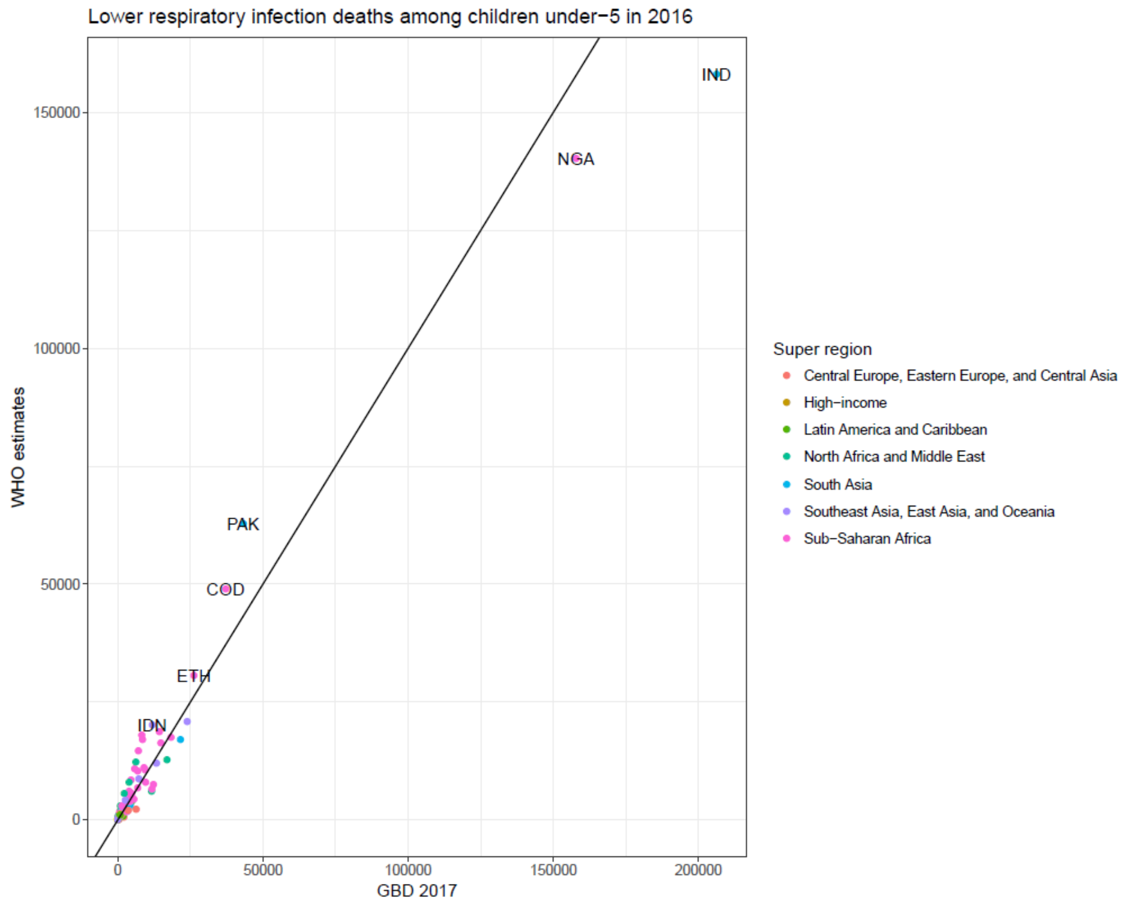

Our estimates of the number of LRI episodes among children under-5 in 2010 (74,130,000, 95% UI 60,610,000-89,700,000) are about half of the estimates produced by the Child Health Epidemiology Research Group (CHERG) (120,400,000, 95% UI 60,800,000-277,000,000) but with overlap in the 95% uncertainty intervals.[20] The

estimates produced by the MCEE are informed by the incidence of pneumonia in 35 cohort studies which informed an envelope of pneumonia incidence that was related to the prevalence of five risk factors for pneumonia to estimate country-level incidence.[5,6] While there is overlap in the risk factors used in both studies, GBD 2016 utilised over 30,000 data points from more than 700 sources to produce internally consistent estimates of LRI incidence, prevalence, and mortality.

Further information

To find a full list of data sources used in the LRI modeling, please visit the Global Health Data Exchange (GHDx: http://ghdx.healthdata.org/gbd-2017/data-input-sources)

To view intermediate models for LRI fatal models, please visit our Visualizations Hub (http://vizhub.healthdata.org/cod/)

To view final results for LRI fatal models, please visit our Visualizations Hub (https://vizhub.healthdata.org/gbd-compare/)

Lower respiratory infection modeling

Modeling LRI incidence methods

Lower respiratory infections

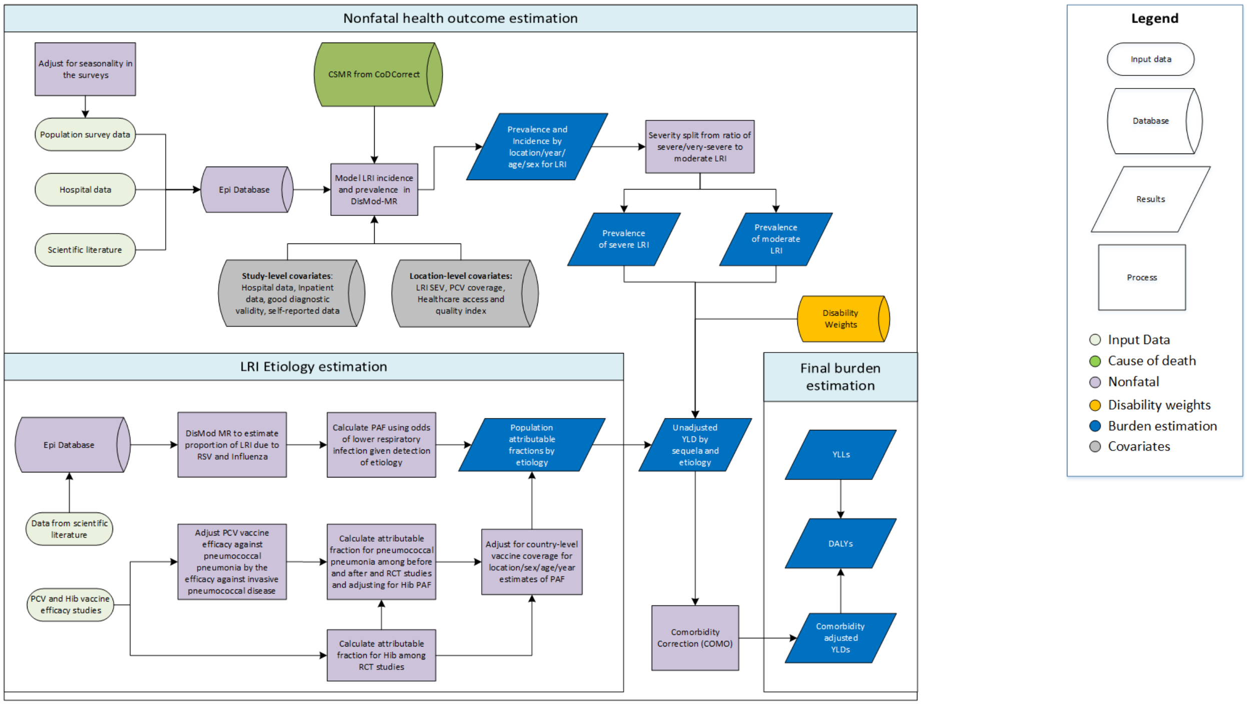

Case definition

We used clinician-diagnosed pneumonia or bronchiolitis as our case definition for lower respiratory infections (LRI). We included ICD9 codes 073.0-073.6, 079.82, 466-469, 480-489, 513.0, and 770.0 and ICD10 codes A48.1, J09-J22, J85.1, P23-P23.9, and U04.

Description of model

Non-fatal outcomes for LRI are modeled using DisMod-MR 2.1. DisMod is is a Bayesian statistical model developed for several main purposes. The first, and most important, is to enforce consistency between incidence, prevalence, recovery, and mortality. It does this using a series of ordinary differential equations to solve transition rates. The second is to enforce consistency in data types. The third thing DisMod does is age-integrate. An important limitation is that there is no cohort effect in DisMod so age-time patterns are static. The last thing DisMod does is to produce estimates of incidence and prevalence by geography-year-age-sex.

We performed a systematic review of the duration of symptoms of LRI. We sought consistency with our case definition of LRI and defined our duration as the time between the onset of symptoms to the resolution of increased work of breathing. Although crucial, there were very limited data on spatial, temporal, or age-specific duration which may vary based on severity, etiology, and treatment. We identified 485 titles from PubMed and extracted 6 studies which were used in a meta-analysis (mean duration 7.79 days, 6.2-9.64 days). This duration is used for converting period to point prevalence and for converting between incidence and prevalence.

Covariates that were used in the model are listed in the table below.

Study covariate	Type	Parameter	Exponentiated beta
Self-reported	Study-level	Prevalence	3.58 (3.36-3.73)
Hospital data	Study-level	Prevalence	1.65 (1.58-1.72)
Marketscan	Study-level	Prevalence	2.39 (2.29-2.45)
LRI SEV	Country-level	Prevalence	1.00 (1.00-1.01)
Socio-demographic Index	Country-level	Prevalence	0.47 (0.42-0.52)
Healthcare access and quality index	Country-level	Excess mortality	0.99 (0.99-0.99)

Data Inputs for LRI morbidity modeling

A summary of the input data by location and type is shown below.

Type of data	Data points (#)
**Overall data points**	**59,168**
Facility - inpatient	42,301
Facility - other/unknown	6540
Survey - cross-sectional	5791
Facility - outpatient	1505
Survey - other/unknown	1749
Survey - longitudinal	470
Survey - cohort	360
Surveillance - facility	190
Surveillance - other/unknown	89

The number of geography-years of data used in the LRI non-fatal modeling by GBD geography is shown.

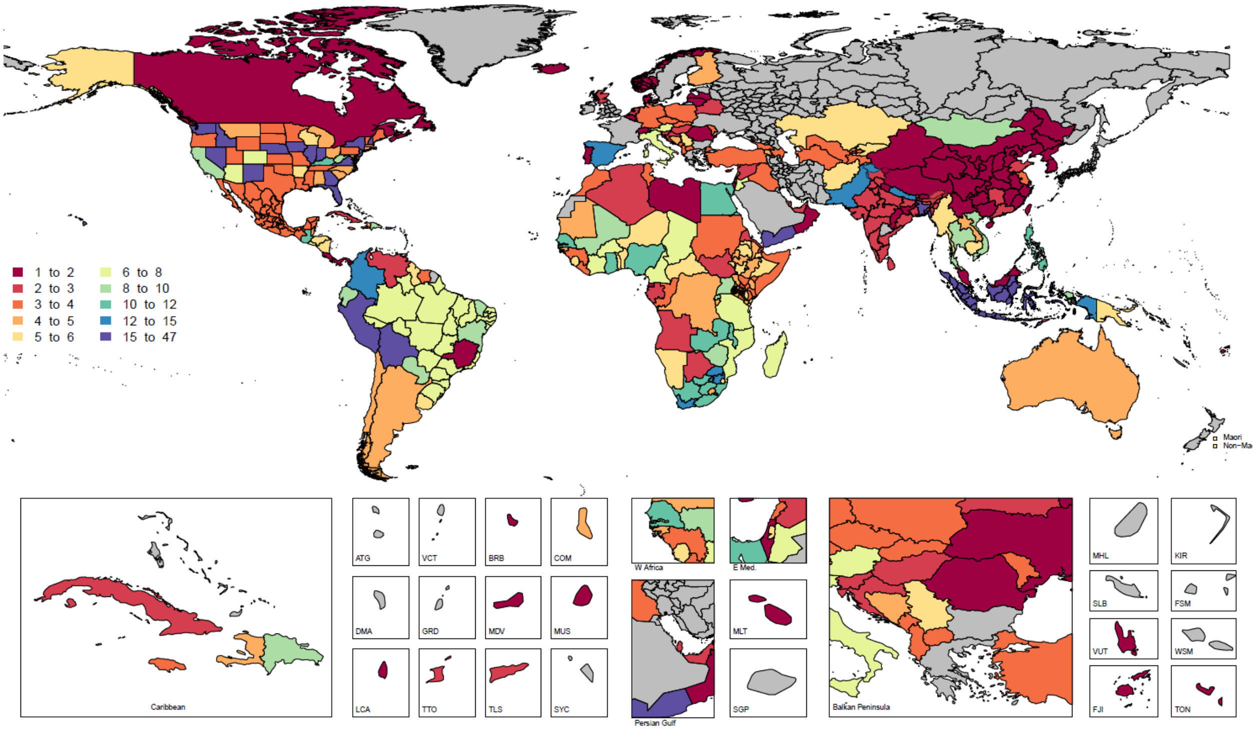

Survey data sources

We used self-reported prevalence of LRI symptoms from population-representative surveys, such as the Demographic and Health Survey and the Multiple Indicator Cluster Survey. When possible, we extracted survey data by 1-year age group and by sex. We converted these data from two-week period prevalence to point prevalence. The equation for this adjustment is

$$1)\quad Point\ Prevalence = \frac{Period\ Prevalence * Duration}{(Recall\ Period + Duration - 1)}$$

We accepted four survey definitions for the prevalence of symptoms of LRI: 1) Cough with difficulty breathing with the symptoms in the chest with a fever was our gold standard but we also accepted 2) Cough with difficulty breathing with the symptoms in the chest *without* fever, 3) Cough with difficulty breathing with fever, and 4) Cough with difficulty breathing *without* fever. To make these definitions comparable, we identified the surveys that met the best case definition (definition 1). Within these surveys, we calculated the ratio of the prevalence of the best case definition to the prevalence of the alternate definitions. This ratio was regressed using age in years (factor), year of the survey, and GBD region (random intercept). The predicted values were used to adjust the prevalence for all the surveys that reported alternate case definitions.

Survey data were adjusted for seasonality. An inclusion criterion for scientific literature is a study duration longer than 1 year to avoid bias in the seasonal timing of LRI. Surveys are frequently conducted over several months. To account for seasonal variation in LRI symptom prevalence, we fit a generalized additive model with a forced periodicity for each GBD region. The model is mixed-effects with random effects on each country. The model accounts for the year of the survey and the case definition used. The percent difference between the monthly model fit LRI prevalence and the mean fitted LRI prevalence is a scalar to adjust survey data by month and geography.

Hospital and claims data

In addition to survey data, hospital inpatient, outpatient data, and US claims data were included in the LRI modeling. To make the data more consistent in the modeling process, we converted all incidence data to prevalence.

We found the ratio of the prevalence of LRI in hospitalization records to the prevalence of LRI in our case definition (clinician-diagnosed pneumonia or bronchiolitis) for locations that contained data on both these prevalence values. We then regressed this ratio using age in years (factor) and GBD region (random intercept) to predict the adjustment factor for hospitalization data to make them compatible with the reference case definition for our modeling.

Further information

To find a full list of data sources used in the LRI modeling, please visit the Global Health Data Exchange (GHDx: http://ghdx.healthdata.org/gbd-2017/data-input-sources)

To view intermediate models for LRI non-fatal models, please visit our Visualizations Hub (http://vizhub.healthdata.org/epi/)

To view final results for LRI non-fatal models, please visit our Visualizations Hub (https://vizhub.healthdata.org/gbd-compare/)

Definitions for risk factors in lower respiratory infection modeling

Type	Risk Factor	Exposure definition	Modeled values	Interpretation
Prevent	Ambient air pollution	A continuous measure of exposure to air particles of less than 2.5 micrometers in diameter in a cubic meter of air, reported in ug/m3	The continuous, population-weighted annual average concentration of particles less than 2.5 micrometers in diameter per cubic meter of air. This value is modeled on a fine spatial resolution and aggregated to GBD locations.	The annual average concentration of ambient particulate matter pollution in a population.
Prevent	No Handwashing	The proportion of the population that does not have access to a handwashing station with available soap and water.	The prevalence of the availability of a handwashing station with soap and water.	The provision of a handwashing station with soap reduces the exposure to infectious agents.
Prevent	Household air pollution	The proportion of households using solid cooking fuels including coal, wood, charcoal, dung, and agricultural residues.	The prevalence of households using solid cooking fuels.	The prevalence of a population that is exposed to household air pollution due to cooking fuel sources.
Prevent	Low Haemophilus influenzae type B vaccine coverage	The proportion of children that are not vaccinated against Hib	The prevalence of children receiving a full course of Hib vaccine.	Not receiving the Hib vaccine puts children younger than 5 at an increased risk of dying from Hib LRI
Prevent	Low Pneumococcal pneumonia vaccine coverage	The proportion of children that are not vaccinated against pneumococcal pneumonia	The prevalence of children receiving a full course of pneumococcal conjugate vaccine.	Not receiving the pneumococcal vaccine puts children younger than 5 at an increased risk of dying from pneumococcal pneumonia LRI. A full course is defined as three doses of either PCV 10 or PCV 13.
Prevent	Second-hand smoking	Current exposure to secondhand tobacco smoke at home	The prevalence of living in a household with a current daily smoker.	The prevalence of exposure to tobacco smoke. Assumed exposure in households with at least one active daily smoker.
Prevent	Zinc deficiency	Consumption of less than 2.5 milligrams of dietary zinc per day	The prevalence of children who do not receive sufficient dietary zinc.	The low consumption of zinc puts children at elevated risks for mortality due to LRI.
Protect	Low antibiotic coverage for LRI	The proportion of children that do not receive antibiotics for episodes of LRI	Prevalence of antibiotic use among children younger than 5 with LRIs	The proportion of children with an LRI episode that did not receive antibiotics as treatment for the episode.
Protect	Childhood stunting	Proportion of children younger than 5 years that are less than	Prevalence of mild, moderate, and severe stunting.	Children who are short for their age, based on international growth standards, have a

		the WHO 2006 growth standard for height-for-age based on z-scores from that standard. The prevalence of mild (<-1 z score), moderate (-1 to -2 z scores), and severe (>-3 zscores) were estimated for each population.		greater risk of dying from LRI than children who are not. Relative risks for LRI mortality by mild, moderate, and severe stunting are used in this analysis as are modeled prevalence estimates for each of the stunting categories.
Protect	Childhood underweight	Proportion of children younger than 5 years that are less than the WHO 2006 growth standard for weight-for-age based on z-scores from that standard. The prevalence of mild (<-1 z score), moderate (-1 to -2 z scores), and severe (>-3 zscores) were estimated for each population.	Prevalence of mild, moderate, and severe underweight.	Children who are low body weight for their age, based on international growth standards, have a greater risk of dying from LRI than children who are not. Relative risks for LRI mortality by mild, moderate, and severe underweight are used in this analysis as are modeled prevalence estimates for each of the underweight categories.
Protect	Childhood wasting	Proportion of children younger than 5 years that are less than the WHO 2006 growth standard for weight-for-height based on z-scores from that standard. The prevalence of mild (<-1 z score), moderate (-1 to -2 z scores), and severe (>-3 zscores) were estimated for each population.	Prevalence of mild, moderate, and severe wasting.	Children who are low weight for their height, based on international growth standards, have a greater risk of dying from LRI than children who are not. Relative risks for LRI mortality by mild, moderate, and severe wasting are used in this analysis as are modeled prevalence estimates for each of the wasting categories.
Protect	Low birth weight and short gestation	A joint estimation of the prevalence of low birth weight (less than 2500 grams) and of short gestation (shorter than 37 weeks gestation).	Jointly estimated prevalence of low weight and short gestational period, measured in a matrix of 500-gram birth weight and 2-week gestational periods.	The prevalence of low birth weight and short gestation period are modeled jointly. The prevalence for each category of birth weight, in 500 gram bins, and short gestation, in 2 week bins, represents the proportion of children in a population that were born prematurely.
Protect	Suboptimal Breastfeeding	Suboptimal breastfeeding is either non-exclusive breastfeeding or discontinued breastfeeding. Non-exclusive breastfeeding is the proportion of children younger than 6 months that are not exclusively	Prevalence of predominant, partial, and no breastfeeding among children younger than 6 months, prevalence of children 6-23 months who receive no breastmilk.	Suboptimal breastfeeding is either non-exclusive breastfeeding for infants under 6 months or discontinued breastfeeding for children 6-23 months.

	breastfed (predominant, partial, and none). Discontinued breastfeeding is the proportion of children 6 to 23 months who receive no breast milk.	

Child Growth Failure (stunting, wasting, underweight)

Flowchart

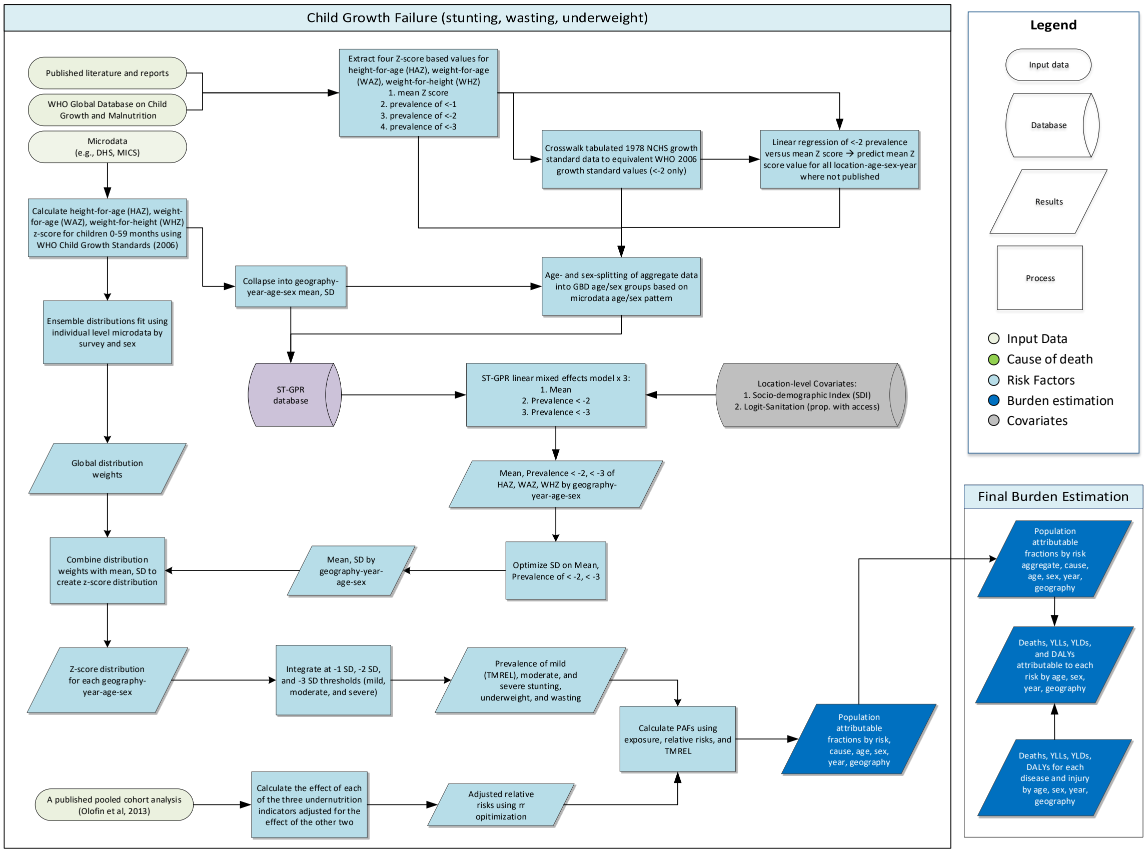

Input data & methodological summary

Exposure

Case definition

Child growth failure is estimated using three indicators, stunting, wasting, and underweight, all of which all of which are based on categorical definitions using the WHO 2006 growth standards for children 0-59 months. Definitions are based on Z scores from the growth standards, which were derived from an international reference population. Mild, moderate, and severe categorical prevalences were estimated for each of the three indicators.

Input data

There are three main inputs for the GBD child growth failure models: microdata from population surveys and tabulated data from reports, published literature, and the WHO Global Database on Child Growth and Malnutrition.[7] The primary data additions in GBD 2017 for child growth failure were from population surveys that include anthropometry. Population surveys include a variety of multi-country and country-specific survey series such as Multiple Indicator Cluster Surveys (MICS), Demographic

and Health Surveys (DHS), Living Standards Measurement Surveys (LSMS), and the China Health and Nutrition Survey (CHNS), as well as other one time country specific surveys such as the Indonesia Family Life Survey and the Brazil National Demographic and Health Survey of Children and Women. These microdata contain information about each individual child's age (from which age in weeks and age in months are calculated), as well as height and/or weight. From that information, a height-for-age z-score (HAZ), weight-for-age z-score (WAZ), and weight-for-height z-score (WHZ) are calculated using the WHO 2006 Child Growth Standards and the LMS method.[8]

All available data from the WHO Global Database on Child Growth and Malnutrition was extracted for GBD 2016 – much of which is from published studies. Exclusions included examination date prior to 1985, non-population representative studies, and those based on self-report. A systematic literature review was last completed in GBD 2010. We looked for four metrics from all sources with tabulated data: mean Z score, prevalence <-1 Z score (mild), prevalence <-2 Z score (moderate), and prevalence <-3 Z score (severe). All data for each metric was extracted for each of stunting (height-for-age Z score; HAZ), wasting (weight-for-height Z score; WHZ), and underweight (weight-for-age Z score; WAZ).

To maximise internal-consistency and comprehensiveness of the modelling dataset, we performed three data transformations. First, any data that were reported using the National Center for Health Statistics (NCHS) 1978 growth standards were crosswalked to corresponding values on the WHO 2006 Growth Standards curves based on a study that evaluated growth standard concordance.[9] Crosswalks from 1978 to 2006 growth standards were performed only on <-2 (i.e. moderate) prevalence data as that is where the concordance was most consistent. Second, for any study that lacked a measure of mean Z score for any of stunting, wasting, or underweight, we predicted a mean value for that study based on an ordinary-least squares regression of mean Z score versus <-2 prevalence for that metric from all sources where both were available. Third, any data that was presented as both sexes combined or for 0-59 months combined, we used the age and sex pattern from all data sources that included that detail to split into corresponding and age- and sex-specific data. All data was uploaded to a database and all inputs are catalogued in the Global Health Data Exchange (http://ghdx.healthdata.org). A representative dataset coverage map for moderate stunting is shown below.

Figure 1: Number of data points in moderate stunting (<-2 HAZ) in males, 1990 to 2017

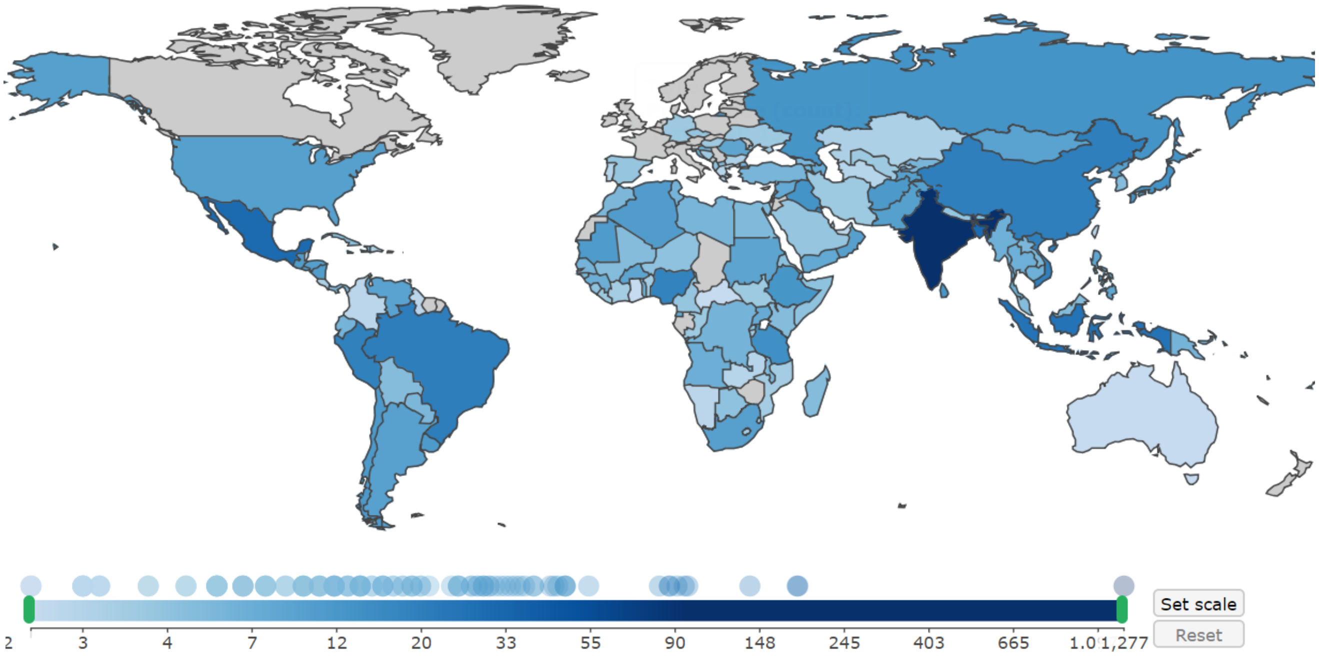

Modelling strategy

Exposure estimation

The following three-step modelling process was applied to each of stunting, wasting, and underweight.

First, all microdata was fit using an ensemble modelling process, a modelling framework developed for GBD 2016 that is described elsewhere in this appendix. A series of 12 individual distributions (normal, log normal, log logistic, exponential, gamma, mirror gamma, inverse gamma, gumbel, mirror gumbel, Weibull, inverse Weibull, and beta) were fit to the entire set of microdata (approximately 2.5 million individual z-scores) at the individual survey level. A weighting algorithm combined each distribution to find the optimal combination of these distributions for each survey, minimising the absolute prediction error across the entire distribution. Ensemble weights for each survey were then averaged across all surveys to produce a single set of global weights of the ensemble distributions. Weights were different for each sex, but invariant across geography, time, and age group. All component distributions that were used to derive weights were parameterised using "method of moments," meaning that each corresponding probability density function (PDF) could be described as a function of the mean and variance of the quantity of interest.

Second, models were developed for mean Z scores and prevalence of moderate and severe growth failure. Individual level microdata were collapsed to calculate three metrics: mean z-score, moderate prevalence, and severe prevalence. These data were combined with that derived from literature, GHDx review, and the WHO Global

Database on Child Growth and Malnutrition. Each of the three metrics was then modelled using spatiotemporal Gaussian process regression (ST-GPR), a common modelling framework used across GBD, generating estimates for each age-group, sex, year, and location. Location-level covariates used in all models included Socio-demographic Index (SDI) and logit-transformed proportion of households with improved sanitation.

Third, we combined estimates of mean, prevalence (moderate and severe) with ensemble weights in an optimisation framework in order to derive the variance that would best correspond to the predicted mean and prevalence. This variance was then paired with the mean and, using the method of moments equation for each of the component distributions of the ensemble, PDF of the distribution of Z-scores were calculated for each location, year, age-group, and sex. PDFs were integrated to determine the prevalence between -1 and -2 Z scores (mild), between -2 and -3 Z scores (moderate), and below -3 Z scores (severe). These were categorical exposures used for subsequent attributable risk analysis.

Ad-hoc data exclusions were limited. In some cases, we identified surveys with evidence of data entry issues (e.g. weights entered in a mixture of pounds and kilograms) that could not be corrected and these data were outliered. We initially ran all models with the complete dataset. Data plausibility inspection began with examination of time trends in stunting. If a given datum was judged to have led to a change in the prevalence of moderate stunting in 1-4 year olds of 50% or greater in 5 years or fewer, and was inconsistent with data prior to and after that year (a change considered implausible), we outliered the offending datum and reran the model. We then further visually-inspected the results of moderate stunting, wasting, and underweight in parallel to look for location-age-sex-years where the results were not internally-consistent (e.g. stunting and wasting decreasing, underweight rapidly increasing). This inspection revealed very few inconsistent data.

Improvements from GBD 2015 to GBD 2016/ 2017

In GBD 2017, the primary changes from GBD 2016 were the 1) addition of a significant volume of new survey data, 2) crosswalking instead of down-weighting data based on NCHS 1978 growth standard, 3) utilisation of updated versions of location-level covariates, and 4) utilisation of an updated version of the ST-GPR modelling framework that empirically derives many of the modelling parameters.

There are several important differences from the GBD 2015 analysis. First, our systematic data searching efforts led to an approximately 30% increase in the number of data sources since GBD 2015, including a significant increase in data sources for Oceania, Latin America, and South Asia. Most notable was the increase in data for India through our collaboration with the India Council for Medical Research (ICMR) and

Public Health Foundation of India (PHFI). Second, while GBD 2015 also used ST-GPR to model growth failure, models were completed for a single 0-5 age group, followed by application of a pooled uniform age-sex split which resulted in the implicit assumption that the age pattern of growth failure is invariant over time and geography. GBD 2016 estimates, owing to smaller sample sizes in younger age groups, do have wider uncertainty in those age groups. Third, GBD 2015, like all analyses of growth failure before it, assumed that high-income countries had zero prevalence of child growth failure. We suspended this assumption in GBD 2016 as it is not accurate and instead made explicit estimates of growth failure in all locations. Fourth, GBD 2015 did not use an ensemble approach or estimate the entire distribution of Z scores. Fifth, we changed the name of this risk factor category changed from childhood undernutrition to child growth failure to more explicitly identify the specific aspects of childhood undernutrition that are covered by the three component indicators.

Theoretical minimum-risk exposure level

Theoretical minimum risk exposure level (TMREL) for underweight, stunting, and wasting was assigned to be greater than or equal to -1 SD of the WHO 2006 standard weight-for-age, height-for-age, and weight-for-height curves respectively. This has not changed since GBD 2010.

Relative risks

The final list of outcomes paired with child growth failure risks included lower respiratory infections (LRI), diarrhea, measles, and protein energy malnutrition (PEM) as shown in Table 1. These were derived from a pooled cohort analysis by Olofin and colleagues.[5]

There is a high degree of correlation between stunting, wasting, and underweight. Failing to account for their covariance and assuming independence would overestimate the total burden significantly. This is the main reason that GBD 2010 only included childhood underweight. In GBD 2013, a method was developed to adjust observed RRs of Olofin and colleagues by simulating the joint distribution of the three indicators using the distribution of each indicator and covariance between indicators in the countries included in the meta-analysis (extracted from Demographic and Health Survey (DHS) micro-data).[10] Based on the analysis done by McDonald and colleagues, we assumed there is an interaction between the three indicators, and extracted the interaction terms from the corresponding analysis. We calculated the adjusted RRs by minimising the error between observed crude RRs (from meta-analysis) and expected crude RRs derived from adjusted RRs.

Of historical note, URI and otitis media were included as outcomes in the GBD 2013 risk analysis, based on the “analogy” causal criterion, assuming there is similar pathway as LRI outcome. However, closer review for GBD 2015 did not find sufficient evidence to support their inclusion and they were excluded, a decision that was carried forward into GBD 2016. We also attributed 100% of PEM to childhood wasting and underweight

but not stunting. To build on the existing literature base for GBD on risk-outcome pairs, a literature search was conducted for GBD 2017 searching for case-control studies published after January 1st, 1985; this search did not return any sources that were appropriate for this work.

Table 1: Adjusted RRs for each risk-outcome pair for child growth failure

Outcome	Stunting	Wasting	Underweight
Diarrhea	<-1: 1.111 (1.023-1.273)<br><-2: 1.222 (1.067-1.5)<br><-3: 1.851 (1.28-2.699)	<-1: 6.601 (2.158-11.243)<br><-2: 23.261 (9.02-35.845)<br><-3: 105.759 (42.198-157.813)	<-1: 1.088 (1.046-1.134)<br><-2: 1.23 (1.163-1.314)<br><-3: 2.332 (2.076-2.802)
Lower respiratory infections (LRI)	<-1: 1.125 (0.998-1.655)<br><-2: 1.318 (1.014-2.165)<br><-3: 2.355 (1.15-5.114)	<-1: 5.941 (1.972-11.992)<br><-2: 20.455 (70.84-37.929)<br><-3: 47.67 (15.923-94.874)	<-1: 1.145 (1.044-1.364)<br><-2: 1.365 (1.215-1.755)<br><-3: 2.593 (1.908-4.39)
Measles	<-1: 1.103 (0.861-1.719)<br><-2: 1.54 (1.029-3.222)<br><-3: 2.487 (1.129-6.528)	<-1: 1.833 (0.569-8.965)<br><-2: 8.477 (1.33-42.777)<br><-3: 37.936 (5.088-199.126)	<-1: 0.995 (0.5-1.726)<br><-2: 2.458 (1.26-5.118)<br><-3: 5.668 (1.767-12.414)
Protein-energy malnutrition	0% PAF	100% PAF	100% PAF

Household Air Pollution

Flowchart

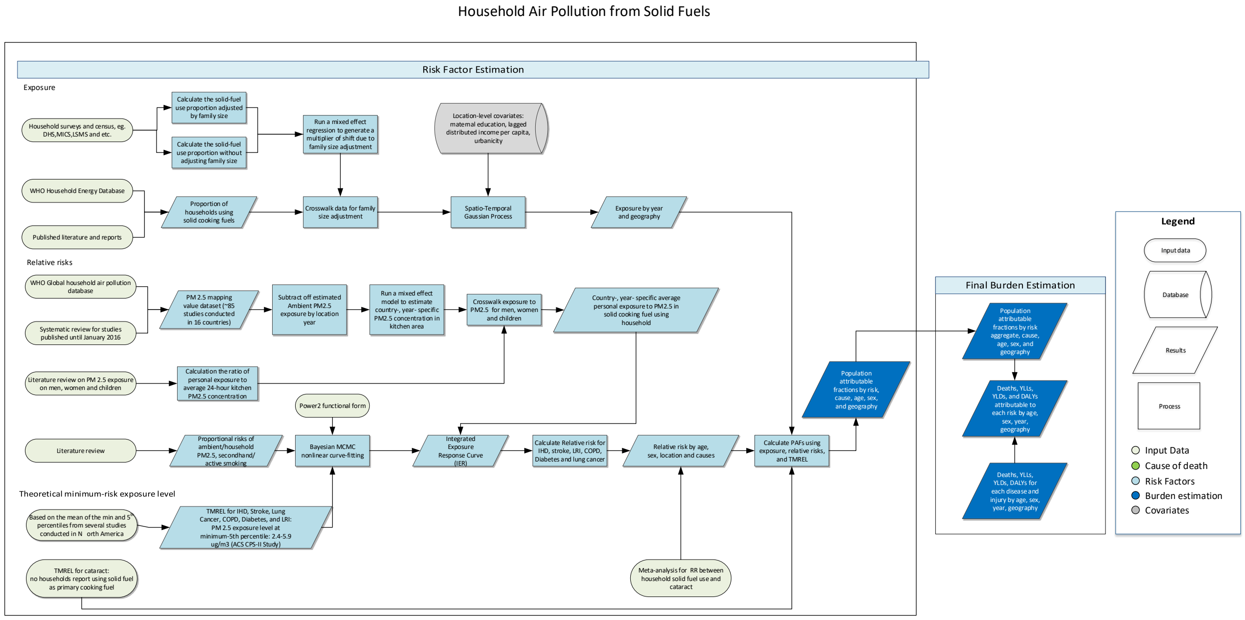

Input Data & Methodological Summary

Exposure

Case definition

Exposure to household air pollution from solid fuels (HAP) is defined as the proportion of households using solid cooking fuels. The definition of solid fuel in our analysis includes coal, wood, charcoal, dung, and agricultural residues.

Input data

Data were extracted from the standard multi-country survey series such as Demographic and Health Surveys (DHS), Living Standards Measurement Surveys (LSMS), Multiple Indicator Cluster Surveys (MICS), and World Health Surveys (WHS), as well as country-specific survey series such as Kenya Welfare Monitoring Survey and South Africa General Household Survey. To fill the gaps of data in surveys and censuses, we also downloaded and updated HAP estimates from WHO Energy Database and extracted from literature through systematic review. Each nationally or sub-nationally representative data point provided an estimate for the percentage of households using solid cooking fuels. Estimates for the usage of solid fuels for non-cooking purpose were excluded, i.e. primary fuels for lighting. The database, with estimates from 1980 to 2017, contained about 680 studies from 150 countries. As updates to systematic reviews are performed on an ongoing schedule across all GBD causes and risk factors, an update for household air pollution will be performed in the next 1-2 iterations.

Modelling strategy

Household air pollution was modelled at household level using a three-step modelling strategy that uses linear regression, spatiotemporal regression and Gaussian Process Regression (GPR). The first step is a mixed-effect linear regression of logit-transformed proportion of households using solid cooking fuels. The linear model contains maternal education, proportion of population living in urban areas, and lagged-distributed income as covariates and has nested random effect by GBD region, and GBD super region respectively. The full ST-GPR process is specified elsewhere this appendix. No substantial modelling changes were made in this round compared to GBD 2016.

Theoretical minimum-risk exposure level

For cataract, the TMREL is defined as no households using solid cooking fuel. For outcomes that utilise evidence based on the Integrated Exposure Response (IER), the TMREL is defined as uniform distribution between 2.4 and 5.9 ug/m^3.

Relative risks

In GBD 2017, we adopted a new approach for risk attribution using the Integrated Exposure-Response Function (IER). Updates to the IER and the new joint-estimation PAF approach is described in the Ambient Particulate Matter appendix.

The relative risks are provided in the table after the methodology for second-hand smoke exposure and is provided for all air pollution risks.

$PM_{2.5}$ mapping value

In order to use the IER curve, we must estimate the exposure to particulate matter with diameter of less than 2.5 micrometers ($PM_{2.5}$). Since GBD 2015 we have been using a mapping model relying on a database of now almost 90 studies which measures $PM_{2.5}$ exposure in households using solid cooking fuel. Using socio-demographic index and study-level factors as covariates, we predict exposure for all location-years.

In GBD 2017, we updated the model to estimate the individual exposure to $PM_{2.5}$ over and above ambient levels due to the use of solid cooking fuel. We did this by subtracting off the estimated ambient level $PM_{2.5}$ for the location-year of each study in the database before inputting them into the model. By doing this we have independent estimates for $PM_{2.5}$ exposure due to ambient and household solid fuel use.

These exposures are cross-walked to values for men, women, and children by generating the ratio of each group's mean exposure to the overall mean personal exposure. The resulting location, year, sex, and age specific $PM_{2.5}$ exposure values are used as inputs in the IER and attributable burden calculation process.

Ambient Air Pollution

Flowchart

Ambient particulate matter pollution

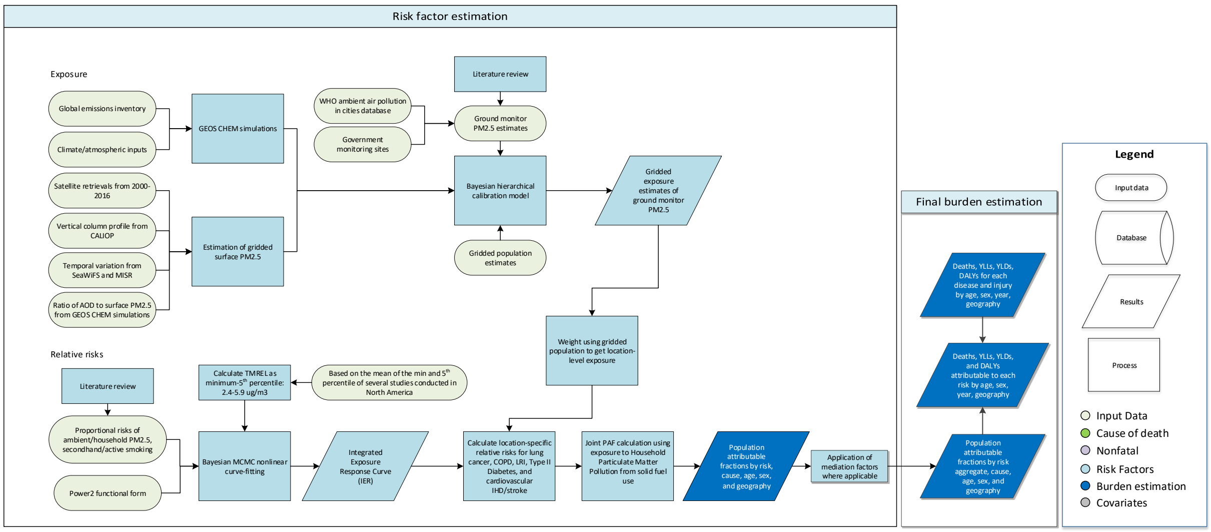

Input data and modeling strategy

Exposure

Definition

Exposure to ambient air pollution is defined as the population-weighted annual average mass concentration of particles with an aerodynamic diameter less than 2.5 micrometers ($PM_{2.5}$) in a cubic meter of air. This measurement is reported in $\mu g/m^3$.

Input Data

The data used to estimate exposure to ambient air pollution is drawn from multiple sources, including satellite observations of aerosols in the atmosphere, ground measurements, chemical transport model simulations, population estimates, and land-use data.

The following details the updates in methodology and input data used in GBD 2017.

$PM_{2.5}$ ground measurement database

Updates of ground measurements used for GBD 2017 include using more recent data than that used previously and the addition of data from new locations. The data from the 2018 update of the WHO Global Ambient Air Quality Database include monitor-specific measurements of concentrations of PM_{10} and $PM_{2.5}$ from 9,960 ground monitors (up from 6,003 in GBD 2016) from 108 countries. The majority of measurements were recorded in 2016 (as there is a lag in reporting measurements, little data from 2017 were available). Annual averages were excluded if they were based on less than 75% coverage within a year. Collection year ranged from 2008 to 2017 in data used. If information on coverage was not available then data were included unless they were already sufficient data within a country (monitor density greater than 0.1).

For locations measuring only PM_{10}, $PM_{2.5}$ measurements were estimated from PM_{10}. This was performed using a hierarchy of conversion factors ($PM_{2.5}/PM_{10}$ ratios): (i) for any location a 'local' conversation factor was used, constructed as the ratio of the average measurements (of $PM_{2.5}$ and PM_{10}) from within 50km and within the same country, if such were available' (ii) if there was not sufficient local information to construct a conversion factor then a country-wide conversion factor was used; and (iii) if there was no appropriate information within a country then a regional factor was used. In each case, to avoid the possible effects of outliers in the measured data (both $PM_{2.5}$ and PM_{10}), extreme values of the ratios were excluded (defined as being greater/lesser than the 95 and 5% quantiles of the empirical distributions of conversion factors) of the latter two cases for the country measurements were available, for both metrics. As in the GBD 2013 and GBD 2015/GBD 2016 databases, in addition to values of $PM_{2.5}$ and whether they were direct measurement or converted from PM_{10}, the database also included additional information, where available, related to the ground measurements such as monitor geo coordinates and monitor site type.

Satellite-based estimates

The updated satellite-based estimates for years 1998-2016 are described in detail in van Donkelaar et al. 2016.[12] These estimates were available at $0.1^{o}\times0.1^{o}$ resolution (~11 x 11 km resolution at the equator) and combine aerosol optical depth retrievals from multiple satellites with the GEOS Chem chemical transport model and land use information.

Population data

A comprehensive set of population data on a high-resolution grid was obtained from the Gridded Population of the World (GPW) database. These estimates are adjusted to match UN2015 Population Prosepectus. These data are provided on a 0.0417^{o}×0.0417^{o} resolution. Aggregation to each 0.1^{o}×0.1^{o} grid cell comprised of summing the central 3 × 3 population cells. As this resulted in a resolution higher than necessary, it was repeated four times, each offset by one cell in a North, South, East and West direction. The average of the resulting five quantities was used as the estimated population for each grid cell. Population estimates for 2000, 2005, 2010, 2015 and 2020 were available from GPW version 4 revision 10. Populations for 2016 and 2017 were obtained by interpolation using natural splines with knots placed at 2000, 2005, 2010, 2015 and 2020. This was performed for each grid cell.

Chemical transport model simulations

Estimates of the sum of particulate sulfate, nitrate, ammonium and organic carbon and the compositional concentrations of mineral dust simulated using the GEOS Chem chemical transport model, and a measure combining elevation and the distance to the nearest urban land surface (as described in van Donkelaar et al. 2016) were available for 2000 to 2016 for each 0.1^{o}×0.1^{o} grid cell. These were not included within the GBD 2013 analysis.

Modelling strategy

Significant advances have been made in the methodology used to estimate exposure to ambient particulate matter pollution since GBD 2013. The following is a summary of the modelling approach, known as the Data Integration Model for Air Quality (DIMAQ) used in GBD 2015, 2016, and 2017.

In GBD 2015 and GBD 2016, coefficients in the calibration model were estimated for each country. Where data were insufficient within a country, information can be `borrowed' from a higher aggregation (region) and if enough information is still not available from an even higher level (super-region). Individual country level estimates were therefore based on a combination of information from the country, its region and super-region. This was implemented within a Bayesian Hierarchical modelling (BHM) framework. BHMs provide an extremely useful and flexible framework in which to model complex relationships and dependencies in data. Uncertainty can also be propagated through the model allowing uncertainty arising from different components, both data sources and models, to be incorporated within estimates of uncertainty associated with the final estimates. The results of the modelling comprise a posterior distribution for each grid cell, rather than just a single point estimate, allowing a variety of summaries to be calculated. The primary outputs here are the median and 95% credible intervals for each grid cell. Based on the availability of ground measurement data, modelling and evaluation was focused on the year 2016.

The GBD 2017 model was updated to also include within country calibration variation. The model used for GBD2017, henceforth referred to as DIMAQ2, provides a number of substantial improvements over the initial formulation of DIMAQ. In DIMAQ, ground measurements from different years were all assumed to have been made in the primary year of interest (i.e. 2014 for GBD2015 before extrapolation) and then regressed against values from other inputs (e.g. satellites etc.) made in that year. In the presence of changes over time therefore, and particularly

in areas where no recent measurements were available, there was the possibility of mismatches between the ground measurements and other variables. In DIMAQ2, ground measurements and matched with other inputs (over time) and the possibility of the (global level) coefficients being allowed to vary over time, subject to smoothing that is induced by a second-order random walk process. In addition, the manner in which spatial variation can be incorporated within the model has developed: where there is sufficient data, the calibration equations can now vary (smoothly) both within and between countries, achieved by allowing the coefficients to follow (smooth) Gaussian processes. Where there is insufficient data within a country, to produce accurate equations, as before information is borrowed from lower down the hierarchy and it is supplemented with information from the wider region.

DIMAQ2 is used for all regions except for the North Africa-Middle East and Sub-Saharan super-regions and remote islands where there is insufficient data to allow the extra complexities of the new model to be implemented. In the North Africa-Middle East and Sub-Saharan super-regions a simplified version of DIMAQ2 is used in which the temporal component is dropped, and for remote islands the original DIMAQ is used.

Due to both the complexity of the models and the size of the data, notably the number of spatial predictions that are required, recently developed techniques that perform 'approximate' Bayesian inference based on integrated nested Laplace approximations (INLA) were used. Computation was performed using the R interface to the INLA computational engine (R-INLA). Fitting the models and performing predictions for each of the ca. 1.4 million grid cells required the use of a high performance computing cluster (HPC) making use of high memory nodes.

Model evaluation

Model development and comparison was performed using within- and out-of-sample assessment. In the evaluation, cross validation was performed using 25 combinations of training (80%) and validation (20%) datasets. Validation sets were obtained by taking a stratified random sample, using sampling probabilities based on the cross-tabulation of $PM_{2.5}$ categories (0-24.9, 25-49.9, 50-74.9, 75-99.9, 100+ µg/m^3) and super-regions, resulting in them having the same distribution of $PM_{2.5}$ concentrations and super-regions as the overall set of sites. The following metrics were calculated for each training/evaluation set combination: for model fit - R^2 and deviance information criteria (DIC, a measure of model fit for Bayesian models); for predictive accuracy - root mean squared error (RMSE) and population weighted root mean squared error (PwRMSE).

All modelling was performed on the log-scale. The choice of which variables were included in the model was made based on their contribution to model fit and predictive ability. The following is a list variables and model structures that were included in DIMAQ.

Continuous explanatory variables:

- (SAT) Estimate of $PM_{2.5}$ (in µgm^{-3}) from satellite remote sensing on the log-scale.
- (POP) Estimate of population for the same year as SAT on the log-scale.
- (SNAOC) Estimate of the sum of sulfate, nitrate, ammonium and organic carbon simulated using the GEOS Chem chemical transport model.

- (DST) Estimate of compositional concentrations of mineral dust simulated using the GEOS Chem chemical transport model.
- (EDxDU) The log of the elevation difference between the elevation at the ground measurement location and the mean elevation within the GEOS Chem simulation grid cell multiplied by the inverse distance to the nearest urban land surface.

Discrete explanatory variables:

- (LOC) Binary variable indicating whether exact location of ground measurement is known.
- (TYPE) Binary variable indicating whether exact type of ground monitor is known.
- (CONV) Binary variable indicating whether ground measurement is $PM_{2.5}$ or converted from PM_{10}.

Random Effects:

- Grid cell random effects on the intercept to allow for multiple ground monitors in a grid cell.
- Country-region-super-region hierarchical random effects for the intercept.
- Country-region-super-region hierarchical random effects for the coefficient associated with SAT.
- Country-region-super-region hierarchical random effects for the coefficient associated with the difference between estimates from CTM and SAT.
- Country-region-super-region hierarchical random effects for the coefficient associated with POP.
- Country level random effects for population uses a neighbourhood structure allowing specific borrowing of information from neighbouring countries.
- Within a region, country level effects of SAT and the difference between SAT AND CTM are assumed to be independent and identically distributed.
- Within a super-region, region level random effects are assumed to be independent and identically distributed.
- Super-region random effects are assumed to be independent and identically distributed.

Interactions*:*

- Interactions between the binary variables and the effects of SAT and CTM.

In addition, DIMAQ2 includes

- Smoothed, spatially varying, random-effects for the intercept
- Smoothed, spatially varying, random-effects for the coefficient of coefficient associated with SAT
- Smoothed, temporally varying, random-effect for the intercept

Results

The final model contained the following variables: SAT, POP, SNAOC, DST, EDxDU, LOC, TYPE, and CONV, together with interactions between SAT and each of LOC, TYPE and CONV. The model structure contained grid cell random effects on the intercept to allow for multiple ground monitors in a grid cell, country-region-super-region hierarchical random effects for intercepts and SAT and country level random effects for population using a neighbourhood structure allowing specific borrowing of information from neighbouring countries together with region-super-region hierarchical random effects for POP. Notably, and as in GBD 2015 and GBD 2016, based on the evaluation of candidate models, including estimates from the TM5 chemical transport model (CTM) used in GBD 2013 did not improve the predictive ability of the model and was therefore not included.

Compared to the model used in GBD2013, DIMAQ showed improved predictions of ground measurements in all super regions with improvements in both within-sample fit; with a global population-weighted RMSE of 12.1 $\mu g/m^3$ compared to 23.1 $\mu g/m^3$ when using the GBD 2013 approach. Using the larger database available for GBD2017, with potentially more variability in measurements, DIMAQ2 shows an additional improvement on DIMAQ: overall population-weighted RMSE reduced from 9.32 to 8.11 (12.12 to 11.17 when using all data, irrespective of within-year coverage). Reductions by super-region can be seen in Figure 1. Reductions can be seen in all super-regions with particular improvement in the Southeast Asia, East Asia and Oceania super-region which is based largely on a substantial increase in accuracy in China, PwRMSE 6 vs 9 $\mu g/m^3$

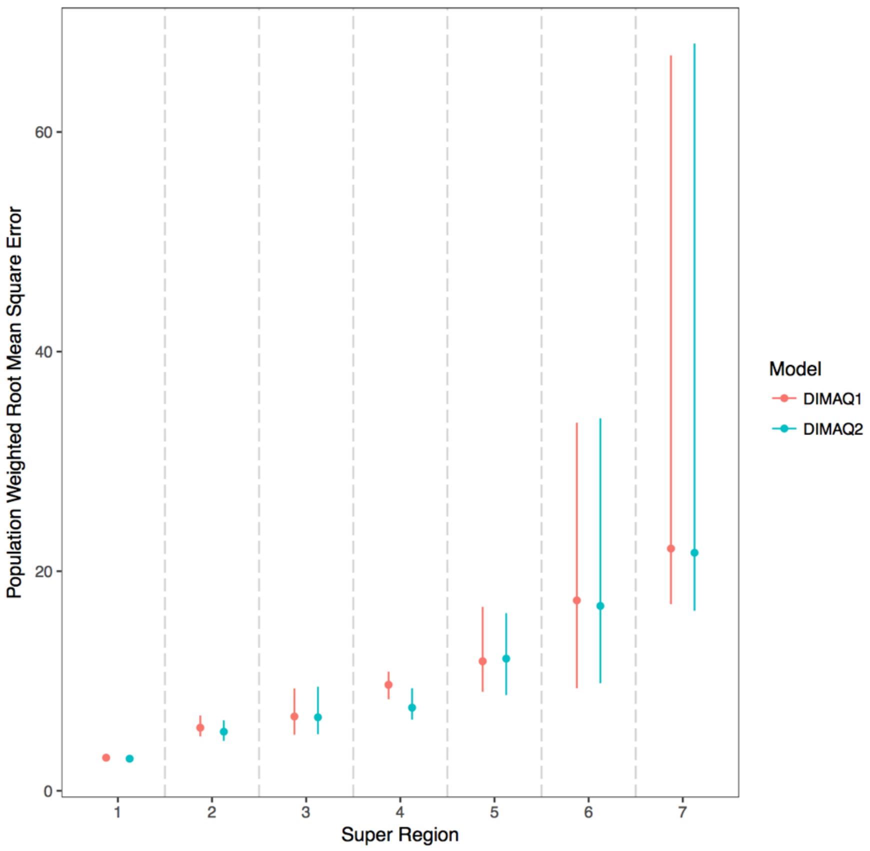

Figure 1: Summary measures of predictive ability, globally and by super-region. Dots denote the median values of population weighted root mean squared error (µg/m³) from 25 validation sets with vertical lines showing the range of values over those sets.

Estimates for other years

In contrast to the method used previously, where estimates (of $PM_{2.5}$) were extrapolated to produce estimates for the year of interest (e.g. 2017 where data was available up to and including 2016) due to the extra complexity of the smooth spatial processes in DIMAQ2 this would not be possible in any straightforward manner. With DIMAQ2 it is the input variables that are extrapolated; this allows estimates for 2017 to be produced in the same way as other years and crucially, allows measures of uncertainty to be produced within the BHM framework rather than by using post-hoc approximations.

Satellite estimates and quantities estimated using the GEOS-Chem model were available for 1990, 1995, 2000, 2005, 2010-2016. Estimates of these input variables for 2017 were produced by extrapolating, on a cell-by-cell basis, using natural splines. Population estimates for 2000, 2005, 2010, 2015 and 2020 were availalble from GPW version 4. For 1990 and 1995 data were extracted from GPW version 3, as in GBD2013.[Error! Reference source not found.] As with populations for 2015, values for each cell for 2011-2017 were obtained by interpolation using natural splines with knots placed at 2000, 2005, 2010, 2015 and 2020.

These were used as inputs to DIMAQ, enabling estimates of exposures to be obtained for each of these years respectively. For 2017, estimates of exposures were obtained from predictions from locally-varying regression models.[13] For each cell a model was fit to the values within that cell over time, with a constraint placed on the rate of change between 2016 and 2017 to avoid unrealistic and/or unjustified extrapolation of trends. Measures of uncertainty were obtained by repeating the procedure for the limits of the 95% credible intervals, again on a cell-by-cell basis.

Population-weighted exposure generation

To generate a distribution of the population-weighted ambient particulate matter, we took a weighted sampling strategy, taking samples from all grid cells in a given location. For example, for a country with n grid cells, we randomly sampled 1000 values from the n (grid cells) x 1000 (samples) where the probability of being sampled was proportional to the population of that grid cell.

Theoretical minimum-risk exposure level

The TMREL was assigned a uniform distribution with lower/upper bounds given by the average of the minimum and 5th percentiles of outdoor air pollution cohort studies exposure distributions conducted in North America, with the assumption that current evidence was insufficient to precisely characterise the shape of the concentration-response function below the 5th percentile of the exposure distributions. The TMREL was defined as a uniform distribution rather than a fixed value in order to represent the uncertainty regarding the level at which the scientific evidence was consistent with adverse effects of exposure. The specific outdoor air pollution cohort studies selected for this averaging were based on the criteria that their 5th percentiles were less than that of the American Cancer Society Cancer Prevention II (CPSII) cohort's 5th percentile of 8.2 based on Turner et al. (2016).[14] This criterion was selected since GBD 2010 used the minimum, 5.8, and 5th percentile solely from the CPS II cohort. The resulting

lower/upper bounds of the distribution for GBD 2017 were 2.4 and 5.9. This has not changed since GBD 2015.

Relative risks and population attributable fractions

We estimated the Ambient Air Pollution-attributable burden of disease based on the relation of long-term exposure to PM2.5 with Ischemic Heart Disease, stroke (ischemic and hemorrhagic), COPD, lung cancer and acute lower respiratory infection. These were also the pollutant-outcome pairs used to estimate the Ambient Air Pollution attributable burden since GBD 2010. For GBD 2017 we also added Type II Diabetes as an outcome of ambient air pollution. We used results from all cohort studies published as of July 2018 that reported cause-specific relative risk estimates based on measured or modelled PM2.5 and that adjusted for potential confounding due to other major risk factors such as tobacco smoking using data for each study participant.

When generating the IER for Type II Diabetes, we included all eight of the studies summarized by Bowe et al. in addition to six other cohorts. Resulting attributable burden estimates were remarkably similar to GBD 2017 results. All citations for studies used in the fitting of the IER curve can be found using the GBD 17 Data Input Sources Tool.

Integrated exposure response function

The Integrated Exposure Response Function (IER) was created to ascertain the shape of the dose response curve for a variety of health outcomes across a wide range of exposure to PM2.5. The IER model is fit by integrating RR information from studies of outdoor air pollution (OAP), Second hand tobacco smoke (SHS), Household Air Pollution (HAP), and Active Smoking (AS). Because OAP studies are often performed at the lower end of the ambient air pollution range, incorporating other exposures to particulate matter enables RR estimation across the global range of exposure. These methods have been described in detail elsewhere.[15,16]

The relative risks are provided in the table after the methodology for second-hand smoke exposure and is provided for all air pollution risks.

Limitations

It is important to recognize the inherent limitations of the IER approach. The use of various sources to construct a risk curve assumes an equitoxicity of particles, consistent with evaluations by US EPA and WHO. However, current evidence suggests there are differences in health impact by source, size, and chemical composition. This is seen when comparing studies of ambient and household particulate matter. As this body of evidence grows, we will continue to re-examine our strategy for the integrated exposure-response curve. For now, the IER is a practical solution to fill gaps in the literature where we do not have sufficient evidence such as household air pollution exposures and ambient in highly polluted areas.

Additionally, currently the exposure concentrations used for both SHS and AS data points when fitting the IER are contrasted with the TMREL and do not take into account ambient particulate matter pollution. In future iterations of fitting the curve, we will test alternate approaches, including a similar approach to HAP, allowing each data point to inform the curve on the range of Exp_{OAP} to (Exp_{OAP} + $Exp_{AS/SHS}$).

Relative risk and proportional PAF approach

For GBD 2017 we developed a new approach to use the IER for obtaining PAFs for both outdoor air pollution (OAP) and household air pollution (HAP). Previously, relative risks for both exposures were obtained from the IER as a function of exposure and relative to the same TMREL. In reality, were a country to reduce only one of these risk factors, the other would remain. We failed to consider the joint effects of particulate matter from outdoor exposure and burning solid fuels for cooking.

In GBD 2017, relative risks were still estimated from the output of the IER curve. Everyone is exposed to some level of OAP, but only a proportion of the population in each location-year use solid cooking fuel and are exposed to HAP. For the proportion of the population not exposed to HAP the relative risk was obtained by $RR_{OAP} = IER(z = Exp_{OAP})$ and used to calculate the PAF for each location based on the population-weighted exposure.

For the proportion of the population exposed to both OAP and HAP, we calculated a joint relative risk from the IER by $RR_{OAP+HAP} = IER(z = Exp_{OAP}+Exp_{HAP})$. This joint relative risk is used to calculate a joint PAF for each location. PAF calculation is detailed in the methods appendix. For each location, we proportioned the joint PAF based on the proportion of exposure due to OAP and HAP respectively. See the table below for equations used to calculate proportional PAFs.

**PAF**	**Population not exposed to HAP**	**Population exposed to HAP**
**OAP**	PAF_{OAP}	$(Exp_{OAP}/(Exp_{OAP}+Exp_{HAP}))*PAF_{OAP+HAP}$
**HAP**	0	$(Exp_{HAP}/(Exp_{OAP}+Exp_{HAP}))*PAF_{OAP+HAP}$

Generally, as expected, this new strategy led to lower PAFs for both ambient and household particulate matter pollution.

Secondhand Smoking

Flowchart

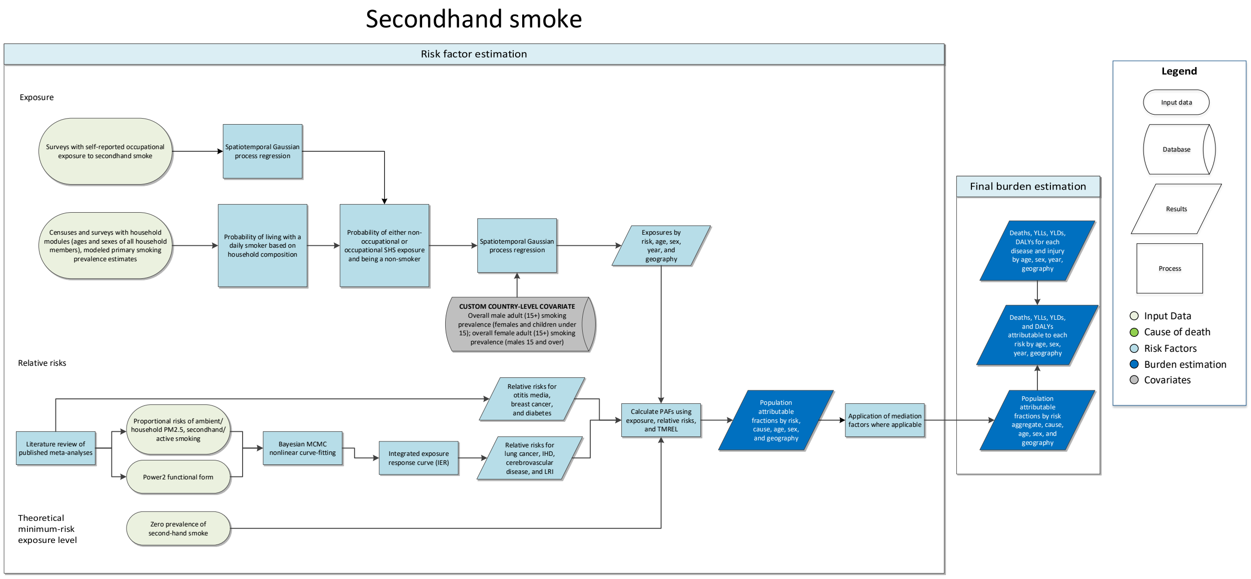

Exposure

Case definition

We define secondhand smoke exposure as current exposure to secondhand tobacco smoke at home, at work, or in other public places. We use household composition as a proxy for non-occupational secondhand smoke exposure and make the assumption that all persons living with a daily smoker are exposed to tobacco smoke. We use surveys to estimate the proportion of individuals exposed to secondhand smoke at work. We only consider non-smokers to be exposed to secondhand smoke. Non-smokers are defined as all persons who are not daily smokers. Ex-smokers and occasional smokers are

considered non-smokers in this analysis. Exposure is evaluated for both children and adults.

Input data

To calculate the proportion of non-smokers who live with at least one smoker, we used unit record data on household composition, which included the ages and sexes of all persons living in the same household. Our sources included representative major survey series with a household composition module, including the Demographic Health Surveys (DHS), the Multiple Indicator Cluster Surveys (MICS), and the Living Standards Measurement Surveys (LSMS); and national and subnational censuses, which included those captured in the IPUMS project and identified using the Global Health Data Exchange catalog (GHDx).

To calculate the proportion of individuals exposed to secondhand smoke at work, by age and sex, we used cross-sectional surveys that ask respondents about self-reported occupational secondhand smoke exposure. Sources include the Global Adult Tobacco Surveys, Eurobarometer Surveys, and WHO STEPS Surveys. We identified sources using the GHDx.

Estimates of primary smoking prevalence in each location were also used in our calculations. Further details on the estimation of primary smoking prevalence can be found in the Smoking methods appendix.

Modelling strategy

We estimated the probability that each person is living with a smoker and is also a non-smoker themselves using set theory. First, household composition data were used at the individual level to capture the ages and sexes of each person in the household. Second, we analyzed surveys with both household composition data and tobacco use questions and determined that the distribution of household size, mean age of the household members, and the age distribution were not significantly different between households with and without a self-reported smoker. Since we did not find that household composition varied between smokers and non-smokers, we then used the GBD 2017 primary smoking prevalence model to calculate the probability that each household member is a smoker. Next, we used the probability of the union of sets on each individual household member to calculate the overall probability that at least one of the other household members was a smoker. We incorporated occupational exposure by modelling prevalence of current exposure to secondhand smoke at work, by age, sex, location, and year, using ST-GPR. In order to avoid double counting we calculated the probability that an individual is exposed through either non-occupational exposure or occupational exposure, given their age, sex, and household composition. Finally, we multiplied this probability of exposure by the probability that the individual is not a smoker themselves (i.e. 1 minus primary smoking prevalence for that person's location,

year, age, and sex). We then collapse these individual-level probabilities to produce average probabilities of exposure by location, year, age, and sex.

These probabilities were modelled in the GBD ST-GPR framework, which generates exposure estimates from a mixed effects hierarchical linear model plus weighted residuals smoothed across time, space, and age. The linear model formula was fit separately by sex using restricted maximum likelihood in R.

We used the sex-specific overall smoking prevalence for adults (age 15 and older) as a country-level covariate in the model. The overall male adult daily smoking prevalence was used as the covariate for females of all ages and for males under age 15. The overall female adult daily smoking prevalence was used as the covariate for males age 15 and older. This was a modelling change from GBD 2015, in which we used the male age-standardised smoking prevalence for the adult female and children under 15 model, and the female age-standardised smoking prevalence for the adult male model.

All input data points from the probability calculation had a measure of uncertainty (variance and sample size) coming from the uncertainty of the primary smoking prevalence model and the sample size from the unit record data going into the modelling process. Geographic r*andom effects were used in model fitting but were not used in prediction.*

Theoretical minimum-risk exposure level

The theoretical minimum-risk exposure level for secondhand smoke is zero exposure among non-smokers, meaning that non-smokers would not live with any primary smokers.

Relative risks

We used country-specific relative risks created using integrated exposure response curves (IER) for PM2.5 air pollution. The relative risks are provided in the table below for all air pollution risks. We used the standard GBD population attributable fraction (PAF) equation to estimate burden based on exposure and relative risks.

Relative risk table for air pollution

The table below provides the relative risks for each category of PM2.5 air pollution. The relative risks are used for indoor air pollution, outdoor air pollution, and second-hand smoke exposure as all three of these risks estimate exposure to PM2.5.

**Air pollution category (PM2.5)**	**Relative risk for lower respiratory infections (95% CI)**
600 µg/m³	2.38<br>(1.968 to 2.776)
500 µg/m³	2.347<br>(1.936 to 2.735)
400 µg/m³	2.297<br>(1.883 to 2.687)
300 µg/m³	2.213<br>(1.809 to 2.615)
200 µg/m³	2.062<br>(1.702 to 2.44)
150 µg/m³	1.938<br>(1.629 to 2.281)
135 µg/m³	1.891<br>(1.6 to 2.209)
120 µg/m³	1.838<br>(1.571 to 2.129)
105 µg/m³	1.778<br>(1.54 to 2.05)

90 µg/m³	1.711<br>(1.505 to 1.945)
75 µg/m³	1.634<br>(1.455 to 1.827)
60 µg/m³	1.546<br>(1.4 to 1.711)
45 µg/m³	1.443<br>(1.323 to 1.576)
30 µg/m³	1.322<br>(1.225 to 1.428)
25 µg/m³	1.276<br>(1.184 to 1.379)
20 µg/m³	1.226<br>(1.14 to 1.335)
15 µg/m³	1.171<br>(1.093 to 1.282)
10 µg/m³	1.108<br>(1.046 to 1.219)
5 µg/m³	1.025<br>(1.0 to 1.119)
0 µg/m³	1.0<br>(1.0 to 1.0)

No Handwashing

Flowchart

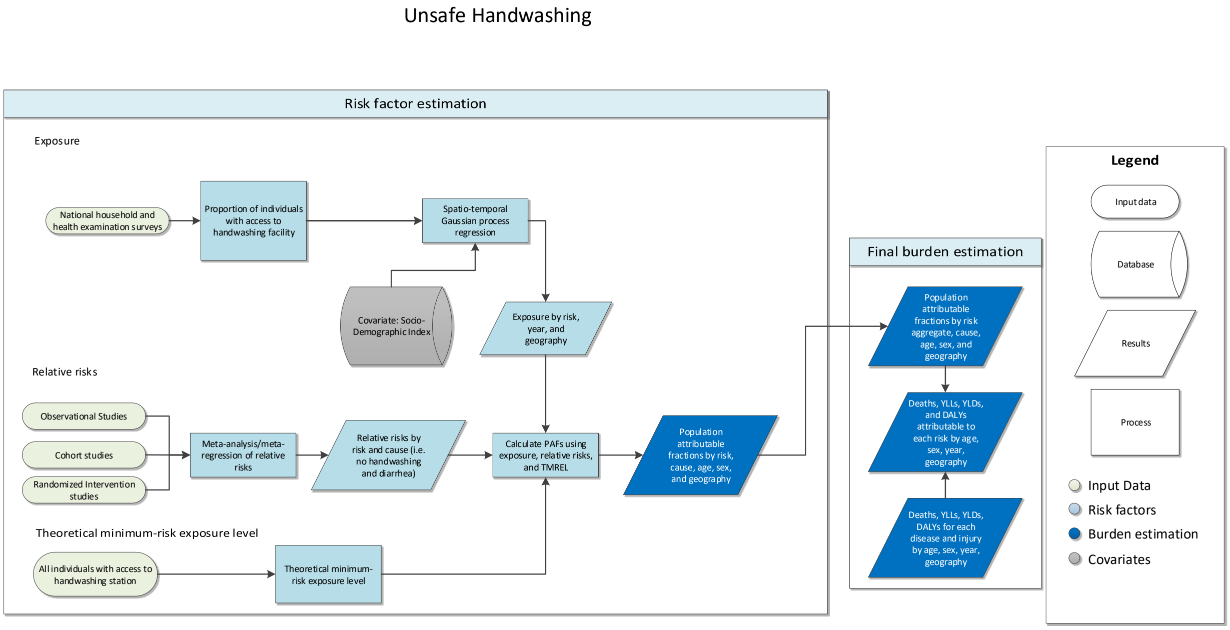

Input data & methodological summary

Exposure

Case definition

Unsafe hygiene is defined as lack of access to a handwashing station with available soap and water. We estimated the burden of unsafe hygiene in both developed and developing settings.

Input data

Since water and soap availability data are very limited, only country-specific Demographic Health Surveys (DHS) and Malaria Indicator Survey Series (MICS) conducted after 2006 were included as input data.

Modelling strategy

By year and location, proportion of households with handwashing facility is modelled using a 3-step modelling scheme of mixed effect linear regression followed by spatio-temporal Gaussian process regression (ST-GPR), which outputs full time series estimates for each GBD 2017 location. Socio-demographic index (SDI), a composite index that include income per capita, education, and fertility, was set as a fixed effect in the linear regression since it proved to have significant coefficient. Random effects were set at GBD 2017 region and super-region levels to fit the model but were not used in the predictions.

The process of vetting and validating models was accomplished primarily through an examination of ST-GPR scatter plots by GBD 2016 location from 1990-2016. Any data

points lacking face validity were re-inspected for error at the level of extraction and survey implementation, and subsequently excluded from analysis if deemed appropriate. In addition to SDI, a number of different potential fixed effects were considered, including lag-distributed income and urbanicity. However, SDI proved to be the strongest predictor.

A considerable limitation for when estimating handwashing practices for over 190 independent locations around the world was data sparseness. Even when data were published on handwashing prevalence, the definition was often altered from the GBD 2017 standard definition or it may only have pertained to certain populations (such as hospital patients) and lacked representativeness at the geographic scale we required. The incorporation of questions about soap and water availability in DHS and MICS added much-needed information but there remains a large data gap to be filled if we are to become more certain in handwashing access estimates.

Theoretical minimum-risk exposure level

The theoretical minimum-risk exposure level for unsafe hygiene is defined as all individuals with access to handwashing facility after any contact with excreta, including children's excreta.

Relative risks

A meta-analysis by Rabie and Curtis[17] provided relative risk evidence for the relationship between lack of facility access and lower respiratory infection (relative risk of LRI given lack of access to hand washing facility was 1.19, 95% CI 1.12 to 1.27).

Antibiotics for LRI

Flowchart

Treatment for Childhood Illness Flowchart

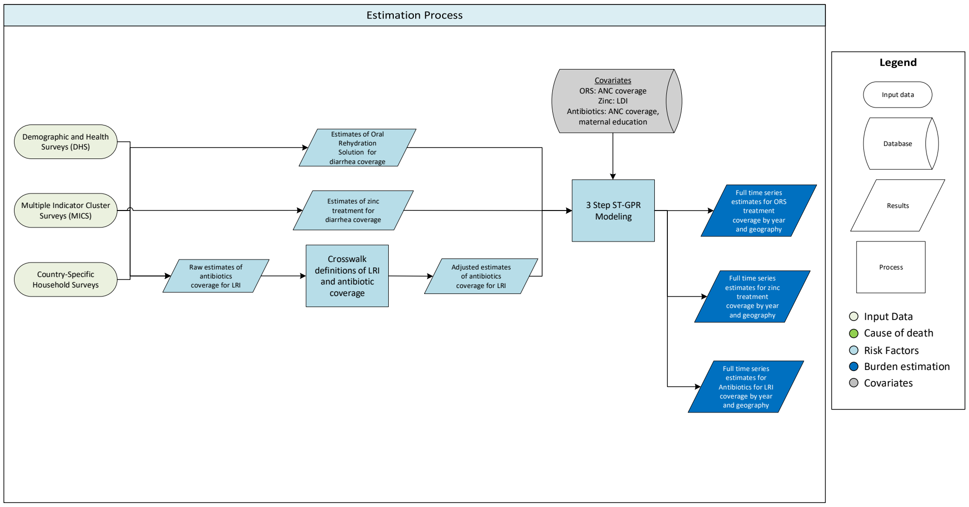

Definition of Indicators

Antibiotics for lower respiratory infection (LRI): Antibiotics for lower respiratory infection (LRI) is defined as the proportion of children ages 0-4 years with *suspected* lower respiratory infection in the past two weeks who received antibiotics for treatment. Suspected LRI is based on a combination of reported symptoms, including cough, difficulty breathing, chest symptoms, and fever, cross-walked to a gold standard definition that includes all of these symptoms.

Input Data

Data Source Identification

Across all childhood illness care indicators, we use individual-level microdata from population health surveys. Individual-level data comes primarily from major multi-country surveys that survey women on recent illnesses of children in their household as well as any care received, including Demographic and Health Surveys (DHS),[18] Multiple Indicator Cluster Surveys (MICS),[19] Reproductive Health Surveys (RHS),[20] and Living Standards Measurement Study (LSMS) surveys.[21] We supplement these with microdata from individual country-specific surveys.

Indicator	No. of Observations	No. of Studies	Year Range
Antibiotics for LRI	413	243	1986-2016

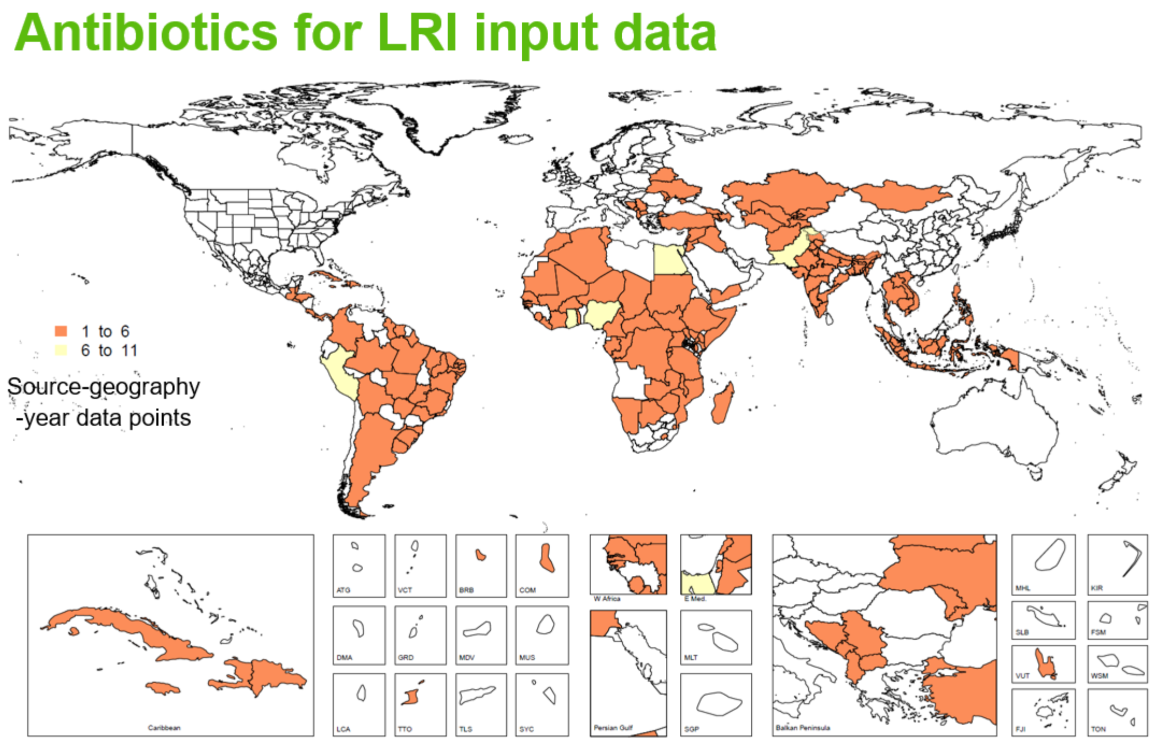

s

Indicator and Source Metadata

Many household surveys collect information on maternal and child health (MCH) indicators for children under 5 and/or mothers who gave birth within five years prior to the time of survey. We include surveys that are geographically representative and rely on maternal-reported care received among children with illness in the prior two weeks, including treatment received for suspected pneumonia. We exclude all data sources that are not nationally representative (but include surveys that are only subnationally representative if they are from one of the subnational units estimated in GBD) as well as surveys with high levels of missingness.

Antibiotic treatment for suspected LRI coverage data is extracted as care received for suspected pneumonia among children reported to have had pneumonia symptoms in the two weeks prior to survey. For all treatment indicators, country- and survey-specific treatments reported in surveys are mapped to broad treatment categories.

Indicator	Numerator	Denominator	Definition
Antibiotics for LRI	# of children with suspected LRI in past 2 weeks who received antibiotics	# of total children with suspected LRI in the past 2 weeks	Proportion of all children 0-4 years who had suspected LRI in the past 2 weeks who received antibiotics

Modeling strategy

We estimated Antibiotics coverage by location and year using Spatiotemporal Gaussian Process Regression (ST-GPR), a three-stage modeling tool used extensively in GBD. We used maternal education and more than 1 antenatal care visit proportion as covariates in our model. The second step of ST-GPR takes the residuals of the linear regression and smooths them based on proximity in space and time. The third step is Gaussian process smoothing that takes into account the uncertainty in the data points and estimates. Random draws of 1,000 samples were obtained from the final distribution for every country and year. Ninety-five percent uncertainty intervals were calculated by taking the ordinal 25th and 975th draws from the sample distribution.

Relative risks for LRI mortality are from Theodoratou et al.[22] and based on 9 before-after studies evaluating their impact (RR 0.65, 95% CI 0.52-0.82).

Haemophilus influenzae type B and Pneumococcal Conjugate Vaccine coverage

Indicator definition

This modeling strategy pertains to measures of vaccine coverage, the proportion of the target population receiving two or three doses of the Haemophilus influenzae type B (Hib) vaccine or Pneumococcal Conjugate Vaccine (PCV). These two vaccines follow the same general structure, described below, but the ratio of Hib and PCV to DTP3 coverage are modeled independently.

Input data

The present study used data from household-level surveys as well as administrative reports of immunization coverage. Survey data which provided person-level information on immunization were identified and extracted. Major multi-country survey programs included in the analysis include the Demographic and Health Surveys (DHS),[23] Multiple Indicator Cluster Surveys (MICS),[24] Reproductive Health Surveys (RHS),[25] Living Standards Measurement Study (LSMS) surveys,[26] and World Health Surveys (WHS).[27] We also conducted a comprehensive search of the Global Health Data Exchange (GHDx),[28] as well as targeted internet searches and review of Ministry of Health websites, to identify national surveys and other multi-country survey programs.

Administrative estimates of immunization coverage were obtained from the Joint Reporting Form (JRF),[29] through which the World Health Organization (WHO) and UNICEF collate annual estimates of immunization coverage reported by UN member states. These immunization coverage estimates are separate from those synthesized by WHO, and are calculated by dividing the number of doses of a given vaccine delivered to the target population (i.e., children aged 12 to 23) by the number of individuals in that target population.

We excluded all data sources that were not nationally representative or had high levels of missingness. We applied survey weights based on survey sampling frames whenever they were available to generate weighted national estimates of vaccination coverage accompanied by estimates of standard error (SE). Estimates of SE, as well as sample sizes, were used to calculate uncertainty, as described below. Any point estimates with sample sizes less than 50 were reviewed to ensure that were not substantive outliers and would otherwise have an undue influence on our analysis.

Modeling strategy

Data processing

Age splitting

Most household surveys collect information on maternal and child health (MCH) indicators for children under 5 and/or mothers who gave birth within five years prior to the time of survey. To maximize data use for our model, we included immunization data for children aged 12 to 59 at the time of survey. Children younger than 12 months of age were excluded to minimize the influence of potentially censored observations. For each vaccine, coverage estimates were assigned to birth-cohort years based on a child's age prior to the time of survey: we used responses recorded for children aged 12 to 23 months for immunization coverage for one year prior to the time of survey, children aged 24 to 35 months for coverage two years prior to the time of survey, and so forth.

Age-specific estimates are easily computed from individual-level microdata, but many published reports and survey summaries present data in broader age aggregates (e.g. coverage for children aged 12 to 35 months). To standardize these age groups, we applied an age-splitting model used in the GBD study[30] and other analyses that generated smoking and obesity prevalence by age group.[31,32]

Using surveys with microdata as the reference, we used the following model to generate standardized age group-specific estimates of immunization coverage:

$$\tilde{P}_{a,c,t,k} = P_{a,c,t,k}^{a+x} \frac{P_{a,c,t,j}}{P_{a,c,t,j}^{a+x}}$$

where $\tilde{P}_{a,c,k}$ is the adjusted estimate of coverage for target age group a in country c and year t of survey k; and $P_{a,c,k}^{a+x}$ is coverage reported from survey k, for country c in year t for the age group spanning age a to age $(a + x)$. The ratio of coverage between the target age group and broader age group from a survey j with microdata from the same country-year was used to split data from survey k. Surveys to be split were ideally matched with DHS or MICS surveys. If microdata were not available for the same year, ratios within five years of the survey that required age-splitting were applied.

Administrative bias adjustment

Intervention coverage estimates based on administrative sources can be biased. Such biases may arise for a number of reasons, including discrepancies in the accurate reporting of services or interventions provided (e.g. number of vaccine doses administered) and target population (e.g. number of children in need of vaccines), as well as capturing these data in a timely manner from both public and private-sector facilities and health care providers. We implemented a vaccine-specific bias adjustment process to account for bias in administrative reports of immunization coverage in the JRF for DTP3 vaccine coverage. Given that the magnitude, direction, and cause of such biases are heterogeneous across space, time, and antigen,[33,34] a vaccine-specific, time-varying, all-location bias correction factor was used.

For immunization coverage, we view individual-level data collected through population health surveys as the most accurate and least biased source of information of vaccination coverage, particularly for geographies with incomplete health information systems. We thus compute administrative bias as the ratio between estimates of coverage from surveys (where available) and matched administrative coverage. We model this bias in a spatiotemporal Gaussian process regression (ST-GPR) framework, described further in the subsequent section of this appendix, using the Socio-demographic index (SDI) as a predictor. This method allows us to estimate antigen-specific administrative bias factors for all geographies and years since 1980, even in places without survey data, by borrowing strength in data across space and time. The GPR framework properly estimates prediction errors in the data synthesis procedure by for uncertainty in bias ratios when generating fitted values. In this framework, more weight is given to survey data with less uncertainty.

Antigen-specific modeled estimates of administrative bias are then used to adjust administrative coverage data for over- or under-reporting to reflect observed survey coverage. Adjusted administrative data are used as inputs into the trend estimation process.

Although we only directly perform an administrative bias adjustment on data for DTP3, our ratio-based modeling strategy for the other antigens (described further below) leverages the bias-corrected administrative data by multiplying the modeled ratio by the coverage of the denominator in the final step.

Trend estimation

We used a spatiotemporal Gaussian process regression (ST-GPR) to synthesize point estimates from multiple data sources and derive a complete time series for each vaccine. This method has been used extensively GBD and related studies, and accounts for uncertainty pertaining to each point estimate while borrowing strength across geographic space and time.[10, 11,15,16] Briefly, we assumed the Gaussian process was defined by a mean function $m(\bullet)$ and covariance function $Cov(\bullet)$.

We estimated the mean function using a two-step approach. Specifically, $m_c(t)$ can be expressed as:

$$m_c(t) = X\beta + h(r_{c,t})$$

where $X\beta$ is a linear model and $h(r_{c,t})$ is a smoothing function for the residuals; and $r_{c,t}$ is derived from the linear model. The following linear model was used to model DPT3 coverage:

$$logit(P_{c,t}) = \beta_0 + \beta_1 \mathrm{HAQ}_{\mathrm{c,t}} + \alpha_c + \gamma_{R[c]} + \omega_{\mathrm{SR[c]}} + \varepsilon_{c,t}$$

where $P_{c,t}$ is vaccination coverage for country c year t; $HAQ_{c,t}$ is value of the Healthcare Access and Quality Index[36] for country c and year t; α_c, $\gamma_{R[c]}$, and $\omega_{SR[c]}$ are country, region, and super-region random intercepts, respectively. These estimates were then modeled through ST-GPR.

Given their recent introduction, there are limited data on coverage Hib3, PCV3, and rotavirus vaccines. To leverage the relatively data-rich DTP3 estimates, we modeled the scale-up of each vaccine over time by modeling their ratio with the more data-rich DTP3 vaccine coverage. We first calculated the ratio of vaccine coverage data (including survey data and uncorrected administrative data) with DTP3 by survey-year. Uncorrected administrative data are before we apply bias corrections to these data, as described above. We then modeled the full time series of the ratios using ST-GPR. The following linear model was used as the mean function for the Hib, PCV, and Rota ratios with DPT3:

$$logit\left(P_{c,i}\right) = \beta_0 + \beta_1 \mathrm{HAQ}_{\mathrm{c,i}} + \alpha_c + \gamma_{R[c]} + \omega_{\mathrm{SR[c]}} + \varepsilon_{c,i}$$

where $P_{c,i}$ is the coverage ratio for country c time since introduction i; $HAQ_{c,i}$ is value of the Healthcare Access and Quality Index[36] for country c and time since introduction i; α_c, $\gamma_{R[c]}$, and $\omega_{SR[c]}$ are country, region, and super-region random intercepts, respectively. We ultimately obtained estimates of coverage by multiplying the modeled ratio by the final estimated DTP3 coverage by location and year.

Random draws of 1,000 samples were obtained from the distributions above for every country for a given vaccine. Ninety-five percent uncertainty intervals were calculated by taking the ordinal 25th and 975th draws from the sample distribution.

To assess the accuracy of our modeled estimates, we performed cross-validation analyses using a knockout structure as previously described[37]. ST-GPR hyperparameters were selected on models that minimized the overall root-mean squared error (RMSE) of the model across a set of 10 knockouts.

Routine introduction

National vaccine schedules and vaccine introduction dates were used as reported from WHO[38] or from the country's Ministry of Health website where otherwise unavailable.

Relative Risks

The vaccine efficacy for Hib vaccine and PCV are from a meta-analysis performed for the GBD and updated in GBD 2017, described in previous publications.[39] The vaccine efficacy for Hib vaccine against pneumonia was 76.6% (95% UI 47.2 to 99.0%). The vaccine efficacy for PCV against pneumonia was 71.0% (95% UI 66.3 to 75.8%).

Zinc Deficiency

Exposure

Case definition

Exposure to zinc deficiency is defined as consumption of less than 2.5 milligrams of zinc per day among children between the ages of 1 and 4 years old.

Input data

We used dietary data from nationally and sub-nationally representative nutrition surveys and United Nations FAO Supply and Utilization Accounts to estimate the mean intake of zinc at the population level.

Modelling strategy

For GBD 2016, we first used a spatio-temporal Gaussian process regression (ST-GPR) framework to estimate the mean intake of zinc by age, sex, country, and year. To assist with estimation for locations and years without data, we used the lag-distributed income of that location-year as a covariate. We considered data from 24-hour diet recall as the gold standard, and adjusted data from other sources to the gold standard method. Using the method described in the dietary risks section, we characterised the distribution of zinc intake for children between ages of 1 and 4 years old and estimate the proportion of the children with intake of less than 2.5 milligrams of zinc per day.

Relative risk

Relative risks used for zinc deficiency is based on the results of randomised trials that measured the effect of zinc supplementation. The relative risk for LRI morbidity given zinc deficiency was 1.84 (95% CI 1.28 to 2.52) and the relative risk for LRI mortality given zinc deficiency was 1.67 (95% CI 0.46 to 4.14).

Theoretical minimum-risk exposure level

The theoretical minimum-risk exposure level for proportion zinc deficient is zero percent deficient.

Low Birth Weight and Short Gestation

Flowchart

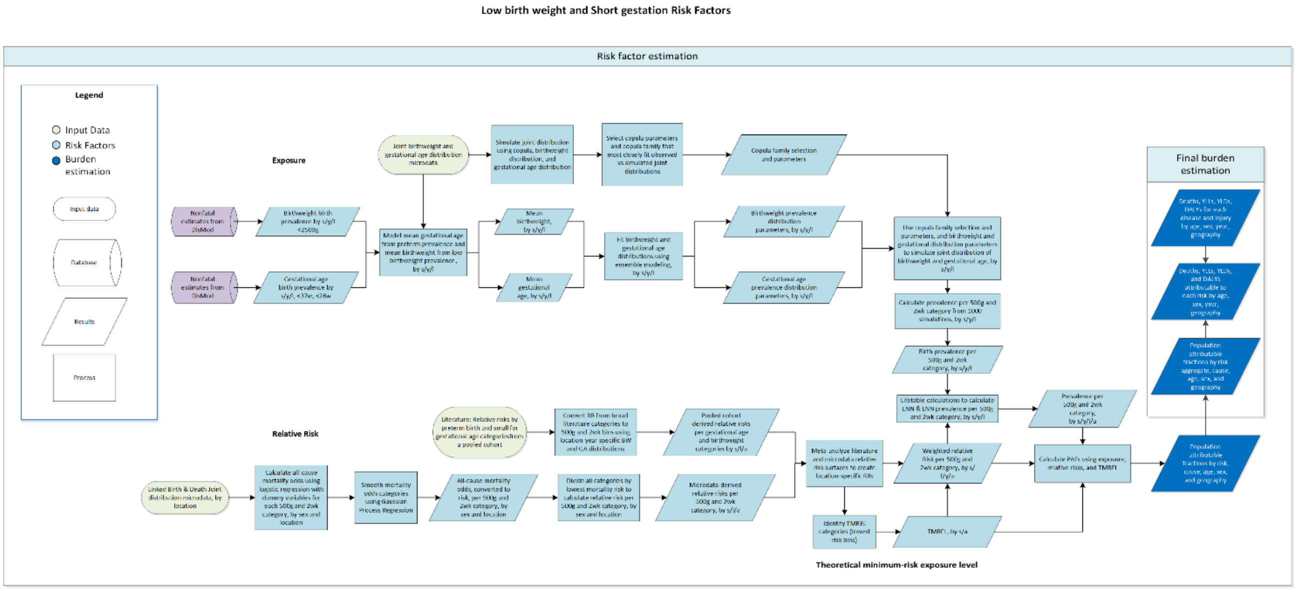

Input data and methodological summary

The “Low Birth Weight and Short Gestation” (LBWSG) risk factor and its child risks “Low Birth Weight for Gestation” and “Short Gestation for Birth Weight” first were included as risk factors in GBD 2016.

Although low birth weight for gestation and short gestation for birth weight are separate risk factors, the exposures and relative risks for both are estimated jointly through the low birth weight and short gestation parent risk factor. As of GBD 2017, LBWSG are the only risk factors estimated jointly.

Case definition

The meaning of the “low birth weight” and “short gestation” in GBD have subtle definitional differences compared to other usages of “low birth weight” and “short gestation” in literature. The term “low birth weight” has historically been used to refer to birth weight (BW) less than 2500 grams. However, because the goal of the GBD risk factors analysis is to quantify the entirety of attributable burden due to each risk factor, the GBD definition of “low birth weight” therefore refers to all birth weight below the Theoretical Minimum Risk Exposure Level (TMREL) for birth weight. Likewise, new-borns have been typically been classified into gestational age (GA) categories of “extremely preterm” (<28 weeks of gestation), “very preterm” (28-<32 weeks of gestation), and “moderate to late preterm” (32-<37 weeks of gestation). “Short gestation” in GBD refers to all gestational ages below the gestational age TMREL.

Exposures and relative risks for the GBD Low birth weight and short gestation risk factors are divided into joint 500-gram birth weight and 2-week gestational age combinations. The lowest risk overall 500-gram/2-week bin is the overall TMREL. The univariate TMRELs vary with GA and BW. The lowest risk GA varies by BW category and the lowest risk BWs vary with GA category. The latter are used to quantify univariate attributable risk. Under this framework, all attributable burden under the joint TMREL is referred to jointly as burden of LBWSG. All attributable burden to BWs under the TMREL for each GA category are, on aggregate, “low birth weight” and all attributable burden to GAs under the TMREL for each BW category are, on aggregate, “short gestation.” Each combination of 500-grams and 2-wks is associated with a relative risk for mortality by neonatal period (early and late neonatal) and by the causes listed in Table 2 and described below, and relative to the joint TMREL.

Exposure

Input data

To model the joint distribution of exposure of low birth weight and short gestation for each location, year, and sex estimated in GBD 2017, three types of information are used:

- Distribution of gestational age for each location, year, and sex
- Distribution of birth weight for each location, year, and sex

- Copula family and parameters, specifying correlation between gestational age and birth weight distributions

Modelling strategy

Distributions of birth weight & gestational age

To model the joint distribution of birth weight and gestational age for every location-sex-year, ensemble model methods standard to GBD risk factors (described elsewhere in the methods appendix), are first used to create separate distributions of birth weight and gestational age for every location-sex-year.

Microdata is the most ideal data source for modelling distributions; however, microdata is not widely available for birth weight and is more scarce for gestational age. Categorical prevalence data is much more readily available, and from a wider range of locations and years, for low birth weight (<2500g), extremely preterm (<28 weeks of gestation), very preterm (28-32 weeks of gestation), moderate to late preterm (32-37 weeks of gestation), and preterm birth (<37 weeks of gestation). From GBD 2010 to GBD 2015, this categorical data has been used model birth prevalence of preterm birth by gestational age (<28 weeks, 28-<32 weeks, and 32-<37 weeks) and low birth weight (<2500g) for every location, sex, and year estimated in GBD. Starting in GBD 2016 with the introduction of the LBWSG risk factors, the full distributions at birth have been modelled for gestational age and birth weight for all GBD locations, estimation years, and both sexes. The gestational age and birth weight distributions are then aggregated into the categorical estimates of <28 weeks, 28-<32 weeks, 32-<37 weeks gestation, and <2500 g birth weight.

Ensemble model methods standard to GBD are used to model the distribution at birth of gestational age and birth weight. Gestational age ensemble distribution models use the prevalence of <37 weeks gestation, the prevalence of <28 weeks gestation, and mean gestational age per each location-year-sex as inputs into the model. Birth weight distribution models use the prevalence of <2500 grams birth weight and mean birth weight per each location-year-sex. Prevalence of <37 weeks gestation and of <2500 grams birth weight was estimated for all location-year-sexes using STGPR modelling processes standard to GBD.

Low birth weight (<2500 grams) data was extracted from literature, vital registration systems, and surveys. DHS survey data were observed to have high missingness; to correct for the missingness, birth weight was imputed using the Amelia package in R. Birth weight was predicted using standard Amelia imputation methods from the following variables also in the DHS surveys: urbanicity, sex, birthweight recorded on card, birth order, maternal education, paternal education, child age, child weight, child height, mother's age at birth, mother's weight, shared toilet facility, and household water treated. Data counts for categorical prevalence models are listed in Table 1.

Table 1: Data Counts for Categorical Prevalence Models

	<28 weeks	<37 weeks	<2500 grams
**Site-years (total)**	1872	2420	2980
**Number of GBD regions with data (out of 21 regions)**	14	21	21
**Number of GBD super-regions with data (out of 7 super-regions)**	6	7	7

Global ensemble weights for gestational age were derived by using a 3 million sample of all available microdata in Table 2 to select the ensemble weights. Of the exponential, gamma, inverse gamma, Weibull, log normal, and normal distributions, the three distribution families that received the highest weights were the Weibull (87%), normal (4%), and inverse gamma (4%) distributions. Global ensemble weights for birth weight were derived using a 3 million sample of all available microdata in Table 2, in addition to birth weight microdata available primarily through the DHS and MICS surveys. Of the exponential, gamma, inverse gamma, Weibull, log normal, and normal distributions, the three distribution families that received the highest weights were the log normal (38%), normal (32%), and Weibull (20%) distributions.

Ordinary least squares was used to model mean gestational age for all location-year-sexes by regressing mean gestational age on prevalence of <37 weeks gestation per location-year. All available microdata (Table 2) was used to fit the model. OLS was also used to model mean birth weight by regressing prevalence of <2500 g birth weight per location-year. All available joint microdata (Table 2), as well as additional birth weight microdata extracted primarily through DHS and MICS surveys, was used to fit the model. As estimates of prevalence of <37 weeks gestation and prevalence of <2500g birth weight are available for all location-year-sexes through STGPR models, mean gestational age and mean birth weight were predicted for all location-year-sexes.

Copula optimisation

In order to model the joint distribution of gestational age and birth weight from separate distributions, information is needed about the correlation between the two distributions. Distributions of gestational age and birth weight are not independent; the Spearman correlation for each country where joint microdata was available (Table 2), pooling across all years of data available, ranged from 0.25-0.49. The overall Spearman correlation was 0.38, pooling across all countries in the dataset.

Table 3: Summary of Data Inputs

Location	Years of data	Total births*	Format of data	Spearman correlation	Used in Ensemble Weight Selection	Used in Copula Parameter Selection	Used in Relative Risk Models
BRA	2016	2,854,380	Microdata	0.37	Yes	Yes	No
ECU	2003-2015	2,473,039	Microdata	0.34	Yes	Yes	No
ESP	1990-2014	8,537,220	Microdata	0.42	Yes	Yes	No
JPN	1995-2015	23,644,506	Tabulations	0.41	No	No	Yes
MEX	2008-2012	10,256,117	Microdata	0.35	Yes	Yes	No
NOR	1990-2014	1,489,210	Microdata	0.44	Yes	Yes	Yes
NZL	1990-2016	1,600,501	Microdata	0.25	Yes	Yes	Yes
SGP	1993-2015	972,775	Tabulations	0.41	No	No	Yes
TWN	1998-2002	1,331,760	Tabulations	0.38	No	No	Yes
URY	1996-2014	698,622	Microdata	0.49	Yes	Yes	No
USA	1990-2014	81,929,879	Microdata	0.38	Yes	Yes	Yes

** Pooled across all year and sexes, excluding data missing year of birth, gestational age, or birth weight*

Copula modelling is used to model joint distributions between the birth weight and gestational age marginal distributions. The Copula and VineCopula packages in R were used to select the optimal copula family and copula parameters to model the joint distribution, using joint microdata from the country-years in Table 2. The copula family selected from the microdata was "Survival BB8", with theta parameter set to 1.75 and delta parameter set to 1.

The joint distribution of birth weight and gestational age per location-year-sex was modelled using the global copula family and parameters selected and the location-year-sex gestational age and birth weight distributions. The joint distribution was simulated 100 times to capture uncertainty. Each simulation consisted of 100,000 simulated joint birth weight and gestational age data points. Each joint distribution was divided into 500g by 2wk bins to match the categorical bins of the relative risk surface. Birth prevalence was then calculated for each 500g by 2wk bin.

Estimating Early Neonatal Prevalence & Late Neonatal Prevalence from Birth Cohorts

Early neonatal prevalence and late neonatal prevalence was estimated using life table approaches for each 500g & 2wk bin. Using the all-cause early neonatal mortality rate for each location-year-sex, births per location-year-sex-bin, and the relative risks for each location-year-sex-bin in the early neonatal period, the all-cause early neonatal mortality rate was calculated for each location-year-sex-*bin*. The early neonatal mortality rate per bin was used to calculate the number of survivors at 7 days and prevalence in the early neonatal period. Using the same process, the all-cause late neonatal mortality rate for each location-year-sex was paired with the number of survivors at 7 days and late neonatal relative risks per bin to calculate late neonatal prevalence and survivors at 28 days.

Relative risks & theoretical minimum-risk exposure level

Input data

In the Norway, New Zealand, and US Linked Birth/Death Cohort microdata datasets, live births are reported with gestational age, birth weight, and an indicator of death at 7 days and 28 days. For this analysis, gestational age was grouped into two-week categories, and birth weight was grouped into 500-gram categories. The Taiwan, Japan, and Singapore datasets were prepared in tabulations of joint 500-gram and two-week categories.

Modelling strategy

For each location, data was pooled across years, and the risk of all-cause mortality at the early neonatal period and late neonatal period at joint birth weight and gestational age combinations was calculated. In all datasets except for the United States, sex-specific data were combined to maximise sample size. The United States analyses were sex-specific. To calculate relative risk at each 500g and 2wk combination, logistic regression was first used to calculate mortality odds for each joint 2-week gestational age and 500-gram birth weight category. Mortality odds were smoothed with Gaussian Process Regression, with the independent distributions of mortality odds by birth weight and mortality odds by gestational age serving as priors in the regression.

A pooled country analysis[40] of mortality risk in the early neonatal period and late neonatal period by SGA category in developing countries in Asia and Sub-Saharan Africa were also converted into 500-gram and 2-week bin mortality odds surfaces. The relative risk surfaces produced from microdata and the Asia and Africa surfaces produced from the pooled country analysis were meta-analyzed, resulting in a meta-analysed mortality odds surface for each location. The meta-analysed mortality odds surface for each location was smoothed using Gaussian Process Regression and then converted into mortality risk. To calculate mortality relative risks, the risk of each joint 2-week gestational age and 500-gram birth weight category were divided by the risk of mortality in the joint gestational age and birth weight category with the lowest mortality risk.

For each of the country-derived relative risk surfaces, the 500 g and 2-week gestational age joint bin with the lowest risk was identified. This bin differed within each country dataset. To identify the universal 500 g and 2-week gestational age category that would serve as the universal TMREL for our analysis, we chose the bins that was identified to be the TMREL in each country dataset to contribute to the universal TMREL. Therefore, the joint categories that served as our universal TMREL for the LBWSG risk factor were “38-40 weeks of gestation and 3500-4000 grams", "38-40 weeks of gestation and 4000-4500 grams", and "40-42 weeks of gestation and 4000-4500 grams". As the joint TMREL, all three categories were assigned to a relative risk equal to 1.

PAF calculations

The total PAF for the low birth weight and short gestation joint risk factor is calculated by summing the PAF calculated from each 500g x 2wk category, with the lowest risk category among all the 500g x 2wk categories serving as the TMREL. The equation for calculating PAF for each 500g x 2wk category is:

$$PAF_{joasgt} = \frac{\sum_{x=1}^{u} RR_{joast}(x)P_{jasgt}(x) - RR_{joasg}(TMRE_{jas})}{\sum_{x=1}^{u} RR_{joas}(x)P_{jasgt}(x)}$$

To calculate the overall PAF for the short gestation for birth weight risk factor, PAF was once again calculated for each joint 500-gram and 2-week category. Unlike the joint PAF calculation, which used only one TMREL for all 500-gram and 2-week categories, the joint 500-gram and 2-week category with the lowest risk for each 500-gram birth weight grouping served as the TMREL for that 500-gram birth weight grouping. For example, the [3000, 3500) gram birth weight grouping contains five joint categories: [34, 36) weeks and [3000, 3500) grams; [36, 37) weeks and [3000, 3500) grams; [37, 38) weeks and [3000, 3500) grams; [38, 40) weeks and [3000, 3500) grams; and [40, 42) weeks and [3000, 3500) grams. The [40, 42) weeks and [3000, 3500) grams joint category has the lowest risk, and so it serves as the TMREL for the [3000, 3500) gram birth weight grouping. In the Relative Risk surface figures, a birth weight grouping is one "column" of the birth weight and gestational age matrix.

The overall PAF for the short gestation for birth weight risk factor was then calculated for all the joint 500-gram and 2-week categories using the formula below:

$$PAF_{1..i} = 1 - \prod_{i=1}^{n}(1 - PAF_i)$$

The same methodology was applied to calculate the total PAF for the low birth weight for gestation risk factor, using two-week gestational age categories (each "row" of the matrix) instead of 500-gram birth weight categories. For example, the [24, 26) weeks gestational age grouping contains three joint categories: [0, 500) grams and [24, 26) weeks; [500, 1000) grams and [24, 26) weeks; and [1000, 1500) grams and [24, 26) weeks. The [1000, 1500) grams and [24, 26) weeks joint category has the lowest risk, and so it serves as the TMREL for the [24, 26) weeks gestational age grouping.

After the short gestation for birth weight PAF and low birth weight for gestational age PAF were calculated, they were then scaled so that the sum of the short gestation for birth weight PAF and low birth weight for gestation PAF equal the low birth weight and short gestation parent PAF calculated for each location/year/sex/age group.

Table for the relative risks for LRI mortality given low birthweight and short gestation joint categories by sex and early neonatal and late neonatal age groups.

Category	Sex	Early neonatal (0-6 days)	Late neonatal (7-27 days)
Birth prevalence - [0, 24) wks, [0, 500) g	Males	1564.792 (1056.542 to 2116.062)	618.595 (458.842 to 812.921)
Birth prevalence - [0, 24) wks, [0, 500) g	Females	1600.122 (1050.664 to 2211.877)	713.571 (526.178 to 921.018)
Birth prevalence - [0, 24) wks, [500, 1000) g	Males	1155.815 (825.412 to 1506.837)	457.5 (352.552 to 573.483)
Birth prevalence - [0, 24) wks, [500, 1000) g	Females	1169.123 (802.003 to 1617.979)	515.406 (396.713 to 641.541)
Birth prevalence - [24, 26) wks, [500, 1000) g	Males	955.583 (723.748 to 1244.265)	443.357 (363.03 to 534.695)
Birth prevalence - [24, 26) wks, [500, 1000) g	Females	947.143 (702.662 to 1237.093)	487.549 (387.307 to 603.498)
Birth prevalence - [26, 28) wks, [500, 1000) g	Males	497.817 (377.617 to 648.547)	330.886 (261.438 to 401.709)
Birth prevalence - [26, 28) wks, [500, 1000) g	Females	483.682 (354.946 to 629.517)	344.618 (274.427 to 419.864)
Birth prevalence - [30, 32) wks, [500, 1000) g	Males	236.614 (163.821 to 324.502)	149.995 (117.866 to 188.368)
Birth prevalence - [30, 32) wks, [500, 1000) g	Females	229.197 (157.606 to 317.194)	152.117 (120.779 to 190.583)
Birth prevalence - [28, 30) wks, [500, 1000) g	Males	297.629 (214.953 to 396.586)	216.995 (173.321 to 271.466)
Birth prevalence - [28, 30) wks, [500, 1000) g	Females	281.056 (198.176 to 386.635)	219.884 (174.264 to 272.704)
Birth prevalence - [26, 28) wks, [1000, 1500) g	Males	267.91 (210.177 to 332.92)	164.167 (132.898 to 200.569)
Birth prevalence - [26, 28) wks, [1000, 1500) g	Females	266.509 (197.461 to 346.932)	174.222 (137.431 to 217.349)
Birth prevalence - [34, 36) wks, [1000, 1500) g	Males	142.056 (98.086 to 197.774)	52.86 (42.914 to 64.617)
Birth prevalence - [34, 36) wks, [1000, 1500) g	Females	141.899 (95.864 to 197.656)	57.421 (46.452 to 71.339)
Birth prevalence - [28, 30) wks, [1500, 2000) g	Males	127.966 (97.178 to 167.026)	50.018 (40.539 to 61.919)
Birth prevalence - [28, 30) wks, [1500, 2000) g	Females	130.924 (96.513 to 172.188)	57.275 (46.36 to 70.038)
Birth prevalence - [28, 30) wks, [1000, 1500) g	Males	158.563 (120.99 to 204.947)	103.32 (83.486 to 127.144)
Birth prevalence - [28, 30) wks, [1000, 1500) g	Females	153.905 (112.327 to 200.786)	107.529 (86.954 to 131.78)
Birth prevalence - [32, 34) wks, [1000, 1500) g	Males	117.142 (81.354 to 161.101)	53.185 (43.049 to 66.274)
Birth prevalence - [32, 34) wks, [1000, 1500) g	Females	115.171 (79.363 to 159.206)	56.034 (45.982 to 68.36)
Birth prevalence - [30, 32) wks, [1000, 1500) g	Males	119.308 (87.769 to 160.885)	67.163 (54.863 to 82.638)

Birth prevalence - [30, 32) wks, [1000, 1500) g	Females	115.448<br>(84.272 to 156.425)	69.14<br>(55.873 to 85.012)
Birth prevalence - [37, 38) wks, [1500, 2000) g	Males	62.972<br>(46.159 to 83.484)	24.148<br>(20.066 to 29.406)
Birth prevalence - [37, 38) wks, [1500, 2000) g	Females	59.988<br>(43.974 to 79.053)	26.719<br>(21.746 to 32.816)
Birth prevalence - [36, 37) wks, [1500, 2000) g	Males	60.218<br>(43.669 to 82.48)	23.031<br>(18.793 to 28.483)
Birth prevalence - [36, 37) wks, [1500, 2000) g	Females	58.527<br>(42.172 to 80.557)	25.143<br>(20.331 to 30.566)
Birth prevalence - [30, 32) wks, [2000, 2500) g	Males	67.971<br>(50.354 to 88.935)	18.03<br>(14.621 to 22.103)
Birth prevalence - [30, 32) wks, [2000, 2500) g	Females	69.383<br>(49.108 to 94.583)	22.069<br>(17.836 to 27.163)
Birth prevalence - [30, 32) wks, [1500, 2000) g	Males	77.369<br>(59.702 to 99.232)	31.079<br>(25.786 to 36.724)
Birth prevalence - [30, 32) wks, [1500, 2000) g	Females	76.134<br>(56.885 to 100.996)	34.756<br>(28.764 to 41.849)
Birth prevalence - [34, 36) wks, [1500, 2000) g	Males	55.555<br>(39.553 to 75.104)	21.346<br>(17.677 to 26.143)
Birth prevalence - [34, 36) wks, [1500, 2000) g	Females	54.335<br>(38.617 to 75.24)	23.046<br>(18.743 to 28.287)
Birth prevalence - [32, 34) wks, [1500, 2000) g	Males	57.155<br>(42.484 to 73.651)	23.114<br>(19.028 to 27.915)
Birth prevalence - [32, 34) wks, [1500, 2000) g	Females	56.101<br>(39.794 to 76.295)	25.149<br>(20.615 to 30.388)
Birth prevalence - [32, 34) wks, [2000, 2500) g	Males	37.444<br>(29.026 to 48.227)	12.233<br>(10.252 to 14.477)
Birth prevalence - [32, 34) wks, [2000, 2500) g	Females	36.874<br>(26.658 to 49.653)	14.384<br>(12.095 to 17.03)
Birth prevalence - [40, 42) wks, [2000, 2500) g	Males	18.092<br>(13.292 to 23.719)	9.23<br>(7.037 to 11.454)
Birth prevalence - [40, 42) wks, [2000, 2500) g	Females	15.574<br>(11.516 to 20.778)	9.975<br>(7.82 to 12.46)
Birth prevalence - [38, 40) wks, [2000, 2500) g	Males	13.104<br>(9.829 to 16.99)	8.198<br>(6.786 to 9.959)
Birth prevalence - [38, 40) wks, [2000, 2500) g	Females	11.308<br>(8.389 to 14.38)	8.577<br>(7.04 to 10.449)
Birth prevalence - [32, 34) wks, [2500, 3000) g	Males	33.063<br>(24.393 to 43.503)	8.441<br>(6.822 to 10.431)
Birth prevalence - [32, 34) wks, [2500, 3000) g	Females	32.812<br>(23.439 to 45.567)	10.398<br>(8.227 to 13.042)
Birth prevalence - [34, 36) wks, [2000, 2500) g	Males	21.925<br>(16.305 to 29.433)	9.367<br>(7.859 to 11.112)
Birth prevalence - [34, 36) wks, [2000, 2500) g	Females	21.297<br>(15.657 to 28.761)	10.295<br>(8.548 to 12.273)
Birth prevalence - [37, 38) wks, [2000, 2500) g	Males	13.0<br>(10.102 to 16.456)	8.096<br>(6.724 to 9.676)
Birth prevalence - [37, 38) wks, [2000, 2500) g	Females	11.563<br>(8.805 to 15.11)	8.467<br>(6.994 to 10.342)
Birth prevalence - [36, 37) wks, [2000, 2500) g	Males	14.401<br>(10.789 to 18.654)	8.221<br>(6.917 to 9.923)

Birth prevalence - [36, 37) wks, [2000, 2500) g	Females	13.513 (9.817 to 17.942)	8.654 (7.215 to 10.369)
Birth prevalence - [34, 36) wks, [2500, 3000) g	Males	13.419 (10.387 to 16.819)	5.562 (4.646 to 6.696)
Birth prevalence - [34, 36) wks, [2500, 3000) g	Females	13.266 (9.666 to 17.689)	6.395 (5.292 to 7.606)
Birth prevalence - [34, 36) wks, [4000, 4500) g	Males	23.096 (14.708 to 35.098)	2.895 (2.245 to 3.716)
Birth prevalence - [34, 36) wks, [4000, 4500) g	Females	25.038 (15.763 to 37.255)	3.778 (2.855 to 4.925)
Birth prevalence - [34, 36) wks, [3000, 3500) g	Males	14.006 (10.222 to 18.478)	4.322 (3.449 to 5.338)
Birth prevalence - [34, 36) wks, [3000, 3500) g	Females	14.375 (10.269 to 20.114)	5.265 (4.145 to 6.564)
Birth prevalence - [36, 37) wks, [2500, 3000) g	Males	4.874 (4.014 to 5.713)	3.699 (3.134 to 4.374)
Birth prevalence - [36, 37) wks, [2500, 3000) g	Females	4.609 (3.731 to 5.61)	3.898 (3.235 to 4.67)
Birth prevalence - [34, 36) wks, [3500, 4000) g	Males	18.024 (12.279 to 25.547)	3.657 (2.838 to 4.675)
Birth prevalence - [34, 36) wks, [3500, 4000) g	Females	19.263 (12.567 to 27.924)	4.634 (3.576 to 5.989)
Birth prevalence - [37, 38) wks, [2500, 3000) g	Males	3.306 (2.82 to 3.843)	3.194 (2.691 to 3.803)
Birth prevalence - [37, 38) wks, [2500, 3000) g	Females	2.991 (2.496 to 3.61)	3.242 (2.745 to 3.817)
Birth prevalence - [40, 42) wks, [2500, 3000) g	Males	3.771 (3.002 to 4.693)	3.175 (2.56 to 3.923)
Birth prevalence - [40, 42) wks, [2500, 3000) g	Females	3.244 (2.486 to 4.159)	3.228 (2.605 to 3.985)
Birth prevalence - [38, 40) wks, [2500, 3000) g	Males	2.755 (2.274 to 3.309)	2.944 (2.44 to 3.548)
Birth prevalence - [38, 40) wks, [2500, 3000) g	Females	2.376 (1.91 to 2.886)	2.938 (2.434 to 3.503)
Birth prevalence - [36, 37) wks, [3000, 3500) g	Males	3.774 (3.094 to 4.497)	2.466 (2.058 to 2.929)
Birth prevalence - [36, 37) wks, [3000, 3500) g	Females	3.73 (2.981 to 4.646)	2.715 (2.277 to 3.218)
Birth prevalence - [36, 37) wks, [4000, 4500) g	Males	6.826 (5.212 to 9.045)	1.77 (1.491 to 2.082)
Birth prevalence - [36, 37) wks, [4000, 4500) g	Females	7.269 (5.144 to 9.821)	2.177 (1.786 to 2.607)
Birth prevalence - [36, 37) wks, [3500, 4000) g	Males	4.544 (3.64 to 5.622)	2.057 (1.74 to 2.44)
Birth prevalence - [36, 37) wks, [3500, 4000) g	Females	4.662 (3.577 to 6.014)	2.398 (1.98 to 2.864)
Birth prevalence - [37, 38) wks, [3000, 3500) g	Males	2.007 (1.759 to 2.293)	1.888 (1.613 to 2.224)
Birth prevalence - [37, 38) wks, [3000, 3500) g	Females	1.925 (1.582 to 2.328)	1.972 (1.669 to 2.313)
Birth prevalence - [37, 38) wks, [4000, 4500) g	Males	3.28 (2.596 to 4.133)	1.335 (1.171 to 1.532)

Birth prevalence - [37, 38) wks, [4000, 4500) g	Females	3.521<br>(2.649 to 4.5)	1.559<br>(1.333 to 1.835)
Birth prevalence - [37, 38) wks, [3500, 4000) g	Males	2.128<br>(1.833 to 2.466)	1.505<br>(1.299 to 1.76)
Birth prevalence - [37, 38) wks, [3500, 4000) g	Females	2.142<br>(1.694 to 2.67)	1.661<br>(1.411 to 1.961)
Birth prevalence - [40, 42) wks, [3000, 3500) g	Males	1.436<br>(1.245 to 1.65)	1.47<br>(1.199 to 1.8)
Birth prevalence - [40, 42) wks, [3000, 3500) g	Females	1.326<br>(1.069 to 1.614)	1.465<br>(1.188 to 1.775)
Birth prevalence - [38, 40) wks, [3000, 3500) g	Males	1.33<br>(1.155 to 1.53)	1.559<br>(1.305 to 1.851)
Birth prevalence - [38, 40) wks, [3000, 3500) g	Females	1.224<br>(1.0 to 1.492)	1.564<br>(1.304 to 1.847)
Birth prevalence - [38, 40) wks, [4000, 4500) g	Males	1.787<br>(1.453 to 2.182)	1.175<br>(1.005 to 1.371)
Birth prevalence - [38, 40) wks, [4000, 4500) g	Females	1.877<br>(1.467 to 2.388)	1.224<br>(1.022 to 1.465)
Birth prevalence - [38, 40) wks, [3500, 4000) g	Males	1.785<br>(1.478 to 2.147)	1.173<br>(1.0 to 1.377)
Birth prevalence - [38, 40) wks, [3500, 4000) g	Females	1.892<br>(1.481 to 2.352)	1.23<br>(1.03 to 1.46)
Birth prevalence - [40, 42) wks, [3500, 4000) g	Males	1.0<br>(1.0 to 1.0)	1.003<br>(1.0 to 1.046)
Birth prevalence - [40, 42) wks, [3500, 4000) g	Females	1.002<br>(1.0 to 1.013)	1.001<br>(1.0 to 1.006)
Birth prevalence - [40, 42) wks, [4000, 4500) g	Males	1.0<br>(1.0 to 1.0)	1.0<br>(1.0 to 1.0)
Birth prevalence - [40, 42) wks, [4000, 4500) g	Females	1.0<br>(1.0 to 1.0)	1.0<br>(1.0 to 1.0)
Birth prevalence - [28, 30) wks, [2000, 2500) g	Males	117.172<br>(83.895 to 158.056)	27.726<br>(21.877 to 34.972)
Birth prevalence - [28, 30) wks, [2000, 2500) g	Females	121.682<br>(84.349 to 171.375)	33.983<br>(26.101 to 43.404)
Birth prevalence - [28, 30) wks, [2500, 3000) g	Males	77.948<br>(54.687 to 105.047)	16.608<br>(12.653 to 21.188)
Birth prevalence - [28, 30) wks, [2500, 3000) g	Females	79.193<br>(54.099 to 112.236)	20.387<br>(15.089 to 26.434)
Birth prevalence - [28, 30) wks, [3000, 3500) g	Males	42.199<br>(29.891 to 57.227)	10.082<br>(7.777 to 13.056)
Birth prevalence - [28, 30) wks, [3000, 3500) g	Females	42.551<br>(28.25 to 63.209)	11.989<br>(9.084 to 15.544)
Birth prevalence - [30, 32) wks, [2500, 3000) g	Males	58.722<br>(42.419 to 78.873)	12.115<br>(9.518 to 15.521)
Birth prevalence - [30, 32) wks, [2500, 3000) g	Females	59.522<br>(42.058 to 82.793)	15.364<br>(11.936 to 19.581)
Birth prevalence - [30, 32) wks, [3000, 3500) g	Males	45.67<br>(32.014 to 65.531)	8.381<br>(6.362 to 10.842)
Birth prevalence - [30, 32) wks, [3000, 3500) g	Females	46.104<br>(30.243 to 66.207)	10.506<br>(8.041 to 13.513)
Birth prevalence - [30, 32) wks, [3500, 4000) g	Males	36.334<br>(21.558 to 54.813)	5.698<br>(4.293 to 7.349)

Birth prevalence - [30, 32) wks, [3500, 4000) g	Females	37.931<br>(22.692 to 61.276)	6.892<br>(5.175 to 9.058)
Birth prevalence - [32, 34) wks, [3000, 3500) g	Males	34.016<br>(23.515 to 48.37)	6.577<br>(4.934 to 8.436)
Birth prevalence - [32, 34) wks, [3000, 3500) g	Females	34.585<br>(22.909 to 49.754)	8.314<br>(6.362 to 10.789)
Birth prevalence - [32, 34) wks, [3500, 4000) g	Males	36.248<br>(23.158 to 54.67)	5.068<br>(3.761 to 6.741)
Birth prevalence - [32, 34) wks, [3500, 4000) g	Females	38.098<br>(23.301 to 59.429)	6.476<br>(4.666 to 8.689)
Birth prevalence - [36, 37) wks, [1000, 1500) g	Males	166.686<br>(118.487 to 222.581)	57.535<br>(45.999 to 71.742)
Birth prevalence - [36, 37) wks, [1000, 1500) g	Females	169.725<br>(119.017 to 229.008)	63.564<br>(50.068 to 80.703)
Birth prevalence - [38, 40) wks, [1000, 1500) g	Males	174.066<br>(125.125 to 232.507)	57.966<br>(44.393 to 73.241)
Birth prevalence - [38, 40) wks, [1000, 1500) g	Females	171.557<br>(121.585 to 237.047)	65.208<br>(48.821 to 84.308)
Birth prevalence - [38, 40) wks, [1500, 2000) g	Males	67.302<br>(49.547 to 89.055)	25.206<br>(20.365 to 31.168)
Birth prevalence - [38, 40) wks, [1500, 2000) g	Females	62.19<br>(45.884 to 83.445)	28.05<br>(22.625 to 35.139)
Birth prevalence - [40, 42) wks, [1500, 2000) g	Males	76.673<br>(56.177 to 102.468)	25.785<br>(19.387 to 34.168)
Birth prevalence - [40, 42) wks, [1500, 2000) g	Females	70.411<br>(49.221 to 97.952)	29.113<br>(21.355 to 38.272)

Suboptimal Breastfeeding

Flowchart

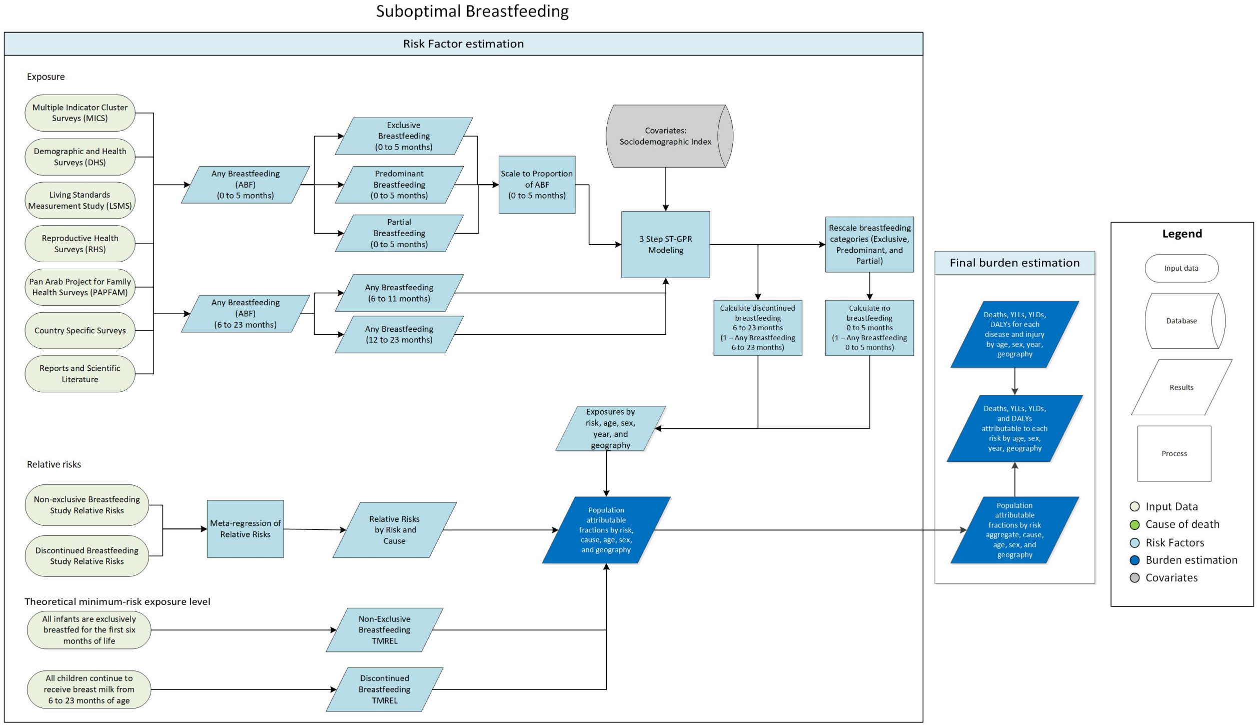

Definitions

Exposure to suboptimal breastfeeding is composed of 2 distinct categories: non-exclusive breastfeeding and discontinued breastfeeding.

Non-exclusive breastfeeding is defined as the proportion of children under 6 months of age who are not exclusively breastfed. We then parse those not exclusively breastfed into 3 categories – predominant, partial, and no breastfeeding. Exclusive breastfeeding is defined as the proportion of children who receive no other food or drink except breast milk (allowing for ORS, drops, or syrups containing vitamins, minerals, or medicines). Predominant breastfeeding is the proportion of children whose predominant source of nourishment is breastmilk but also receive other liquids. Partial breastfeeding refers to those infants who receive breastmilk as well as food and liquids, including non-human milk and formula. No breastfeeding refers to infants who do not receive breast milk as a source of nourishment.

Discontinued breastfeeding is defined as the proportion of children between 6 to 23 months who receive no breast milk as a source of nourishment.

Input Data

We made substantial exposure data updates for GBD 2017, including extracting identified surveys not included in previous rounds and re-extracting all surveys for new GBD 2017 subnational locations. We searched the Global Health Data Exchange

(GHDx) database for sources using the keyword "Breastfeeding." Of 2,026 potential sources identified, we extracted 1,081 unique country-years of data (2,262 unique geography-years, including subnational geographies) that met our inclusion criteria. The data used in the analysis consists mostly of processed individual-level microdata from surveys; in the cases where microdata was unavailable, we used reported tabulated data from survey reports and scientific literature. Data used to categorize type of non-exclusive breastfeeding (predominant, partial, and none) come from surveys with 24-hour dietary logs based on maternal recall.

Exposure Modelling

Using the processed microdata and tabulated data from reports, we generated a complete time series from 1980 to 2017 for the prevalence of breastfeeding patterns for children 0 to 5 months and 6 to 23 months using a three-step spatio-temporal Gaussian process regression modelling process.

First, we estimated a robust linear regression using each geography's sociodemographic index as a covariate. The following linear model was used for the estimation of breastfeeding indicators:

$$logit(P_{x,c,t}) = \beta_0 + \beta_1 SDI_{\mathrm{c,t}} + \alpha_c + \gamma_{R[c]} + \omega_{\mathrm{SR[c]}} + \varepsilon_{c,t}$$

where $P_{x,c,t}$ is prevalence for breastfeeding category x in country c and year t; $SDI_{c,t}$ is value of the Sociodemographic Index for country c and year t; α_c, $\gamma_{R[c]}$, and $\omega_{\mathrm{SR[c]}}$ are country, region, and super-region random intercepts, respectively.

We then followed this with a spatio-temporal regression that uses the residuals of the predictions from the linear regression to perform a locally-weighted regression that provides a greater weighting factor to those nearer in space and time. The predicted residuals from this step are then added to those created in the linear regression step.

Finally, we run a Gaussian process regression that incorporates the variance of the input data as well as the variance of the model predictions. It uses predictions from the spatio-temporal regression as the mean function and generates draws from a multinomial distribution (based on the data uncertainty in the prior) to generate the final prevalence estimates and their confidence intervals.

We estimated six models to produce each of our categories: the proportion of currently breastfeeding infants 0-5 months of age, the ratio of infants exclusively breastfed to breastfed infants 0-5 months of age, the ratio of infants predominantly breastfed to breastfed infants 0-5 months of age, the ratio of infants partially breastfed to breastfed infants 0-5 months of age, the proportion of currently breastfeeding infants 6-11 months of age, and the proportion of currently breastfeeding infants 12-23 months of age. We convert the ratios of exclusive, predominant, and partial breastfeeding to the total category prevalence proportions by multiplying each ratio by the estimates of any

breastfeeding among infants aged 0-5 months. This ensures that these categories sum correctly to the "any breastfeeding 0-5 months" envelope. We calculate the proportion of infants receiving no breastmilk 0-5 months of age by subtracting the estimates of current breastfeeding from 1. We perform the same operation to estimate discontinued breastfeeding in the 6-11 months and 12-23 months categories.

Estimating Attributable Burden

Assessment of risk-outcome pairs

We included outcomes based on the strength of available evidence supporting a causal relationship. Studies evaluating the causal evidence for our risk-outcome pairs came primarily from articles found in a review published by the World Health Organization.[1] Non-exclusive breastfeeding was paired with diarrhea and lower-respiratory infection as diseases outcomes. Discontinued breastfeeding was paired with diarrhea as an outcome.

Theoretical minimum-risk exposure level

For non-exclusive breastfeeding, those children that received no source of nourishment other than breastmilk ("exclusively breastfed") were considered to be at the lowest risk of any of the disease outcomes. For discontinued breastfeeding, we assumed that children aged 6 to 23 months who received any breastmilk as a source of nourishment to be at the lowest risk of disease outcome.

Relative Risks

We estimate relative risks for non-exclusive breastfeeding in a meta-analysis using relative risks from studies compiled in a published review by the World Health Organization.[41]

**Breastfeeding category**	**Relative risk**
None	1.739<br>(1.493 to 2.025)
Partial	1.483<br>(1.206 to 1.792)
Predominant	1.369<br>(1.055 to 1.8)
Exclusive	1.0<br>(1.0 to 1.0)

References

1 Foreman KJ, Lozano R, Lopez AD, Murray CJ. Modeling causes of death: an integrated approach using CODEm. *Popul Health Metr* 2012; **10**: 1.

2 GBD 2017 Causes of Death Collaborators. Global, regional, and national age-sex-specific mortality for 282 causes of death in 195 countries and territories, 1980-2017: a systematic analysis for the Global Burden of Disease Study 2017. *The Lancet* 2018; **392**: 1736–88.

3 GBD 2016 Mortality Collaborators. Global, regional, and national under-5 mortality, adult mortality, age-specific mortality, and life expectancy, 1970-2016: a systematic analysis for the Global Burden of Disease Study 2016. *Lancet Lond Engl* 2017; **390**: 1084–150.

4 Foreman KJ, Lozano R, Lopez AD, Murray CJ. Modeling causes of death: an integrated approach using CODEm. *Popul Health Metr* 2012; **10**: 1.

5 Walker CLF, Rudan I, Liu L, *et al.* Global burden of childhood pneumonia and diarrhoea. *The Lancet* 2013; **381**: 1405–16.

6 Rudan I, O'Brien KL, Nair H, *et al.* Epidemiology and etiology of childhood pneumonia in 2010: estimates of incidence, severe morbidity, mortality, underlying risk factors and causative pathogens for 192 countries. *J Glob Health* 2013; **3**. DOI:10.7189/jogh.03.010401.

7 WHO | WHO Global Database on Child Growth and Malnutrition. WHO. http://www.who.int/nutgrowthdb/en/ (accessed July 30, 2018).

8 Wang Y, Chen H-J. Use of Percentiles and Z-Scores in Anthropometry. In: Preedy VR, ed. Handbook of Anthropometry. New York, NY: Springer New York, 2012: 29–48.

9 Uribe Á, Cecilia M, López Gaviria A, Estrada Restrepo A. Concordance between Z scores from WHO 2006 and the NCHS 1978 growth standards of children younger than five. Antioquia-Colombia. *Perspect En Nutr Humana* 2008; **10**: 177–87.

10 McDonald CM, Olofin I, Flaxman S, *et al.* The effect of multiple anthropometric deficits on child mortality: meta-analysis of individual data in 10 prospective studies from developing countries. *Am J Clin Nutr* 2013; **97**: 896–901.

11 Olofin I, McDonald CM, Ezzati M, *et al.* Associations of Suboptimal Growth with All-Cause and Cause-Specific Mortality in Children under Five Years: A Pooled Analysis of Ten Prospective Studies. *PLOS ONE* 2013; **8**: e64636.

12 van Donkelaar A, Martin RV, Brauer M, *et al.* Global Estimates of Fine Particulate Matter using a Combined Geophysical-Statistical Method with Information from Satellites, Models, and Monitors. *Environ Sci Technol* 2016; **50**: 3762–72.

13 Bowe B, Xie Y, Li T, Yan Y, Xian H, Al-Aly Z. The 2016 global and national burden of diabetes mellitus attributable to PM2·5 air pollution. *Lancet Planet Health* 2018; **2**: e301–12.

14 Turner MC, Jerrett M, Pope CA, *et al.* Long-Term Ozone Exposure and Mortality in a Large Prospective Study. *Am J Respir Crit Care Med* 2016; **193**: 1134–42.

15 Burnett RT, Pope CA, Ezzati M, *et al.* An integrated risk function for estimating the global burden of disease attributable to ambient fine particulate matter exposure. *Environ Health Perspect* 2014; **122**: 397–403.

16 Cohen AJ, Brauer M, Burnett R, *et al.* Estimates and 25-year trends of the global burden of disease attributable to ambient air pollution: an analysis of data from the Global Burden of Diseases Study 2015. *Lancet Lond Engl* 2017; **389**: 1907–18.

17 Rabie T, Curtis V. Handwashing and risk of respiratory infections: a quantitative systematic review. *Trop Med Int Health TM IH* 2006; **11**: 258–67.

18 The DHS Program - Quality information to plan, monitor and improve population, health, and nutrition programs. https://dhsprogram.com/ (accessed Oct 25, 2018).

19 UNICEF Multiple Indicator Cluster Survey (MICS). http://mics.unicef.org/ (accessed Oct 25, 2018).

20 Centers for Disease Control and Prevention. Reproductive Health Surveys. 2018; published online April 11. https://www.cdc.gov/reproductivehealth/global/tools/surveys.htm (accessed Oct 25, 2018).

21 World Bank. Living Standards Measurement Study (LSMS). http://surveys.worldbank.org/lsms (accessed Oct 25, 2018).

22 Theodoratou E, Johnson S, Jhass A, *et al.* The effect of Haemophilus influenzae type b and pneumococcal conjugate vaccines on childhood pneumonia incidence, severe morbidity and mortality. *Int J Epidemiol* 2010; **39 Suppl 1**: i172-185.

23 Measure DHS: Demographic and Health Surveys. http://www.measuredhs.com (accessed Aug 11, 2015).

24 UNICEF Stat. Monit. Multiple Indicator Cluster Survey (MICS). http://www.unicef.org/statistics/index_24302.html (accessed Aug 11, 2015).

25 Cent. Dis. Control Prev. Reproductive Health Surveys (RHS). http://www.cdc.gov/reproductivehealth/Global/surveys.htm (accessed Aug 11, 2015).

26 World Bank. Living Standard Measruement Studies (LSMS). http://go.worldbank.org/UK1ETMHBN0 (accessed Aug 11, 2015).

27 WHO Multi-Ctry. Stud. Data Arch. World Health Survey (WHS). http://apps.who.int/healthinfo/systems/surveydata/index.php/catalog/whs/about (accessed Aug 11, 2015).

28 IHME GHDx. Global Helath Data Exchange. http://ghdx.healthdata.org/ (accessed Aug 11, 2015).

29 WHO | WHO/UNICEF Joint Reporting Process. WHO. http://www.who.int/immunization/monitoring_surveillance/routine/reporting/reporting/en/ (accessed Aug 17, 2015).

30 Prospective Studies Collaboration. Age-specific relevance of usual blood pressure to vascular mortality: a meta-analysis of individual data for one million adults in 61 prospective studies. *The Lancet* 2002; **360**: 1903–13.

31 Ng M, Freeman MK, Fleming TD, *et al.* Smoking Prevalence and Cigarette Consumption in 187 Countries, 1980-2012. *JAMA* 2014; **311**: 183.

32 Ng M, Fleming T, Robinson M, *et al.* Global, regional, and national prevalence of overweight and obesity in children and adults during 1980–2013: a systematic analysis for the Global Burden of Disease Study 2013. *The Lancet* 2014; **384**: 766–81.

33 Murray CJ, Shengelia B, Gupta N, Moussavi S, Tandon A, Thieren M. Validity of reported vaccination coverage in 45 countries. *The Lancet* 2003; **362**: 1022–1027.

34 Haddad S, Bicaba A, Feletto M, Fournier P, Zunzunegui MV. Heterogeneity in the validity of administrative-based estimates of immunization coverage across health districts in Burkina Faso: implications for measurement, monitoring and planning. *Health Policy Plan* 2010; **25**: 393–405.

35 Wang H, Liddell CA, Coates MM, *et al.* Global, regional, and national levels of neonatal, infant, and under-5 mortality during 1990–2013: a systematic analysis for the Global Burden of Disease Study 2013. *The Lancet* 13; **384**: 957–79.

36 Barber RM, Fullman N, Sorensen RJD, *et al.* Healthcare Access and Quality Index based on mortality from causes amenable to personal health care in 195 countries and territories, 1990?2015: a novel analysis from the Global Burden of Disease Study 2015. *The Lancet* 2017; published online May. DOI:10.1016/S0140-6736(17)30818-8.

37 Foreman KJ, Lozano R, Lopez AD, Murray CJ. Modeling causes of death: an integrated approach using CODEm. *Popul Health Metr* 2012; **10**: 1.

38 WHO | Data, statistics and graphics. WHO. http://www.who.int/immunization/monitoring_surveillance/data/en/ (accessed June 12, 2017).

39 GBD 2016 Lower respiratory infection collaborators. Estimates of the global, regional, and national morbidity, mortality, and aetiologies of diarrhoea in 195 countries: a systematic analysis for the Global Burden of Disease Study 2016. *Lancet Infect Dis* 2018.

40 Katz J, Lee AC, Kozuki N, *et al.* Mortality risk in preterm and small-for-gestational-age infants in low-income and middle-income countries: a pooled country analysis. *Lancet Lond Engl* 2013; **382**: 417–25.

41 Horta BL, Victora CG. Short-term effects of breastfeeding: a systematic review on the benefits of breastfeeding on diarrhoea and pneumonia mortality. *World Health Organ* 2013.

Supplementary Results

Supplementary Results Table 1. Estimates of the deaths, mortality rate, incidence, percent change in mortality rate and in incidence, case fatality, and attributable fractions for all risk factors and for aggregate prevention and protection risk factors for each country and GBD region and super region are shown.

Location	Deaths (95% UI)	Mortality rate per 100,000 (95% UI)	Percent change mortality rate 1990-2017 (95% UI)	Incidence per 100,000 (95% UI)	Percent change incidence 1990 to 2017 (95% UI)	Case fatality ratio (95% UI)	Attributable fraction for all risks (95% UI)	Attributable fraction for prevention-associated risks (95% UI)	Attributable fraction for protection-associated risks (95% UI)
Global	808,920 (747,286-873,591)	118.8 (109.8-128.3)	-67.2% (-70.2 to -63.6%)	12,197.8 (9,762.1-14,908.7)	-32.4% (-37.5 to -27.2%)	1.0% (0.9-1.1%)	93.4% (90.3 to 95.7%)	65.2% (50.2 to 77.6%)	82.0% (62.6 to 92.1%)
Southeast Asia, East Asia, and Oceania	63,661 (58,190-69,821)	45.0 (41.1-49.3)	-85.7% (-87.2 to -83.7%)	13,383.7 (10,686.7-16,401.7)	-38.8% (-44.3 to -32.3%)	0.3% (0.3-0.4%)	88.7% (83.6 to 92.8%)	61.2% (45.3 to 75.0%)	78.8% (56.2 to 90.8%)
East Asia	22,824 (20,743-25,438)	27.1 (24.6-30.2)	-90.7% (-91.9 to -89.2%)	9,376.4 (7,387.3-11,625.0)	-54.8% (-59.6 to -49.2%)	0.3% (0.3-0.3%)	83.2% (76.5 to 88.9%)	61.5% (41.9 to 77.8%)	70.9% (46.3 to 85.7%)
China	21,173 (19,193-23,750)	26.3 (23.9-29.5)	-91.2% (-92.3 to -89.7%)	9,231.5 (7,279.8-11,420.9)	-56.0% (-60.6 to -50.5%)	0.3% (0.3-0.3%)	82.8% (76.0 to 88.7%)	61.3% (41.2 to 78.0%)	70.4% (45.7 to 85.3%)
North Korea	1,232 (888-1,678)	91.8 (66.1-125.0)	-20.7% (-42.6 to 9.3%)	17,274.4 (13,741.4-21,672.0)	5.1% (-7.9 to 19.0%)	0.5% (0.5-0.6%)	90.7% (86.3 to 94.0%)	68.3% (55.6 to 79.0%)	79.8% (55.2 to 92.3%)
Taiwan	51 (42-63)	5.0 (4.1-6.0)	-63.6% (-70.9 to -55.1%)	10,393.3 (8,028.4-13,129.9)	11.1% (-4.1 to 29.4%)	<0.1% (<0.1-0.1%)	73.4% (61.0 to 83.5%)	36.4% (21.6 to 53.1%)	67.4% (38.5 to 84.8%)

Location	Deaths (95% UI)	Mortality rate per 100,000 (95% UI)	Percent change mortality rate 1990-2017 (95% UI)	Incidence per 100,000 (95% UI)	Percent change incidence 1990 to 2017 (95% UI)	Case fatality ratio (95% UI)	Attributable fraction for all risks (95% UI)	Attributable fraction for prevention-associated risks (95% UI)	Attributable fraction for protection-associated risks (95% UI)
Southeast Asia	39,066 (34,532-44,291)	70.2 (62.1-79.6)	-80.7% (-83.5 to -77.1%)	19,344.3 (15,535.4-23,655.9)	-20.7% (-27.5 to -13.0%)	0.4% (0.3-0.4%)	91.7% (87.5 to 94.8%)	61.2% (46.0 to 74.3%)	82.6% (61.1 to 93.1%)
Cambodia	3,039 (2,342-4,002)	169.6 (130.8-223.4)	-81.9% (-86.6 to -75.2%)	20,672.9 (16,726.4-25,205.1)	-44.7% (-52.3 to -36.5%)	0.8% (0.8-0.9%)	93.7% (90.3 to 95.9%)	58.2% (41.6 to 73.6%)	81.7% (60.4 to 92.4%)
Indonesia	10,895 (9,656-12,370)	51.9 (46.0-58.9)	-86.1% (-88.3 to -83.4%)	20,588.9 (16,365.0-25,316.8)	2.0% (-7.5 to 12.3%)	0.3% (0.2-0.3%)	93.3% (89.8 to 95.7%)	64.3% (48.7 to 77.1%)	84.8% (67.4 to 93.7%)
Laos	2,419 (1,756-3,304)	300.5 (218.1-410.4)	-75.9% (-82.4 to -68.0%)	14,937.9 (11,948.2-18,517.6)	-55.2% (-60.0 to -49.6%)	2.0% (1.8-2.2%)	92.0% (87.9 to 95.1%)	63.6% (43.8 to 79.1%)	82.6% (55.2 to 95.0%)
Malaysia	234 (162-321)	9.0 (6.2-12.4)	-77.0% (-85.4 to -64.4%)	15,422.2 (11,619.5-19,910.9)	-3.0% (-16.3 to 13.6%)	0.1% (0.1-0.1%)	88.4% (81.4 to 93.0%)	53.4% (37.9 to 67.0%)	81.5% (60.1 to 92.8%)
Maldives	2 (1-3)	5.3 (3.9-7.1)	-94.5% (-96.4 to -91.9%)	14,226.3 (10,743.1-18,163.8)	-28.7% (-38.6 to -17.2%)	<0.1% (<0.1-<0.1%)	87.6% (80.0 to 92.6%)	53.4% (41.1 to 66.9%)	80.9% (58.8 to 92.5%)
Mauritius	10 (8-14)	16.2 (12.7-21.1)	-53.7% (-64.9 to -38.6%)	13,571.0 (10,562.7-17,167.1)	-6.2% (-18.1 to 7.4%)	0.1% (0.1-0.1%)	88.2% (80.2 to 93.3%)	43.7% (23.5 to 63.7%)	82.3% (62.3 to 92.4%)
Myanmar	6,727 (5,063-8,807)	152.6 (114.9-199.8)	-80.4% (-85.8 to -73.5%)	14,434.9 (11,573.7-17,777.8)	-46.8% (-52.4 to -40.3%)	1.1% (1.0-1.1%)	91.8% (87.3 to 95.1%)	64.9% (46.0 to 79.9%)	80.9% (57.5 to 92.5%)
Philippines	12,396 (9,115-16,482)	103.8 (76.3-138.0)	-74.7% (-81.5 to -64.7%)	22,386.8 (18,354.3-26,797.9)	-42.3% (-50.9 to -32.8%)	0.5% (0.4-0.5%)	90.5% (85.3 to 94.3%)	56.6% (37.7 to 73.1%)	83.0% (57.1 to 94.6%)

Location	Deaths (95% UI)	Mortality rate per 100,000 (95% UI)	Percent change mortality rate 1990-2017 (95% UI)	Incidence per 100,000 (95% UI)	Percent change incidence 1990 to 2017 (95% UI)	Case fatality ratio (95% UI)	Attributable fraction for all risks (95% UI)	Attributable fraction for prevention-associated risks (95% UI)	Attributable fraction for protection-associated risks (95% UI)
Sri Lanka	117 (79-171)	7.3 (4.9-10.6)	-83.2% (-89.6 to -73.6%)	15,783.5 (11,988.7-20,228.8)	-7.8% (-20.6 to 7.4%)	<0.1% (<0.1-0.1%)	89.9% (82.6 to 94.4%)	56.5% (42.6 to 71.1%)	83.0% (63.3 to 92.9%)
Seychelles	3 (3-4)	40.9 (32.8-50.1)	-18.1% (-39.7 to 8.7%)	17,440.0 (13,831.9-21,502.5)	2.3% (-8.8 to 14.1%)	0.2% (0.2-0.2%)	83.7% (74.1 to 90.5%)	44.7% (23.6 to 65.0%)	76.8% (53.4 to 89.2%)
Thailand	507 (371-650)	15.0 (11.0-19.2)	-83.2% (-87.9 to -77.2%)	10,554.3 (8,307.9-13,118.5)	-34.7% (-41.5 to -27.1%)	0.1% (0.1-0.1%)	86.3% (78.3 to 91.8%)	68.2% (44.3 to 84.3%)	77.8% (52.8 to 90.7%)
Timor-Leste	243 (161-322)	139.7 (92.6-184.7)	-84.9% (-89.8 to -78.9%)	20,676.0 (16,099.9-26,261.7)	-46.8% (-53.5 to -39.5%)	0.7% (0.6-0.7%)	95.9% (93.2 to 97.7%)	68.6% (54.0 to 81.2%)	90.2% (71.7 to 97.5%)
Vietnam	2,423 (1,771-3,235)	31.3 (22.9-41.8)	-82.5% (-87.8 to -74.8%)	20,164.5 (16,075.4-25,012.6)	-14.0% (-24.6 to -2.7%)	0.2% (0.1-0.2%)	89.3% (83.5 to 93.2%)	64.6% (48.6 to 77.8%)	79.1% (55.5 to 91.2%)
Oceania	1,770 (1,295-2,325)	99.5 (72.8-130.7)	-48.1% (-63.1 to -26.9%)	16,573.7 (13,249.1-20,596.4)	-12.5% (-22.0 to -1.8%)	0.6% (0.5-0.6%)	93.1% (89.9 to 95.6%)	68.3% (50.0 to 82.7%)	85.2% (64.2 to 94.7%)
American Samoa	1 (1-2)	23.8 (16.5-33.3)	-47.1% (-64.4 to -19.8%)	13,047.5 (10,074.1-16,548.6)	21.9% (5.2 to 37.9%)	0.2% (0.2-0.2%)	79.0% (67.5 to 87.3%)	61.6% (36.6 to 80.0%)	75.1% (48.3 to 89.0%)
Federated States of Micronesia	4 (3-6)	40.7 (25.2-56.3)	-73.8% (-84.2 to -60.5%)	13,742.9 (10,648.4-17,509.7)	-11.3% (-23.6 to 1.9%)	0.3% (0.2-0.3%)	84.3% (75.3 to 90.5%)	51.5% (27.1 to 72.8%)	76.9% (51.2 to 90.6%)
Fiji	62 (42-87)	65.9 (45.3-93.5)	-30.5% (-55.3 to 8.7%)	14,474.8 (11,530.5-18,113.5)	8.6% (-2.5 to 21.5%)	0.5% (0.4-0.5%)	83.3% (72.9 to 90.5%)	36.4% (18.7 to 57.0%)	77.8% (54.5 to 89.7%)

Location	Deaths (95% UI)	Mortality rate per 100,000 (95% UI)	Percent change mortality rate 1990-2017 (95% UI)	Incidence per 100,000 (95% UI)	Percent change incidence 1990 to 2017 (95% UI)	Case fatality ratio (95% UI)	Attributable fraction for all risks (95% UI)	Attributable fraction for prevention-associated risks (95% UI)	Attributable fraction for protection-associated risks (95% UI)
Guam	6 (4-7)	35.2 (26.7-45.4)	-5.9% (-32.1 to 27.2%)	11,973.7 (9,439.0-14,824.5)	31.0% (17.1 to 45.5%)	0.3% (0.3-0.3%)	76.5% (64.4 to 85.6%)	59.8% (34.7 to 78.4%)	73.6% (47.5 to 87.9%)
Kiribati	10 (7-14)	58.6 (40.3-82.0)	-70.3% (-80.7 to -55.6%)	13,204.8 (10,353.3-16,466.6)	-16.5% (-26.2 to -5.6%)	0.4% (0.4-0.5%)	91.9% (87.1 to 95.2%)	63.6% (44.1 to 79.9%)	83.0% (59.8 to 93.9%)
Marshall Islands	4 (3-5)	60.4 (42.8-83.3)	-46.2% (-61.8 to -24.0%)	13,867.8 (10,904.6-17,313.2)	-1.5% (-12.9 to 11.4%)	0.4% (0.4-0.5%)	84.5% (76.0 to 90.4%)	53.2% (29.9 to 73.1%)	77.2% (52.5 to 90.5%)
Northern Mariana Islands	0 (0-0)	13.4 (9.8-17.9)	-42.0% (-58.6 to -16.0%)	12,353.8 (9,695.5-15,383.0)	27.6% (14.4 to 40.4%)	0.1% (0.1-0.1%)	77.4% (66.2 to 85.7%)	60.3% (36.4 to 78.9%)	72.9% (48.2 to 86.9%)
Papua New Guinea	1,466 (1,027-1,995)	108.2 (75.8-147.2)	-52.1% (-67.8 to -29.1%)	17,141.9 (13,714.3-21,352.0)	-19.0% (-28.7 to -7.9%)	0.6% (0.6-0.7%)	94.0% (91.2 to 96.2%)	70.0% (51.0 to 84.3%)	86.1% (65.5 to 95.4%)
Samoa	8 (6-11)	29.8 (21.3-40.3)	-49.7% (-65.1 to -27.9%)	12,920.8 (10,118.0-16,089.0)	1.4% (-10.1 to 14.5%)	0.2% (0.2-0.3%)	83.4% (76.0 to 89.7%)	67.6% (51.4 to 81.7%)	72.0% (45.7 to 86.8%)
Solomon Islands	67 (47-92)	71.3 (49.7-97.9)	-60.0% (-72.4 to -42.5%)	15,193.0 (11,985.6-18,833.3)	-11.3% (-21.8 to -0.8%)	0.5% (0.4-0.5%)	89.4% (83.8 to 93.4%)	59.9% (41.8 to 77.3%)	78.0% (51.9 to 91.6%)
Tonga	4 (3-5)	35.3 (25.3-48.0)	-44.5% (-60.2 to -20.2%)	12,841.8 (10,210.2-15,943.5)	3.5% (-6.9 to 16.7%)	0.3% (0.2-0.3%)	84.1% (74.4 to 90.5%)	55.2% (41.9 to 69.7%)	77.0% (52.9 to 89.3%)
Vanuatu	40 (28-56)	98.8 (69.6-137.6)	-21.8% (-47.4 to 20.5%)	14,293.5 (11,346.1-17,932.9)	11.7% (-0.1 to 24.4%)	0.7% (0.6-0.8%)	89.7% (84.4 to 93.5%)	69.5% (53.0 to 82.8%)	79.2% (54.0 to 91.5%)

Location	Deaths (95% UI)	Mortality rate per 100,000 (95% UI)	Percent change mortality rate 1990-2017 (95% UI)	Incidence per 100,000 (95% UI)	Percent change incidence 1990 to 2017 (95% UI)	Case fatality ratio (95% UI)	Attributable fraction for all risks (95% UI)	Attributable fraction for prevention-associated risks (95% UI)	Attributable fraction for protection-associated risks (95% UI)
Central Europe, Eastern Europe, and Central Asia	16,040 (14,296-18,051)	57.3 (51.0-64.4)	-66.8% (-70.7 to -62.3%)	9,219.6 (7,103.3-11,586.8)	-36.3% (-43.2 to -28.6%)	0.6% (0.6-0.7%)	84.5% (77.5 to 89.7%)	47.8% (33.5 to 62.2%)	75.8% (53.6 to 88.4%)
Central Asia	13,937 (12,246-15,922)	145.4 (127.7-166.1)	-69.9% (-73.8 to -65.2%)	6,206.9 (5,003.0-7,601.2)	-46.9% (-53.8 to -39.2%)	2.3% (2.2-2.6%)	85.1% (78.1 to 90.1%)	47.5% (32.6 to 62.2%)	77.2% (54.6 to 89.5%)
Armenia	73 (62-86)	35.2 (29.6-41.5)	-85.5% (-88.3 to -81.7%)	4,724.4 (3,797.5-5,871.8)	-39.4% (-47.9 to -30.1%)	0.7% (0.7-0.8%)	83.0% (74.8 to 89.5%)	48.6% (31.2 to 65.5%)	73.9% (48.2 to 88.2%)
Azerbaijan	1,931 (1,470-2,459)	227.1 (172.9-289.2)	-71.2% (-78.7 to -61.9%)	7,130.8 (5,711.2-8,745.2)	-43.2% (-51.5 to -34.2%)	3.2% (3.0-3.3%)	83.3% (75.6 to 89.5%)	46.3% (25.8 to 66.0%)	74.8% (51.2 to 87.7%)
Georgia	41 (31-57)	16.0 (11.9-22.2)	-93.9% (-95.5 to -91.2%)	4,707.3 (3,609.8-5,886.6)	-44.8% (-53.4 to -33.5%)	0.3% (0.3-0.4%)	80.2% (71.2 to 87.7%)	53.7% (35.6 to 71.2%)	68.4% (39.7 to 85.3%)
Kazakhstan	664 (488-890)	35.2 (25.9-47.3)	-84.7% (-88.8 to -79.2%)	4,799.9 (3,777.2-6,066.8)	-46.9% (-55.2 to -35.9%)	0.7% (0.7-0.8%)	78.9% (69.1 to 86.9%)	45.0% (23.4 to 67.3%)	72.0% (46.3 to 87.2%)
Kyrgyzstan	506 (426-592)	65.3 (55.0-76.5)	-87.1% (-89.5 to -84.1%)	6,130.4 (4,975.2-7,670.2)	-54.3% (-61.1 to -46.1%)	1.1% (1.0-1.1%)	76.6% (66.8 to 84.9%)	45.9% (24.3 to 68.2%)	69.2% (38.6 to 87.1%)
Mongolia	458 (357-593)	119.7 (93.3-155.0)	-87.5% (-90.5 to -83.5%)	6,579.8 (5,181.8-8,177.3)	-56.6% (-63.9 to -48.9%)	1.8% (1.8-1.9%)	80.6% (72.5 to 87.0%)	47.5% (25.2 to 66.5%)	66.5% (38.9 to 84.0%)

Location	Deaths (95% UI)	Mortality rate per 100,000 (95% UI)	Percent change mortality rate 1990-2017 (95% UI)	Incidence per 100,000 (95% UI)	Percent change incidence 1990 to 2017 (95% UI)	Case fatality ratio (95% UI)	Attributable fraction for all risks (95% UI)	Attributable fraction for prevention-associated risks (95% UI)	Attributable fraction for protection-associated risks (95% UI)
Tajikistan	3,550 (2,829-4,460)	284.7 (226.8-357.6)	-59.8% (-69.2 to -48.1%)	6,750.2 (5,408.0-8,249.0)	-54.0% (-60.7 to -44.8%)	4.2% (4.2-4.3%)	93.5% (89.8 to 95.9%)	63.4% (46.8 to 78.7%)	85.7% (67.3 to 94.5%)
Turkmenistan	786 (563-1,091)	142.4 (102.0-197.8)	-77.3% (-84.2 to -68.3%)	6,053.2 (4,806.2-7,467.7)	-58.0% (-64.5 to -50.9%)	2.4% (2.1-2.6%)	78.0% (66.6 to 86.4%)	56.4% (38.8 to 71.9%)	71.5% (44.7 to 86.7%)
Uzbekistan	5,928 (4,797-7,483)	172.7 (139.8-218.0)	-62.9% (-70.7 to -52.4%)	6,755.6 (5,383.2-8,307.9)	-43.3% (-52.2 to -34.1%)	2.6% (2.6-2.6%)	83.4% (75.1 to 89.8%)	36.7% (16.3 to 58.2%)	75.2% (50.9 to 88.7%)
Central Europe	707 (632-795)	12.5 (11.2-14.1)	-84.2% (-86.1 to -82.1%)	8,700.5 (6,862.8-10,868.4)	-26.9% (-34.5 to -18.2%)	0.1% (0.1-0.2%)	80.5% (71.7 to 87.6%)	53.0% (39.9 to 66.1%)	71.4% (47.0 to 85.5%)
Albania	66 (42-99)	37.9 (24.1-56.9)	-88.9% (-93.2 to -82.6%)	11,153.1 (8,686.3-14,025.3)	-55.9% (-62.8 to -48.2%)	0.3% (0.3-0.4%)	88.5% (82.3 to 92.6%)	39.0% (26.3 to 53.1%)	78.8% (54.2 to 91.3%)
Bosnia and Herzegovina	6 (4-8)	3.9 (2.8-5.2)	-68.5% (-80.2 to -49.2%)	10,795.0 (8,352.2-13,799.0)	8.5% (-4.2 to 23.0%)	<0.1% (<0.1-<0.1%)	84.9% (79.7 to 89.5%)	67.3% (50.9 to 80.7%)	71.9% (52.5 to 84.7%)
Bulgaria	62 (46-80)	19.3 (14.4-24.9)	-77.9% (-83.6 to -70.8%)	8,855.9 (6,866.2-11,051.3)	-41.7% (-50.5 to -31.0%)	0.2% (0.2-0.2%)	80.5% (70.9 to 88.3%)	42.5% (26.2 to 60.1%)	70.1% (43.8 to 85.9%)
Croatia	3 (3-4)	1.8 (1.4-2.3)	-83.0% (-87.1 to -77.7%)	6,120.2 (4,848.5-7,765.6)	-12.0% (-23.4 to 1.4%)	<0.1% (<0.1-<0.1%)	79.1% (69.7 to 86.6%)	57.4% (44.2 to 70.2%)	68.7% (45.0 to 83.5%)
Czech Republic	15 (11-19)	2.7 (2.0-3.5)	-79.6% (-84.9 to -72.9%)	6,908.5 (5,259.2-8,721.3)	6.0% (-5.5 to 19.3%)	<0.1% (<0.1-<0.1%)	79.3% (68.9 to 87.3%)	55.1% (43.4 to 67.5%)	69.8% (47.2 to 83.5%)

Location	Deaths (95% UI)	Mortality rate per 100,000 (95% UI)	Percent change mortality rate 1990-2017 (95% UI)	Incidence per 100,000 (95% UI)	Percent change incidence 1990 to 2017 (95% UI)	Case fatality ratio (95% UI)	Attributable fraction for all risks (95% UI)	Attributable fraction for prevention-associated risks (95% UI)	Attributable fraction for protection-associated risks (95% UI)
Hungary	21 (15-28)	4.7 (3.4-6.3)	-80.9% (-86.5 to -73.8%)	11,123.4 (8,379.8-14,359.9)	32.7% (14.4 to 55.5%)	<0.1% (<0.1-<0.1%)	79.9% (71.0 to 87.4%)	34.5% (21.8 to 48.4%)	69.6% (45.1 to 84.7%)
Macedonia	14 (11-19)	12.3 (9.1-16.0)	-89.0% (-92.2 to -84.3%)	10,319.6 (7,873.7-13,210.6)	-23.9% (-33.6 to -12.6%)	0.1% (0.1-0.1%)	81.0% (71.9 to 88.4%)	64.9% (50.4 to 77.5%)	67.2% (40.2 to 84.2%)
Montenegro	1 (1-2)	3.9 (2.7-5.2)	-87.1% (-91.8 to -80.3%)	10,705.7 (8,214.9-13,546.1)	2.4% (-10.3 to 16.2%)	<0.1% (<0.1-<0.1%)	82.7% (74.4 to 89.2%)	64.3% (51.5 to 76.3%)	72.1% (45.4 to 87.3%)
Poland	64 (50-81)	3.4 (2.7-4.3)	-84.9% (-88.7 to -80.1%)	7,152.6 (5,384.8-9,023.9)	-1.6% (-14.1 to 11.3%)	<0.1% (<0.1-<0.1%)	77.0% (66.6 to 85.5%)	56.3% (43.3 to 68.6%)	67.3% (41.4 to 83.2%)
Romania	410 (350-482)	43.6 (37.3-51.3)	-78.7% (-81.9 to -74.7%)	10,716.6 (8,757.5-13,054.0)	-50.3% (-57.3 to -41.6%)	0.4% (0.4-0.4%)	80.0% (70.1 to 87.4%)	57.5% (43.1 to 71.3%)	71.7% (46.4 to 86.4%)
Serbia	18 (14-24)	3.9 (2.9-5.1)	-91.7% (-94.3 to -87.4%)	9,343.1 (7,382.6-11,707.6)	6.0% (-7.2 to 20.9%)	<0.1% (<0.1-<0.1%)	84.1% (76.3 to 90.0%)	62.6% (49.3 to 74.3%)	70.8% (46.5 to 85.3%)
Slovakia	26 (19-35)	9.5 (7.0-12.7)	-74.3% (-81.5 to -63.6%)	8,901.6 (7,021.3-11,349.5)	-12.1% (-24.4 to 0.5%)	0.1% (0.1-0.1%)	75.9% (64.9 to 84.8%)	32.9% (20.4 to 48.1%)	67.5% (39.9 to 84.0%)
Slovenia	1 (1-1)	1.0 (0.8-1.3)	-88.2% (-91.3 to -84.2%)	8,543.9 (6,529.1-11,248.1)	8.3% (-4.9 to 21.4%)	<0.1% (<0.1-<0.1%)	77.9% (68.3 to 85.4%)	45.0% (29.8 to 61.8%)	67.9% (44.2 to 83.1%)
Eastern Europe	1,396 (1,277-1,509)	10.9 (10.0-11.8)	-78.2% (-80.2 to -76.5%)	11,710.4 (8,712.6-15,012.1)	-32.5% (-41.5 to -23.2%)	0.1% (0.1-0.1%)	81.2% (72.5 to 87.8%)	49.0% (34.2 to 63.1%)	72.6% (50.7 to 85.1%)

Location	Deaths (95% UI)	Mortality rate per 100,000 (95% UI)	Percent change mortality rate 1990-2017 (95% UI)	Incidence per 100,000 (95% UI)	Percent change incidence 1990 to 2017 (95% UI)	Case fatality ratio (95% UI)	Attributable fraction for all risks (95% UI)	Attributable fraction for prevention-associated risks (95% UI)	Attributable fraction for protection-associated risks (95% UI)
Belarus	26 (19-36)	4.8 (3.5-6.6)	-90.0% (-93.0 to -85.9%)	20,005.5 (15,178.0-25,805.8)	-7.5% (-22.1 to 10.7%)	<0.1% (<0.1-<0.1%)	77.2% (67.4 to 85.4%)	35.9% (22.1 to 51.6%)	69.1% (45.7 to 83.2%)
Estonia	4 (3-5)	5.3 (4.1-6.7)	-78.6% (-83.9 to -71.9%)	14,649.5 (10,618.2-18,971.5)	-3.1% (-18.1 to 13.1%)	<0.1% (<0.1-<0.1%)	73.4% (61.1 to 83.2%)	51.4% (40.0 to 64.0%)	68.4% (42.6 to 84.1%)
Latvia	6 (4-8)	5.9 (4.3-7.7)	-68.8% (-77.2 to -58.9%)	15,877.3 (11,780.0-20,415.0)	7.2% (-7.6 to 24.8%)	<0.1% (<0.1-<0.1%)	78.0% (68.3 to 85.5%)	34.3% (19.5 to 51.1%)	69.6% (47.9 to 83.4%)
Lithuania	12 (9-14)	7.9 (6.2-9.8)	-46.9% (-59.8 to -31.4%)	16,021.4 (11,913.1-20,625.1)	9.7% (-3.5 to 27.2%)	<0.1% (<0.1-0.1%)	73.6% (62.2 to 82.9%)	30.1% (16.6 to 46.0%)	68.2% (44.1 to 83.3%)
Moldova	100 (74-132)	52.4 (38.9-69.2)	-65.6% (-75.3 to -52.5%)	15,239.8 (11,934.0-18,866.7)	-41.2% (-49.3 to -31.2%)	0.3% (0.3-0.4%)	82.8% (77.4 to 87.3%)	51.6% (32.9 to 69.3%)	72.9% (59.4 to 82.3%)
Russian Federation	1,030 (947-1,119)	11.0 (10.1-11.9)	-79.1% (-80.8 to -77.4%)	11,060.9 (8,124.5-14,239.9)	-34.7% (-43.6 to -25.2%)	0.1% (0.1-0.1%)	81.1% (72.5 to 88.0%)	45.5% (31.7 to 60.5%)	72.8% (50.4 to 85.6%)
Ukraine	218 (171-266)	9.3 (7.3-11.3)	-74.4% (-80.1 to -68.0%)	11,525.2 (8,523.5-14,806.0)	-32.9% (-42.8 to -23.0%)	0.1% (0.1-0.1%)	82.0% (72.8 to 89.2%)	65.3% (43.6 to 81.6%)	73.6% (48.2 to 87.9%)
High-income	1,857 (1,702-2,027)	3.2 (2.9-3.5)	-70.6% (-73.2 to -67.9%)	4,843.7 (3,772.5-6,137.4)	-19.5% (-24.9 to -13.4%)	0.1% (0.1-0.1%)	67.7% (55.8 to 78.1%)	28.8% (16.0 to 44.7%)	66.5% (42.0 to 82.1%)
High-income Asia Pacific	180 (163-197)	2.4 (2.2-2.6)	-72.2% (-75.9 to -68.4%)	8,472.0 (6,595.5-10,872.2)	-3.9% (-14.8 to 8.2%)	<0.1% (<0.1-<0.1%)	75.3% (63.3 to 84.6%)	33.3% (20.9 to 48.9%)	70.5% (43.5 to 85.9%)

Location	Deaths (95% UI)	Mortality rate per 100,000 (95% UI)	Percent change mortality rate 1990-2017 (95% UI)	Incidence per 100,000 (95% UI)	Percent change incidence 1990 to 2017 (95% UI)	Case fatality ratio (95% UI)	Attributable fraction for all risks (95% UI)	Attributable fraction for prevention-associated risks (95% UI)	Attributable fraction for protection-associated risks (95% UI)
Brunei	5 (4-6)	15.2 (11.7-19.0)	-5.9% (-31.0 to 25.5%)	9,781.2 (7,705.5-12,229.4)	-11.5% (-22.0 to 1.0%)	0.2% (0.2-0.2%)	74.3% (63.3 to 83.2%)	48.3% (37.6 to 61.3%)	71.8% (46.0 to 87.0%)
Japan	131 (118-146)	2.6 (2.4-2.9)	-57.6% (-62.3 to -52.8%)	8,410.7 (6,517.1-10,820.2)	25.0% (13.8 to 37.6%)	<0.1% (<0.1-<0.1%)	76.1% (64.2 to 85.3%)	31.3% (18.5 to 48.1%)	72.1% (44.4 to 87.4%)
South Korea	33 (27-41)	1.5 (1.2-1.8)	-88.2% (-91.2 to -84.3%)	8,063.1 (6,026.6-10,501.4)	-36.6% (-47.5 to -24.9%)	<0.1% (<0.1-<0.1%)	71.9% (60.4 to 81.8%)	39.3% (26.0 to 54.2%)	63.7% (36.5 to 81.2%)
Singapore	11 (8-14)	3.6 (2.8-4.7)	-78.2% (-84.0 to -70.7%)	12,485.7 (10,004.1-15,571.7)	2.4% (-11.7 to 18.6%)	<0.1% (<0.1-<0.1%)	76.4% (64.7 to 85.9%)	33.9% (19.2 to 50.2%)	71.7% (46.9 to 85.9%)
Australasia	42 (32-53)	2.3 (1.8-2.9)	-65.4% (-76.2 to -53.4%)	5,798.0 (4,448.5-7,449.4)	2.2% (-6.7 to 11.5%)	<0.1% (<0.1-<0.1%)	66.6% (53.8 to 78.1%)	23.0% (10.1 to 41.9%)	65.8% (40.8 to 82.1%)
Australia	34 (25-46)	2.3 (1.7-3.0)	-56.4% (-71.6 to -38.6%)	4,994.6 (3,739.8-6,462.2)	-7.1% (-17.0 to 3.5%)	<0.1% (<0.1-<0.1%)	65.9% (52.6 to 77.9%)	23.7% (10.3 to 43.9%)	65.5% (39.2 to 82.6%)
New Zealand	7 (6-9)	2.5 (2.1-3.0)	-81.1% (-85.8 to -75.9%)	9,948.5 (7,729.8-12,518.8)	41.9% (24.7 to 58.9%)	<0.1% (<0.1-<0.1%)	69.8% (58.4 to 79.7%)	20.1% (8.3 to 38.5%)	67.8% (46.3 to 82.1%)
Western Europe	383 (350-426)	1.7 (1.6-1.9)	-72.0% (-75.6 to -68.8%)	1,940.4 (1,526.6-2,431.0)	-19.1% (-24.6 to -13.2%)	0.1% (0.1-0.1%)	67.4% (55.2 to 78.1%)	28.1% (16.0 to 43.6%)	64.5% (40.9 to 80.1%)
Andorra	0 (0-0)	1.5 (1.0-2.2)	-76.5% (-86.8 to -60.3%)	1,614.7 (1,224.0-2,116.0)	-39.8% (-47.8 to -31.9%)	0.1% (0.1-0.1%)	63.8% (49.4 to 76.9%)	27.5% (12.9 to 49.3%)	60.8% (32.0 to 80.1%)

Location	Deaths (95% UI)	Mortality rate per 100,000 (95% UI)	Percent change mortality rate 1990-2017 (95% UI)	Incidence per 100,000 (95% UI)	Percent change incidence 1990 to 2017 (95% UI)	Case fatality ratio (95% UI)	Attributable fraction for all risks (95% UI)	Attributable fraction for prevention-associated risks (95% UI)	Attributable fraction for protection-associated risks (95% UI)
Austria	4 (3-6)	1.0 (0.8-1.3)	-84.5% (-88.8 to -78.6%)	2,293.2 (1,772.3-2,875.3)	-17.2% (-26.2 to -7.4%)	<0.1% (<0.1-<0.1%)	70.5% (59.2 to 80.2%)	54.1% (40.1 to 68.0%)	65.0% (40.4 to 81.1%)
Belgium	11 (8-14)	1.7 (1.3-2.2)	-62.8% (-73.8 to -48.9%)	2,140.9 (1,654.2-2,730.4)	-9.1% (-18.4 to 1.4%)	0.1% (0.1-0.1%)	67.8% (55.8 to 78.7%)	29.9% (17.4 to 45.5%)	63.3% (36.8 to 80.9%)
Cyprus	1 (1-1)	1.0 (0.8-1.3)	-90.6% (-93.9 to -84.4%)	1,496.1 (1,152.0-1,895.2)	-15.4% (-23.9 to -6.4%)	0.1% (0.1-0.1%)	77.0% (67.3 to 84.8%)	38.8% (24.9 to 53.6%)	68.3% (46.3 to 82.9%)
Denmark	5 (4-6)	1.6 (1.2-2.1)	-71.1% (-80.8 to -58.0%)	1,821.7 (1,401.5-2,349.1)	-19.5% (-27.6 to -10.5%)	0.1% (0.1-0.1%)	65.8% (52.6 to 77.0%)	23.6% (11.6 to 39.2%)	63.7% (38.1 to 80.4%)
Finland	1 (1-2)	0.4 (0.3-0.6)	-88.7% (-92.6 to -83.2%)	2,066.9 (1,588.5-2,659.2)	-7.9% (-17.0 to 1.7%)	<0.1% (<0.1-<0.1%)	66.8% (54.8 to 77.3%)	23.5% (10.3 to 42.8%)	65.1% (41.3 to 80.6%)
France	45 (36-56)	1.2 (0.9-1.5)	-67.9% (-76.5 to -57.5%)	1,610.0 (1,217.2-2,053.4)	-16.3% (-25.4 to -6.2%)	0.1% (0.1-0.1%)	63.8% (49.7 to 76.0%)	27.2% (12.5 to 46.7%)	62.8% (35.9 to 80.5%)
Germany	47 (36-60)	1.3 (1.0-1.7)	-66.6% (-75.2 to -55.1%)	1,943.5 (1,493.3-2,482.1)	-11.2% (-20.6 to -1.3%)	0.1% (0.1-0.1%)	63.5% (51.2 to 75.4%)	29.8% (15.7 to 48.2%)	61.7% (35.9 to 79.3%)
Greece	17 (12-26)	3.8 (2.6-5.7)	-25.4% (-48.0 to 11.2%)	1,975.0 (1,523.9-2,521.1)	-16.2% (-24.8 to -5.7%)	0.2% (0.2-0.2%)	71.4% (59.6 to 81.9%)	36.2% (20.9 to 53.3%)	65.2% (36.8 to 83.4%)
Iceland	0 (0-1)	2.1 (1.5-2.8)	-63.6% (-74.3 to -47.0%)	1,782.2 (1,372.3-2,311.9)	-21.4% (-29.8 to -11.6%)	0.1% (0.1-0.1%)	63.2% (48.9 to 76.0%)	25.9% (10.9 to 50.0%)	62.4% (36.2 to 79.7%)

Location	Deaths (95% UI)	Mortality rate per 100,000 (95% UI)	Percent change mortality rate 1990-2017 (95% UI)	Incidence per 100,000 (95% UI)	Percent change incidence 1990 to 2017 (95% UI)	Case fatality ratio (95% UI)	Attributable fraction for all risks (95% UI)	Attributable fraction for prevention-associated risks (95% UI)	Attributable fraction for protection-associated risks (95% UI)
Ireland	4 (3-5)	1.1 (0.9-1.4)	-79.2% (-84.8 to -72.0%)	1,587.0 (1,206.0-2,063.3)	-26.5% (-34.9 to -17.2%)	0.1% (0.1-0.1%)	68.5% (56.9 to 78.3%)	25.3% (12.6 to 42.6%)	65.1% (41.4 to 80.3%)
Israel	17 (13-22)	1.9 (1.5-2.5)	-80.6% (-85.4 to -74.6%)	1,362.5 (1,042.6-1,738.5)	-46.4% (-54.5 to -38.1%)	0.1% (0.1-0.1%)	72.2% (60.6 to 82.3%)	34.7% (19.3 to 53.7%)	65.2% (40.6 to 81.5%)
Italy	36 (27-48)	1.4 (1.1-1.9)	-81.0% (-85.6 to -74.2%)	1,812.3 (1,460.2-2,226.6)	-21.9% (-31.6 to -11.1%)	0.1% (0.1-0.1%)	72.0% (60.2 to 82.1%)	29.8% (15.1 to 46.7%)	65.6% (40.6 to 81.9%)
Luxembourg	0 (0-1)	1.2 (0.8-1.6)	-79.5% (-87.1 to -69.5%)	1,939.0 (1,505.9-2,447.5)	-14.7% (-24.7 to -3.1%)	0.1% (0.1-0.1%)	67.1% (54.6 to 78.1%)	25.6% (13.2 to 42.1%)	63.6% (37.9 to 80.7%)
Malta	1 (1-1)	4.0 (2.8-5.6)	-50.3% (-65.8 to -28.5%)	1,993.6 (1,538.0-2,523.7)	-19.8% (-29.7 to -9.4%)	0.2% (0.2-0.2%)	73.0% (62.0 to 82.3%)	55.0% (43.4 to 66.6%)	66.0% (41.9 to 81.7%)
Netherlands	11 (9-14)	1.3 (1.0-1.7)	-63.3% (-73.9 to -49.0%)	1,358.9 (1,030.1-1,759.7)	-19.0% (-27.5 to -9.5%)	0.1% (0.1-0.1%)	69.5% (58.3 to 79.5%)	29.2% (15.7 to 46.9%)	65.3% (41.0 to 81.3%)
Norway	2 (2-2)	0.7 (0.6-0.8)	-84.0% (-86.4 to -81.3%)	5,411.7 (4,115.6-7,016.1)	58.9% (44.4 to 75.1%)	<0.1% (<0.1-<0.1%)	65.9% (54.0 to 76.5%)	21.5% (10.4 to 38.3%)	64.2% (41.6 to 79.2%)
Portugal	13 (9-17)	3.1 (2.2-4.2)	-86.4% (-90.1 to -81.6%)	1,730.7 (1,342.6-2,180.0)	-51.1% (-59.0 to -43.0%)	0.2% (0.2-0.2%)	70.9% (59.2 to 80.7%)	26.6% (12.0 to 45.2%)	67.5% (44.1 to 82.4%)
Spain	32 (25-42)	1.5 (1.2-2.0)	-77.2% (-82.9 to -70.3%)	1,400.7 (1,088.4-1,802.6)	-20.7% (-29.4 to -12.4%)	0.1% (0.1-0.1%)	69.6% (56.7 to 80.2%)	30.3% (15.3 to 48.7%)	66.3% (42.2 to 81.5%)

Location	Deaths (95% UI)	Mortality rate per 100,000 (95% UI)	Percent change mortality rate 1990-2017 (95% UI)	Incidence per 100,000 (95% UI)	Percent change incidence 1990 to 2017 (95% UI)	Case fatality ratio (95% UI)	Attributable fraction for all risks (95% UI)	Attributable fraction for prevention-associated risks (95% UI)	Attributable fraction for protection-associated risks (95% UI)
Sweden	7 (5-9)	1.2 (0.9-1.5)	-72.3% (-79.6 to -63.8%)	2,327.1 (1,763.4-3,076.6)	-12.6% (-20.3 to -3.5%)	0.1% (<0.1-0.1%)	62.5% (48.6 to 74.6%)	17.1% (6.8 to 34.7%)	63.5% (37.9 to 80.3%)
Switzerland	5 (4-7)	1.2 (0.9-1.6)	-83.1% (-88.1 to -76.7%)	1,652.9 (1,292.4-2,077.9)	-24.6% (-33.5 to -15.0%)	0.1% (0.1-0.1%)	69.2% (57.2 to 79.4%)	30.8% (17.1 to 47.5%)	64.8% (40.3 to 80.9%)
United Kingdom	123 (113-131)	3.1 (2.9-3.3)	-64.4% (-69.4 to -60.7%)	2,584.8 (2,080.3-3,193.4)	-24.9% (-29.7 to -19.4%)	0.1% (0.1-0.1%)	66.7% (53.9 to 77.4%)	24.3% (12.0 to 41.6%)	65.0% (42.0 to 80.5%)
England	108 (100-114)	3.2 (3.0-3.4)	-64.8% (-69.7 to -61.4%)	2,746.8 (2,217.5-3,380.9)	-24.9% (-29.6 to -19.4%)	0.1% (0.1-0.1%)	66.5% (53.5 to 77.4%)	24.9% (12.4 to 42.3%)	64.8% (41.8 to 80.4%)
Northern Ireland	3 (2-4)	2.7 (2.0-3.5)	-72.4% (-80.8 to -61.3%)	1,718.1 (1,302.1-2,222.6)	-38.7% (-46.2 to -30.0%)	0.2% (0.2-0.2%)	69.7% (58.1 to 79.7%)	21.1% (8.9 to 38.6%)	66.2% (42.8 to 81.4%)
Scotland	7 (5-10)	2.6 (1.8-3.8)	-59.5% (-74.4 to -38.3%)	1,499.4 (1,154.4-1,942.9)	-26.1% (-35.0 to -15.5%)	0.2% (0.2-0.2%)	67.1% (54.6 to 78.1%)	19.2% (6.5 to 39.2%)	66.0% (40.2 to 82.4%)
Wales	4 (3-6)	2.6 (2.0-3.5)	-61.1% (-72.6 to -44.8%)	1,784.3 (1,372.8-2,311.8)	-33.0% (-41.0 to -24.5%)	0.1% (0.1-0.1%)	67.9% (55.2 to 78.9%)	21.5% (8.7 to 41.0%)	66.2% (41.4 to 82.0%)
Southern Latin America	568 (466-696)	11.1 (9.1-13.6)	-77.5% (-82.0 to -72.3%)	11,895.3 (9,444.5-14,789.3)	-11.2% (-23.8 to 2.8%)	0.1% (0.1-0.1%)	78.3% (67.7 to 86.7%)	37.1% (19.7 to 56.4%)	73.2% (48.2 to 87.0%)
Argentina	477 (374-595)	12.9 (10.2-16.2)	-71.5% (-78.0 to -63.5%)	11,947.8 (9,538.9-14,855.8)	0.5% (-14.9 to 17.9%)	0.1% (0.1-0.1%)	79.3% (68.5 to 87.7%)	38.0% (19.3 to 58.8%)	74.4% (49.2 to 88.1%)

Location	Deaths (95% UI)	Mortality rate per 100,000 (95% UI)	Percent change mortality rate 1990-2017 (95% UI)	Incidence per 100,000 (95% UI)	Percent change incidence 1990 to 2017 (95% UI)	Case fatality ratio (95% UI)	Attributable fraction for all risks (95% UI)	Attributable fraction for prevention-associated risks (95% UI)	Attributable fraction for protection-associated risks (95% UI)
Chile	63 (45-90)	5.3 (3.8-7.5)	-91.4% (-94.0 to -87.7%)	12,174.2 (9,422.1-15,325.6)	-28.8% (-40.4 to -15.3%)	<0.1% (<0.1-<0.1%)	73.1% (62.0 to 82.0%)	33.9% (15.2 to 51.9%)	66.5% (42.1 to 82.2%)
Uruguay	28 (20-36)	11.7 (8.6-15.2)	-67.7% (-76.6 to -55.5%)	9,666.6 (7,725.9-11,917.4)	-25.1% (-35.1 to -13.7%)	0.1% (0.1-0.1%)	73.5% (61.9 to 82.7%)	29.5% (14.5 to 48.0%)	68.5% (41.6 to 84.8%)
High-income North America	684 (618-742)	3.2 (2.9-3.5)	-60.6% (-65.9 to -56.2%)	4,791.2 (3,679.0-6,155.8)	-29.6% (-33.9 to -25.3%)	0.1% (0.1-0.1%)	57.1% (43.7 to 70.2%)	21.0% (9.1 to 37.6%)	61.0% (36.9 to 78.4%)
Canada	47 (34-62)	2.3 (1.7-3.2)	-51.1% (-65.3 to -32.8%)	4,986.8 (3,778.6-6,538.3)	-9.2% (-17.7 to 0.4%)	<0.1% (<0.1-<0.1%)	65.4% (53.0 to 77.0%)	28.0% (13.7 to 47.9%)	64.9% (39.3 to 81.7%)
Greenland	1 (0-1)	13.2 (9.5-18.2)	-72.3% (-82.2 to -55.9%)	6,108.1 (4,690.4-7,843.3)	-32.5% (-40.1 to -24.3%)	0.2% (0.2-0.2%)	66.0% (51.3 to 77.8%)	27.5% (10.1 to 48.3%)	65.1% (37.2 to 82.7%)
United States	637 (574-691)	3.3 (2.9-3.5)	-61.1% (-66.3 to -56.5%)	4,771.1 (3,662.4-6,137.6)	-31.2% (-35.4 to -26.8%)	0.1% (0.1-0.1%)	56.5% (43.0 to 69.7%)	20.5% (8.6 to 37.3%)	60.7% (36.6 to 78.2%)
Latin America and Caribbean	21,606 (19,618-24,079)	42.4 (38.5-47.3)	-79.1% (-81.9 to -75.8%)	12,192.4 (9,920.3-14,782.1)	-37.4% (-42.4 to -31.9%)	0.3% (0.3-0.4%)	78.8% (70.5 to 85.9%)	44.5% (30.5 to 58.4%)	72.4% (45.9 to 87.8%)
Caribbean	3,932 (2,985-5,131)	100.5 (76.3-131.2)	-51.8% (-63.9 to -35.7%)	11,164.6 (8,986.4-13,596.2)	-9.9% (-18.2 to 0.5%)	0.9% (0.8-1.0%)	89.3% (84.1 to 93.2%)	68.9% (53.4 to 81.9%)	76.7% (52.9 to 90.1%)
Antigua and Barbuda	2 (1-2)	29.9 (20.0-43.2)	24.1% (-21.3 to 94.5%)	10,133.5 (8,041.2-12,805.7)	27.8% (12.9 to 44.0%)	0.3% (0.2-0.3%)	74.2% (62.5 to 83.3%)	53.2% (38.9 to 67.3%)	68.9% (44.2 to 83.9%)

Location	Deaths (95% UI)	Mortality rate per 100,000 (95% UI)	Percent change mortality rate 1990-2017 (95% UI)	Incidence per 100,000 (95% UI)	Percent change incidence 1990 to 2017 (95% UI)	Case fatality ratio (95% UI)	Attributable fraction for all risks (95% UI)	Attributable fraction for prevention-associated risks (95% UI)	Attributable fraction for protection-associated risks (95% UI)
The Bahamas	7 (5-10)	30.1 (21.6-42.3)	-42.0% (-60.1 to -15.6%)	8,021.9 (6,381.3-10,038.5)	1.5% (-9.6 to 14.7%)	0.4% (0.3-0.4%)	74.1% (62.9 to 83.4%)	32.3% (14.1 to 52.0%)	69.4% (44.1 to 84.8%)
Barbados	3 (2-4)	20.6 (13.3-30.3)	-24.4% (-52.2 to 15.3%)	9,199.1 (7,197.5-11,446.7)	22.6% (7.7 to 39.5%)	0.2% (0.2-0.3%)	78.0% (68.0 to 85.8%)	36.1% (19.0 to 53.9%)	70.5% (49.5 to 83.9%)
Belize	18 (13-25)	46.7 (33.1-65.9)	-63.1% (-74.4 to -44.8%)	7,261.9 (5,796.6-9,041.0)	-30.9% (-39.7 to -21.6%)	0.6% (0.6-0.7%)	82.2% (74.2 to 88.4%)	57.9% (41.2 to 73.8%)	72.6% (50.6 to 85.8%)
Bermuda	0 (0-0)	6.7 (5.0-8.8)	-50.8% (-64.8 to -31.7%)	7,448.5 (5,767.0-9,438.2)	9.0% (-4.3 to 23.6%)	0.1% (0.1-0.1%)	70.9% (58.3 to 81.3%)	61.8% (37.7 to 80.4%)	67.1% (41.3 to 83.1%)
Cuba	59 (47-72)	9.8 (7.8-12.0)	-61.6% (-70.1 to -50.5%)	6,606.6 (5,277.9-8,196.7)	1.6% (-10.1 to 15.0%)	0.1% (0.1-0.1%)	76.7% (65.8 to 85.5%)	54.3% (39.6 to 68.5%)	68.4% (45.0 to 82.8%)
Dominica	3 (2-4)	69.2 (46.9-98.4)	150.8% (62.6 to 287.8%)	10,175.6 (8,011.3-12,717.9)	55.0% (35.0 to 78.2%)	0.7% (0.6-0.8%)	76.9% (65.9 to 85.4%)	54.7% (40.6 to 68.5%)	70.3% (45.7 to 85.1%)
Dominican Republic	434 (301-619)	43.5 (30.2-62.0)	-72.5% (-81.9 to -58.9%)	11,556.7 (9,361.5-14,220.0)	-20.0% (-30.4 to -8.2%)	0.4% (0.3-0.4%)	80.6% (71.6 to 87.9%)	52.4% (32.4 to 71.1%)	72.1% (48.6 to 86.4%)
Grenada	2 (2-4)	30.6 (19.2-46.9)	-47.0% (-69.3 to -9.3%)	9,495.2 (7,296.0-12,039.1)	-5.7% (-18.6 to 8.2%)	0.3% (0.3-0.4%)	79.1% (69.1 to 86.9%)	56.2% (40.7 to 71.0%)	71.1% (46.7 to 85.4%)
Guyana	29 (20-39)	38.9 (27.4-52.9)	-48.0% (-65.0 to -23.8%)	6,981.9 (5,581.7-8,569.0)	-13.4% (-23.6 to -2.3%)	0.6% (0.5-0.6%)	87.7% (80.5 to 92.7%)	37.9% (19.6 to 58.5%)	78.1% (57.3 to 89.6%)

Location	Deaths (95% UI)	Mortality rate per 100,000 (95% UI)	Percent change mortality rate 1990-2017 (95% UI)	Incidence per 100,000 (95% UI)	Percent change incidence 1990 to 2017 (95% UI)	Case fatality ratio (95% UI)	Attributable fraction for all risks (95% UI)	Attributable fraction for prevention-associated risks (95% UI)	Attributable fraction for protection-associated risks (95% UI)
Haiti	3,144 (2,270-4,339)	212.1 (153.1-292.7)	-63.1% (-74.1 to -48.0%)	14,386.3 (11,426.0-17,880.2)	-28.6% (-36.4 to -18.6%)	1.5% (1.3-1.6%)	91.2% (86.5 to 94.5%)	72.4% (56.7 to 84.9%)	78.2% (53.5 to 91.6%)
Jamaica	26 (17-39)	13.9 (9.2-20.7)	-67.3% (-79.5 to -46.3%)	7,223.1 (5,589.5-8,962.2)	-15.3% (-27.5 to -1.6%)	0.2% (0.2-0.2%)	78.3% (68.9 to 85.7%)	45.5% (32.0 to 60.0%)	70.2% (46.2 to 84.5%)
Puerto Rico	14 (11-18)	8.8 (7.0-11.0)	-56.5% (-67.0 to -43.5%)	8,335.1 (6,301.8-10,767.1)	38.5% (18.9 to 62.1%)	0.1% (0.1-0.1%)	73.7% (62.5 to 82.8%)	21.5% (6.2 to 41.6%)	70.5% (49.9 to 83.4%)
Saint Lucia	2 (1-3)	18.3 (11.9-26.6)	-41.8% (-64.0 to -9.8%)	8,615.0 (6,742.8-10,813.8)	-3.0% (-16.1 to 11.7%)	0.2% (0.2-0.2%)	80.2% (70.6 to 87.7%)	54.8% (40.7 to 69.2%)	72.1% (51.3 to 84.6%)
Saint Vincent and the Grenadines	3 (2-4)	30.9 (21.2-43.1)	-37.2% (-59.3 to -4.3%)	7,694.2 (6,105.3-9,606.1)	-10.3% (-20.8 to 1.8%)	0.4% (0.3-0.4%)	80.1% (70.1 to 87.7%)	55.9% (41.6 to 69.9%)	72.1% (48.9 to 86.0%)
Suriname	22 (17-28)	45.3 (35.3-57.5)	-48.9% (-62.8 to -31.1%)	6,249.9 (4,922.9-7,763.3)	-18.0% (-27.0 to -7.8%)	0.7% (0.7-0.7%)	83.8% (74.3 to 90.4%)	54.0% (30.9 to 73.0%)	76.1% (52.1 to 89.0%)
Trinidad and Tobago	22 (15-31)	23.5 (16.0-33.8)	-42.8% (-62.6 to -14.1%)	7,784.3 (6,191.7-9,792.1)	6.9% (-6.4 to 22.6%)	0.3% (0.3-0.3%)	80.7% (70.1 to 89.0%)	51.2% (28.1 to 71.3%)	71.1% (49.1 to 84.7%)
Virgin Islands, U.S.	1 (0-1)	8.9 (5.8-12.7)	-59.5% (-74.2 to -38.6%)	7,997.5 (6,145.2-10,061.5)	27.9% (11.1 to 46.9%)	0.1% (0.1-0.1%)	74.5% (64.0 to 83.3%)	36.6% (14.1 to 60.1%)	70.1% (49.3 to 83.4%)
Andean Latin America	3,787 (2,988-4,694)	56.5 (44.6-70.0)	-87.0% (-90.0 to -83.3%)	16,610.1 (14,120.4-19,324.6)	-40.3% (-46.5 to -33.0%)	0.3% (0.3-0.4%)	74.7% (64.8 to 83.0%)	40.0% (23.0 to 57.9%)	68.6% (40.4 to 86.2%)

Location	Deaths (95% UI)	Mortality rate per 100,000 (95% UI)	Percent change mortality rate 1990-2017 (95% UI)	Incidence per 100,000 (95% UI)	Percent change incidence 1990 to 2017 (95% UI)	Case fatality ratio (95% UI)	Attributable fraction for all risks (95% UI)	Attributable fraction for prevention-associated risks (95% UI)	Attributable fraction for protection-associated risks (95% UI)
Bolivia	1,247 (771-1,813)	86.0 (53.1-125.0)	-87.8% (-92.5 to -81.4%)	15,368.5 (12,246.0-19,221.4)	-49.7% (-57.1 to -41.7%)	0.6% (0.4-0.7%)	75.3% (65.2 to 84.0%)	35.3% (17.0 to 56.3%)	67.2% (38.9 to 85.5%)
Ecuador	795 (551-1,112)	51.1 (35.4-71.4)	-69.1% (-78.9 to -56.4%)	18,755.8 (15,191.2-22,984.0)	-16.3% (-27.4 to -2.9%)	0.3% (0.2-0.3%)	74.5% (63.2 to 83.7%)	36.1% (15.9 to 58.4%)	71.9% (41.8 to 89.0%)
Peru	1,745 (1,215-2,423)	47.2 (32.9-65.6)	-89.7% (-93.1 to -84.9%)	16,192.8 (14,067.7-18,290.6)	-44.5% (-51.7 to -36.3%)	0.3% (0.2-0.4%)	74.4% (64.6 to 83.0%)	45.1% (24.4 to 65.2%)	67.4% (38.2 to 85.6%)
Central Latin America	9,257 (8,062-10,826)	38.3 (33.3-44.7)	-73.6% (-77.4 to -68.5%)	15,259.9 (12,336.1-18,680.2)	-39.9% (-45.4 to -33.9%)	0.3% (0.2-0.3%)	78.9% (70.7 to 85.6%)	41.8% (26.9 to 56.8%)	72.5% (45.2 to 88.0%)
Colombia	1,160 (804-1,628)	27.4 (19.0-38.5)	-71.5% (-80.8 to -59.6%)	17,728.8 (14,161.0-22,384.8)	-43.2% (-51.7 to -33.4%)	0.2% (0.1-0.2%)	72.5% (61.6 to 81.6%)	31.9% (13.8 to 53.3%)	69.0% (41.3 to 85.8%)
Costa Rica	27 (19-38)	7.6 (5.5-10.8)	-78.0% (-83.7 to -70.6%)	19,307.1 (14,911.7-24,711.4)	14.5% (-2.1 to 32.5%)	<0.1% (<0.1-<0.1%)	74.6% (64.7 to 82.9%)	27.7% (15.0 to 42.1%)	67.0% (43.5 to 82.3%)
El Salvador	148 (93-225)	27.3 (17.1-41.4)	-76.6% (-86.2 to -61.8%)	21,804.2 (17,465.9-27,277.5)	-29.1% (-39.2 to -17.7%)	0.1% (0.1-0.2%)	78.7% (68.9 to 86.5%)	38.8% (23.5 to 55.1%)	72.4% (41.7 to 89.1%)
Guatemala	3,069 (2,203-4,181)	154.4 (110.9-210.4)	-62.2% (-73.5 to -47.3%)	27,126.3 (22,443.4-32,304.3)	-40.7% (-49.1 to -29.3%)	0.6% (0.5-0.7%)	80.5% (73.7 to 86.5%)	49.4% (30.8 to 67.4%)	72.5% (40.6 to 90.4%)
Honduras	163 (105-263)	13.9 (9.0-22.4)	-88.0% (-92.8 to -80.8%)	21,655.3 (17,136.7-27,068.7)	-29.4% (-38.4 to -19.3%)	0.1% (0.1-0.1%)	83.7% (77.0 to 89.0%)	45.1% (30.4 to 61.0%)	73.1% (46.4 to 88.6%)

Location	Deaths (95% UI)	Mortality rate per 100,000 (95% UI)	Percent change mortality rate 1990-2017 (95% UI)	Incidence per 100,000 (95% UI)	Percent change incidence 1990 to 2017 (95% UI)	Case fatality ratio (95% UI)	Attributable fraction for all risks (95% UI)	Attributable fraction for prevention-associated risks (95% UI)	Attributable fraction for protection-associated risks (95% UI)
Mexico	3,383 (3,090-3,832)	28.1 (25.6-31.8)	-81.2% (-83.5 to -77.6%)	11,184.0 (9,064.8-13,846.6)	-48.0% (-52.6 to -43.1%)	0.3% (0.2-0.3%)	78.4% (69.1 to 85.8%)	37.3% (21.4 to 54.1%)	73.3% (47.0 to 87.8%)
Nicaragua	318 (240-411)	46.7 (35.3-60.3)	-79.3% (-84.9 to -71.9%)	14,361.8 (11,293.1-18,075.4)	-49.9% (-56.8 to -41.8%)	0.3% (0.3-0.3%)	80.1% (72.6 to 86.3%)	38.4% (24.9 to 52.8%)	70.7% (46.1 to 85.9%)
Panama	164 (113-236)	45.4 (31.3-65.3)	7.0% (-26.5 to 50.3%)	21,622.0 (17,514.5-26,571.8)	22.7% (7.1 to 39.5%)	0.2% (0.2-0.2%)	71.7% (60.8 to 81.2%)	33.4% (16.2 to 53.2%)	67.0% (39.1 to 85.0%)
Venezuela	824 (605-1,101)	29.2 (21.5-39.1)	-63.2% (-73.6 to -49.4%)	15,595.5 (12,520.1-19,280.5)	-20.7% (-31.8 to -8.6%)	0.2% (0.2-0.2%)	84.2% (75.4 to 90.3%)	49.6% (28.0 to 68.5%)	76.9% (56.7 to 88.1%)
Tropical Latin America	4,630 (4,163-5,158)	28.8 (25.9-32.0)	-85.8% (-88.6 to -83.7%)	5,990.7 (4,896.3-7,296.4)	-45.2% (-49.3 to -40.9%)	0.5% (0.4-0.5%)	73.2% (62.3 to 82.7%)	28.0% (17.2 to 41.5%)	70.6% (41.9 to 87.4%)
Brazil	4,490 (4,033-4,991)	29.2 (26.3-32.5)	-85.8% (-88.6 to -83.6%)	5,702.3 (4,674.9-6,910.3)	-47.3% (-51.0 to -43.2%)	0.5% (0.5-0.6%)	73.1% (62.2 to 82.6%)	27.8% (16.9 to 41.5%)	70.6% (41.9 to 87.4%)
Paraguay	141 (90-208)	18.9 (12.1-28.0)	-81.4% (-88.3 to -71.6%)	11,952.5 (9,280.5-15,225.4)	-17.1% (-31.4 to -2.0%)	0.2% (0.1-0.2%)	76.3% (65.0 to 85.4%)	35.6% (21.8 to 50.4%)	71.6% (41.0 to 88.7%)
North Africa and Middle East	43,558 (37,550-49,735)	67.7 (58.3-77.3)	-76.5% (-80.7 to -71.1%)	19,258.4 (15,414.9-23,501.0)	-25.6% (-32.2 to -19.0%)	0.4% (0.3-0.4%)	91.9% (87.6 to 94.9%)	62.3% (46.8 to 75.6%)	81.1% (58.5 to 92.3%)
Afghanistan	16,998 (12,918-21,804)	288.7 (219.4-370.3)	-76.1% (-83.4 to -61.0%)	26,953.5 (21,763.9-33,723.3)	-28.6% (-36.5 to -16.5%)	1.1% (1.0-1.1%)	93.8% (90.4 to 96.1%)	68.0% (46.8 to 83.5%)	80.1% (54.6 to 92.8%)

Location	Deaths (95% UI)	Mortality rate per 100,000 (95% UI)	Percent change mortality rate 1990-2017 (95% UI)	Incidence per 100,000 (95% UI)	Percent change incidence 1990 to 2017 (95% UI)	Case fatality ratio (95% UI)	Attributable fraction for all risks (95% UI)	Attributable fraction for prevention-associated risks (95% UI)	Attributable fraction for protection-associated risks (95% UI)
Algeria	811 (561-1,106)	18.1 (12.5-24.7)	-82.8% (-88.4 to -74.0%)	16,656.7 (13,376.0-20,601.4)	-21.1% (-30.8 to -10.4%)	0.1% (0.1-0.1%)	87.4% (79.7 to 92.6%)	41.2% (20.8 to 60.7%)	78.7% (55.8 to 91.2%)
Bahrain	4 (3-5)	3.6 (2.6-4.7)	-78.9% (-86.3 to -67.6%)	16,011.8 (11,849.5-20,588.1)	28.2% (10.8 to 43.8%)	<0.1% (<0.1-<0.1%)	82.9% (73.5 to 90.0%)	46.6% (34.3 to 60.2%)	74.0% (45.8 to 89.0%)
Egypt	10,375 (7,761-13,648)	87.9 (65.8-115.7)	-85.2% (-89.6 to -79.5%)	26,658.4 (20,785.2-33,161.9)	-18.1% (-32.1 to -3.0%)	0.3% (0.3-0.3%)	89.3% (82.6 to 93.7%)	67.4% (52.6 to 79.1%)	78.1% (52.8 to 91.0%)
Iran	1,125 (1,025-1,225)	16.5 (15.0-18.0)	-86.3% (-88.2 to -84.3%)	8,456.8 (6,439.9-10,657.2)	-49.0% (-54.5 to -42.7%)	0.2% (0.2-0.2%)	85.5% (77.4 to 91.3%)	60.1% (50.4 to 69.3%)	75.7% (54.1 to 87.7%)
Iraq	2,042 (1,577-2,635)	34.4 (26.6-44.4)	-72.9% (-81.0 to -62.4%)	16,000.4 (12,505.4-20,197.5)	-30.2% (-37.4 to -22.8%)	0.2% (0.2-0.2%)	89.7% (82.7 to 94.3%)	63.3% (43.5 to 79.6%)	81.0% (56.7 to 92.5%)
Jordan	361 (267-476)	30.9 (22.9-40.8)	-68.0% (-77.6 to -54.2%)	15,440.7 (12,396.1-19,031.8)	-20.5% (-29.8 to -11.3%)	0.2% (0.2-0.2%)	82.1% (73.3 to 88.9%)	59.7% (47.3 to 71.2%)	69.9% (45.5 to 84.7%)
Kuwait	23 (18-29)	7.6 (5.9-9.6)	-71.9% (-79.7 to -62.7%)	15,295.4 (11,690.9-19,395.5)	-13.1% (-26.0 to 0.1%)	<0.1% (<0.1-0.1%)	81.7% (72.1 to 89.0%)	47.6% (34.9 to 61.5%)	69.6% (43.5 to 84.5%)
Lebanon	45 (28-69)	5.0 (3.1-7.7)	-87.0% (-92.6 to -77.5%)	21,680.6 (16,166.6-28,291.3)	40.8% (21.0 to 59.1%)	<0.1% (<0.1-<0.1%)	83.0% (73.8 to 89.6%)	45.6% (28.4 to 62.4%)	74.1% (48.1 to 88.6%)
Libya	52 (34-76)	8.2 (5.4-11.9)	-84.2% (-90.4 to -73.2%)	17,053.0 (13,295.7-21,743.4)	-7.3% (-18.4 to 5.6%)	<0.1% (<0.1-0.1%)	85.2% (75.3 to 91.9%)	48.4% (23.5 to 71.5%)	76.1% (47.6 to 90.5%)

Location	Deaths (95% UI)	Mortality rate per 100,000 (95% UI)	Percent change mortality rate 1990-2017 (95% UI)	Incidence per 100,000 (95% UI)	Percent change incidence 1990 to 2017 (95% UI)	Case fatality ratio (95% UI)	Attributable fraction for all risks (95% UI)	Attributable fraction for prevention-associated risks (95% UI)	Attributable fraction for protection-associated risks (95% UI)
Morocco	952 (642-1,321)	31.1 (20.9-43.1)	-86.5% (-90.9 to -79.5%)	17,262.5 (13,358.0-21,781.8)	-37.9% (-46.7 to -28.9%)	0.2% (0.2-0.2%)	86.2% (79.5 to 91.3%)	39.4% (23.5 to 55.9%)	75.9% (52.8 to 89.1%)
Palestine	61 (45-82)	9.4 (6.8-12.5)	-84.7% (-90.1 to -76.4%)	15,832.6 (12,508.7-19,705.0)	-24.9% (-33.7 to -12.8%)	0.1% (0.1-0.1%)	79.7% (69.1 to 87.8%)	36.5% (20.1 to 55.9%)	71.1% (42.8 to 86.9%)
Oman	34 (27-43)	8.6 (6.8-10.8)	-86.7% (-91.0 to -80.4%)	15,406.6 (11,718.2-19,677.5)	-28.5% (-38.3 to -18.1%)	0.1% (0.1-0.1%)	88.8% (81.4 to 93.7%)	38.5% (23.6 to 54.4%)	80.8% (57.9 to 91.9%)
Qatar	6 (4-8)	3.9 (2.6-5.4)	-64.5% (-76.7 to -46.8%)	18,589.9 (13,860.0-24,183.7)	34.6% (16.9 to 57.4%)	<0.1% (<0.1-<0.1%)	84.3% (75.8 to 90.9%)	50.4% (36.3 to 65.4%)	73.7% (45.5 to 88.8%)
Saudi Arabia	46 (26-75)	1.8 (1.0-3.0)	-95.4% (-97.7 to -91.3%)	15,259.1 (11,414.0-19,865.6)	-20.3% (-31.5 to -6.6%)	<0.1% (<0.1-<0.1%)	79.7% (68.1 to 88.5%)	46.4% (26.0 to 66.2%)	70.1% (39.3 to 87.8%)
Sudan	5,885 (3,779-9,171)	97.0 (62.3-151.2)	-76.8% (-85.4 to -61.9%)	21,088.0 (16,347.0-26,711.2)	-35.3% (-43.7 to -25.6%)	0.5% (0.4-0.6%)	94.4% (91.0 to 96.7%)	55.1% (31.2 to 76.0%)	85.8% (65.6 to 95.4%)
Syria	199 (128-284)	13.9 (8.9-19.8)	-85.1% (-91.0 to -76.1%)	16,509.7 (12,702.8-20,991.7)	-23.4% (-32.5 to -15.0%)	0.1% (0.1-0.1%)	89.9% (82.5 to 94.6%)	65.7% (45.3 to 83.2%)	84.6% (60.7 to 95.1%)
Tunisia	75 (52-104)	8.2 (5.7-11.4)	-91.9% (-95.1 to -87.4%)	22,085.7 (17,186.4-27,873.4)	-5.0% (-18.0 to 11.0%)	<0.1% (<0.1-<0.1%)	83.1% (74.9 to 89.4%)	62.4% (48.8 to 74.8%)	72.5% (47.5 to 87.2%)
Turkey	642 (473-869)	10.9 (8.0-14.8)	-96.4% (-97.6 to -94.4%)	12,480.0 (9,915.4-15,809.3)	-55.9% (-61.6 to -49.8%)	0.1% (0.1-0.1%)	79.2% (70.2 to 87.2%)	44.6% (30.0 to 60.1%)	69.5% (39.3 to 87.3%)

Location	Deaths (95% UI)	Mortality rate per 100,000 (95% UI)	Percent change mortality rate 1990-2017 (95% UI)	Incidence per 100,000 (95% UI)	Percent change incidence 1990 to 2017 (95% UI)	Case fatality ratio (95% UI)	Attributable fraction for all risks (95% UI)	Attributable fraction for prevention-associated risks (95% UI)	Attributable fraction for protection-associated risks (95% UI)
United Arab Emirates	17 (11-25)	4.0 (2.7-5.9)	-71.6% (-83.0 to -53.2%)	15,511.9 (11,672.5-19,724.4)	15.7% (-1.6 to 33.8%)	<0.1% (<0.1-<0.1%)	87.6% (78.2 to 93.2%)	38.9% (22.8 to 56.9%)	80.1% (56.8 to 91.4%)
Yemen	3,765 (2,471-5,669)	78.4 (51.4-118.0)	-81.4% (-88.6 to -68.5%)	25,249.8 (20,078.9-31,217.1)	-37.7% (-43.7 to -31.3%)	0.3% (0.3-0.4%)	96.3% (93.5 to 98.0%)	61.1% (34.9 to 81.2%)	89.0% (71.2 to 96.9%)
South Asia	249,595 (225,643-275,313)	143.1 (129.4-157.9)	-71.2% (-74.9 to -66.7%)	13,153.1 (10,465.5-16,238.0)	-22.7% (-28.7 to -16.2%)	1.1% (1.0-1.2%)	95.9% (93.9 to 97.4%)	65.4% (50.5 to 77.3%)	83.2% (66.8 to 92.1%)
Bangladesh	19,678 (14,740-25,631)	134.8 (101.0-175.6)	-80.0% (-85.4 to -72.3%)	15,084.5 (12,414.6-17,938.3)	-42.4% (-49.1 to -34.0%)	0.9% (0.8-1.0%)	95.0% (92.0 to 97.0%)	57.0% (39.7 to 72.2%)	79.7% (59.4 to 91.3%)
Bhutan	59 (40-84)	66.1 (44.6-94.0)	-87.3% (-91.7 to -80.6%)	14,290.2 (10,769.2-18,203.8)	-40.8% (-48.6 to -31.4%)	0.5% (0.4-0.5%)	88.1% (82.6 to 92.3%)	59.8% (47.8 to 71.6%)	74.6% (51.6 to 88.3%)
India	185,429 (167,676-204,328)	143.4 (129.6-158.0)	-69.6% (-73.5 to -64.6%)	11,090.8 (8,713.9-13,886.2)	-23.3% (-29.7 to -16.6%)	1.3% (1.1-1.5%)	96.3% (94.4 to 97.5%)	67.7% (52.7 to 79.3%)	83.5% (67.8 to 92.2%)
Nepal	3,949 (2,883-5,233)	129.0 (94.2-170.9)	-83.2% (-88.3 to -76.2%)	13,381.8 (10,931.2-16,153.6)	-45.2% (-52.2 to -37.2%)	1.0% (0.9-1.1%)	91.5% (86.9 to 94.9%)	60.5% (40.1 to 77.6%)	75.3% (51.2 to 89.2%)
Pakistan	40,480 (28,805-57,002)	148.2 (105.4-208.6)	-66.2% (-77.4 to -49.9%)	21,854.0 (17,401.5-26,601.4)	-5.6% (-15.1 to 6.3%)	0.7% (0.6-0.8%)	95.1% (92.3 to 97.0%)	59.1% (39.0 to 77.0%)	84.4% (62.7 to 94.8%)
Sub-Saharan Africa	412,604 (357,299-471,442)	252.5 (218.7-288.6)	-62.9% (-67.6 to -56.8%)	10,493.2 (8,558.0-12,858.7)	-34.5% (-39.0 to -29.4%)	2.4% (2.2-2.6%)	94.0% (90.9 to 96.2%)	68.0% (50.6 to 81.8%)	82.8% (61.9 to 93.2%)

Location	Deaths (95% UI)	Mortality rate per 100,000 (95% UI)	Percent change mortality rate 1990-2017 (95% UI)	Incidence per 100,000 (95% UI)	Percent change incidence 1990 to 2017 (95% UI)	Case fatality ratio (95% UI)	Attributable fraction for all risks (95% UI)	Attributable fraction for prevention-associated risks (95% UI)	Attributable fraction for protection-associated risks (95% UI)
Central Sub-Saharan Africa	47,357 (37,232-58,184)	239.7 (188.4-294.5)	-61.8% (-69.9 to -51.0%)	11,728.4 (9,490.0-14,347.2)	-28.4% (-36.3 to -19.7%)	2.0% (2.0-2.1%)	94.2% (91.0 to 96.4%)	68.5% (48.8 to 83.9%)	82.9% (58.6 to 94.3%)
Angola	8,037 (5,924-10,551)	162.0 (119.4-212.6)	-83.0% (-88.3 to -76.2%)	8,296.9 (6,710.9-10,066.0)	-54.3% (-59.5 to -48.8%)	2.0% (1.8-2.1%)	92.4% (88.1 to 95.3%)	70.1% (48.5 to 85.4%)	81.7% (55.5 to 93.9%)
Central African Republic	3,095 (2,034-4,270)	510.6 (335.6-704.5)	-18.6% (-46.9 to 23.5%)	13,683.9 (10,646.9-17,269.4)	-15.0% (-25.5 to -3.8%)	3.7% (3.2-4.1%)	94.6% (91.7 to 96.8%)	74.8% (52.5 to 89.0%)	83.5% (59.4 to 94.6%)
Congo	578 (403-802)	91.5 (63.9-127.0)	-68.6% (-78.4 to -55.3%)	9,299.6 (7,351.2-11,422.1)	-28.0% (-37.1 to -19.1%)	1.0% (0.9-1.1%)	93.2% (88.9 to 96.0%)	63.5% (38.6 to 83.0%)	78.1% (55.4 to 91.0%)
Democratic Republic of the Congo	35,375 (26,138-46,339)	268.6 (198.5-351.9)	-52.5% (-64.3 to -34.6%)	13,133.0 (10,449.7-16,256.7)	-19.0% (-30.1 to -6.4%)	2.0% (1.9-2.2%)	94.6% (91.6 to 96.7%)	67.9% (46.2 to 84.5%)	83.4% (58.5 to 94.8%)
Equatorial Guinea	123 (75-189)	65.0 (39.6-99.6)	-91.5% (-94.8 to -86.0%)	8,327.3 (6,329.4-10,709.8)	-54.6% (-60.9 to -46.8%)	0.8% (0.6-0.9%)	89.8% (84.2 to 93.8%)	72.4% (50.9 to 87.2%)	76.9% (51.4 to 90.8%)
Gabon	149 (101-212)	74.9 (50.8-106.5)	-64.0% (-75.9 to -47.1%)	9,337.4 (7,350.2-11,891.8)	-16.7% (-26.8 to -6.2%)	0.8% (0.7-0.9%)	87.5% (81.0 to 92.3%)	66.3% (48.5 to 81.4%)	74.5% (50.0 to 89.0%)
Eastern Sub-Saharan Africa	111,613 (99,529-124,670)	176.3 (157.2-196.9)	-71.2% (-75.4 to -65.6%)	12,894.4 (10,363.6-15,813.8)	-33.7% (-38.7 to -28.6%)	1.4% (1.2-1.5%)	93.3% (90.0 to 95.7%)	66.7% (51.4 to 78.4%)	81.3% (59.0 to 92.6%)
Burundi	4,060 (2,952-5,484)	221.7 (161.2-299.4)	-57.6% (-69.6 to -38.8%)	14,768.2 (11,505.2-18,500.6)	-23.5% (-32.9 to -13.6%)	1.5% (1.4-1.6%)	95.2% (92.6 to 97.0%)	63.1% (44.9 to 79.1%)	83.7% (60.8 to 94.5%)

Location	Deaths (95% UI)	Mortality rate per 100,000 (95% UI)	Percent change mortality rate 1990-2017 (95% UI)	Incidence per 100,000 (95% UI)	Percent change incidence 1990 to 2017 (95% UI)	Case fatality ratio (95% UI)	Attributable fraction for all risks (95% UI)	Attributable fraction for prevention-associated risks (95% UI)	Attributable fraction for protection-associated risks (95% UI)
Comoros	100 (68-144)	117.2 (79.4-168.4)	-77.8% (-85.5 to -66.5%)	10,822.5 (8,437.6-13,465.5)	-37.9% (-45.5 to -30.4%)	1.1% (0.9-1.3%)	94.1% (90.4 to 96.6%)	70.4% (56.7 to 81.8%)	82.4% (60.1 to 93.2%)
Djibouti	167 (106-256)	110.9 (70.3-169.6)	-74.5% (-84.1 to -59.8%)	11,102.7 (8,642.9-14,273.1)	-41.8% (-50.3 to -32.5%)	1.0% (0.8-1.2%)	94.4% (90.6 to 97.0%)	62.8% (36.4 to 82.8%)	86.2% (66.8 to 95.0%)
Eritrea	1,787 (1,210-2,649)	218.1 (147.6-323.3)	-70.3% (-81.0 to -50.7%)	15,187.6 (11,907.4-19,320.1)	-39.4% (-46.8 to -30.7%)	1.4% (1.2-1.7%)	95.1% (91.9 to 97.3%)	56.9% (38.5 to 74.3%)	85.5% (62.6 to 95.4%)
Ethiopia	25,574 (21,346-29,909)	153.8 (128.4-179.9)	-77.2% (-82.6 to -70.5%)	10,649.3 (8,483.1-13,185.8)	-41.6% (-46.5 to -37.0%)	1.4% (1.4-1.5%)	94.5% (91.8 to 96.3%)	70.5% (53.0 to 83.1%)	82.7% (61.1 to 93.2%)
Kenya	9,544 (8,304-10,846)	146.6 (127.5-166.6)	-64.5% (-70.5 to -57.5%)	15,486.6 (12,319.6-19,066.2)	-7.0% (-14.5 to 0.5%)	0.9% (0.9-1.0%)	91.6% (87.9 to 94.4%)	60.5% (45.1 to 73.8%)	79.0% (57.4 to 91.0%)
Madagascar	11,639 (8,210-16,182)	267.5 (188.7-371.9)	-54.4% (-68.3 to -36.6%)	18,124.2 (14,388.4-22,688.6)	-11.1% (-24.0 to 5.3%)	1.5% (1.3-1.6%)	94.8% (91.9 to 96.8%)	65.8% (49.3 to 79.1%)	83.4% (59.3 to 94.5%)
Malawi	4,070 (2,993-5,431)	148.5 (109.2-198.2)	-73.8% (-82.3 to -54.6%)	14,349.1 (11,546.1-17,726.5)	-27.3% (-35.7 to -16.6%)	1.0% (0.9-1.1%)	90.9% (86.4 to 94.1%)	61.8% (45.2 to 76.1%)	76.6% (49.9 to 90.7%)
Mozambique	5,628 (4,103-7,618)	119.7 (87.3-162.0)	-83.9% (-89.0 to -76.2%)	9,658.4 (7,530.4-12,294.7)	-48.9% (-54.9 to -42.2%)	1.2% (1.2-1.3%)	91.0% (86.4 to 94.5%)	60.4% (45.4 to 73.6%)	76.6% (49.1 to 91.1%)
Rwanda	2,844 (2,012-3,937)	148.6 (105.1-205.7)	-75.4% (-83.7 to -63.6%)	17,505.2 (14,105.5-21,637.0)	-38.1% (-46.9 to -28.3%)	0.8% (0.7-1.0%)	90.2% (85.5 to 93.7%)	58.2% (39.4 to 74.9%)	74.7% (45.0 to 90.4%)

Location	Deaths (95% UI)	Mortality rate per 100,000 (95% UI)	Percent change mortality rate 1990-2017 (95% UI)	Incidence per 100,000 (95% UI)	Percent change incidence 1990 to 2017 (95% UI)	Case fatality ratio (95% UI)	Attributable fraction for all risks (95% UI)	Attributable fraction for prevention-associated risks (95% UI)	Attributable fraction for protection-associated risks (95% UI)
Somalia	7,945 (5,306-11,508)	267.9 (178.9-388.0)	-66.0% (-78.7 to -41.0%)	12,560.0 (9,899.2-16,282.5)	-34.3% (-40.9 to -27.3%)	2.1% (1.8-2.4%)	96.1% (93.7 to 97.7%)	80.6% (62.7 to 91.2%)	86.6% (66.5 to 95.8%)
South Sudan	9,220 (6,749-12,362)	527.7 (386.2-707.5)	-33.7% (-54.3 to 1.6%)	17,253.5 (13,445.4-21,652.7)	-14.5% (-23.4 to -5.2%)	3.1% (2.9-3.3%)	96.4% (94.1 to 97.9%)	78.5% (60.7 to 89.7%)	87.8% (69.2 to 96.0%)
Tanzania	17,618 (13,049-23,649)	195.5 (144.8-262.4)	-72.2% (-80.4 to -59.2%)	12,455.4 (10,142.4-15,228.1)	-36.7% (-45.1 to -28.9%)	1.6% (1.4-1.7%)	91.4% (86.9 to 94.7%)	61.3% (43.6 to 77.2%)	78.3% (51.5 to 92.0%)
Uganda	6,729 (4,927-8,753)	96.9 (70.9-126.0)	-73.4% (-81.3 to -63.1%)	12,921.5 (10,210.1-15,978.8)	-35.9% (-44.3 to -27.8%)	0.7% (0.7-0.8%)	90.5% (85.7 to 94.1%)	63.4% (44.1 to 79.8%)	75.9% (47.5 to 90.9%)
Zambia	4,617 (3,338-6,220)	161.0 (116.4-216.9)	-77.9% (-85.2 to -67.7%)	10,235.3 (8,388.1-12,370.5)	-53.6% (-58.9 to -47.5%)	1.6% (1.4-1.8%)	92.2% (88.1 to 95.0%)	53.5% (37.3 to 69.7%)	79.7% (55.1 to 92.3%)
Southern Sub-Saharan Africa	10,513 (9,192-12,063)	123.1 (107.7-141.3)	-54.7% (-61.5 to -46.6%)	7,357.1 (6,032.7-8,847.9)	-30.3% (-35.5 to -24.5%)	1.7% (1.6-1.8%)	87.4% (81.5 to 92.1%)	51.9% (36.5 to 66.3%)	77.0% (51.0 to 90.7%)
Botswana	113 (79-156)	47.0 (33.0-65.0)	-59.9% (-73.7 to -38.6%)	6,661.2 (5,223.5-8,249.0)	-23.7% (-32.5 to -14.0%)	0.7% (0.6-0.8%)	89.4% (83.6 to 93.7%)	52.2% (31.0 to 71.5%)	79.0% (54.5 to 91.6%)
Lesotho	400 (267-568)	176.8 (118.1-251.0)	-40.7% (-61.2 to -13.4%)	11,796.1 (9,313.9-14,588.3)	14.1% (1.2 to 28.2%)	1.5% (1.3-1.7%)	89.9% (84.8 to 93.6%)	54.9% (39.5 to 69.1%)	77.3% (47.5 to 92.4%)
Namibia	278 (185-391)	96.2 (63.9-135.1)	-53.8% (-69.3 to -31.6%)	11,138.2 (8,866.3-13,856.9)	-18.2% (-28.3 to -8.4%)	0.9% (0.7-1.0%)	90.4% (84.1 to 94.5%)	47.6% (29.1 to 67.0%)	80.4% (56.4 to 92.5%)

Location	Deaths (95% UI)	Mortality rate per 100,000 (95% UI)	Percent change mortality rate 1990-2017 (95% UI)	Incidence per 100,000 (95% UI)	Percent change incidence 1990 to 2017 (95% UI)	Case fatality ratio (95% UI)	Attributable fraction for all risks (95% UI)	Attributable fraction for prevention-associated risks (95% UI)	Attributable fraction for protection-associated risks (95% UI)
South Africa	4,104 (3,573-4,714)	75.5 (65.7-86.7)	-74.4% (-78.6 to -69.4%)	5,915.3 (4,732.9-7,293.2)	-41.9% (-46.7 to -36.6%)	1.3% (1.2-1.4%)	85.3% (77.4 to 90.8%)	45.2% (29.1 to 61.3%)	78.0% (52.5 to 91.1%)
Swaziland	219 (155-299)	151.0 (106.5-206.0)	-39.0% (-56.7 to -12.7%)	11,236.3 (8,906.4-14,084.3)	-5.7% (-16.5 to 7.0%)	1.3% (1.2-1.5%)	82.1% (74.3 to 88.1%)	48.0% (31.9 to 63.8%)	73.9% (42.0 to 91.0%)
Zimbabwe	5,398 (4,288-6,857)	245.1 (194.7-311.4)	4.5% (-24.0 to 43.2%)	9,782.9 (8,283.3-11,420.4)	-12.9% (-24.5 to -1.3%)	2.5% (2.4-2.7%)	88.9% (83.6 to 93.0%)	57.9% (40.0 to 74.1%)	76.1% (48.9 to 90.9%)
Western Sub-Saharan Africa	243,122 (198,471-290,155)	338.7 (276.5-404.3)	-60.2% (-67.1 to -50.7%)	8,408.3 (6,875.4-10,219.7)	-37.7% (-42.7 to -31.7%)	4.0% (4.0-4.0%)	94.5% (91.7 to 96.5%)	69.3% (49.0 to 84.9%)	83.8% (63.7 to 93.8%)
Benin	4,016 (2,902-5,538)	210.4 (152.0-290.2)	-67.6% (-77.1 to -55.2%)	6,726.6 (5,371.9-8,273.8)	-48.2% (-55.4 to -39.7%)	3.1% (2.8-3.5%)	94.2% (90.5 to 96.5%)	66.7% (45.2 to 83.2%)	83.1% (58.5 to 94.5%)
Burkina Faso	11,993 (8,618-16,061)	321.2 (230.8-430.2)	-56.3% (-68.7 to -38.8%)	8,954.0 (7,092.7-11,256.2)	-33.9% (-42.2 to -23.7%)	3.6% (3.3-3.8%)	96.2% (93.7 to 97.9%)	62.9% (44.0 to 78.3%)	86.0% (67.3 to 94.9%)
Cameroon	6,457 (4,604-8,966)	157.0 (111.9-217.9)	-62.9% (-74.5 to -46.1%)	7,996.1 (6,404.9-9,899.1)	-28.5% (-36.2 to -19.8%)	2.0% (1.7-2.2%)	91.1% (86.1 to 94.8%)	58.9% (35.3 to 78.1%)	79.6% (52.4 to 92.8%)
Cape Verde	19 (13-28)	38.6 (25.6-56.1)	-68.7% (-80.6 to -50.3%)	5,758.6 (4,446.7-7,235.2)	-22.1% (-31.7 to -12.4%)	0.7% (0.6-0.8%)	83.9% (75.2 to 90.7%)	63.1% (48.8 to 76.0%)	74.5% (47.6 to 89.0%)
Chad	14,330 (10,890-18,202)	467.5 (355.3-593.8)	-42.7% (-57.8 to -20.7%)	13,186.2 (10,722.8-16,410.6)	-12.4% (-22.1 to -0.1%)	3.5% (3.3-3.6%)	96.3% (94.0 to 97.8%)	77.4% (57.1 to 90.2%)	88.5% (68.2 to 96.6%)

Location	Deaths (95% UI)	Mortality rate per 100,000 (95% UI)	Percent change mortality rate 1990-2017 (95% UI)	Incidence per 100,000 (95% UI)	Percent change incidence 1990 to 2017 (95% UI)	Case fatality ratio (95% UI)	Attributable fraction for all risks (95% UI)	Attributable fraction for prevention-associated risks (95% UI)	Attributable fraction for protection-associated risks (95% UI)
Cote d'Ivoire	8,646 (6,343-11,555)	223.4 (163.9-298.5)	-50.1% (-65.0 to -29.5%)	8,338.2 (6,801.6-10,212.1)	-22.5% (-31.1 to -13.2%)	2.7% (2.4-2.9%)	92.7% (88.7 to 95.7%)	63.8% (45.3 to 79.4%)	80.0% (54.0 to 92.9%)
The Gambia	392 (278-543)	123.1 (87.1-170.2)	-73.3% (-81.4 to -61.9%)	8,290.0 (6,763.9-10,177.8)	-39.3% (-47.7 to -29.9%)	1.5% (1.3-1.7%)	94.9% (91.4 to 97.3%)	63.6% (46.1 to 78.5%)	80.3% (59.7 to 91.6%)
Ghana	3,664 (2,590-5,153)	90.3 (63.8-127.0)	-67.2% (-77.3 to -52.8%)	6,969.4 (5,572.1-8,598.3)	-33.1% (-41.6 to -23.9%)	1.3% (1.1-1.5%)	91.8% (87.4 to 95.1%)	52.4% (33.6 to 71.2%)	79.8% (55.8 to 92.4%)
Guinea	6,643 (5,043-8,563)	337.9 (256.5-435.5)	-72.0% (-79.3 to -61.1%)	11,626.5 (9,396.8-14,494.0)	-37.8% (-45.3 to -29.1%)	2.9% (2.7-3.0%)	93.9% (90.0 to 96.5%)	77.3% (60.4 to 88.6%)	82.8% (58.8 to 94.0%)
Guinea-Bissau	458 (340-619)	149.5 (111.2-202.1)	-74.8% (-83.1 to -63.4%)	7,609.0 (6,133.0-9,357.2)	-40.5% (-47.1 to -32.4%)	2.0% (1.8-2.2%)	93.5% (89.4 to 96.1%)	66.9% (48.8 to 81.5%)	80.0% (55.5 to 92.4%)
Liberia	1,038 (713-1,484)	146.8 (100.8-209.8)	-85.0% (-90.1 to -78.1%)	10,357.2 (8,186.1-13,091.4)	-40.5% (-47.9 to -32.2%)	1.4% (1.2-1.6%)	93.1% (89.1 to 96.0%)	69.0% (51.3 to 82.9%)	80.5% (54.5 to 93.2%)
Mali	8,885 (6,242-12,064)	237.9 (167.1-323.0)	-59.7% (-72.2 to -42.3%)	6,432.9 (5,120.5-7,913.3)	-42.7% (-49.6 to -34.1%)	3.7% (3.3-4.1%)	95.4% (92.4 to 97.4%)	71.9% (51.8 to 86.5%)	82.4% (62.2 to 93.3%)
Mauritania	661 (464-926)	115.5 (81.2-161.9)	-63.0% (-74.9 to -46.4%)	7,455.4 (5,691.6-9,512.8)	-27.3% (-36.6 to -17.2%)	1.5% (1.4-1.7%)	96.4% (94.2 to 97.7%)	57.6% (33.8 to 77.8%)	86.4% (70.3 to 94.9%)
Niger	14,402 (10,090-19,728)	329.7 (231.0-451.6)	-75.6% (-82.7 to -65.5%)	10,415.8 (8,572.5-12,645.2)	-46.0% (-52.7 to -38.6%)	3.2% (2.7-3.6%)	97.2% (95.4 to 98.4%)	68.8% (44.8 to 85.7%)	89.0% (70.3 to 96.8%)

Location	Deaths (95% UI)	Mortality rate per 100,000 (95% UI)	Percent change mortality rate 1990-2017 (95% UI)	Incidence per 100,000 (95% UI)	Percent change incidence 1990 to 2017 (95% UI)	Case fatality ratio (95% UI)	Attributable fraction for all risks (95% UI)	Attributable fraction for prevention-associated risks (95% UI)	Attributable fraction for protection-associated risks (95% UI)
Nigeria	153,069 (115,332-196,193)	444.4 (334.8-569.6)	-59.8% (-69.7 to -46.0%)	7,708.1 (6,213.1-9,474.7)	-43.2% (-49.3 to -36.3%)	5.8% (5.4-6.0%)	94.3% (91.4 to 96.3%)	70.7% (44.5 to 88.3%)	83.5% (63.0 to 93.8%)
Sao Tome and Principe	19 (14-25)	75.4 (55.6-100.5)	-80.6% (-86.9 to -71.5%)	8,221.7 (6,460.5-10,377.6)	-43.6% (-51.9 to -34.6%)	0.9% (0.9-1.0%)	89.9% (84.3 to 94.0%)	47.7% (31.7 to 63.5%)	77.1% (52.4 to 90.4%)
Senegal	3,099 (2,282-4,153)	136.7 (100.7-183.2)	-67.9% (-77.0 to -55.2%)	11,495.7 (9,447.9-13,836.7)	-22.0% (-32.1 to -9.2%)	1.2% (1.1-1.3%)	93.3% (88.9 to 96.2%)	58.2% (40.4 to 74.1%)	80.7% (57.9 to 92.2%)
Sierra Leone	3,981 (2,880-5,350)	333.1 (241.0-447.7)	-71.4% (-79.6 to -59.6%)	11,782.8 (9,369.2-14,596.5)	-35.0% (-43.4 to -25.1%)	2.8% (2.6-3.1%)	94.0% (90.6 to 96.5%)	64.2% (46.3 to 79.2%)	83.9% (58.7 to 94.8%)
Togo	1,347 (960-1,849)	126.0 (89.7-172.8)	-72.2% (-80.7 to -60.6%)	7,744.2 (6,238.6-9,461.0)	-38.9% (-45.8 to -30.7%)	1.6% (1.4-1.8%)	94.2% (90.2 to 96.6%)	60.9% (42.2 to 77.1%)	81.9% (59.8 to 92.8%)

Supplementary Results Table 2. The percent change in deaths due to LRI between 1990 and 2017 due to changes in exposure to risk factors is shown for each country. These are the results shown in the Manuscript Figure 4 with 95% uncertainty intervals presented in parentheses. The countries are ordered to match the ordering of the panels in the manuscript.

Location Name	Ambient pollution	Household air pollution	Low Hib vaccine coverage	Low pneumococcal vaccine coverage	No handwashing	Secondhand smoke	Zinc deficiency	Breastfeeding	Low antibiotic coverage	Low birth weight and short gestation	Stunting	Underweight	Wasting	All risk factors
Global	4.1% (2.7 to 6.2%)	-8.4% (-9.2 to -6.8%)	-11.4% (-24.5 to 0.0%)	-6.3% (-6.3 to -6.1%)	0.7% (0.5 to 0.9%)	-3.7% (-5.0 to -2.5%)	-0.6% (-2.5 to 0.0%)	-0.5% (-0.9 to -0.2%)	2.3% (0.9 to 3.9%)	7.8% (7.2 to 8.4%)	-3.8% (-7.3 to -0.8%)	-4.0% (-5.8 to -2.8%)	-5.5% (-6.7 to -4.5%)	-12.2% (-13.1 to -11.6%)
**High mortality, high incidence**														
North Korea	-0.4% (-0.9 to -0.5%)	-3.1% (-4.2 to -1.8%)	-19.4% (-43.8 to 0.0%)	6.6% (3.3 to 9.8%)	-0.2% (-0.3 to -0.2%)	-3.0% (-3.6 to -2.3%)	0.2% (0.0 to 0.9%)	-0.1% (-0.1 to -0.2%)	2.0% (0.6 to 3.2%)	0.2% (0.4 to 0.2%)	-4.9% (-10.5 to -0.5%)	-4.2% (-8.7 to -2.5%)	-32.1% (-38.1 to -24.6%)	-50.5% (-54.5 to -45.7%)
El Salvador	3.9% (2.0 to 5.5%)	-14.6% (-18.2 to -10.7%)	-12.8% (-26.2 to 0.0%)	-15.9% (-17.6 to -13.8%)	-2.2% (-2.9 to -1.4%)	-0.2% (-0.1 to -0.2%)	-0.2% (-0.8 to 0.0%)	-1.3% (-2.3 to -0.5%)	1.4% (0.4 to 3.2%)	1.7% (1.8 to 1.5%)	-4.2% (-10.2 to -0.5%)	-2.6% (-4.8 to -1.9%)	-16.1% (-19.3 to -10.9%)	-37.6% (-40.8 to -34.6%)
Iraq	1.3% (0.8 to 2.1%)	-1.7% (-3.2 to -0.8%)	-15.5% (-31.5 to 0.0%)	-0.5% (3.7 to -3.8%)	-0.6% (-0.8 to -0.3%)	-2.0% (-2.5 to -1.5%)	0.8% (0.0 to 4.4%)	-0.5% (-1.3 to 0.0%)	2.4% (1.0 to 4.7%)	3.0% (3.4 to 2.6%)	-5.3% (-12.4 to -1.0%)	-5.5% (-10.1 to -3.9%)	-10.7% (-10.9 to -7.7%)	-23.7% (-21.1 to -26.1%)
Moldova	0.6% (-0.3 to 1.4%)	-8.1% (-10.6 to -5.8%)	-8.4% (-16.8 to 0.0%)	-4.2% (-3.2 to -4.6%)	-1.1% (-1.6 to -0.6%)	-1.8% (-2.3 to -1.2%)	0.0% (0.0 to 0.0%)	0.0% (-0.3 to 0.0%)	1.6% (0.8 to 2.7%)	2.4% (2.2 to 2.0%)	-1.3% (-3.3 to 0.0%)	-0.7% (-1.3 to -0.4%)	-8.2% (-11.7 to -4.6%)	-17.9% (-21.3 to -15.2%)
Honduras	2.1% (1.4 to 2.6%)	-14.5% (-18.4 to -10.6%)	-14.7% (-29.4 to 0.0%)	-16.4% (-17.8 to -14.3%)	-0.8% (-1.3 to -0.5%)	-2.5% (-3.9 to -1.5%)	-0.2% (-1.0 to 0.0%)	-0.3% (-1.1 to 0.3%)	0.0% (0.2 to -1.7%)	2.1% (2.4 to 1.8%)	-6.2% (-14.2 to -1.4%)	-4.0% (-7.1 to -3.0%)	-12.3% (-14.7 to -8.2%)	-33.7% (-35.6 to -31.2%)
Iran	3.5% (2.6 to 4.2%)	-3.5% (-5.4 to -2.0%)	-13.1% (-26.4 to 0.0%)	4.7% (2.3 to 7.2%)	-0.8% (-1.1 to -0.5%)	-0.8% (-1.1 to -0.5%)	-0.2% (-0.7 to 0.0%)	-0.3% (-0.8 to 0.2%)	2.7% (1.1 to 4.5%)	5.2% (5.2 to 5.1%)	-4.0% (-9.7 to -0.6%)	-4.4% (-8.0 to -3.0%)	-17.8% (-19.0 to -13.8%)	-21.7% (-22.2 to -20.4%)
Solomon Islands	0.7% (0.3 to 1.9%)	-11.5% (-11.3 to -8.7%)	-18.9% (-39.4 to 0.0%)	-18.6% (-19.0 to -17.1%)	-2.4% (-3.3 to -1.5%)	-1.6% (-2.1 to -1.1%)	-1.2% (-4.8 to 0.0%)	-0.1% (-0.2 to 0.0%)	2.6% (1.1 to 4.8%)	1.6% (1.4 to 1.4%)	-3.0% (-6.7 to -0.5%)	-3.4% (-6.6 to -2.2%)	-14.0% (-18.1 to -9.4%)	-36.5% (-41.8 to -33.7%)
Ecuador	1.5% (0.0 to 2.6%)	-8.5% (-11.4 to -5.6%)	-13.4% (-27.6 to 0.0%)	-15.9% (-15.7 to -14.9%)	-1.6% (-2.2 to -1.1%)	-1.9% (-2.5 to -1.3%)	-0.9% (-4.4 to 0.0%)	-0.8% (-1.4 to -0.5%)	1.7% (0.7 to 2.6%)	1.8% (1.7 to 1.8%)	-4.7% (-10.5 to -0.9%)	-3.4% (-6.3 to -2.6%)	-11.9% (-13.9 to -7.3%)	-31.6% (-33.6 to -30.3%)

Location Name	Ambient pollution	Household air pollution	Low Hib vaccine coverage	Low pneumococcal vaccine coverage	No handwashing	Secondhand smoke	Zinc deficiency	Breastfeeding	Low antibiotic coverage	Low birth weight and short gestation	Stunting	Underweight	Wasting	All risk factors
Dominican Republic	2.5% (0.4 to 4.9%)	-12.6% (-15.5 to -9.8%)	-9.9% (-20.2 to 0.0%)	-5.5% (-4.5 to -6.0%)	-0.8% (-1.0 to -0.5%)	-3.7% (-5.0 to -2.4%)	-1.0% (-3.9 to 0.0%)	0.2% (0.1 to 0.3%)	1.2% (0.6 to 2.0%)	1.4% (1.2 to 1.7%)	-2.2% (-5.6 to -0.2%)	-1.6% (-3.1 to -1.1%)	-2.6% (-2.7 to -1.0%)	-21.2% (-22.0 to -20.9%)
Federated States of Micronesia	1.0% (0.2 to 2.4%)	-11.9% (-14.5 to -9.3%)	-5.6% (-11.5 to 0.0%)	-7.3% (-6.6 to -7.3%)	-1.6% (-2.2 to -1.0%)	-0.6% (-0.8 to -0.4%)	0.0% (-0.2 to 0.0%)	-0.3% (-0.7 to -0.1%)	1.9% (0.6 to 3.3%)	1.6% (1.6 to 1.3%)	-1.7% (-4.3 to -0.1%)	-1.8% (-3.4 to -1.1%)	-10.0% (-12.4 to -7.4%)	-25.5% (-27.5 to -24.2%)
Central African Republic	3.7% (0.9 to 9.9%)	-10.6% (-9.9 to -10.1%)	-9.1% (-20.2 to 0.0%)	-3.1% (-0.3 to -5.5%)	-0.2% (-0.1 to 0.0%)	-0.9% (-1.0 to -0.8%)	-2.2% (-9.2 to 0.0%)	-2.2% (-3.2 to -1.3%)	0.5% (0.1 to 1.4%)	1.2% (0.9 to 1.3%)	-2.7% (-4.1 to -1.2%)	-1.7% (-3.4 to -1.6%)	-9.6% (-8.5 to -9.2%)	-27.4% (-28.2 to -28.0%)
Papua New Guinea	0.7% (0.2 to 1.5%)	-14.1% (-14.8 to -10.0%)	-15.0% (-31.3 to 0.0%)	-5.2% (-2.4 to -7.5%)	-2.1% (-2.6 to -1.4%)	-0.2% (-0.4 to 0.0%)	-0.1% (-0.2 to 0.0%)	-0.4% (-0.9 to 0.1%)	1.5% (0.5 to 2.9%)	1.5% (1.0 to 2.2%)	-5.4% (-10.4 to -1.5%)	-5.5% (-11.7 to -3.2%)	-19.8% (-23.4 to -15.7%)	-48.2% (-51.3 to -45.2%)
Mexico	1.7% (0.6 to 2.8%)	-7.2% (-9.4 to -5.4%)	-12.5% (-25.2 to 0.0%)	-14.1% (-14.8 to -12.7%)	-0.7% (-1.0 to -0.4%)	-4.4% (-6.1 to -2.6%)	0.1% (0.0 to 0.3%)	0.5% (0.3 to 0.7%)	1.1% (0.5 to 1.8%)	2.2% (2.3 to 2.2%)	-2.5% (-6.2 to -0.4%)	-1.5% (-2.9 to -1.1%)	-13.0% (-17.0 to -8.9%)	-24.1% (-26.5 to -21.7%)
Kiribati	0.3% (0.1 to 0.7%)	-9.6% (-10.9 to -7.0%)	-11.4% (-23.5 to 0.0%)	-13.0% (-12.8 to -12.4%)	-2.1% (-3.1 to -1.4%)	0.5% (0.3 to 0.5%)	0.0% (-0.4 to 0.0%)	-0.4% (-0.7 to -0.2%)	1.8% (0.8 to 2.8%)	0.8% (0.7 to 1.0%)	-3.4% (-8.0 to -0.6%)	-2.5% (-4.0 to -1.7%)	-12.6% (-13.5 to -10.8%)	-29.5% (-28.2 to -29.6%)
Vietnam	4.5% (1.9 to 6.9%)	-16.3% (-19.8 to -12.1%)	-11.6% (-23.3 to 0.0%)	7.1% (4.1 to 10.4%)	-0.6% (-0.8 to -0.4%)	0.7% (0.9 to 0.4%)	-0.8% (-3.3 to 0.0%)	-0.3% (-1.0 to 0.0%)	5.0% (1.7 to 9.0%)	2.9% (2.5 to 4.3%)	-6.1% (-13.4 to -1.2%)	-6.5% (-11.1 to -4.5%)	-13.7% (-15.6 to -10.5%)	-35.5% (-36.0 to -35.1%)
Romania	-0.2% (-0.2 to -0.7%)	-5.7% (-7.4 to -4.1%)	-10.8% (-21.3 to 0.0%)	2.1% (0.3 to 3.7%)	-0.5% (-0.8 to -0.3%)	-1.8% (-1.9 to -1.5%)	0.0% (0.1 to 0.0%)	0.0% (0.2 to -0.2%)	2.2% (0.9 to 3.5%)	0.5% (0.5 to 0.5%)	-1.0% (-2.3 to -0.1%)	-1.1% (-2.1 to -0.7%)	-9.2% (-9.5 to -6.0%)	-19.9% (-20.1 to -18.1%)
Nicaragua	2.3% (1.1 to 4.1%)	-16.3% (-19.4 to -13.1%)	-15.3% (-31.9 to 0.0%)	-19.6% (-22.8 to -16.1%)	-1.8% (-2.4 to -1.2%)	-0.6% (-0.4 to -0.5%)	-0.4% (-1.8 to 0.0%)	-0.5% (-1.2 to -0.1%)	2.7% (0.6 to 6.7%)	1.0% (1.0 to 0.8%)	-3.2% (-7.3 to -0.5%)	-1.9% (-3.7 to -1.5%)	-16.3% (-20.1 to -10.3%)	-36.5% (-39.7 to -33.4%)
Morocco	2.5% (1.2 to 3.5%)	-2.0% (-3.4 to -1.1%)	-12.7% (-25.5 to 0.0%)	-11.7% (-12.8 to -10.1%)	-2.2% (-2.8 to -1.4%)	-3.1% (-3.6 to -2.4%)	0.0% (-0.1 to 0.0%)	0.5% (0.5 to 0.4%)	2.6% (0.9 to 5.1%)	6.9% (8.1 to 5.4%)	-3.2% (-8.5 to -0.4%)	-1.9% (-3.6 to -1.2%)	-7.1% (-10.6 to -4.8%)	-10.2% (-12.4 to -8.8%)
Guatemala	4.2% (2.5 to 5.6%)	-20.1% (-23.8 to -15.1%)	-15.3% (-32.0 to 0.0%)	-20.9% (-22.0 to -18.9%)	-2.9% (-4.0 to -1.9%)	-1.0% (-1.0 to -0.8%)	-0.1% (-0.7 to 0.0%)	-0.3% (-1.5 to 0.5%)	1.4% (1.0 to -1.0%)	1.0% (1.0 to 0.8%)	-8.5% (-17.5 to -2.1%)	-8.2% (-14.6 to -5.8%)	-42.6% (-55.3 to -26.3%)	-85.3% (-95.1 to -75.2%)

Location Name	Ambient pollution	Household air pollution	Low Hib vaccine coverage	Low pneumococcal vaccine coverage	No handwashing	Secondhand smoke	Zinc deficiency	Breastfeeding	Low antibiotic coverage	Low birth weight and short gestation	Stunting	Underweight	Wasting	All risk factors
Kenya	3.3% (2.5 to 3.8%)	-14.0% (-15.2 to -11.0%)	-16.4% (-34.9 to 0.0%)	-16.5% (-16.8 to -15.2%)	-0.4% (-0.6 to -0.3%)	-1.8% (-2.5 to -1.2%)	-0.1% (-0.4 to 0.0%)	-1.5% (-3.0 to -0.4%)	-0.2% (-0.4 to -0.1%)	5.9% (5.6 to 6.0%)	-7.1% (-14.8 to -1.0%)	-6.8% (-11.9 to -4.6%)	-16.2% (-21.3 to -11.0%)	-39.5% (-42.5 to -37.2%)
Uganda	5.7% (2.5 to 9.4%)	-16.5% (-16.1 to -14.3%)	-17.9% (-37.0 to 0.0%)	-20.3% (-21.2 to -18.3%)	-0.6% (-0.8 to -0.4%)	-0.4% (-0.8 to -0.1%)	-0.5% (-2.6 to 0.0%)	-0.3% (-1.3 to 0.4%)	1.7% (0.2 to 5.7%)	1.7% (1.7 to 1.2%)	-10.6% (-22.8 to -2.0%)	-6.8% (-13.2 to -4.3%)	-13.7% (-18.9 to -8.9%)	-42.1% (-45.8 to -38.8%)
South Sudan	2.3% (0.8 to 4.1%)	-8.0% (-8.2 to -7.1%)	-10.8% (-22.4 to 0.0%)	8.4% (3.9 to 12.9%)	-0.1% (-0.1 to 0.0%)	0.5% (0.5 to 0.1%)	0.0% (-0.1 to 0.0%)	-2.0% (-4.2 to -0.4%)	1.8% (0.5 to 4.5%)	1.9% (2.0 to 2.1%)	-7.4% (-15.5 to -2.2%)	-9.4% (-16.3 to -5.9%)	-14.9% (-18.0 to -10.7%)	-41.0% (-39.6 to -40.4%)
China	6.6% (5.1 to 8.3%)	-13.7% (-16.2 to -10.5%)	-7.3% (-13.3 to 0.0%)	-7.4% (-3.5 to -9.0%)	-1.9% (-2.7 to -1.2%)	-1.8% (-2.3 to -1.4%)	-0.3% (-1.3 to 0.0%)	-0.6% (-1.2 to -0.2%)	2.4% (0.9 to 3.9%)	3.0% (3.0 to 3.1%)	-3.6% (-9.2 to -0.2%)	-3.2% (-6.0 to -2.0%)	-12.9% (-17.2 to -8.0%)	-26.2% (-29.8 to -23.0%)
Turkey	4.2% (3.3 to 5.1%)	-4.4% (-6.8 to -2.6%)	-11.3% (-21.9 to 0.0%)	-13.0% (-14.5 to -11.0%)	-1.1% (-1.6 to -0.7%)	-6.0% (-7.1 to -4.9%)	-0.1% (-0.2 to 0.0%)	-0.7% (-1.6 to -0.1%)	0.9% (0.3 to 1.5%)	6.8% (6.5 to 5.5%)	-2.5% (-6.5 to -0.2%)	-1.9% (-3.5 to -1.4%)	-5.1% (-7.1 to -3.0%)	-12.7% (-14.5 to -10.7%)
Senegal	2.7% (1.0 to 5.5%)	-8.4% (-8.4 to -6.2%)	-18.9% (-40.4 to 0.0%)	-21.5% (-23.8 to -18.4%)	-0.5% (-0.7 to -0.3%)	-2.3% (-3.2 to -1.4%)	-1.3% (-5.5 to 0.0%)	-1.8% (-3.0 to -0.7%)	-0.5% (-2.5 to 0.0%)	2.3% (2.4 to 2.3%)	-5.7% (-13.2 to -0.8%)	-4.2% (-7.2 to -2.8%)	-6.0% (-7.1 to -5.3%)	-27.9% (-29.3 to -27.5%)
Pakistan	9.2% (6.0 to 12.8%)	-20.7% (-24.5 to -15.1%)	-15.8% (-32.8 to 0.0%)	-13.8% (-13.6 to -13.0%)	-1.8% (-2.4 to -1.1%)	-3.7% (-5.0 to -2.6%)	-0.8% (-2.8 to 0.0%)	-0.2% (-1.1 to 0.4%)	2.4% (1.1 to 3.8%)	19.1% (22.6 to 17.2%)	-6.5% (-11.4 to -1.9%)	-3.6% (-6.6 to -2.2%)	4.5% (2.5 to 8.6%)	-13.7% (-11.9 to -14.1%)
Democratic Republic of the Congo	2.3% (0.3 to 4.8%)	-7.8% (-7.7 to -5.7%)	-18.1% (-38.4 to 0.0%)	-18.3% (-18.3 to -17.2%)	0.0% (0.0 to 0.0%)	-1.9% (-3.0 to -1.2%)	-0.9% (-3.0 to 0.0%)	-1.4% (-2.4 to -0.7%)	2.0% (0.7 to 2.8%)	4.4% (4.1 to 3.5%)	-2.4% (-3.5 to -1.2%)	-4.0% (-6.9 to -3.0%)	5.0% (3.6 to 9.9%)	-7.5% (-5.7 to -10.6%)
Burundi	1.0% (0.5 to 0.7%)	-4.6% (-5.3 to -3.2%)	-23.5% (-51.0 to 0.0%)	-27.2% (-30.5 to -23.1%)	-0.1% (-0.1 to -0.1%)	-1.2% (-1.8 to -0.8%)	3.9% (0.0 to 18.4%)	-0.6% (-1.3 to 0.0%)	9.3% (3.8 to 15.7%)	3.7% (4.7 to 3.1%)	0.4% (-0.4 to 3.0%)	-2.3% (-3.7 to -1.8%)	-8.1% (-5.2 to -6.7%)	-8.9% (-0.1 to -10.3%)
Albania	1.2% (0.5 to 1.9%)	-7.9% (-9.4 to -6.1%)	-10.9% (-21.6 to 0.0%)	-12.8% (-14.8 to -10.6%)	-0.3% (-0.5 to -0.2%)	-0.7% (-0.7 to -0.5%)	-0.2% (-0.7 to 0.0%)	-0.3% (-0.9 to 0.1%)	2.5% (1.0 to 4.0%)	1.6% (1.6 to 1.5%)	-3.6% (-8.2 to -0.3%)	-2.0% (-3.8 to -1.3%)	-7.9% (-9.0 to -6.0%)	-19.5% (-20.4 to -19.1%)
Philippines	0.3% (0.2 to 0.4%)	-5.3% (-6.7 to -4.3%)	-13.7% (-28.3 to 0.0%)	-10.9% (-10.7 to -10.6%)	-0.8% (-1.1 to -0.5%)	-3.2% (-4.0 to -2.7%)	-1.2% (-5.2 to 0.0%)	-0.1% (-0.2 to -0.1%)	-0.8% (-1.0 to -0.3%)	1.4% (2.2 to 1.0%)	-3.5% (-8.0 to -0.7%)	-3.9% (-6.4 to -2.7%)	-6.9% (-5.4 to -5.4%)	-25.6% (-26.7 to -25.1%)

Location Name	Ambient pollution	House-hold air pollution	Low Hib vaccine coverage	Low pneumo-coccal vaccine coverage	No hand-washing	Second-hand smoke	Zinc deficiency	Breast-feeding	Low antibiotic coverage	Low birth weight and short gestation	Stunting	Under-weight	Wasting	All risk factors
Sao Tome and Principe	4.0% (1.9 to 7.1%)	-17.3% (-20.0 to -13.7%)	-15.3% (-31.5 to 0.0%)	-18.7% (-20.7 to -15.9%)	-2.1% (-2.9 to -1.3%)	-0.6% (-1.0 to -0.3%)	-0.5% (-2.9 to 0.0%)	-0.7% (-1.5 to -0.1%)	1.4% (0.7 to 2.8%)	1.1% (1.2 to 0.7%)	-6.4% (-14.1 to -0.9%)	-4.3% (-8.1 to -2.9%)	-7.5% (-6.4 to -5.8%)	-34.8% (-35.3 to -33.4%)
Madagascar	1.1% (0.6 to 1.3%)	-6.0% (-6.6 to -2.8%)	-22.2% (-47.5 to 0.0%)	-23.7% (-24.8 to -21.1%)	0.1% (0.1 to 0.1%)	-3.2% (-4.4 to -2.1%)	0.2% (0.0 to 1.0%)	-1.5% (-2.0 to -0.7%)	-0.2% (-0.8 to 0.1%)	0.6% (0.1 to 1.5%)	-3.4% (-6.0 to -1.2%)	-3.1% (-4.1 to -2.5%)	-23.6% (-21.6 to -18.6%)	-41.1% (-37.4 to -42.7%)
Sudan	8.8% (3.3 to 15.7%)	-20.3% (-23.3 to -16.8%)	-16.0% (-32.6 to 0.0%)	-18.9% (-20.2 to -16.8%)	-0.4% (-0.8 to -0.3%)	-0.4% (-0.7 to 0.0%)	-1.8% (-8.3 to 0.0%)	-2.4% (-4.8 to -0.5%)	7.0% (2.9 to 11.9%)	5.3% (4.0 to 5.6%)	-7.2% (-15.7 to -1.2%)	-7.2% (-11.7 to -4.6%)	-15.6% (-13.5 to -15.2%)	-42.1% (-42.1 to -43.4%)
Indonesia	2.1% (1.5 to 2.9%)	-12.7% (-15.4 to -10.1%)	-9.0% (-18.0 to 0.0%)	4.1% (1.8 to 6.5%)	-0.3% (-0.4 to -0.2%)	-0.7% (-0.7 to -0.9%)	-0.4% (-1.9 to 0.0%)	-0.2% (-0.5 to 0.0%)	2.6% (1.1 to 4.2%)	1.5% (1.6 to 1.5%)	-3.9% (-8.2 to -0.7%)	-3.9% (-6.4 to -2.6%)	-10.0% (-11.3 to -8.5%)	-27.5% (-28.5 to -26.9%)
Djibouti	11.1% (4.0 to 23.1%)	-26.7% (-32.9 to -21.0%)	-10.5% (-21.5 to 0.0%)	-10.6% (-9.6 to -10.8%)	-0.7% (-1.0 to -0.4%)	-0.9% (-0.7 to -0.8%)	-1.1% (-4.6 to 0.0%)	-0.1% (-0.4 to 0.3%)	-0.6% (-0.8 to -0.1%)	2.1% (2.8 to 1.6%)	-4.0% (-9.0 to -0.3%)	-7.4% (-13.2 to -4.6%)	-15.4% (-17.5 to -13.2%)	-46.2% (-47.9 to -45.7%)
India	9.7% (6.7 to 11.7%)	-14.7% (-16.7 to -11.5%)	-12.3% (-26.0 to 0.0%)	1.1% (-0.8 to 3.4%)	-1.4% (-1.9 to -0.9%)	-1.6% (-2.0 to -0.9%)	-0.4% (-1.8 to 0.0%)	-0.6% (-1.3 to -0.1%)	3.9% (1.6 to 6.3%)	7.0% (7.4 to 6.9%)	-7.4% (-15.1 to -1.6%)	-9.1% (-14.7 to -6.4%)	-11.4% (-13.1 to -9.6%)	-30.5% (-30.4 to -31.1%)
Yemen	12.0% (4.7 to 23.5%)	-25.3% (-29.6 to -18.9%)	-10.4% (-21.1 to 0.0%)	-11.0% (-10.3 to -10.8%)	-2.0% (-2.8 to -1.3%)	-2.2% (-2.7 to -1.8%)	-0.3% (-1.3 to 0.0%)	0.2% (0.9 to -0.9%)	4.9% (1.9 to 9.4%)	0.6% (0.6 to 0.5%)	-8.5% (-18.2 to -2.3%)	-4.9% (-8.9 to -2.7%)	-4.4% (-5.3 to -4.1%)	-38.4% (-35.4 to -41.2%)
Chad	4.6% (1.4 to 10.0%)	-13.5% (-11.3 to -13.2%)	-13.8% (-28.0 to 0.0%)	10.3% (4.8 to 15.9%)	-0.6% (-0.7 to -0.4%)	0.2% (0.2 to 0.0%)	0.0% (0.0 to 0.0%)	0.3% (0.6 to -0.2%)	1.9% (0.8 to 3.2%)	3.9% (2.6 to 4.0%)	-6.6% (-14.0 to -1.4%)	-7.3% (-13.8 to -4.5%)	-25.3% (-23.0 to -24.2%)	-49.0% (-43.6 to -50.7%)
Haiti	1.0% (0.4 to 2.1%)	-11.5% (-13.5 to -8.6%)	-11.8% (-24.0 to 0.0%)	9.4% (5.3 to 13.6%)	0.1% (0.0 to 0.1%)	-1.2% (-1.8 to -0.8%)	-0.1% (-0.2 to 0.0%)	-2.0% (-2.9 to -1.2%)	5.2% (2.0 to 8.8%)	0.7% (-1.4 to 0.9%)	-6.5% (-14.7 to -0.8%)	-5.8% (-10.6 to -3.9%)	-7.9% (-10.0 to -5.8%)	-34.0% (-35.3 to -35.2%)
Comoros	1.9% (0.9 to 3.2%)	-12.7% (-14.8 to -9.2%)	-13.2% (-27.0 to 0.0%)	8.0% (4.7 to 11.6%)	-0.7% (-1.0 to -0.5%)	-1.1% (-1.7 to -0.7%)	0.1% (0.0 to 0.6%)	0.1% (0.5 to -0.6%)	1.0% (0.5 to 1.3%)	3.7% (3.9 to 3.6%)	-5.2% (-10.9 to -1.3%)	-4.8% (-8.6 to -3.0%)	-11.9% (-14.2 to -9.2%)	-30.2% (-30.7 to -29.3%)
Peru	3.3% (1.0 to 7.3%)	-12.7% (-16.4 to -9.4%)	-10.9% (-21.6 to 0.0%)	-11.0% (-11.3 to -10.2%)	-1.4% (-1.9 to -0.9%)	-0.4% (-0.4 to -0.3%)	-1.5% (-6.3 to 0.0%)	-2.0% (-3.1 to -0.9%)	1.5% (0.7 to 2.5%)	1.7% (1.9 to 1.4%)	-7.4% (-17.4 to -1.1%)	-1.7% (-3.4 to -1.1%)	-10.7% (-16.3 to -5.4%)	-31.4% (-36.3 to -27.8%)

Location Name	Ambient pollution	House-hold air pollution	Low Hib vaccine coverage	Low pneumo-coccal vaccine coverage	No hand-washing	Second-hand smoke	Zinc deficiency	Breast-feeding	Low antibiotic coverage	Low birth weight and short gestation	Stunting	Under-weight	Wasting	All risk factors
Malawi	1.4% (0.8 to 1.9%)	-8.7% (-9.0 to - 6.9%)	-17.0% (-35.3 to 0.0%)	-19.4% (-21.0 to - 16.8%)	0.1% (0.1 to 0.1%)	-1.6% (-2.2 to - 0.9%)	-0.1% (-0.5 to 0.0%)	-2.7% (-4.0 to - 1.4%)	-0.7% (-2.2 to - 0.2%)	4.5% (4.9 to 3.6%)	-6.8% (-14.2 to - 1.4%)	-5.6% (-10.1 to - 3.7%)	-17.6% (-21.7 to -12.0%)	-37.4% (-40.6 to - 35.8%)
Tajikistan	4.0% (1.7 to 10.2%)	-12.5% (-16.2 to - 9.2%)	-19.8% (-42.7 to 0.0%)	6.8% (3.3 to 10.4%)	-0.1% (-0.3 to - 0.1%)	-5.1% (-7.5 to - 3.4%)	0.0% (0.0 to 0.0%)	-0.5% (-1.4 to 0.0%)	1.6% (0.6 to 2.9%)	-0.4% (-0.5 to - 0.4%)	-4.2% (-9.5 to - 0.4%)	-2.7% (-5.0 to - 1.8%)	70.5% (44.5 to 99.7%)	33.1% (17.4 to 50.6%)
Bhutan	7.6% (4.6 to 11.6%)	-18.6% (-22.2 to - 14.4%)	-13.8% (-28.1 to 0.0%)	8.4% (5.1 to 12.0%)	-3.4% (-4.6 to - 2.2%)	-1.3% (-2.2 to - 0.8%)	-0.4% (-1.8 to 0.0%)	-0.4% (-0.9 to 0.2%)	2.7% (1.0 to 3.8%)	7.2% (7.1 to 7.3%)	-6.0% (-12.6 to - 1.2%)	-4.9% (-8.7 to - 3.5%)	-8.4% (-8.5 to - 6.6%)	-28.9% (-28.5 to - 27.9%)
Rwanda	2.4% (1.0 to 5.0%)	-8.5% (-8.5 to - 7.1%)	-18.0% (-37.7 to 0.0%)	-21.6% (-24.7 to - 18.0%)	-0.1% (-0.2 to - 0.1%)	-0.6% (-0.9 to - 0.4%)	-0.5% (-2.4 to 0.0%)	-0.4% (-0.6 to - 0.2%)	7.9% (3.4 to 11.4%)	6.0% (6.2 to 4.0%)	-7.0% (-14.3 to - 1.6%)	-4.5% (-8.1 to - 3.2%)	-8.8% (-10.0 to -5.6%)	-22.3% (-22.6 to - 21.6%)
Turkmeni stan	-1.1% (-3.5 to - 1.2%)	-0.4% (-0.7 to - 0.2%)	-13.7% (-28.0 to 0.0%)	7.1% (4.0 to 10.5%)	-0.8% (-1.2 to - 0.6%)	-1.9% (-2.3 to - 1.5%)	-0.8% (-3.7 to 0.0%)	-1.4% (-2.5 to - 0.4%)	0.3% (0.2 to 0.1%)	1.4% (1.4 to 1.6%)	-4.4% (-11.4 to - 0.6%)	-3.1% (-6.0 to - 2.1%)	-15.1% (-18.6 to -10.4%)	-26.8% (-29.8 to - 24.7%)
Egypt	5.9% (2.9 to 7.5%)	-3.4% (-5.6 to - 1.8%)	-15.9% (-32.3 to 0.0%)	10.3% (6.2 to 14.6%)	-1.4% (-1.9 to - 0.9%)	-3.6% (-4.6 to - 2.8%)	0.0% (0.2 to 0.0%)	0.2% (0.0 to 0.4%)	1.7% (0.7 to 2.7%)	7.2% (7.5 to 6.8%)	-1.5% (-3.9 to - 0.3%)	-1.3% (-2.6 to - 1.0%)	-7.7% (-7.7 to - 6.0%)	-8.7% (-7.2 to - 9.5%)
Tanzania	2.3% (1.2 to 5.9%)	-10.9% (-11.7 to - 9.2%)	-19.7% (-41.2 to 0.0%)	-23.7% (-26.0 to - 20.4%)	-0.3% (-0.3 to - 0.2%)	-0.8% (-1.6 to - 0.4%)	-0.2% (-1.0 to 0.0%)	-2.6% (-4.1 to - 1.3%)	1.8% (0.8 to 1.9%)	1.3% (1.3 to 0.9%)	-6.4% (-13.9 to - 1.3%)	-6.5% (-11.3 to - 4.5%)	-16.9% (-18.8 to -12.0%)	-44.1% (-44.8 to - 41.9%)
Eritrea	5.2% (1.8 to 10.4%)	-15.6% (-17.2 to - 12.0%)	-20.4% (-43.8 to 0.0%)	-25.2% (-29.7 to - 20.6%)	-0.3% (-0.6 to - 0.2%)	-1.5% (-2.0 to - 1.2%)	-0.8% (-3.3 to 0.0%)	-0.3% (-0.9 to 0.2%)	2.2% (0.5 to 5.6%)	2.9% (3.4 to 2.4%)	-8.0% (-14.1 to - 1.9%)	-10.6% (-17.2 to - 7.3%)	-25.8% (-25.0 to -22.7%)	-56.2% (-53.5 to - 57.4%)
Ethiopia	-0.1% (-1.0 to 0.2%)	17.3% (11.2 to 21.7%)	-9.9% (-20.0 to 0.0%)	-7.4% (-6.0 to - 7.9%)	-0.2% (-0.3 to - 0.1%)	-0.4% (-0.8 to - 0.2%)	0.0% (0.0 to 0.0%)	-0.5% (-1.6 to 0.2%)	7.4% (3.1 to 12.5%)	9.3% (6.9 to 10.6%)	-6.7% (-12.9 to - 1.7%)	-8.6% (-14.0 to - 5.9%)	-10.9% (-13.8 to -7.7%)	-0.3% (1.9 to - 4.4%)
Somalia	1.3% (0.4 to 2.8%)	-5.8% (-5.1 to - 5.9%)	-3.1% (-6.0 to 0.0%)	8.9% (4.4 to 13.6%)	-0.3% (-0.4 to - 0.3%)	-1.2% (-1.9 to - 0.6%)	0.6% (0.0 to 2.9%)	0.0% (0.2 to - 0.6%)	3.2% (1.7 to 5.1%)	3.1% (2.0 to 2.9%)	-4.7% (-8.8 to - 1.4%)	-4.9% (-8.0 to - 3.1%)	-24.4% (-26.2 to -20.1%)	-37.9% (-38.3 to - 39.6%)
Banglades h	5.1% (3.5 to 6.3%)	-11.8% (-13.4 to - 9.3%)	-12.8% (-26.1 to 0.0%)	-11.8% (-12.6 to - 10.3%)	-0.5% (-0.6 to - 0.4%)	-1.0% (-1.0 to - 0.8%)	-0.6% (-2.8 to 0.0%)	-0.6% (-1.6 to 0.0%)	7.4% (3.2 to 12.1%)	0.8% (0.8 to 0.9%)	-6.3% (-13.4 to - 1.3%)	-8.0% (-13.1 to - 5.2%)	-11.7% (-13.4 to -9.7%)	-32.2% (-33.2 to - 31.9%)

Location Name	Ambient pollution	House-hold air pollution	Low Hib vaccine coverage	Low pneumo-coccal vaccine coverage	No hand-washing	Second-hand smoke	Zinc deficiency	Breast-feeding	Low antibiotic coverage	Low birth weight and short gestation	Stunting	Under-weight	Wasting	All risk factors
Zambia	2.0% (1.3 to 2.2%)	-10.5% (-11.1 to -7.2%)	-21.1% (-44.6 to 0.0%)	-25.3% (-29.3 to -20.9%)	-0.5% (-0.7 to -0.4%)	-1.7% (-1.6 to -1.4%)	-0.1% (-0.4 to 0.0%)	-2.3% (-3.3 to -1.4%)	3.1% (1.2 to 5.0%)	1.1% (1.5 to 1.0%)	-5.4% (-11.6 to -1.1%)	-3.3% (-6.2 to -2.1%)	-2.1% (-0.7 to -2.3%)	-26.4% (-27.0 to -26.2%)
Bolivia	4.6% (1.4 to 9.7%)	-16.5% (-20.1 to -13.1%)	-14.0% (-27.7 to 0.0%)	-16.6% (-17.9 to -14.6%)	-1.8% (-2.4 to -1.2%)	-0.9% (-0.9 to -0.7%)	-0.4% (-1.7 to 0.0%)	-0.6% (-1.1 to -0.1%)	6.1% (2.6 to 10.6%)	1.6% (1.6 to 1.5%)	-6.8% (-15.2 to -1.1%)	-2.3% (-4.3 to -1.7%)	-11.1% (-14.3 to -6.7%)	-33.0% (-34.1 to -31.3%)
Mozambique	1.7% (0.9 to 3.0%)	-10.6% (-10.8 to -9.0%)	-18.7% (-38.6 to 0.0%)	-22.4% (-25.9 to -18.7%)	-0.3% (-0.5 to -0.2%)	-0.5% (-0.7 to -0.3%)	-3.3% (-10.8 to 0.0%)	-0.6% (-1.6 to 0.1%)	1.9% (1.0 to 3.7%)	2.0% (2.1 to 2.6%)	-9.8% (-21.2 to -1.8%)	-7.0% (-12.3 to -4.8%)	-29.6% (-35.6 to -21.6%)	-56.0% (-62.4 to -51.5%)
Myanmar	3.5% (2.0 to 5.3%)	-12.1% (-14.3 to -9.1%)	-9.0% (-18.0 to 0.0%)	-5.3% (-4.1 to -6.0%)	-0.6% (-0.7 to -0.4%)	-1.8% (-2.1 to -1.3%)	-1.7% (-6.1 to 0.0%)	-1.4% (-3.0 to -0.2%)	1.7% (0.4 to 4.2%)	2.2% (2.6 to 2.4%)	-5.2% (-12.7 to -0.7%)	-6.3% (-10.7 to -4.2%)	-14.1% (-15.9 to -10.5%)	-35.8% (-38.1 to -33.2%)
Nepal	9.2% (5.7 to 13.1%)	-14.1% (-15.8 to -10.8%)	-12.4% (-24.8 to 0.0%)	-5.8% (-3.8 to -6.5%)	-2.0% (-2.7 to -1.3%)	-1.2% (-2.3 to -0.4%)	-0.3% (-1.4 to 0.0%)	0.0% (-0.4 to 0.2%)	5.3% (2.2 to 8.9%)	7.8% (8.0 to 7.8%)	-6.6% (-16.3 to -0.6%)	-6.4% (-12.7 to -3.6%)	-12.0% (-15.5 to -8.1%)	-24.9% (-27.4 to -22.2%)
Equatorial Guinea	12.8% (5.9 to 22.2%)	-27.6% (-31.0 to -22.2%)	-5.6% (-10.7 to 0.0%)	8.6% (4.7 to 12.8%)	-1.9% (-2.6 to -1.2%)	-0.7% (-1.0 to -0.5%)	-5.2% (-16.6 to 0.0%)	0.1% (0.8 to -0.9%)	3.0% (0.9 to 7.7%)	11.8% (12.4 to 9.8%)	-14.3% (-28.9 to -2.5%)	-10.4% (-18.2 to -6.6%)	-20.7% (-27.4 to -14.2%)	-51.1% (-62.1 to -45.8%)
Cambodia	2.2% (1.2 to 3.9%)	-11.2% (-11.5 to -9.0%)	-12.9% (-26.0 to 0.0%)	-11.8% (-12.3 to -10.6%)	-2.4% (-3.1 to -1.6%)	-1.8% (-2.5 to -1.3%)	-0.7% (-2.8 to 0.0%)	-1.8% (-3.6 to -0.3%)	10.0% (4.2 to 16.7%)	2.5% (2.3 to 2.7%)	-7.3% (-14.2 to -1.8%)	-9.4% (-15.7 to -6.0%)	-15.3% (-19.0 to -12.8%)	-41.2% (-44.5 to -40.6%)
Timor-Leste	1.9% (0.8 to 3.7%)	-13.3% (-14.2 to -9.5%)	-13.4% (-27.1 to 0.0%)	2.7% (0.5 to 4.9%)	-1.5% (-2.1 to -1.0%)	-3.2% (-4.0 to -2.2%)	-0.4% (-1.9 to 0.0%)	-0.4% (-0.9 to 0.0%)	2.9% (0.8 to 6.3%)	0.6% (0.7 to 0.5%)	-7.9% (-15.6 to -1.9%)	-11.9% (-19.7 to -7.5%)	-16.4% (-18.1 to -13.8%)	-47.8% (-49.6 to -47.5%)
Angola	6.4% (3.0 to 11.2%)	-27.6% (-33.3 to -20.4%)	-8.3% (-17.5 to 0.0%)	-3.4% (-0.5 to -5.3%)	-0.4% (-0.6 to -0.3%)	1.0% (0.5 to 1.8%)	-5.5% (-18.9 to 0.0%)	-1.7% (-3.5 to -0.3%)	2.7% (0.8 to 6.7%)	9.5% (10.6 to 7.5%)	-10.2% (-18.3 to -2.2%)	-10.6% (-18.0 to -6.8%)	-32.6% (-39.4 to -25.0%)	-74.5% (-91.7 to -65.8%)
Liberia	1.2% (0.5 to 2.2%)	-7.6% (-7.7 to -7.3%)	-14.1% (-28.6 to 0.0%)	-16.2% (-15.9 to -15.2%)	0.0% (0.0 to 0.0%)	-0.5% (-1.1 to -0.1%)	1.4% (0.0 to 7.4%)	-2.2% (-3.6 to -1.0%)	1.8% (0.6 to 3.4%)	4.4% (4.7 to 3.5%)	-7.5% (-16.8 to -1.9%)	-4.0% (-7.1 to -2.5%)	-8.4% (-9.8 to -6.9%)	-24.2% (-22.7 to -23.4%)
Sierra Leone	1.8% (0.8 to 3.7%)	-9.7% (-9.4 to -7.7%)	-18.1% (-37.6 to 0.0%)	-21.6% (-23.0 to -19.1%)	-0.2% (-0.2 to -0.1%)	-2.3% (-3.2 to -1.5%)	-4.5% (-15.6 to 0.0%)	-0.8% (-1.8 to 0.1%)	1.4% (0.5 to 2.6%)	3.7% (3.6 to 4.2%)	-5.7% (-13.3 to -1.6%)	-5.8% (-9.9 to -4.0%)	-14.0% (-10.8 to -12.7%)	-38.8% (-42.0 to -37.5%)

Location Name	Ambient pollution	Household air pollution	Low Hib vaccine coverage	Low pneumococcal vaccine coverage	No handwashing	Secondhand smoke	Zinc deficiency	Breastfeeding	Low antibiotic coverage	Low birth weight and short gestation	Stunting	Underweight	Wasting	All risk factors
Mongolia	4.4% (1.4 to 5.0%)	-9.3% (-12.7 to -6.7%)	-13.9% (-27.9 to 0.0%)	-17.0% (-18.7 to -14.6%)	-0.1% (-0.2 to 0.0%)	0.2% (0.2 to 0.1%)	-0.1% (0.1 to 0.0%)	0.0% (0.2 to -0.2%)	1.7% (0.6 to 2.5%)	0.9% (0.7 to 1.0%)	-7.0% (-16.3 to -0.7%)	-4.1% (-8.0 to -2.7%)	-13.7% (-20.2 to -8.0%)	-28.1% (-31.3 to -26.2%)
Guinea	2.0% (0.7 to 5.1%)	-9.6% (-9.3 to -9.7%)	-8.9% (-18.4 to 0.0%)	6.9% (3.4 to 10.5%)	-0.2% (-0.2 to -0.1%)	-0.3% (-0.3 to -0.2%)	-0.5% (-2.5 to 0.0%)	-0.4% (-1.2 to 0.3%)	0.8% (0.1 to 1.8%)	5.7% (5.1 to 6.1%)	-4.1% (-9.7 to -0.8%)	-4.2% (-7.5 to -2.9%)	-24.9% (-25.5 to -19.8%)	-37.7% (-37.6 to -37.6%)
Afghanistan	5.5% (2.8 to 9.9%)	-19.3% (-19.5 to -17.3%)	-18.9% (-38.0 to 0.0%)	-15.2% (-13.8 to -15.6%)	-0.8% (-1.2 to -0.7%)	1.7% (1.3 to 1.7%)	3.1% (0.0 to 14.7%)	-0.3% (-1.0 to 0.1%)	8.5% (3.3 to 13.5%)	8.6% (10.7 to 7.8%)	-8.9% (-17.7 to -2.5%)	-9.5% (-17.6 to -5.7%)	-23.3% (-27.9 to -16.3%)	-44.0% (-39.9 to -44.4%)
Laos	2.7% (1.1 to 4.7%)	-13.0% (-14.6 to -10.3%)	-11.6% (-23.6 to 0.0%)	-12.4% (-12.4 to -11.6%)	-1.0% (-1.2 to -0.6%)	-2.0% (-2.4 to -1.6%)	-0.7% (-3.0 to 0.0%)	-1.0% (-2.4 to 0.2%)	1.0% (0.3 to 2.8%)	2.1% (2.2 to 2.1%)	-5.3% (-9.3 to -1.6%)	-9.7% (-16.9 to -6.6%)	-37.2% (-42.2 to -29.0%)	-66.7% (-72.3 to -62.4%)
Niger	3.1% (0.9 to 5.7%)	-7.4% (-5.7 to -6.2%)	-18.2% (-37.3 to 0.0%)	-19.8% (-20.0 to -18.6%)	-0.5% (-0.6 to -0.4%)	0.2% (0.2 to 0.0%)	-0.8% (-2.9 to 0.0%)	-0.7% (-1.4 to 0.0%)	-0.6% (-1.4 to -0.3%)	3.9% (4.7 to 3.8%)	-8.3% (-14.7 to -2.0%)	-7.9% (-13.6 to -5.3%)	-6.2% (-6.3 to -4.4%)	-26.1% (-24.5 to -25.8%)
**High mortality, low incidence**														
Zimbabwe	1.6% (0.9 to 1.9%)	-10.8% (-15.0 to -6.9%)	-25.8% (-57.6 to 0.0%)	-28.1% (-29.8 to -24.7%)	0.2% (0.0 to 0.1%)	-3.5% (-4.8 to -2.6%)	0.1% (0.0 to 0.3%)	-3.6% (-5.2 to -2.2%)	4.0% (1.8 to 6.3%)	2.8% (1.9 to 2.5%)	-2.9% (-9.8 to -0.5%)	-0.2% (-0.1 to -0.5%)	-0.1% (1.0 to -0.6%)	-19.4% (-20.5 to -18.1%)
Vanuatu	1.0% (0.3 to 1.1%)	-17.2% (-21.4 to -13.2%)	-18.1% (-38.5 to 0.0%)	10.4% (5.4 to 15.5%)	-1.9% (-2.7 to -1.2%)	-2.1% (-3.5 to -1.3%)	-0.2% (-1.0 to 0.0%)	-0.2% (-1.0 to 0.2%)	2.4% (0.8 to 3.7%)	1.0% (0.7 to 0.8%)	-2.0% (-7.9 to -0.5%)	-2.5% (-4.7 to -1.7%)	-19.5% (-24.3 to -14.8%)	-48.0% (-54.7 to -41.6%)
Botswana	4.3% (2.1 to 6.4%)	-18.0% (-21.4 to -13.4%)	-14.9% (-30.8 to 0.0%)	-13.7% (-12.5 to -13.5%)	-2.6% (-3.3 to -1.7%)	-1.7% (-1.9 to -1.3%)	-0.1% (-0.6 to 0.0%)	0.3% (0.6 to -0.2%)	1.4% (0.4 to 2.2%)	4.3% (5.2 to 3.4%)	-3.0% (-7.5 to -0.6%)	-3.2% (-5.9 to -2.1%)	-18.4% (-22.0 to -13.4%)	-42.9% (-47.0 to -38.3%)
Belize	4.3% (1.9 to 6.8%)	-13.9% (-18.0 to -9.9%)	-14.9% (-31.3 to 0.0%)	6.2% (3.0 to 9.4%)	0.1% (0.1 to 0.1%)	-0.9% (-1.2 to -0.7%)	-0.4% (-1.9 to 0.0%)	-1.3% (-1.2 to -1.4%)	3.6% (1.3 to 5.9%)	1.7% (1.7 to 1.8%)	-4.8% (-13.1 to -0.5%)	-1.1% (-2.3 to -0.8%)	-4.2% (-9.9 to -2.3%)	-22.9% (-27.0 to -20.1%)
Cape Verde	6.6% (3.4 to 11.6%)	-19.5% (-22.7 to -15.3%)	-15.9% (-33.9 to 0.0%)	5.2% (2.4 to 7.9%)	-1.4% (-1.9 to -0.9%)	-1.1% (-1.4 to -0.7%)	-0.3% (-1.4 to 0.0%)	-0.9% (-1.3 to -0.5%)	0.7% (0.1 to 1.7%)	1.7% (2.3 to 1.6%)	-5.5% (-14.4 to -0.7%)	-4.2% (-7.9 to -2.9%)	-15.5% (-15.4 to -11.2%)	-40.1% (-39.4 to -38.3%)

Location Name	Ambient pollution	House-hold air pollution	Low Hib vaccine coverage	Low pneumo-coccal vaccine coverage	No hand-washing	Second-hand smoke	Zinc deficiency	Breast-feeding	Low antibiotic coverage	Low birth weight and short gestation	Stunting	Under-weight	Wasting	All risk factors
Swaziland	3.8% (2.3 to 5.3%)	-24.6% (-30.6 to -17.9%)	-18.2% (-39.4 to 0.0%)	-21.5% (-23.0 to -18.9%)	-2.4% (-3.2 to -1.5%)	-0.7% (-1.2 to -0.2%)	0.3% (0.0 to 1.3%)	-1.2% (-2.0 to -0.5%)	2.1% (0.7 to 3.3%)	2.0% (3.8 to 1.5%)	-5.7% (-12.4 to -0.7%)	-2.1% (-3.8 to -1.3%)	-6.0% (-9.6 to -2.8%)	-40.8% (-46.7 to -35.2%)
Namibia	3.7% (1.8 to 6.6%)	-17.2% (-22.2 to -12.7%)	-20.2% (-44.0 to 0.0%)	-20.0% (-20.1 to -18.0%)	-3.2% (-4.1 to -2.1%)	-3.1% (-4.2 to -2.2%)	0.0% (0.0 to 0.1%)	-1.7% (-3.2 to -0.5%)	8.9% (4.0 to 13.5%)	5.1% (6.0 to 3.6%)	-5.3% (-14.3 to -0.8%)	-5.1% (-9.0 to -3.4%)	-9.7% (-14.1 to -6.3%)	-40.0% (-43.4 to -36.1%)
Lesotho	2.7% (1.6 to 4.0%)	-10.3% (-11.9 to -8.3%)	-19.0% (-42.3 to 0.0%)	-19.7% (-21.6 to -16.7%)	-0.2% (-0.3 to -0.1%)	-1.3% (-2.2 to -0.9%)	-0.2% (-1.0 to 0.0%)	-2.2% (-3.9 to -0.8%)	0.5% (0.2 to 1.3%)	4.4% (5.1 to 3.1%)	-3.7% (-5.7 to -1.1%)	-3.4% (-6.0 to -2.4%)	-40.7% (-49.4 to -28.0%)	-66.2% (-74.1 to -57.6%)
Gabon	11.8% (5.5 to 19.1%)	-22.2% (-29.3 to -14.9%)	-14.4% (-29.8 to 0.0%)	9.6% (5.7 to 13.7%)	-1.9% (-2.6 to -1.2%)	-0.4% (-0.9 to -0.1%)	-0.2% (-0.9 to 0.0%)	0.1% (0.4 to -0.5%)	2.5% (1.5 to 3.5%)	3.7% (5.9 to 2.1%)	-6.2% (-14.9 to -0.9%)	-3.0% (-5.6 to -1.9%)	-6.1% (-8.1 to -3.5%)	-30.7% (-30.5 to -28.2%)
Brazil	1.5% (0.5 to 3.3%)	-11.2% (-13.7 to -8.6%)	-13.6% (-27.5 to 0.0%)	-17.5% (-20.0 to -14.5%)	-2.3% (-3.1 to -1.6%)	-4.2% (-4.9 to -3.4%)	-0.1% (-0.4 to 0.0%)	-1.1% (-1.6 to -0.5%)	2.6% (1.0 to 4.3%)	1.9% (2.0 to 1.9%)	-1.8% (-4.8 to -0.2%)	-1.0% (-1.9 to -0.7%)	-7.6% (-10.1 to -4.4%)	-23.4% (-25.6 to -21.6%)
Ghana	4.5% (1.6 to 9.7%)	-16.6% (-20.8 to -12.5%)	-19.7% (-41.3 to 0.0%)	-21.1% (-22.9 to -18.2%)	-0.5% (-0.7 to -0.3%)	-0.5% (-0.8 to -0.3%)	-6.5% (-24.1 to 0.0%)	-2.1% (-3.9 to -0.7%)	-0.5% (-1.2 to -0.1%)	-1.1% (0.0 to -1.8%)	-6.2% (-13.6 to -1.1%)	-5.5% (-9.7 to -3.9%)	-11.3% (-10.5 to -8.5%)	-48.4% (-55.7 to -45.7%)
Kazakhstan	0.2% (-0.4 to 0.9%)	-5.5% (-7.5 to -3.8%)	-10.7% (-21.7 to 0.0%)	-10.2% (-10.2 to -9.5%)	-1.2% (-1.8 to -0.8%)	-1.4% (-1.6 to -1.0%)	0.2% (0.0 to 1.2%)	-0.7% (-1.4 to -0.2%)	1.4% (0.5 to 2.4%)	1.1% (1.3 to 0.2%)	-2.6% (-7.0 to -0.2%)	-1.6% (-3.2 to -0.9%)	-6.4% (-9.9 to -3.5%)	-18.2% (-19.2 to -17.0%)
Mauritania	3.3% (1.4 to 5.3%)	-5.7% (-5.6 to -4.0%)	-16.2% (-34.6 to 0.0%)	-11.7% (-10.8 to -11.7%)	-1.4% (-1.8 to -0.9%)	-1.6% (-2.2 to -1.0%)	0.2% (0.0 to 1.1%)	-1.3% (-2.9 to 0.0%)	1.2% (0.4 to 2.6%)	10.3% (12.2 to 9.4%)	-3.7% (-8.1 to -0.8%)	-3.4% (-6.2 to -1.8%)	3.7% (2.3 to 3.5%)	-5.3% (-7.3 to -4.3%)
Congo	7.3% (2.5 to 13.3%)	-20.3% (-26.2 to -14.3%)	-11.6% (-23.1 to 0.0%)	-11.2% (-9.9 to -11.3%)	-1.0% (-1.4 to -0.6%)	-0.7% (-1.2 to -0.4%)	-0.9% (-4.7 to 0.0%)	-0.1% (-0.7 to 0.3%)	6.3% (2.7 to 8.9%)	2.4% (2.7 to 1.5%)	-4.1% (-10.5 to -0.7%)	-4.6% (-8.6 to -2.9%)	11.0% (7.8 to 12.7%)	-17.0% (-19.0 to -18.8%)
Armenia	2.7% (1.2 to 3.1%)	-6.9% (-9.1 to -4.7%)	-10.9% (-21.6 to 0.0%)	-12.1% (-13.3 to -10.4%)	-0.7% (-1.0 to -0.4%)	-0.8% (-1.1 to -0.6%)	-0.4% (-1.9 to 0.0%)	-0.4% (-1.0 to 0.0%)	-0.6% (-1.1 to -0.2%)	2.1% (2.5 to 1.6%)	-1.8% (-4.6 to -0.1%)	-0.6% (-1.2 to -0.3%)	1.3% (0.5 to 0.9%)	-7.4% (-8.3 to -7.6%)
South Africa	5.0% (3.2 to 7.0%)	-13.1% (-16.8 to -10.1%)	-13.8% (-28.1 to 0.0%)	-15.7% (-16.4 to -14.2%)	-1.8% (-2.6 to -1.2%)	-5.0% (-6.3 to -3.5%)	-0.1% (-0.3 to 0.0%)	-0.2% (-0.4 to -0.1%)	1.6% (0.7 to 2.5%)	2.7% (3.3 to 2.4%)	-3.4% (-9.2 to -0.5%)	-2.5% (-4.7 to -1.8%)	-8.6% (-11.3 to -6.0%)	-28.4% (-30.2 to -26.6%)

Location Name	Ambient pollution	Household air pollution	Low Hib vaccine coverage	Low pneumococcal vaccine coverage	No handwashing	Secondhand smoke	Zinc deficiency	Breastfeeding	Low antibiotic coverage	Low birth weight and short gestation	Stunting	Underweight	Wasting	All risk factors
Cote d'Ivoire	2.9% (1.0 to 5.1%)	-14.9% (-16.7 to -10.2%)	-19.4% (-41.2 to 0.0%)	-17.6% (-17.1 to -16.5%)	-0.5% (-0.7 to -0.3%)	0.4% (0.1 to 1.0%)	-0.5% (-2.2 to 0.0%)	-1.3% (-2.6 to -0.4%)	5.1% (2.3 to 7.9%)	3.0% (3.8 to 2.6%)	-6.7% (-15.7 to -1.5%)	-3.1% (-5.4 to -2.1%)	-11.4% (-13.3 to -8.2%)	-37.4% (-38.7 to -34.4%)
Georgia	-0.1% (0.0 to -0.2%)	-2.8% (-3.8 to -2.2%)	-9.6% (-18.6 to 0.0%)	-7.9% (-7.7 to -7.5%)	-0.3% (-0.4 to -0.2%)	2.4% (2.0 to 2.7%)	-0.1% (-0.4 to 0.0%)	-0.5% (-0.7 to -0.4%)	1.1% (0.3 to 2.3%)	2.7% (2.6 to 2.7%)	-0.6% (-1.4 to -0.1%)	-0.2% (-0.5 to -0.1%)	-4.2% (-6.2 to -2.2%)	-4.6% (-5.8 to -4.1%)
Cameroon	9.4% (4.2 to 17.8%)	-20.4% (-26.5 to -13.7%)	-21.5% (-45.4 to 0.0%)	-22.8% (-24.0 to -20.4%)	-0.6% (-0.6 to -0.5%)	-1.3% (-1.5 to -0.9%)	-0.6% (-3.2 to 0.0%)	-1.5% (-2.5 to -0.5%)	-0.6% (-1.2 to -0.2%)	7.0% (6.5 to 5.6%)	-10.8% (-26.2 to -2.0%)	-4.7% (-8.7 to -3.0%)	-10.7% (-13.4 to -7.6%)	-43.1% (-42.8 to -41.1%)
Uzbekistan	2.6% (0.8 to 2.6%)	-10.1% (-14.2 to -6.2%)	-17.1% (-36.4 to 0.0%)	-18.8% (-21.0 to -16.0%)	-1.5% (-2.3 to -1.0%)	-0.5% (-0.8 to -0.3%)	-0.2% (-1.2 to 0.0%)	-0.5% (-1.3 to 0.1%)	1.3% (0.5 to 2.1%)	1.9% (1.8 to 1.3%)	-7.3% (-17.4 to -1.2%)	-3.6% (-6.9 to -2.3%)	-19.0% (-23.2 to -13.2%)	-40.8% (-41.9 to -39.2%)
Togo	2.6% (1.2 to 4.4%)	-11.1% (-11.6 to -8.7%)	-16.9% (-35.7 to 0.0%)	-18.1% (-19.2 to -16.0%)	-0.2% (-0.3 to -0.2%)	-0.9% (-1.5 to -0.5%)	-1.0% (-4.5 to 0.0%)	-1.7% (-2.9 to -0.7%)	2.7% (1.2 to 4.3%)	4.8% (5.5 to 3.8%)	-5.8% (-13.1 to -1.0%)	-4.3% (-8.0 to -2.9%)	7.6% (5.5 to 6.5%)	-13.5% (-15.5 to -14.7%)
The Gambia	2.8% (1.5 to 5.3%)	-11.5% (-11.7 to -8.3%)	-18.0% (-37.4 to 0.0%)	-19.9% (-21.4 to -17.6%)	-0.3% (-0.5 to -0.2%)	-1.2% (-2.2 to -0.7%)	-2.6% (-11.8 to 0.0%)	-0.8% (-1.7 to 0.0%)	5.8% (2.9 to 7.6%)	4.6% (5.2 to 4.2%)	-6.6% (-16.6 to -1.0%)	-5.7% (-9.9 to -3.7%)	-5.3% (-9.5 to -4.0%)	-29.0% (-34.1 to -26.4%)
Mali	2.2% (0.6 to 6.8%)	-9.7% (-7.0 to -9.5%)	-15.0% (-30.8 to 0.0%)	-12.6% (-11.4 to -12.9%)	-0.3% (-0.4 to -0.2%)	-0.4% (-0.6 to -0.2%)	0.3% (0.0 to 1.7%)	-1.2% (-3.5 to 0.6%)	14.9% (6.2 to 23.9%)	5.9% (5.4 to 6.3%)	-6.0% (-14.4 to -0.5%)	-8.2% (-15.1 to -5.4%)	-20.2% (-22.1 to -15.8%)	-41.8% (-41.5 to -40.8%)
Burkina Faso	2.9% (1.0 to 6.5%)	-11.7% (-12.8 to -11.2%)	-25.3% (-54.9 to 0.0%)	-28.5% (-31.9 to -24.2%)	-0.3% (-0.3 to -0.1%)	-2.4% (-3.1 to -1.6%)	0.1% (0.0 to 1.0%)	-0.9% (-1.9 to 0.0%)	2.3% (0.7 to 5.0%)	5.4% (6.5 to 4.8%)	-11.7% (-26.0 to -2.2%)	-8.8% (-16.1 to -5.8%)	11.5% (8.7 to 11.7%)	-21.1% (-16.0 to -24.8%)
Benin	3.4% (1.3 to 8.8%)	-14.0% (-13.2 to -11.5%)	-15.3% (-31.7 to 0.0%)	-16.0% (-15.7 to -15.6%)	-0.7% (-0.8 to -0.5%)	-1.6% (-2.3 to -1.0%)	-0.8% (-3.6 to 0.0%)	-1.0% (-1.8 to -0.3%)	0.6% (0.2 to 1.7%)	3.5% (2.6 to 3.4%)	-3.9% (-7.0 to -1.3%)	-4.4% (-7.5 to -3.0%)	-9.8% (-6.9 to -9.3%)	-31.9% (-29.3 to -33.9%)
Kyrgyzstan	0.8% (0.0 to 2.2%)	-6.1% (-8.7 to -4.0%)	-12.2% (-24.0 to 0.0%)	-10.7% (-10.3 to -10.2%)	-0.9% (-1.2 to -0.5%)	-1.0% (-1.3 to -0.7%)	-0.2% (-1.2 to 0.0%)	-0.7% (-1.2 to -0.3%)	1.5% (0.7 to 2.3%)	2.4% (2.1 to 2.6%)	-3.0% (-8.0 to -0.3%)	-0.9% (-1.8 to -0.5%)	-3.0% (-5.5 to -1.9%)	-13.4% (-14.5 to -12.4%)
Guinea-Bissau	2.1% (1.0 to 3.3%)	-9.6% (-8.4 to -8.5%)	-14.3% (-29.3 to 0.0%)	-13.6% (-13.3 to -12.8%)	-0.2% (-0.3 to -0.1%)	-0.5% (-0.7 to -0.4%)	-1.2% (-6.1 to 0.0%)	-1.1% (-1.9 to -0.5%)	0.3% (0.1 to 0.9%)	4.9% (4.7 to 4.4%)	-8.6% (-19.4 to -1.0%)	-4.2% (-8.0 to -2.4%)	-14.5% (-20.0 to -10.8%)	-32.9% (-37.7 to -30.4%)

Location Name	Ambient pollution	Household air pollution	Low Hib vaccine coverage	Low pneumococcal vaccine coverage	No handwashing	Second-hand smoke	Zinc deficiency	Breast-feeding	Low antibiotic coverage	Low birth weight and short gestation	Stunting	Under-weight	Wasting	All risk factors
Azerbaijan	0.9% (-0.3 to 1.9%)	-6.8% (-9.2 to -4.7%)	-12.5% (-25.5 to 0.0%)	-11.7% (-11.7 to -10.8%)	-1.5% (-2.1 to -0.9%)	-0.3% (-0.4 to -0.3%)	-1.4% (-6.2 to 0.0%)	-0.1% (-0.6 to 0.5%)	1.5% (0.6 to 2.3%)	-0.5% (-0.8 to 0.0%)	-2.5% (-7.2 to -0.4%)	-1.7% (-3.4 to -1.1%)	-7.2% (-7.2 to -5.2%)	-21.8% (-24.0 to -19.5%)
Nigeria	7.6% (3.1 to 14.7%)	-18.3% (-22.9 to -11.9%)	-9.2% (-18.7 to 0.0%)	-0.2% (3.6 to -3.1%)	-0.3% (-0.4 to -0.2%)	-0.4% (-1.2 to 0.2%)	-1.9% (-9.1 to 0.0%)	-0.5% (-2.0 to 0.7%)	1.4% (0.6 to 1.5%)	21.2% (17.2 to 24.5%)	-6.4% (-13.5 to -1.4%)	-2.5% (-5.0 to -1.6%)	-9.9% (-8.5 to -11.1%)	-17.1% (-12.3 to -20.4%)
**Low mortality, high incidence**														
Panama	2.3% (-0.5 to 5.4%)	-17.4% (-22.1 to -12.6%)	-26.3% (-58.4 to 0.0%)	-33.5% (-36.0 to -29.6%)	-4.2% (-5.4 to -2.7%)	-3.1% (-4.4 to -2.0%)	-0.7% (-3.8 to 0.0%)	0.3% (1.0 to -0.3%)	3.5% (2.7 to -0.1%)	1.6% (1.9 to 0.9%)	-1.0% (-1.1 to -0.2%)	-1.9% (-3.8 to -1.3%)	-33.3% (-40.8 to -19.7%)	-63.1% (-74.7 to -52.1%)
Lithuania	-2.6% (-2.8 to -2.1%)	-0.2% (-0.3 to -0.1%)	-15.6% (-34.1 to 0.0%)	-15.0% (-15.8 to -13.2%)	-0.4% (-0.7 to -0.3%)	-4.7% (-5.7 to -3.7%)	0.0% (0.0 to 0.0%)	-0.1% (-0.4 to 0.2%)	1.8% (0.7 to 3.1%)	1.5% (1.7 to 1.2%)	-1.4% (-4.3 to -0.1%)	-0.5% (-1.1 to -0.3%)	-7.4% (-11.6 to -4.6%)	-15.6% (-18.3 to -12.9%)
Seychelles	2.8% (0.3 to 6.5%)	-8.5% (-12.7 to -5.0%)	-21.2% (-47.0 to 0.0%)	-8.9% (-0.1 to -13.7%)	-0.3% (-0.5 to -0.2%)	-2.2% (-2.7 to -1.6%)	-0.4% (-1.9 to 0.0%)	-0.6% (-1.1 to -0.2%)	3.0% (0.8 to 6.4%)	2.9% (2.9 to 2.0%)	-0.8% (-2.8 to 0.0%)	-1.3% (-2.7 to -0.8%)	0.5% (0.8 to 0.6%)	-10.7% (-11.3 to -11.4%)
Latvia	-1.8% (-1.7 to -1.7%)	-1.8% (-2.8 to -1.0%)	-13.4% (-28.0 to 0.0%)	-11.0% (-11.5 to -9.8%)	-0.4% (-0.5 to -0.2%)	-0.3% (-0.3 to -0.2%)	0.0% (-0.1 to 0.0%)	0.0% (0.2 to -0.2%)	1.7% (0.5 to 2.9%)	2.5% (2.9 to 2.3%)	-1.1% (-3.2 to 0.0%)	-0.4% (-0.9 to -0.2%)	-5.9% (-8.9 to -3.5%)	-9.4% (-11.7 to -8.1%)
Mauritius	1.3% (-0.5 to 3.5%)	-5.2% (-7.9 to -3.0%)	-13.8% (-28.9 to 0.0%)	-9.0% (-5.5 to -10.7%)	-0.3% (-0.4 to -0.2%)	-1.6% (-2.1 to -1.1%)	-0.4% (-1.7 to 0.0%)	-0.4% (-0.7 to -0.2%)	2.0% (0.6 to 3.9%)	0.9% (1.0 to 0.7%)	-2.3% (-6.4 to -0.4%)	-2.9% (-5.4 to -2.2%)	-11.5% (-9.4 to -9.8%)	-23.3% (-20.3 to -24.1%)
Estonia	-0.9% (-0.2 to -1.2%)	-3.0% (-4.5 to -1.8%)	-12.8% (-26.2 to 0.0%)	5.2% (2.7 to 7.9%)	-0.3% (-0.4 to -0.2%)	-1.1% (-1.4 to -0.7%)	0.3% (0.0 to 1.3%)	0.0% (0.4 to -0.2%)	1.8% (0.8 to 2.6%)	2.8% (3.1 to 2.5%)	-1.3% (-4.0 to 0.0%)	-0.4% (-0.9 to -0.2%)	-6.3% (-9.4 to -3.5%)	-10.1% (-12.3 to -7.5%)
Kuwait	0.6% (0.5 to 0.6%)	-0.5% (-0.9 to -0.2%)	-20.3% (-43.2 to 0.0%)	-25.5% (-30.0 to -20.9%)	-0.2% (-0.3 to -0.1%)	1.0% (0.9 to 1.0%)	0.4% (0.0 to 1.7%)	-0.5% (-0.3 to -0.5%)	4.5% (1.7 to 8.4%)	2.0% (2.2 to 2.0%)	-0.9% (-3.0 to 0.0%)	-0.3% (-0.7 to -0.2%)	-7.4% (-8.2 to -5.1%)	-7.2% (-8.1 to -6.5%)
Costa Rica	1.8% (0.2 to 3.6%)	-7.7% (-9.8 to -5.5%)	-14.6% (-30.3 to 0.0%)	-18.0% (-20.8 to -14.9%)	-1.0% (-1.4 to -0.6%)	-1.1% (-1.7 to -0.6%)	-0.1% (-0.3 to 0.0%)	-0.3% (-0.6 to 0.1%)	1.2% (1.0 to 1.5%)	0.8% (0.9 to 0.8%)	-1.7% (-5.5 to 0.0%)	-0.4% (-0.9 to -0.2%)	-7.0% (-11.0 to -3.8%)	-17.5% (-19.9 to -15.5%)

Location Name	Ambient pollution	House-hold air pollution	Low Hib vaccine coverage	Low pneumo-coccal vaccine coverage	No hand-washing	Second-hand smoke	Zinc deficiency	Breast-feeding	Low antibiotic coverage	Low birth weight and short gestation	Stunting	Under-weight	Wasting	All risk factors
Ukraine	-2.2% (-3.3 to -1.4%)	-1.4% (-2.3 to -0.8%)	-4.3% (-9.2 to 0.0%)	6.7% (3.8 to 9.8%)	-0.2% (-0.3 to -0.1%)	-2.6% (-3.3 to -2.1%)	0.0% (0.0 to 0.0%)	-0.2% (-0.3 to -0.2%)	1.1% (0.5 to 1.7%)	2.2% (2.6 to 1.8%)	-1.4% (-2.5 to -0.2%)	-0.3% (-0.7 to -0.2%)	-6.1% (-7.0 to -3.9%)	-12.4% (-14.0 to -10.7%)
Malaysia	1.6% (-0.6 to 4.0%)	-5.5% (-8.7 to -3.2%)	-14.6% (-30.0 to 0.0%)	5.2% (2.4 to 8.0%)	-0.2% (-0.3 to -0.1%)	-3.6% (-4.1 to -3.0%)	-0.2% (-1.0 to 0.0%)	-0.5% (-0.8 to -0.2%)	2.3% (0.6 to 4.4%)	1.3% (1.5 to 1.2%)	-4.0% (-10.6 to -0.2%)	-2.9% (-5.2 to -1.9%)	-3.1% (-3.9 to -2.1%)	-17.8% (-17.0 to -19.1%)
Lebanon	0.1% (-0.3 to 0.0%)	0.0% (-0.1 to 0.0%)	-14.5% (-29.1 to 0.0%)	-12.7% (-10.8 to -12.4%)	-0.7% (-1.0 to -0.4%)	-2.3% (-2.6 to -2.0%)	0.0% (0.0 to 0.2%)	-0.1% (-1.0 to 0.5%)	3.5% (1.3 to 6.1%)	7.0% (7.5 to 6.4%)	-2.8% (-7.6 to -0.2%)	-1.2% (-2.5 to -0.7%)	-9.5% (-13.4 to -5.4%)	-10.4% (-14.2 to -8.7%)
Sri Lanka	-1.7% (1.2 to -2.3%)	-13.2% (-15.7 to -10.4%)	-13.6% (-27.6 to 0.0%)	4.2% (1.8 to 6.6%)	-2.1% (-2.9 to -1.3%)	-2.5% (-3.1 to -1.9%)	-0.5% (-2.2 to 0.0%)	-1.3% (-1.8 to -0.8%)	2.0% (0.7 to 3.9%)	1.5% (1.6 to 1.2%)	-4.8% (-11.2 to -0.6%)	-5.7% (-10.1 to -4.0%)	-13.5% (-12.4 to -11.2%)	-41.7% (-40.3 to -42.5%)
Saudi Arabia	7.4% (4.7 to 8.0%)	-4.2% (-7.3 to -1.9%)	-11.8% (-22.9 to 0.0%)	-15.7% (-17.7 to -13.3%)	-1.0% (-1.4 to -0.6%)	-1.9% (-2.1 to -1.4%)	-0.2% (-0.8 to 0.0%)	-0.4% (-1.1 to 0.1%)	3.4% (1.3 to 6.7%)	1.4% (1.6 to 1.2%)	-4.9% (-12.1 to -0.7%)	-4.7% (-8.6 to -3.3%)	-13.7% (-17.1 to -8.7%)	-23.1% (-24.3 to -21.6%)
Russian Federation	-2.3% (-2.1 to -1.8%)	0.0% (-0.1 to 0.0%)	-13.5% (-27.0 to 0.0%)	-6.9% (-6.0 to -7.2%)	-0.4% (-0.6 to -0.3%)	-0.5% (-0.5 to -0.4%)	-0.1% (-0.4 to 0.0%)	0.0% (0.2 to -0.1%)	2.1% (0.9 to 3.3%)	2.2% (2.3 to 2.1%)	-2.5% (-6.7 to -0.4%)	-0.7% (-1.5 to -0.4%)	-6.2% (-5.5 to -4.6%)	-10.4% (-8.3 to -10.2%)
Belarus	-1.6% (-0.7 to -1.6%)	-1.1% (-1.9 to -0.6%)	-12.1% (-24.1 to 0.0%)	-13.8% (-15.9 to -11.5%)	-0.7% (-1.0 to -0.5%)	-0.4% (-0.6 to -0.2%)	0.0% (0.0 to 0.2%)	-0.2% (-0.4 to 0.1%)	2.6% (1.0 to 4.0%)	1.9% (2.4 to 1.6%)	-1.1% (-3.2 to 0.0%)	-0.4% (-1.0 to -0.2%)	-6.3% (-9.9 to -3.3%)	-9.8% (-12.0 to -8.0%)
Libya	2.0% (0.0 to 4.0%)	-0.3% (-0.5 to -0.1%)	-12.5% (-25.1 to 0.0%)	-14.7% (-15.6 to -13.0%)	0.0% (0.0 to 0.0%)	-0.8% (-0.9 to -0.7%)	0.2% (0.0 to 0.9%)	-0.3% (-0.5 to 0.0%)	2.8% (1.2 to 5.0%)	3.4% (3.9 to 2.5%)	-1.8% (-4.5 to -0.1%)	-1.0% (-1.9 to -0.7%)	-4.6% (-3.4 to -3.3%)	-4.1% (-1.4 to -4.7%)
Venezuela	-1.7% (-1.0 to -2.5%)	-1.7% (-2.8 to -0.9%)	-13.5% (-27.8 to 0.0%)	0.1% (-2.9 to 3.4%)	-1.4% (-2.0 to -0.9%)	-3.1% (-3.8 to -2.2%)	-0.2% (-1.1 to 0.0%)	-0.1% (-0.7 to 0.3%)	2.3% (0.7 to 2.7%)	1.6% (1.9 to 1.7%)	-1.7% (-4.4 to -0.2%)	-1.0% (-2.0 to -0.8%)	-2.8% (-1.3 to -2.6%)	-13.1% (-9.5 to -14.4%)
Palestine	0.6% (0.2 to 1.6%)	-1.2% (-2.2 to -0.6%)	-17.7% (-36.3 to 0.0%)	-23.2% (-26.6 to -19.3%)	-1.1% (-1.6 to -0.8%)	-1.4% (-1.6 to -0.9%)	-0.2% (-1.0 to 0.0%)	-0.3% (-0.9 to 0.1%)	7.1% (2.9 to 11.3%)	0.9% (0.9 to 0.7%)	-3.0% (-8.8 to -0.2%)	-3.6% (-6.8 to -2.5%)	-21.4% (-25.6 to -14.7%)	-30.7% (-32.9 to -26.7%)
Chile	0.7% (-0.5 to 1.6%)	-4.1% (-5.0 to -3.0%)	-11.5% (-22.5 to 0.0%)	-13.4% (-14.6 to -11.7%)	-0.5% (-0.6 to -0.3%)	-2.4% (-2.9 to -1.7%)	-0.1% (-0.3 to 0.0%)	-0.2% (-0.5 to -0.1%)	1.9% (0.7 to 3.0%)	2.0% (2.1 to 2.0%)	-1.2% (-3.8 to -0.1%)	-0.4% (-0.7 to -0.2%)	-5.7% (-6.0 to -3.3%)	-12.4% (-12.6 to -11.3%)

Location Name	Ambient pollution	House-hold air pollution	Low Hib vaccine coverage	Low pneumo-coccal vaccine coverage	No hand-washing	Second-hand smoke	Zinc deficiency	Breast-feeding	Low antibiotic coverage	Low birth weight and short gestation	Stunting	Under-weight	Wasting	All risk factors
Oman	6.3% (3.9 to 9.2%)	-8.0% (-12.1 to -4.5%)	-16.0% (-32.6 to 0.0%)	-19.7% (-23.0 to -16.3%)	-2.2% (-3.1 to -1.4%)	-3.3% (-4.6 to -2.3%)	-0.7% (-3.4 to 0.0%)	-0.1% (-0.6 to 0.3%)	3.6% (1.5 to 6.2%)	3.9% (4.3 to 3.2%)	-4.3% (-12.0 to -0.5%)	-4.9% (-8.7 to -3.2%)	-15.9% (-19.5 to -12.9%)	-30.0% (-32.6 to -28.5%)
Jordan	-0.9% (-2.6 to 0.3%)	-0.3% (-0.5 to -0.1%)	-23.8% (-51.4 to 0.0%)	11.0% (6.2 to 15.9%)	-1.0% (-1.3 to -0.7%)	-3.3% (-4.0 to -3.0%)	-0.3% (-1.3 to 0.0%)	-0.7% (-1.2 to -0.2%)	5.0% (1.9 to 8.6%)	12.0% (12.6 to 12.7%)	-2.8% (-8.8 to -0.2%)	-1.2% (-2.4 to -0.7%)	-7.9% (-12.5 to -3.6%)	-9.1% (-14.3 to -6.5%)
Bulgaria	-1.2% (-1.3 to -1.3%)	-3.3% (-4.7 to -2.2%)	-11.8% (-23.9 to 0.0%)	-14.2% (-15.5 to -12.2%)	-0.2% (-0.2 to -0.1%)	-3.0% (-3.5 to -2.6%)	0.1% (0.0 to 0.5%)	0.3% (0.4 to 0.2%)	1.7% (0.7 to 3.0%)	0.8% (0.7 to 1.0%)	-1.2% (-3.6 to -0.1%)	-0.6% (-1.4 to -0.3%)	-5.1% (-7.7 to -2.9%)	-14.9% (-16.1 to -13.6%)
Colombia	1.9% (0.0 to 3.9%)	-9.8% (-12.0 to -7.4%)	-14.4% (-29.9 to 0.0%)	-16.1% (-17.4 to -14.0%)	-1.1% (-1.6 to -0.7%)	-3.2% (-4.1 to -2.2%)	-0.2% (-1.1 to 0.0%)	-1.7% (-2.5 to -0.8%)	2.1% (0.0 to 7.2%)	3.7% (3.9 to 3.3%)	-2.1% (-5.1 to -0.3%)	-1.8% (-3.5 to -1.4%)	-19.5% (-24.2 to -12.3%)	-34.7% (-39.4 to -30.7%)
Thailand	4.3% (2.0 to 6.3%)	-12.8% (-16.0 to -9.7%)	0.0% (0.0 to 0.0%)	6.0% (3.4 to 8.9%)	-0.6% (-0.8 to -0.3%)	-3.9% (-5.0 to -2.8%)	-0.2% (-0.9 to 0.0%)	-0.1% (-0.3 to 0.1%)	4.2% (1.7 to 7.2%)	2.8% (3.1 to 2.2%)	-2.6% (-6.2 to -0.5%)	-2.8% (-5.0 to -2.0%)	-7.4% (-7.0 to -5.7%)	-24.7% (-24.3 to -24.5%)
Syria	-0.5% (-0.2 to -0.2%)	-0.2% (-0.4 to -0.1%)	-6.6% (-13.3 to 0.0%)	1.9% (0.1 to 3.5%)	-0.7% (-1.1 to -0.5%)	-2.1% (-2.7 to -1.5%)	-0.1% (-0.3 to 0.0%)	-0.2% (-0.6 to 0.2%)	1.1% (0.5 to 1.8%)	1.0% (1.2 to 0.8%)	-3.7% (-7.6 to -0.7%)	-3.5% (-6.3 to -2.4%)	-20.4% (-21.8 to -16.9%)	-27.8% (-28.2 to -27.6%)
Paraguay	1.5% (0.6 to 2.6%)	-13.6% (-16.7 to -10.8%)	-15.5% (-31.8 to 0.0%)	-19.1% (-21.5 to -16.1%)	-3.5% (-4.6 to -2.3%)	-2.1% (-2.3 to -1.6%)	0.0% (0.0 to -0.1%)	-1.7% (-3.0 to -0.6%)	1.1% (0.3 to 2.0%)	2.0% (2.4 to 1.4%)	-3.7% (-11.1 to -0.1%)	-0.5% (-1.1 to -0.3%)	-2.0% (-3.7 to -0.9%)	-23.3% (-24.2 to -22.3%)
Algeria	1.4% (0.0 to 1.1%)	-1.0% (-1.8 to -0.4%)	-14.7% (-29.9 to 0.0%)	-18.2% (-20.3 to -15.5%)	-0.8% (-1.1 to -0.5%)	-2.6% (-3.6 to -2.1%)	-0.8% (-3.4 to 0.0%)	0.2% (0.4 to -0.1%)	0.7% (0.3 to 1.0%)	2.1% (2.0 to 1.8%)	-6.4% (-15.5 to -0.8%)	-6.1% (-10.9 to -4.3%)	-5.8% (-6.4 to -5.5%)	-20.8% (-19.9 to -22.7%)
Maldives	1.4% (0.3 to 3.6%)	-18.5% (-22.2 to -14.4%)	-12.2% (-24.0 to 0.0%)	3.1% (1.1 to 5.2%)	-1.7% (-2.2 to -1.1%)	-1.8% (-2.1 to -1.7%)	-1.0% (-4.1 to 0.0%)	-0.4% (-1.0 to 0.1%)	2.3% (0.6 to 4.8%)	2.2% (2.2 to 2.1%)	-7.1% (-15.3 to -1.4%)	-10.5% (-17.0 to -7.3%)	-20.8% (-22.1 to -18.5%)	-43.7% (-44.4 to -43.2%)
Tunisia	3.7% (1.9 to 4.5%)	-4.0% (-6.4 to -2.3%)	-11.6% (-22.6 to 0.0%)	4.3% (2.0 to 6.6%)	-1.0% (-1.5 to -0.6%)	-2.8% (-3.7 to -2.1%)	-0.1% (-0.7 to 0.0%)	0.2% (0.2 to 0.0%)	2.3% (0.9 to 3.4%)	3.8% (4.1 to 3.2%)	-2.0% (-5.5 to -0.1%)	-1.1% (-2.2 to -0.7%)	-22.7% (-26.2 to -17.0%)	-25.0% (-28.3 to -21.6%)
**Low mortality, low incidence**														

Location Name	Ambient pollution	House-hold air pollution	Low Hib vaccine coverage	Low pneumo-coccal vaccine coverage	No hand-washing	Second-hand smoke	Zinc deficiency	Breast-feeding	Low antibiotic coverage	Low birth weight and short gestation	Stunting	Under-weight	Wasting	All risk factors
Dominica	6.1% (3.1 to 8.8%)	-27.4% (-35.9 to -19.9%)	-31.1% (-72.4 to 0.0%)	12.4% (6.9 to 17.8%)	-3.5% (-4.9 to -2.5%)	-1.7% (-2.6 to -1.0%)	-0.3% (-2.0 to 0.0%)	-0.3% (-1.1 to 0.3%)	3.5% (1.7 to 6.1%)	-2.2% (-1.6 to -3.2%)	-3.3% (-9.7 to -0.1%)	-2.8% (-5.6 to -1.7%)	-15.9% (-24.7 to -6.8%)	-62.0% (-73.9 to -57.3%)
Antigua and Barbuda	-1.7% (-1.7 to -2.1%)	-2.7% (-4.7 to -1.4%)	-25.7% (-59.3 to 0.0%)	11.7% (6.6 to 16.8%)	-1.4% (-1.8 to -0.8%)	-0.9% (-1.2 to -0.6%)	-0.4% (-2.2 to 0.0%)	-0.3% (-0.5 to 0.0%)	2.9% (1.3 to 4.5%)	1.8% (2.1 to 1.2%)	-1.8% (-6.1 to -0.1%)	-1.2% (-2.6 to -0.6%)	-7.8% (-12.8 to -4.0%)	-18.8% (-23.8 to -13.5%)
Brunei	-1.4% (2.5 to -1.6%)	-2.2% (-5.1 to -0.6%)	-25.8% (-59.8 to 0.0%)	7.7% (3.8 to 11.7%)	-0.2% (-0.3 to -0.1%)	-6.0% (-7.8 to -4.7%)	0.0% (0.1 to 0.0%)	-0.2% (-0.5 to -0.1%)	2.3% (1.1 to 4.0%)	1.3% (1.6 to 1.3%)	-0.3% (-1.2 to 0.1%)	-0.6% (-0.9 to -0.4%)	-2.4% (-4.0 to -0.4%)	-12.8% (-16.4 to -9.9%)
Greece	-2.7% (-2.8 to -2.9%)	-0.8% (-1.5 to -0.3%)	-21.6% (-49.0 to 0.0%)	-28.9% (-33.1 to -23.9%)	0.0% (0.0 to 0.0%)	-8.8% (-10.9 to -7.3%)	0.4% (0.0 to 1.6%)	0.2% (0.3 to 0.2%)	1.7% (0.6 to 3.0%)	1.6% (1.8 to 1.7%)	-0.6% (-2.0 to 0.0%)	-0.3% (-0.8 to -0.2%)	-4.4% (-7.1 to -2.4%)	-16.1% (-18.1 to -14.5%)
Guam	0.3% (-0.7 to 1.6%)	-1.5% (-2.5 to -0.7%)	0.0% (0.0 to 0.0%)	7.2% (3.3 to 11.0%)	-0.7% (-1.1 to -0.5%)	0.2% (0.5 to -0.6%)	0.1% (0.0 to -0.2%)	-0.4% (-0.7 to -0.2%)	1.3% (0.5 to 1.6%)	0.2% (-0.2 to 0.7%)	-0.8% (-2.3 to 0.0%)	-0.4% (-0.8 to -0.2%)	-7.7% (-10.1 to -4.8%)	-12.6% (-15.1 to -10.3%)
Netherlands	-2.9% (-2.8 to -2.2%)	-0.2% (-0.3 to -0.1%)	-16.5% (-35.2 to 0.0%)	-20.6% (-23.1 to -17.4%)	0.0% (0.0 to 0.0%)	-2.7% (-3.3 to -1.9%)	0.5% (0.0 to 2.0%)	0.2% (0.1 to 0.3%)	1.3% (0.4 to 2.3%)	0.7% (0.7 to 0.6%)	-0.3% (-1.0 to 0.0%)	-0.1% (-0.4 to -0.1%)	-2.3% (-3.9 to -1.4%)	-7.5% (-8.5 to -6.6%)
Canada	-1.9% (-2.6 to -0.1%)	0.0% (-0.1 to 0.0%)	-2.5% (-6.1 to 0.0%)	-20.4% (-21.8 to -17.8%)	0.0% (-0.1 to 0.0%)	-2.1% (-2.7 to -1.4%)	0.5% (0.0 to 2.3%)	-0.3% (-0.4 to -0.4%)	1.2% (0.5 to 2.1%)	0.9% (0.8 to 1.0%)	-0.4% (-1.0 to 0.0%)	-0.2% (-0.6 to -0.1%)	-2.7% (-4.8 to -1.7%)	-6.9% (-8.5 to -5.7%)
France	-2.5% (-2.7 to -1.7%)	-0.4% (-0.8 to -0.2%)	-16.5% (-34.9 to 0.0%)	-20.7% (-23.2 to -17.5%)	0.0% (0.0 to 0.0%)	-6.5% (-7.6 to -5.3%)	0.0% (0.2 to 0.0%)	-0.2% (-0.2 to -0.1%)	1.5% (0.9 to 2.8%)	0.9% (0.8 to 1.0%)	-0.4% (-1.4 to 0.0%)	-0.2% (-0.4 to -0.1%)	-2.9% (-4.0 to -1.3%)	-12.0% (-13.6 to -10.7%)
Germany	-2.9% (-3.2 to -1.9%)	-0.3% (-0.6 to -0.1%)	-6.7% (-15.0 to 0.0%)	-18.2% (-19.7 to -15.9%)	0.0% (0.0 to 0.0%)	-3.3% (-4.3 to -2.5%)	0.2% (0.0 to 0.9%)	0.0% (0.1 to 0.1%)	1.0% (0.4 to 1.3%)	0.6% (0.6 to 0.7%)	-0.2% (-0.7 to 0.0%)	-0.2% (-0.4 to -0.1%)	-1.7% (-3.3 to -0.6%)	-7.7% (-8.7 to -6.9%)
Belgium	-3.1% (-2.9 to -2.2%)	-0.2% (-0.3 to -0.1%)	-18.0% (-39.0 to 0.0%)	-23.3% (-26.8 to -19.4%)	0.0% (0.0 to 0.0%)	-5.2% (-6.4 to -4.1%)	-0.2% (-0.6 to 0.0%)	-0.2% (-0.4 to -0.1%)	1.2% (0.7 to 2.2%)	0.7% (0.8 to 0.7%)	-0.3% (-0.9 to 0.0%)	-0.2% (-0.4 to -0.1%)	-2.5% (-4.2 to -0.8%)	-11.3% (-11.7 to -10.4%)
Australia	-1.9% (-1.5 to -0.4%)	-0.2% (-0.3 to 0.0%)	-19.9% (-43.5 to 0.0%)	-24.9% (-28.5 to -20.6%)	-0.2% (-0.2 to -0.1%)	-0.5% (-0.8 to 0.0%)	-0.1% (-0.7 to 0.0%)	0.1% (0.2 to 0.1%)	1.8% (0.6 to 3.4%)	1.2% (1.3 to 1.1%)	-0.6% (-1.9 to 0.0%)	-0.2% (-0.5 to -0.1%)	-3.2% (-5.4 to -1.2%)	-5.6% (-8.2 to -4.4%)

Location Name	Ambient pollution	House-hold air pollution	Low Hib vaccine coverage	Low pneumo-coccal vaccine coverage	No hand-washing	Second-hand smoke	Zinc deficiency	Breast-feeding	Low antibiotic coverage	Low birth weight and short gestation	Stunting	Under-weight	Wasting	All risk factors
Sweden	-1.7% (-1.7 to - 0.7%)	-0.3% (-0.6 to - 0.1%)	-16.4% (-34.6 to 0.0%)	-19.4% (-22.1 to - 16.2%)	0.0% (-0.1 to 0.0%)	-2.9% (-4.1 to - 2.0%)	0.3% (0.0 to 1.3%)	-0.5% (-0.7 to - 0.5%)	0.8% (0.3 to 1.0%)	0.9% (1.0 to 0.8%)	-0.3% (-1.4 to 0.0%)	-0.2% (-0.4 to - 0.1%)	-2.6% (-4.1 to - 1.1%)	-7.5% (-9.4 to - 6.2%)
Finland	-1.3% (-1.6 to - 0.3%)	-0.2% (-0.4 to 0.0%)	-0.2% (-0.7 to 0.0%)	-12.6% (-13.1 to - 11.2%)	0.0% (0.0 to 0.0%)	0.4% (0.5 to 0.3%)	0.0% (0.0 to 0.0%)	-0.1% (-0.1 to 0.0%)	1.0% (0.4 to 2.0%)	2.0% (2.1 to 1.9%)	-0.2% (-0.8 to 0.0%)	-0.1% (-0.3 to 0.0%)	-1.7% (-2.4 to - 0.7%)	-1.3% (-2.8 to - 0.3%)
Japan	-1.8% (-1.5 to - 1.4%)	-0.1% (-0.2 to - 0.1%)	-16.5% (-35.4 to 0.0%)	-21.4% (-24.9 to - 17.7%)	-0.1% (-0.1 to - 0.1%)	-0.7% (-1.0 to - 0.5%)	-0.2% (-0.8 to 0.0%)	-0.1% (-0.1 to 0.0%)	1.1% (0.5 to 1.7%)	1.1% (1.2 to 1.2%)	-0.6% (-1.8 to 0.0%)	-0.5% (-1.0 to - 0.4%)	-2.5% (-3.3 to - 1.8%)	-5.7% (-6.5 to - 5.0%)
Iceland	-1.8% (-3.6 to - 0.5%)	-0.2% (-0.5 to 0.0%)	2.1% (0.0 to 6.1%)	-18.8% (-20.3 to - 16.2%)	0.0% (0.0 to 0.0%)	-7.5% (-9.4 to - 5.6%)	0.8% (0.0 to 3.4%)	0.0% (-0.1 to 0.0%)	1.2% (0.5 to 1.4%)	1.7% (1.7 to 1.8%)	-0.3% (-1.7 to 0.0%)	-0.2% (-0.4 to - 0.1%)	-2.3% (-3.8 to - 1.2%)	-10.1% (-9.6 to - 9.4%)
Norway	-1.6% (-1.5 to - 1.1%)	-0.3% (-0.6 to - 0.1%)	-14.5% (-29.6 to 0.0%)	-16.0% (-17.8 to - 13.6%)	0.0% (0.0 to 0.0%)	-3.2% (-4.2 to - 2.2%)	0.2% (0.0 to 1.3%)	0.0% (-0.1 to 0.0%)	1.1% (0.5 to 1.7%)	1.7% (1.6 to 1.7%)	-0.3% (-1.0 to 0.0%)	-0.2% (-0.4 to - 0.1%)	-2.0% (-4.1 to - 1.1%)	-5.9% (-6.8 to - 5.2%)
Denmark	-2.3% (-2.6 to - 1.7%)	-0.1% (-0.2 to 0.0%)	-16.4% (-34.8 to 0.0%)	-21.1% (-24.4 to - 17.4%)	0.0% (0.0 to 0.0%)	-3.2% (-4.3 to - 2.2%)	0.4% (0.0 to 1.8%)	-0.7% (-0.9 to - 0.5%)	0.9% (0.2 to 1.3%)	1.0% (1.1 to 0.9%)	-0.2% (-0.9 to 0.0%)	-0.1% (-0.3 to - 0.1%)	-2.2% (-3.3 to - 1.0%)	-7.6% (-8.7 to - 6.6%)
Malta	-1.7% (-1.2 to - 1.6%)	-0.5% (-0.9 to - 0.2%)	-17.3% (-37.7 to 0.0%)	7.4% (4.1 to 10.8%)	0.0% (0.0 to 0.0%)	-3.1% (-3.8 to - 2.4%)	0.0% (0.0 to 0.2%)	-0.2% (-0.3 to 0.0%)	1.4% (0.6 to 2.3%)	1.8% (1.9 to 1.6%)	-0.4% (-1.6 to 0.0%)	-0.2% (-0.5 to - 0.1%)	-2.9% (-5.1 to - 1.6%)	-7.6% (-9.1 to - 6.1%)
Ireland	-1.7% (-1.7 to - 1.4%)	-0.4% (-0.9 to - 0.2%)	-15.7% (-32.2 to 0.0%)	-17.0% (-18.7 to - 14.5%)	0.0% (0.0 to 0.0%)	-3.5% (-4.4 to - 2.7%)	0.0% (-0.2 to 0.0%)	-0.2% (-0.2 to 0.0%)	1.3% (0.6 to 1.9%)	2.1% (2.3 to 1.9%)	-0.4% (-1.4 to 0.0%)	-0.2% (-0.5 to - 0.1%)	-3.0% (-4.7 to - 1.4%)	-7.6% (-10.0 to - 6.1%)
Luxembo urg	-2.5% (-2.2 to - 2.0%)	-0.2% (-0.5 to - 0.1%)	-17.5% (-36.7 to 0.0%)	-20.4% (-23.2 to - 17.1%)	0.0% (0.0 to 0.0%)	-2.6% (-3.4 to - 2.1%)	0.0% (-0.2 to 0.0%)	-0.2% (-0.4 to - 0.2%)	1.3% (0.5 to 1.7%)	1.8% (1.9 to 1.8%)	-0.2% (-1.1 to 0.0%)	-0.1% (-0.3 to - 0.1%)	-2.1% (-3.4 to - 1.1%)	-6.5% (-8.6 to - 5.5%)
Andorra	-2.5% (-2.1 to - 1.8%)	-0.3% (-0.6 to - 0.1%)	-16.0% (-33.6 to 0.0%)	-20.2% (-23.0 to - 16.8%)	0.0% (0.0 to 0.0%)	-1.5% (-2.0 to - 1.0%)	0.0% (0.0 to 0.1%)	-0.1% (-0.2 to - 0.1%)	1.1% (0.4 to 1.8%)	0.8% (0.8 to 0.7%)	-0.2% (-0.8 to 0.0%)	-0.1% (-0.2 to - 0.1%)	-1.2% (-1.9 to - 0.7%)	-5.4% (-6.3 to - 5.3%)
United States	-2.2% (-2.2 to - 1.4%)	0.0% (-0.1 to 0.0%)	-16.3% (-34.6 to 0.0%)	-21.3% (-24.0 to - 18.0%)	-0.1% (-0.1 to 0.0%)	-4.5% (-5.8 to - 3.3%)	-0.4% (-1.7 to 0.0%)	-0.6% (-1.1 to - 0.5%)	0.8% (0.3 to 1.2%)	1.1% (1.0 to 1.1%)	-0.3% (-1.0 to 0.0%)	-0.2% (-0.4 to - 0.1%)	-1.3% (-2.1 to - 0.5%)	-8.3% (-10.0 to - 7.3%)

Location Name	Ambient pollution	House-hold air pollution	Low Hib vaccine coverage	Low pneumo-coccal vaccine coverage	No hand-washing	Second-hand smoke	Zinc deficiency	Breast-feeding	Low antibiotic coverage	Low birth weight and short gestation	Stunting	Under-weight	Wasting	All risk factors
Spain	-1.4% (-0.4 to - 1.6%)	-1.4% (-2.5 to - 0.6%)	-15.3% (-31.7 to 0.0%)	-15.4% (-14.9 to - 14.1%)	0.0% (0.0 to 0.0%)	-4.9% (-6.1 to - 3.9%)	-0.1% (-0.3 to 0.0%)	0.1% (0.1 to 0.0%)	1.5% (0.7 to 2.8%)	2.3% (2.2 to 2.2%)	-0.2% (-1.2 to 0.0%)	-0.2% (-0.3 to - 0.1%)	-1.7% (-2.8 to - 1.2%)	-8.0% (-8.6 to - 7.7%)
Austria	-2.2% (-2.3 to - 2.0%)	-0.4% (-0.8 to - 0.2%)	-12.8% (-25.7 to 0.0%)	4.1% (1.7 to 6.6%)	0.0% (-0.1 to 0.0%)	-1.2% (-1.5 to - 0.9%)	0.0% (0.0 to 0.1%)	-0.2% (-0.2 to - 0.1%)	1.0% (0.4 to 1.8%)	1.1% (1.2 to 1.2%)	-0.3% (-1.1 to 0.0%)	-0.2% (-0.3 to - 0.1%)	-2.3% (-4.0 to - 1.1%)	-5.8% (-7.0 to - 4.7%)
United Kingdom	-2.0% (-1.0 to - 1.9%)	-0.7% (-1.4 to - 0.3%)	-17.0% (-36.1 to 0.0%)	-20.5% (-23.1 to - 17.2%)	0.0% (-0.1 to 0.0%)	-2.7% (-3.4 to - 1.9%)	-0.2% (-0.8 to 0.0%)	-0.6% (-0.9 to - 0.3%)	1.8% (0.7 to 2.9%)	1.2% (1.2 to 1.3%)	-0.5% (-1.6 to 0.0%)	-0.2% (-0.5 to - 0.1%)	-3.0% (-4.5 to - 1.2%)	-8.7% (-11.3 to - 7.5%)
Switzerla nd	-2.4% (-2.8 to - 1.7%)	-0.2% (-0.3 to - 0.1%)	-4.7% (-13.4 to 0.0%)	-15.5% (-16.7 to - 13.6%)	0.0% (0.0 to 0.0%)	-2.8% (-2.9 to - 2.3%)	0.0% (-0.1 to 0.0%)	0.1% (0.0 to 0.1%)	0.8% (0.4 to 1.0%)	0.7% (0.7 to 0.7%)	-0.2% (-0.8 to 0.0%)	-0.1% (-0.1 to 0.0%)	-1.7% (-2.6 to - 0.7%)	-6.6% (-8.9 to - 6.4%)
Italy	-1.9% (-3.0 to - 1.5%)	-0.4% (-0.7 to - 0.2%)	-14.4% (-29.7 to 0.0%)	-16.9% (-19.1 to - 14.2%)	0.0% (0.0 to 0.0%)	-4.0% (-5.1 to - 3.1%)	0.0% (0.0 to - 0.1%)	-0.1% (-0.1 to - 0.1%)	1.1% (0.5 to 1.3%)	1.4% (1.4 to 1.5%)	-0.3% (-1.0 to 0.0%)	-0.1% (-0.3 to - 0.1%)	-2.1% (-3.8 to - 1.2%)	-7.9% (-9.5 to - 6.9%)
Barbados	-2.7% (-4.0 to - 1.6%)	-0.1% (-0.2 to 0.0%)	-17.6% (-38.4 to 0.0%)	-15.8% (-16.3 to - 14.2%)	-0.4% (-0.6 to - 0.2%)	-0.1% (-0.6 to 0.1%)	0.0% (-0.1 to 0.0%)	-0.2% (-0.3 to 0.0%)	2.4% (1.0 to 3.4%)	1.9% (1.9 to 1.6%)	-0.8% (-2.5 to 0.0%)	-0.5% (-1.1 to - 0.3%)	-3.8% (-6.6 to - 1.7%)	-7.2% (-9.4 to - 6.1%)
Bermuda	0.2% (-0.2 to 0.8%)	-0.8% (-1.5 to - 0.3%)	0.0% (0.0 to 0.0%)	5.3% (2.5 to 8.2%)	-0.1% (-0.1 to - 0.1%)	-0.3% (-0.6 to - 0.2%)	0.4% (0.0 to 1.8%)	-0.1% (-0.2 to - 0.1%)	1.2% (0.5 to 2.1%)	0.9% (1.0 to 0.8%)	-0.6% (-2.1 to 0.0%)	-0.2% (-0.5 to - 0.1%)	-1.8% (-3.5 to - 0.8%)	-3.1% (-3.2 to - 3.3%)
Qatar	0.0% (1.3 to 0.2%)	0.0% (-0.1 to 0.0%)	-31.5% (-68.2 to 0.0%)	-40.1% (-45.3 to - 33.6%)	-0.7% (-1.0 to - 0.4%)	-0.6% (-1.1 to - 0.6%)	0.1% (0.0 to 0.6%)	-2.3% (-3.4 to - 1.4%)	6.0% (2.5 to 11.2%)	4.8% (5.1 to 3.9%)	-3.2% (-8.5 to - 0.2%)	-2.3% (-4.3 to - 1.6%)	-15.4% (-13.7 to -11.8%)	-22.1% (-21.2 to - 18.9%)
Slovenia	-1.6% (-1.2 to - 1.6%)	-2.4% (-3.8 to - 1.4%)	-12.6% (-25.0 to 0.0%)	-9.5% (-8.6 to - 9.5%)	-0.1% (-0.1 to - 0.1%)	-4.2% (-5.3 to - 3.4%)	0.2% (0.0 to 0.8%)	0.3% (0.2 to 0.1%)	1.8% (0.7 to 3.1%)	0.8% (0.9 to 0.8%)	-0.9% (-2.9 to 0.0%)	-0.3% (-0.7 to - 0.1%)	-5.2% (-7.8 to - 2.7%)	-14.2% (-16.2 to - 13.0%)
Israel	-1.9% (-0.2 to - 1.8%)	-0.3% (-0.5 to - 0.1%)	-18.5% (-37.9 to 0.0%)	-22.9% (-25.7 to - 19.5%)	0.0% (0.0 to 0.0%)	-4.1% (-4.6 to - 3.3%)	0.4% (0.0 to 2.1%)	-0.2% (-0.3 to - 0.1%)	1.2% (0.4 to 1.8%)	1.9% (1.9 to 1.9%)	-0.4% (-1.8 to 0.0%)	-0.2% (-0.4 to - 0.1%)	-3.4% (-4.4 to - 1.9%)	-8.6% (-8.0 to - 8.2%)
Bosnia and Herzegov ina	3.7% (1.8 to 5.8%)	-11.6% (-14.0 to - 9.0%)	-9.0% (-18.3 to 0.0%)	8.5% (5.4 to 11.9%)	-0.4% (-0.6 to - 0.3%)	1.8% (1.4 to 2.2%)	0.0% (0.0 to 0.6%)	-0.1% (-0.3 to 0.1%)	2.2% (1.0 to 4.1%)	2.3% (2.6 to 1.8%)	-1.9% (-5.3 to - 0.2%)	-0.7% (-1.4 to - 0.4%)	-8.2% (-12.2 to -4.8%)	-17.1% (-19.4 to - 14.4%)

Location Name	Ambient pollution	House-hold air pollution	Low Hib vaccine coverage	Low pneumo-coccal vaccine coverage	No hand-washing	Second-hand smoke	Zinc deficiency	Breast-feeding	Low antibiotic coverage	Low birth weight and short gestation	Stunting	Under-weight	Wasting	All risk factors
Croatia	-0.6% (-0.5 to -0.9%)	-2.3% (-3.6 to -1.4%)	-12.0% (-24.1 to 0.0%)	3.9% (1.7 to 6.0%)	-0.1% (-0.1 to -0.1%)	-5.4% (-6.4 to -4.5%)	-0.3% (-1.2 to 0.0%)	0.3% (0.2 to 0.4%)	1.5% (0.6 to 2.3%)	1.2% (1.3 to 0.9%)	-0.7% (-2.8 to 0.0%)	-0.3% (-0.6 to -0.1%)	-4.2% (-6.2 to -2.3%)	-13.4% (-15.3 to -11.9%)
Taiwan	1.8% (0.0 to 2.1%)	-6.6% (-9.7 to -4.1%)	-14.4% (-30.3 to 0.0%)	-19.2% (-21.4 to -16.1%)	-0.6% (-0.9 to -0.4%)	-5.7% (-7.0 to -4.3%)	0.5% (0.0 to 2.4%)	-0.4% (-0.8 to -0.2%)	2.4% (0.9 to 4.2%)	-0.1% (-0.1 to -0.2%)	-1.0% (-3.1 to -0.1%)	-0.4% (-0.9 to -0.2%)	-8.2% (-9.8 to -5.0%)	-21.0% (-22.2 to -19.0%)
Northern Mariana Islands	-0.3% (0.8 to -0.8%)	-3.4% (-4.7 to -2.1%)	0.0% (0.0 to 0.0%)	7.1% (4.1 to 10.3%)	-0.2% (-0.3 to -0.1%)	-0.2% (-0.7 to 0.1%)	-0.5% (-2.6 to 0.0%)	-0.2% (-0.4 to -0.1%)	1.8% (0.8 to 3.4%)	0.5% (0.5 to 0.3%)	0.0% (-0.3 to 0.0%)	-0.1% (-0.1 to 0.0%)	-1.5% (-2.6 to -0.4%)	-6.7% (-8.9 to -5.9%)
Cyprus	-1.4% (-1.2 to -1.4%)	-0.5% (-0.9 to -0.2%)	-13.1% (-26.1 to 0.0%)	-10.4% (-10.9 to -9.4%)	0.0% (-0.1 to 0.0%)	-0.8% (-0.7 to -0.6%)	0.0% (0.0 to 0.0%)	-0.1% (-0.2 to 0.0%)	1.3% (0.7 to 1.8%)	3.0% (3.6 to 2.2%)	-0.4% (-1.5 to 0.0%)	-0.2% (-0.5 to -0.1%)	-2.7% (-3.7 to -1.4%)	-3.6% (-4.9 to -2.1%)
United Arab Emirates	1.5% (0.5 to 3.4%)	-1.3% (-2.2 to -0.7%)	-20.0% (-42.1 to 0.0%)	-25.3% (-27.8 to -21.6%)	-1.1% (-1.6 to -0.7%)	-2.1% (-2.8 to -1.5%)	-0.7% (-3.0 to 0.0%)	-0.6% (-0.9 to -0.5%)	4.5% (1.8 to 7.5%)	2.3% (2.2 to 2.0%)	-3.4% (-9.2 to -0.5%)	-1.0% (-2.0 to -0.6%)	-23.2% (-21.8 to -19.5%)	-31.5% (-27.7 to -33.1%)
Czech Republic	-3.5% (-3.8 to -2.5%)	-0.5% (-0.9 to -0.3%)	-14.3% (-29.5 to 0.0%)	8.3% (5.0 to 12.0%)	-0.1% (-0.1 to -0.1%)	-1.6% (-2.1 to -1.3%)	0.0% (0.0 to 0.1%)	0.0% (0.0 to 0.0%)	1.7% (0.6 to 2.9%)	4.3% (4.4 to 4.0%)	-0.3% (-0.9 to 0.0%)	-0.3% (-0.6 to -0.1%)	-7.0% (-6.7 to -4.9%)	-10.1% (-9.4 to -8.2%)
New Zealand	-1.1% (-0.8 to -0.2%)	-0.2% (-0.5 to -0.1%)	-14.3% (-28.9 to 0.0%)	-15.6% (-17.0 to -13.5%)	-0.1% (-0.2 to -0.1%)	-1.4% (-1.6 to -0.9%)	-0.1% (-0.3 to 0.0%)	0.0% (0.0 to 0.0%)	1.2% (0.5 to 1.6%)	1.4% (1.4 to 1.2%)	-0.4% (-1.1 to 0.0%)	-0.2% (-0.4 to -0.1%)	-2.6% (-4.7 to -1.4%)	-4.7% (-5.9 to -3.9%)
South Korea	-1.2% (-1.0 to -1.2%)	-0.4% (-0.7 to -0.2%)	-11.9% (-23.6 to 0.0%)	-14.9% (-16.9 to -12.5%)	-0.2% (-0.2 to -0.1%)	-5.1% (-6.2 to -4.1%)	-0.2% (-0.6 to 0.0%)	-0.1% (-0.2 to -0.1%)	1.7% (0.5 to 2.8%)	0.8% (0.9 to 0.7%)	-0.3% (-1.2 to 0.0%)	-0.2% (-0.5 to -0.1%)	-1.9% (-3.1 to -1.1%)	-8.9% (-10.7 to -7.4%)
Puerto Rico	0.1% (-0.2 to 0.6%)	0.0% (0.0 to 0.0%)	-13.0% (-27.0 to 0.0%)	-11.9% (-11.6 to -10.9%)	-0.3% (-0.6 to -0.2%)	-0.1% (-0.1 to 0.0%)	-0.1% (-0.2 to 0.0%)	-0.1% (-0.3 to 0.0%)	1.2% (0.5 to 1.7%)	2.1% (2.2 to 1.4%)	-0.6% (-2.1 to 0.0%)	-0.2% (-0.4 to -0.1%)	-2.3% (-3.4 to -1.1%)	-2.1% (-3.1 to -1.1%)
Singapore	0.6% (0.3 to 0.3%)	-0.7% (-1.4 to -0.3%)	-16.4% (-33.6 to 0.0%)	-17.0% (-18.1 to -15.0%)	-0.2% (-0.3 to -0.1%)	-2.1% (-2.8 to -1.4%)	0.1% (0.0 to 0.8%)	-1.4% (-1.7 to -1.1%)	2.1% (0.8 to 3.9%)	2.5% (2.4 to 3.0%)	-0.5% (-1.2 to 0.0%)	-0.7% (-1.6 to -0.4%)	-8.0% (-10.6 to -4.6%)	-10.9% (-13.2 to -8.1%)
Virgin Islands, U.S.	0.1% (-0.2 to 0.8%)	-0.6% (-1.2 to -0.2%)	-9.6% (-18.8 to 0.0%)	-6.9% (-4.0 to -8.2%)	-0.4% (-0.6 to -0.3%)	-0.1% (-0.2 to 0.0%)	0.0% (0.0 to 0.4%)	-0.1% (-0.4 to 0.1%)	2.7% (1.2 to 4.7%)	2.1% (2.9 to 1.6%)	-0.7% (-2.3 to 0.0%)	-0.2% (-0.5 to -0.1%)	-2.4% (-4.9 to -0.8%)	-2.9% (-5.2 to -1.4%)

Location Name	Ambient pollution	House-hold air pollution	Low Hib vaccine coverage	Low pneumo-coccal vaccine coverage	No hand-washing	Second-hand smoke	Zinc deficiency	Breast-feeding	Low antibiotic coverage	Low birth weight and short gestation	Stunting	Under-weight	Wasting	All risk factors
Saint Lucia	3.8% (1.9 to 5.5%)	-12.7% (-17.0 to -9.0%)	-13.7% (-29.0 to 0.0%)	7.8% (4.6 to 11.2%)	-1.2% (-1.6 to -0.7%)	-0.1% (0.0 to -0.1%)	-0.1% (-1.1 to 0.0%)	0.0% (0.3 to -0.1%)	1.9% (0.7 to 3.1%)	1.6% (1.8 to 1.5%)	-1.5% (-5.1 to 0.0%)	-1.4% (-2.8 to -0.8%)	-8.6% (-14.0 to -4.7%)	-22.6% (-26.8 to -19.7%)
Bahrain	1.2% (1.0 to 1.4%)	-0.5% (-0.9 to -0.3%)	-18.8% (-39.2 to 0.0%)	-24.2% (-27.8 to -20.0%)	-0.1% (-0.1 to -0.1%)	-1.4% (-1.8 to -0.9%)	0.0% (0.0 to 0.2%)	-0.4% (-0.9 to -0.2%)	3.2% (1.3 to 5.5%)	3.5% (3.6 to 3.4%)	-2.7% (-8.0 to -0.2%)	-0.7% (-1.4 to -0.7%)	-20.4% (-24.7 to -14.2%)	-22.6% (-25.1 to -20.3%)
Cuba	-0.7% (-1.6 to -0.6%)	-2.0% (-3.2 to -1.1%)	-14.6% (-30.7 to 0.0%)	8.9% (5.4 to 12.6%)	-0.9% (-1.2 to -0.5%)	-3.3% (-5.2 to -1.9%)	0.0% (-0.2 to 0.0%)	-0.1% (-0.4 to -0.1%)	0.5% (0.4 to 0.4%)	2.4% (2.3 to 2.5%)	0.1% (0.0 to 0.1%)	-0.2% (-0.3 to -0.1%)	1.0% (0.1 to 3.5%)	-4.3% (-2.6 to -4.7%)
Trinidad and Tobago	-1.5% (-1.7 to -0.6%)	-1.0% (-1.8 to -0.5%)	-9.4% (-19.7 to 0.0%)	-7.1% (-6.2 to -7.5%)	-0.6% (-0.7 to -0.4%)	-0.8% (-1.0 to -0.5%)	-0.2% (-0.9 to 0.0%)	-0.4% (-0.5 to -0.1%)	1.1% (0.5 to 1.7%)	0.9% (1.5 to 0.8%)	-0.4% (-1.8 to 0.0%)	-0.7% (-1.5 to -0.3%)	-5.3% (-7.6 to -2.6%)	-10.8% (-11.2 to -9.5%)
Saint Vincent and the Grenadines	5.4% (2.3 to 8.8%)	-21.8% (-27.8 to -16.1%)	-15.7% (-34.1 to 0.0%)	7.3% (4.1 to 10.6%)	-1.5% (-2.0 to -0.9%)	-1.0% (-1.5 to -0.6%)	-0.3% (-1.5 to 0.0%)	-0.2% (-0.5 to -0.1%)	1.7% (0.8 to 2.8%)	1.5% (1.5 to 1.2%)	-2.0% (-6.0 to -0.1%)	-1.8% (-3.9 to -1.1%)	-8.0% (-10.7 to -4.6%)	-33.2% (-37.5 to -31.4%)
Poland	-1.2% (-2.1 to -1.3%)	-3.5% (-5.3 to -2.0%)	-12.5% (-25.3 to 0.0%)	5.2% (2.7 to 7.8%)	-0.2% (-0.3 to -0.1%)	-3.2% (-3.8 to -2.6%)	-0.1% (-0.5 to 0.0%)	-0.1% (-0.3 to 0.1%)	1.9% (0.8 to 3.6%)	2.4% (2.7 to 2.2%)	-1.4% (-4.1 to -0.1%)	-0.5% (-1.0 to -0.3%)	-6.6% (-9.3 to -3.8%)	-15.4% (-18.2 to -13.0%)
Portugal	-0.7% (0.8 to -1.5%)	-2.4% (-4.2 to -1.2%)	-12.5% (-25.0 to 0.0%)	-13.8% (-14.3 to -12.3%)	-0.1% (-0.2 to -0.1%)	-2.1% (-2.5 to -1.6%)	0.0% (0.1 to 0.0%)	0.0% (0.1 to 0.1%)	1.5% (0.4 to 2.1%)	1.8% (1.8 to 1.7%)	-0.5% (-1.6 to 0.0%)	-0.4% (-0.8 to -0.2%)	-3.7% (-6.0 to -1.8%)	-8.4% (-10.0 to -6.7%)
Hungary	-0.7% (-0.6 to -1.1%)	-4.2% (-5.7 to -2.8%)	-13.4% (-27.6 to 0.0%)	-13.9% (-16.1 to -11.5%)	-0.1% (-0.2 to -0.1%)	-3.2% (-3.6 to -2.8%)	0.1% (0.0 to 0.5%)	0.2% (0.2 to 0.0%)	1.7% (0.7 to 2.5%)	4.0% (4.6 to 3.8%)	-1.1% (-2.7 to -0.1%)	-0.4% (-1.0 to -0.2%)	-6.1% (-9.1 to -3.6%)	-13.1% (-15.8 to -10.6%)
American Samoa	1.6% (0.3 to 2.9%)	-7.2% (-9.7 to -5.2%)	0.0% (0.0 to 0.0%)	6.2% (3.2 to 9.4%)	-1.0% (-1.5 to -0.6%)	-0.4% (-0.6 to -0.2%)	0.3% (0.0 to 1.4%)	-0.3% (-0.5 to -0.2%)	1.9% (0.7 to 3.4%)	1.0% (1.2 to 0.7%)	-0.8% (-3.4 to 0.0%)	-0.4% (-0.7 to -0.3%)	-5.6% (-9.0 to -3.4%)	-14.6% (-17.3 to -12.3%)
The Bahamas	-1.2% (-1.2 to -0.8%)	-2.3% (-4.0 to -1.3%)	-17.4% (-37.9 to 0.0%)	-19.0% (-20.6 to -16.6%)	-0.3% (-0.3 to -0.1%)	-1.2% (-1.7 to -0.7%)	0.0% (0.0 to 0.0%)	-0.3% (-0.4 to -0.2%)	2.1% (0.9 to 3.2%)	1.5% (1.9 to 1.4%)	-0.8% (-3.0 to 0.0%)	-0.3% (-0.8 to -0.2%)	-3.1% (-4.9 to -1.4%)	-9.0% (-11.4 to -7.8%)
Uruguay	-0.8% (0.2 to -1.1%)	-1.4% (-2.3 to -0.6%)	-14.9% (-31.3 to 0.0%)	-18.4% (-20.4 to -15.8%)	-0.5% (-0.6 to -0.3%)	-3.8% (-4.8 to -2.9%)	0.1% (0.0 to 0.4%)	-0.4% (-1.0 to -0.2%)	1.0% (0.6 to 1.9%)	2.5% (2.6 to 2.5%)	-1.6% (-3.8 to -0.1%)	-0.9% (-1.8 to -0.6%)	-9.8% (-11.1 to -6.0%)	-17.0% (-17.5 to -15.4%)

Location Name	Ambient pollution	House-hold air pollution	Low Hib vaccine coverage	Low pneumo-coccal vaccine coverage	No hand-washing	Second-hand smoke	Zinc deficiency	Breast-feeding	Low antibiotic coverage	Low birth weight and short gestation	Stunting	Under-weight	Wasting	All risk factors
Montenegro	-1.1% (-1.9 to -0.7%)	-2.9% (-3.9 to -2.1%)	-11.3% (-22.4 to 0.0%)	3.6% (1.5 to 5.8%)	-0.1% (-0.1 to -0.1%)	2.4% (1.8 to 2.8%)	0.0% (0.0 to 0.0%)	-0.1% (-0.2 to 0.0%)	2.0% (0.7 to 3.7%)	1.7% (2.0 to 1.5%)	-0.6% (-1.6 to 0.0%)	-0.3% (-0.6 to -0.1%)	-6.1% (-9.0 to -3.3%)	-8.9% (-12.2 to -6.5%)
Grenada	3.9% (1.3 to 5.2%)	-12.0% (-16.4 to -8.0%)	-13.5% (-28.6 to 0.0%)	6.7% (3.7 to 9.8%)	-1.6% (-2.2 to -1.1%)	-0.6% (-0.8 to -0.4%)	-0.3% (-1.5 to 0.0%)	-0.2% (-0.5 to 0.1%)	2.0% (0.9 to 3.1%)	0.8% (0.9 to 0.9%)	-2.2% (-6.5 to -0.1%)	-2.0% (-4.0 to -1.2%)	-8.9% (-14.3 to -4.8%)	-25.6% (-29.6 to -23.4%)
Slovakia	-2.7% (-2.5 to -2.3%)	-0.8% (-1.4 to -0.4%)	-13.8% (-28.6 to 0.0%)	-16.6% (-19.1 to -13.7%)	-0.1% (-0.2 to -0.1%)	-3.8% (-4.7 to -3.0%)	0.1% (0.0 to 0.6%)	0.0% (-0.1 to 0.1%)	1.5% (0.6 to 2.9%)	1.2% (1.2 to 1.3%)	-1.2% (-3.8 to -0.1%)	-0.4% (-0.8 to -0.2%)	-5.7% (-8.7 to -3.0%)	-14.5% (-17.0 to -12.6%)
Tonga	1.2% (0.4 to 2.9%)	-17.0% (-21.4 to -13.0%)	-18.6% (-40.5 to 0.0%)	6.7% (3.4 to 10.1%)	-1.8% (-2.4 to -1.1%)	-1.3% (-1.8 to -0.8%)	-0.2% (-0.8 to 0.0%)	-0.3% (-0.6 to -0.2%)	1.6% (0.6 to 2.8%)	1.3% (1.1 to 1.4%)	-2.1% (-6.1 to -0.1%)	-1.2% (-2.5 to -0.7%)	-12.4% (-18.9 to -8.7%)	-36.5% (-42.9 to -32.4%)
Jamaica	1.4% (-0.1 to 3.0%)	-8.7% (-11.7 to -6.3%)	-14.2% (-30.1 to 0.0%)	-3.7% (-2.5 to -4.6%)	-1.0% (-1.4 to -0.6%)	-0.5% (-0.4 to -0.5%)	-0.2% (-1.0 to 0.0%)	-0.4% (-0.7 to -0.2%)	1.7% (0.8 to 3.1%)	0.4% (0.5 to 0.3%)	-1.2% (-3.7 to -0.1%)	-1.1% (-2.3 to -0.8%)	-13.7% (-15.9 to -8.7%)	-25.0% (-27.2 to -21.8%)
Fiji	1.3% (0.0 to 3.9%)	-16.9% (-20.4 to -13.9%)	-19.5% (-42.9 to 0.0%)	-20.8% (-22.4 to -18.2%)	-2.3% (-3.2 to -1.3%)	-0.5% (-0.7 to -0.2%)	-0.2% (-0.9 to 0.0%)	-0.4% (-0.7 to -0.1%)	1.5% (0.9 to 2.7%)	0.8% (0.8 to 1.2%)	-0.6% (-2.8 to 0.0%)	-1.4% (-2.7 to -1.0%)	-17.1% (-14.2 to -13.4%)	-40.7% (-37.4 to -40.1%)
Samoa	-0.3% (0.1 to -0.3%)	-7.2% (-8.1 to -5.1%)	-13.4% (-28.7 to 0.0%)	7.7% (3.8 to 11.6%)	-0.8% (-1.1 to -0.5%)	-3.0% (-3.7 to -2.3%)	-0.2% (-0.8 to 0.0%)	-0.1% (-0.1 to 0.0%)	2.1% (0.8 to 3.9%)	1.5% (1.5 to 1.1%)	0.0% (0.3 to 0.0%)	-0.2% (-0.6 to -0.1%)	-3.0% (-2.2 to -1.8%)	-15.2% (-15.5 to -14.0%)
Argentina	-0.5% (-1.1 to -0.7%)	-3.3% (-4.6 to -2.1%)	-14.8% (-30.5 to 0.0%)	-17.6% (-19.0 to -15.5%)	-2.4% (-3.2 to -1.5%)	-2.8% (-3.6 to -2.2%)	0.0% (-0.3 to 0.0%)	0.0% (0.2 to -0.2%)	1.5% (0.5 to 2.5%)	1.9% (1.8 to 1.9%)	-0.9% (-1.8 to -0.1%)	-1.3% (-2.8 to -0.9%)	11.9% (7.6 to 20.8%)	-0.6% (5.9 to -4.4%)
Greenland	-1.9% (-1.6 to -1.5%)	-0.3% (-0.6 to -0.1%)	-13.5% (-27.8 to 0.0%)	-16.8% (-18.8 to -14.3%)	-0.1% (-0.1 to 0.0%)	-4.0% (-4.9 to -3.0%)	-0.2% (-0.4 to 0.0%)	0.1% (0.1 to 0.0%)	1.2% (0.5 to 2.3%)	2.3% (2.3 to 2.1%)	-0.5% (-2.5 to 0.0%)	-0.3% (-0.6 to -0.1%)	-2.8% (-5.7 to -1.4%)	-7.8% (-10.6 to -6.0%)
Guyana	2.2% (1.2 to 3.5%)	-6.5% (-9.1 to -4.2%)	-15.8% (-34.0 to 0.0%)	-16.8% (-18.7 to -14.2%)	1.2% (0.9 to 1.5%)	-0.7% (-0.9 to -0.5%)	-0.6% (-2.7 to 0.0%)	-0.6% (-1.1 to -0.3%)	1.7% (0.7 to 2.8%)	2.0% (1.0 to 1.9%)	-1.3% (-4.2 to -0.1%)	-3.7% (-7.0 to -2.6%)	-20.6% (-23.3 to -16.2%)	-30.3% (-33.1 to -27.8%)
Serbia	0.5% (0.3 to 0.4%)	-5.0% (-6.3 to -3.8%)	-11.1% (-21.9 to 0.0%)	3.0% (1.0 to 4.9%)	-0.3% (-0.4 to -0.2%)	-1.0% (-1.3 to -0.7%)	0.0% (-0.1 to 0.0%)	0.0% (-0.1 to 0.1%)	2.8% (1.1 to 3.7%)	1.8% (1.8 to 1.7%)	-1.2% (-3.8 to -0.1%)	-0.4% (-0.9 to -0.2%)	-6.7% (-8.6 to -4.7%)	-13.8% (-15.7 to -12.2%)

Location Name	Ambient pollution	House-hold air pollution	Low Hib vaccine coverage	Low pneumo-coccal vaccine coverage	No hand-washing	Second-hand smoke	Zinc deficiency	Breast-feeding	Low antibiotic coverage	Low birth weight and short gestation	Stunting	Under-weight	Wasting	All risk factors
Suriname	1.8% (1.0 to 1.8%)	-8.3% (-11.4 to -5.1%)	-14.9% (-31.5 to 0.0%)	-4.3% (1.2 to -8.1%)	-1.2% (-1.8 to -0.8%)	0.6% (0.4 to 0.8%)	-0.1% (-0.5 to 0.0%)	0.0% (0.0 to 0.1%)	0.1% (0.1 to -0.1%)	2.6% (2.0 to 2.3%)	-1.7% (-5.4 to 0.0%)	-1.9% (-3.7 to -1.2%)	-8.1% (-10.8 to -5.2%)	-18.6% (-20.7 to -17.5%)
Marshall Islands	0.7% (0.2 to 1.4%)	-9.6% (-12.3 to -7.2%)	-7.5% (-15.4 to 0.0%)	-5.8% (-4.2 to -6.9%)	-2.8% (-3.8 to -1.8%)	-0.7% (-1.0 to -0.4%)	-0.1% (-0.4 to 0.0%)	-0.3% (-0.4 to -0.2%)	2.3% (0.9 to 4.4%)	1.3% (1.3 to 1.0%)	-2.6% (-6.8 to -0.1%)	-2.5% (-4.9 to -1.5%)	-13.5% (-18.7 to -9.6%)	-33.6% (-38.2 to -29.8%)
Macedonia	0.3% (0.0 to -0.8%)	-5.7% (-7.4 to -3.9%)	-10.8% (-21.3 to 0.0%)	1.7% (0.0 to 3.3%)	-0.2% (-0.3 to -0.1%)	-0.1% (-0.3 to -0.1%)	-0.1% (-0.4 to 0.0%)	0.0% (-0.3 to 0.3%)	0.8% (0.6 to 1.1%)	0.9% (1.0 to 0.7%)	-1.0% (-3.4 to 0.0%)	-0.2% (-0.5 to -0.1%)	-2.4% (-2.8 to -1.5%)	-11.6% (-12.8 to -10.4%)

Supplementary Figure. Maps of the Socio-demographic Index by country in 1990, 2017, and the difference between 1990-2017. The difference shown is in absolute terms. The SDI is scaled between 0 and 1 so the difference is simply the difference in the value in 2017 and 1990. A) The SDI value in 1990; B) The SDI value in 2017; and C) The difference in SDI between 1990 and 2017.

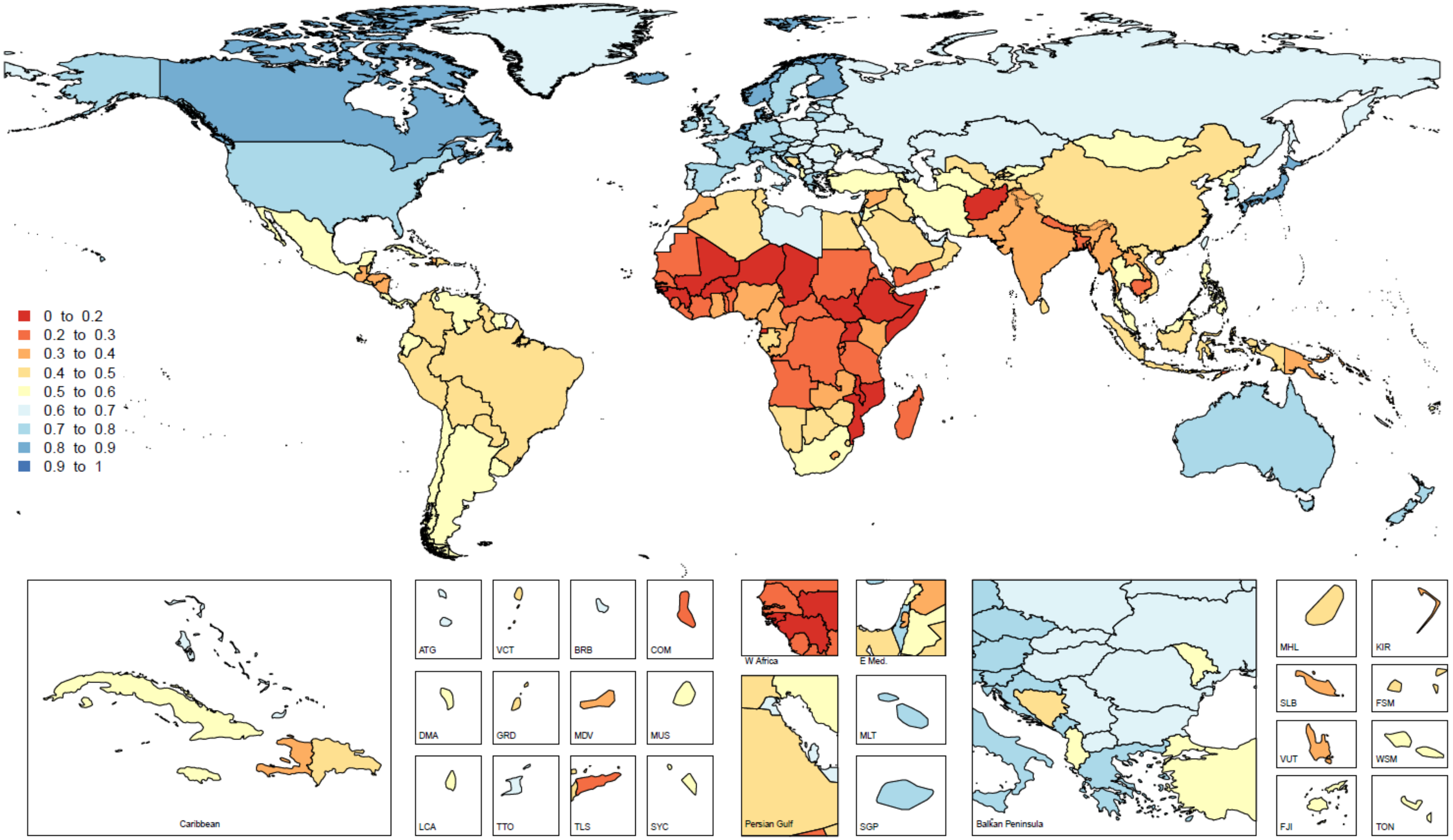

A)

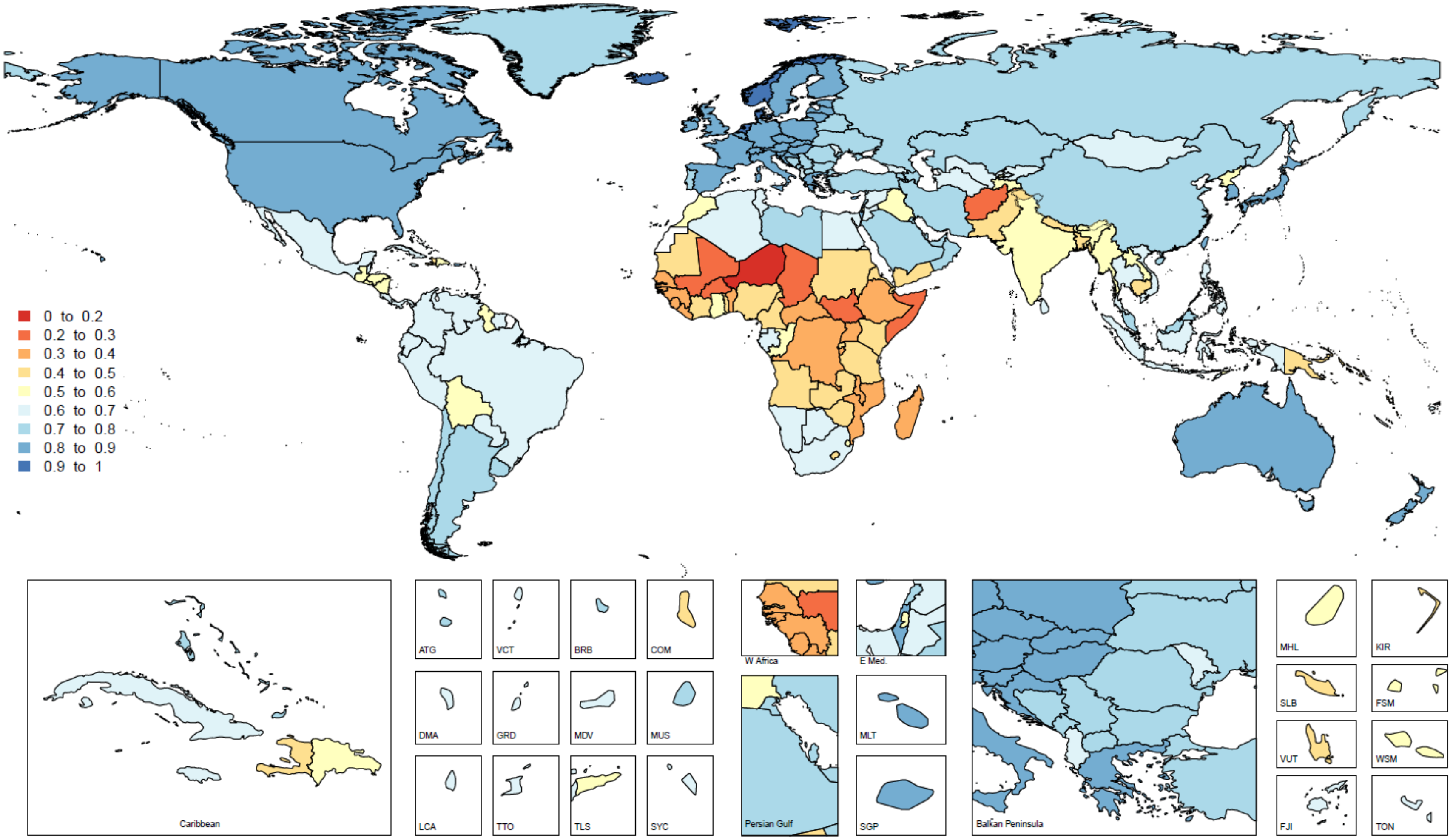
0 to 0.2
0.2 to 0.3
0.3 to 0.4
0.4 to 0.5
0.5 to 0.6
0.6 to 0.7
0.7 to 0.8
0.8 to 0.9
0.9 to 1
Caribbean
ATG
VCT
BRB
COM
DMA
GRD
MDV
MUS
LCA
TTO
TLS
SYC
W Africa
E Med.
Persian Gulf
MLT
SGP
Balkan Peninsula
MHL
KIR
SLB
FSM
VUT
WSM
FJI
TON

B)

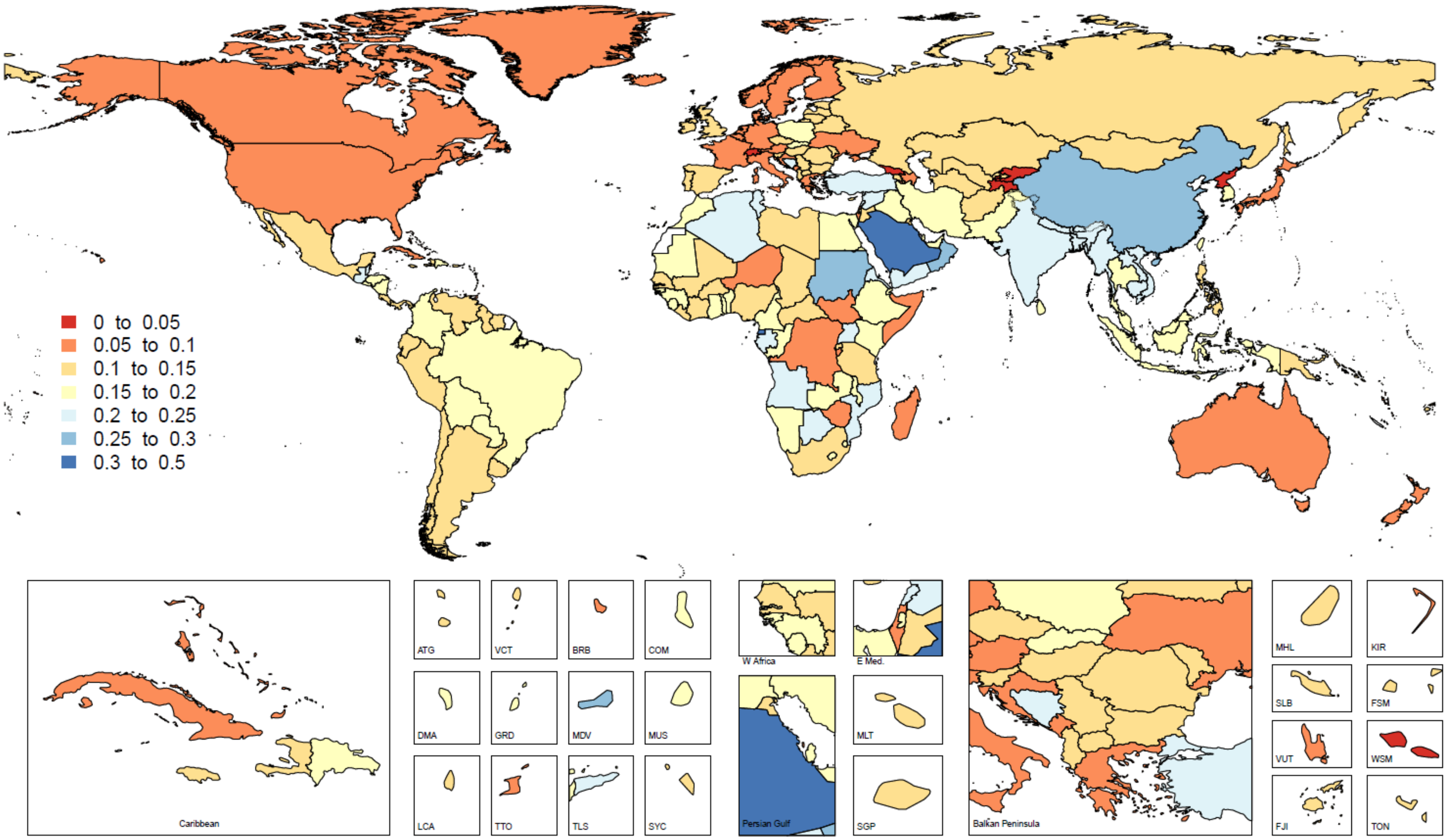
0 to 0.05
0.05 to 0.1
0.1 to 0.15
0.15 to 0.2
0.2 to 0.25
0.25 to 0.3
0.3 to 0.5
Caribbean
ATG
VCT
BRB
COM
DMA
GRD
MDV
MUS
LCA
TTO
TLS
SYC
W Africa
E Med.
Persian Gulf
MLT
SGP
Balkan Peninsula
MHL
KIR
SLB
FSM
VUT
WSM
FJI
TON

C)
